# Application of the Horner–Wadsworth–Emmons Olefination in the Construction of *E*‑α,β-Unsaturated β‑Boryl Nitriles

**DOI:** 10.1021/acs.joc.4c02915

**Published:** 2025-05-21

**Authors:** Kaja Gosak, Martin Črnugelj, Zdenko Časar

**Affiliations:** a Lek Pharmaceuticals d.d., Sandoz Development Center Slovenia, Verovškova ulica 57, 1526 Ljubljana, Slovenia; b University of Ljubljana, Faculty of Chemistry and Chemical Technology, Večna pot 113, SI-1000 Ljubljana, Slovenia; c University of Ljubljana, Faculty of Pharmacy, Aškerčeva cesta 7, SI-1000 Ljubljana, Slovenia

## Abstract

Herein, we describe a simple transition-metal-free synthesis of *E*-α,β-unsaturated β-boryl nitriles by the Horner–Wadsworth–Emmons reaction starting from potassium acyltrifluoroborates. The methodology encompasses a broad range of alkyl and aryl substrates. The reaction conditions are simple, and the reaction products are in most cases isolated with good yields as pure *E* isomers without column chromatography.

## Graphical abstract

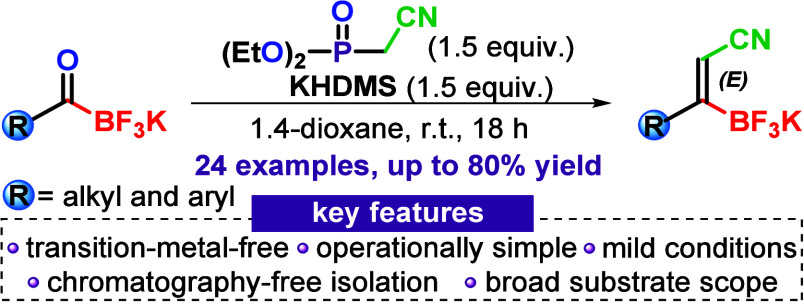

Organonitriles^1^ and organoboron compounds^2^ represent some of the most useful reagent classes and building blocks in organic synthesis. In this context, research on the preparation of alkenyl boronates (Scheme 1A) has gained momentum due to the plethora of transformation options available at either the boron moiety or the carbon–carbon double bond.^3^ Their proven utility as reagents in various transformations, including Suzuki–Miyaura cross-coupling, Diels–Alder reactions, asymmetric carbon–carbon double bond reductions, and others, further underscores their significance.^3,4^ Selective preparation of trisubstituted alkenyl boronates heavily relies on transition-metal-catalyzed hydroboration of alkynes.^3,5^ Other methods for the assembly of trisubstituted alkenyl boronates remain limited,^6^ while transition-metal-free approaches are scarce.^7^ α,β-Unsaturated β-boryl nitriles encompass features of nitriles and alkenyl boronates and are thus valuable building blocks for the construction of other molecules by transformation of the cyano group, boron moiety, or carbon–carbon double bond. Surprisingly, until now, they have been accessible only by three methods. Two of these are palladium-catalyzed approaches, which utilize uncommon ligands and boron precursors above 115 °C and provide opposite stereochemistry (Scheme 1B).^8,9^ The latest method is phosphine-catalyzed hydroboration of cyanoacetylene derivatives, performed under cryogenic conditions in dichloromethane (Scheme 1B).^10^ This fact attracted our attention and stimulated us to consider olefination of acylboranes,^11^ which are now easily accessible,^12^ as a viable option for the facile construction of α,β-unsaturated β-boryl nitriles. Interestingly, while the typical reactivity of carbonyl groups in acylboranes was recently confirmed through imine^13^ and heterocycle formation,^14^ to the best of our knowledge, only one literature report describes the olefination of a single “aldehyde type” acylborane derivative.^15^ Therefore, additional examples of carbonyl group transformations in acylboranes would further substantiate the typical characteristics of carbonyl compounds. In this study, we present the discovery of a simple method for the transition-metal-free assembly of *E*-α,β-unsaturated β-boryl nitriles. This is achieved using the Horner–Wadsworth–Emmons (HWE) reaction^16,17^ between potassium acyltrifluoroborates (KATs) and diethyl (cyanomethyl)­phosphonate, which provides pure *E* products with mostly good yields, without the need for chromatography (Scheme 1C).

**Scheme 1:**
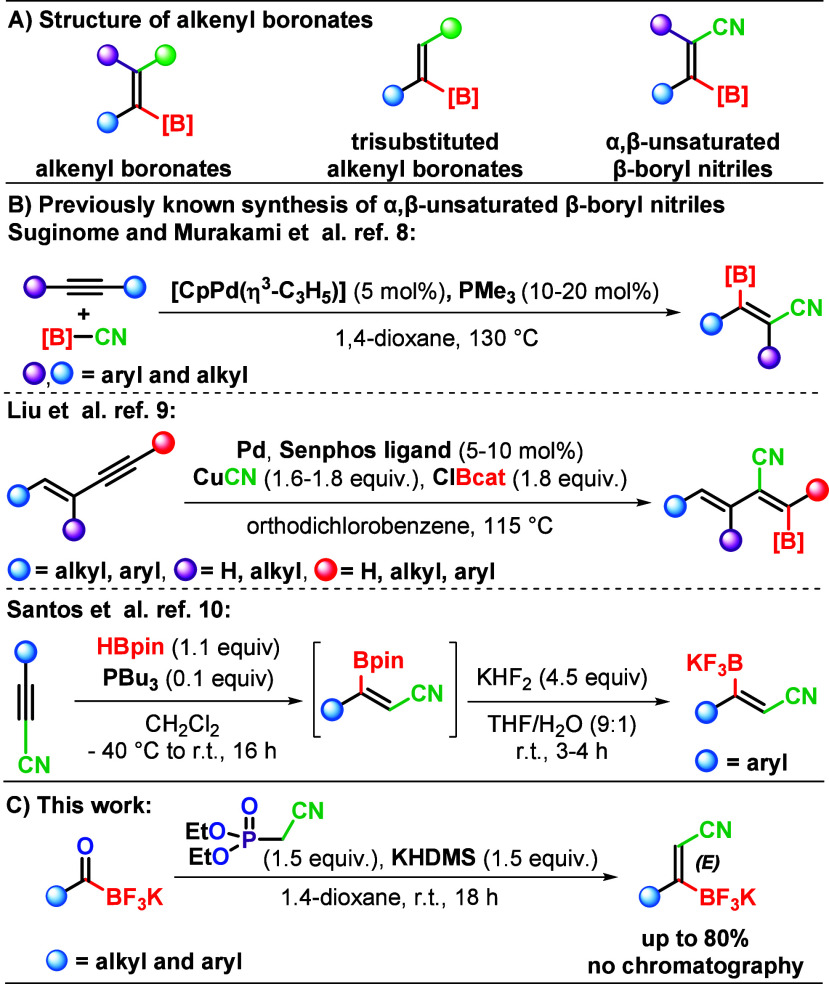
Background and Context

At the outset of our investigation, we considered the HWE reaction as an olefination option using KATs **1** as the substrates. This choice was driven by the potentially easier separation of phosphorus byproducts and the higher nucleophilicity of phosphonate carbanions compared to Wittig ylides,^16,17^ which might facilitate the olefination with sterically more demanding KAT substrates **1** compared to the “aldehyde type” acylborane used by Erker.^15^ Namely, from a steric effect perspective, KATs **1** may resemble ketones, which are known to be more challenging substrates for HWE olefination due to their lower reactivity and stereoselectivity.^16,18^ We began our investigation by testing different phosphonate precursors, such as diethyl cyanomethylphosphonate (DECMP) and diphenyl cyanomethylphosphonate (DPCMP), in an olefination reaction with *p*-bromophenyl KAT **1a**. Our initial results using K_2_CO_3_ as a base demonstrated that DECMP yielded notably superior conversion outcomes compared to DPCMP (this effect was also observed when other bases were used; see entry 1 vs entry 2 of Table S1 and entries 14–16 vs 17–19). This suggests that steric factors play an important role in the olefination of compound **1a**. Therefore, DECMP was selected for further optimization of the olefination (Table 1).

**Table 1:**
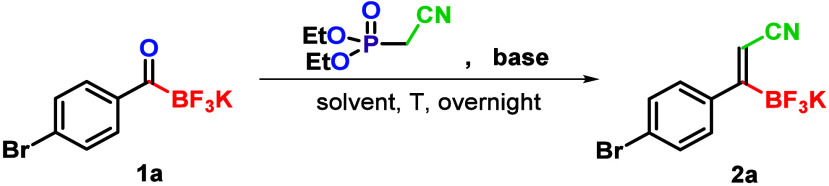
Reaction Screening and Optimization^a^

entry	base (equiv)	solvent	*T* (°C)	*Z*-**2** (%)^e^	*E*-**2** (%)^e^	conversion (%)^f^
1^b^	K_2_CO_3_ (5)	*i*-PrOH	83	39	61	100
2^b^	K_2_CO_3_ (5)	*t*-BuOH	83	25	75	100
3^c^	LiHMDS (1.2)	THF	–20	57	43	100
4^c^	NaHMDS (1.2)	THF	–20	45	55	95
5^c^	KHMDS (1.2)	THF	–20	10	90	100
6^c^,^g^	LiHMDS (1.2)	THF	–20	73	27	100
7^c^,^g^	KHMDS (1.2)	THF	–20	64	36	86
8^c^	LDA (1.2)	THF	–20	49	51	100
9^c^	KHMDS (1.2)	MTBE	–20	11	89	69
10^c^	KHMDS (1.2)	DME	–20	39	61	100
11^c^,^h^	KHMDS (1.2)	dioxane	15	5	95	100
12^d^,^h^	KHMDS (1.5)	dioxane	23	6	94	100

a Reactions were conducted by using **1a** (20 mg, 0.069 mmol) at a concentration of 0.01 g/mL.

b With 2.0 equiv of diethyl (cyanomethyl)­phosphonate.

c With 1.3 equiv of diethyl (cyanomethyl)­phosphonate.

d With 1.5 equiv of diethyl (cyanomethyl)­phosphonate.

e Percentages of formed *E* and *Z* isomers in the reaction mixture after extraction and evaporation (see the Supporting Information) determined by ^1^H NMR and calculated as *I*_*E*‑**2a** or *Z*‑**2a**_/(*I*_*E*‑**2a**_ + *I*_*Z*‑**2a**_) × 100%, where *I*_*E*‑**2a**_ and *I*_*Z*‑**2a**_ are integrals for olefinic protons of *E*-**2** and *Z*-**2**, respectively.

f The conversion was determined by ^1^H NMR and was calculated as (*I*_*Z*‑**2a**_ + *I*_*E*‑**2a**_)/(*I*_*Z*‑**2a**_ + *I*_*E*‑**2a**_ + *I*_**1a**_/2) × 100%, where *I*_*E*‑**2a**_ and *I*_*Z*‑**2a**_ are integrals for olefinic protons of *E*-**2** and *Z*-**2**, respectively, and *I*_**1a**_ is the integral of the *ortho* proton of the aromatic ring (see the Supporting Information).

g With 18-crown-6 (1.5 equiv).

h With 1,4-dioxane.

First, we evaluated the effect of different solvents on the olefination of **1a** with DECMP (2 equiv) using K_2_CO_3_ (5 equiv) as the initial base (see entries 2–9 of Table S1). These experiments showed that in combination with K_2_CO_3_ as a base the reaction best proceeds in refluxing *i*-PrOH or *t*-BuOH, where full conversions were achieved, albeit at moderate stereoselectivity (61% *E* and 39% *Z* (Table 1, entry 1) and 75% *E* and 25% *Z* (Table 1, entry 2)). Next, we evaluated various inorganic bases (3 equiv) in refluxing *i*-PrOH (as a more practical solvent option compared to *t*-BuOH) for the deprotonation of DECMP (2 equiv) in the reaction with **1a**. However, this approach did not result in improved stereoselectivity already achieved with K_2_CO_3_ (see entries 9–13 of Table S1). Therefore, in the next set of experiments, we turned our attention to organic bases such as metal bis­(trimethylsilyl)­amides (MHMDS) and lithium diisopropylamide (LDA) (see entries 14–23 of Table S1). The reaction of **1a** with DECMP (1.3 equiv) in tetrahydrofuran (THF) at −20 °C using LiHMDS and NaHMDS (1.2 equiv) resulted in 100% and 95% conversion, respectively, with very low stereoselectivity (Table 1, entries 3 and 4, respectively). However, LiHMDS favored the formation of *Z*-**2a**. A breakthrough was achieved when KHMDS was used as a base under the same conditions, giving a full conversion and high stereoselectivity in favor of *E*-**2a** (90% *E* and 10% *Z* (Table 1, entry 5)). The application of Still–Gennari conditions by addition of 18-crown-6 (1.5 equiv)^19^ to the reaction mixture amplified the formation of *Z*-**2a** for LiHMDS (27% *E* and 73% *Z* (Table 1, entry 6; compare entries 3 and 6)) and reversed the stereoselectivity in favor of the *Z* stereosiomer for KHMDS (36% *E* and 64% *Z* (Table 1, entry 7; compare entries 5 and 7)). The use of LDA as a base under the same conditions provided full conversion and almost equal amounts of both stereoisomers (Table 1, entry 8). The use of MeTHF or MeCN solvents (see entries 24–30 of Table S1) did not enhance the stereoselectivity compared to the results obtained in THF (Table 1, entry 5), although full conversion was achieved. Reaction in methyl *tert*-butyl ether (MTBE) provided a comparable result with the one in THF in terms of stereoselectivity even though only 69% conversion was obtained (Table 1, entry 9 vs entry 5). Next, the reaction in dimethoxyethane (DME) provided lower stereoselectivities at full conversion (Table 1, entry 10). Finally, when the olefination of **1a** was conducted in 1,4-dioxane using KHMDS as a base at 15 °C, full conversion was achieved along with the highest stereoselectivity in favor of *E*-**2a** (95% *E* and 5% *Z* (Table 1, entry 11)). Conducting the olefination in 1,4-dioxane at a more convenient ambient temperature and with a slightly larger amount of base (1.5 equiv) to ensure better process robustness yielded nearly the same results (94% *E* and 6% *Z* (Table 1, entry 12)).

With the optimal reaction conditions in hand, we evaluated the scope of the olefination transformation at a 300 mg scale of KAT substrates **1** (Scheme 2). For this preparative scale, we developed an isolation procedure based on the precipitation of products *E*-**2** from MeCN with 10% aqueous KHCO_3_ (Table S3), which allowed isolation of pure products *E*-**2a**–*E*-**2q**. Aromatic substrates **1a**–**1i** bearing different electron-withdrawing groups (EWGs) and electron-donating groups (EDGs) on the aromatic ring in general afforded high *E*:*Z* stereoselectivities ranging from 100:0 (for **1b**) to 5.9:1 (for **1e**) and allowed the isolation of pure *E*-**2a**–*E*-**2i** in 63–80% yields using the aforementioned precipitation procedure (Scheme 2A). Interestingly, the stereoselectivity trend observed across aromatic substrates **1a**–**1i** indicates that substrates with EWGs on the aromatic core (**1a**, **1d**, and **1f**–**1i**) generally provide higher stereoselectivities (100:0 to 7.7:1 *E*:*Z*) compared to substrates **1c** and **1e** with EDGs on the aromatic core (8.3:1 to 5.9:1 *E*:*Z*).

**Scheme 2:**
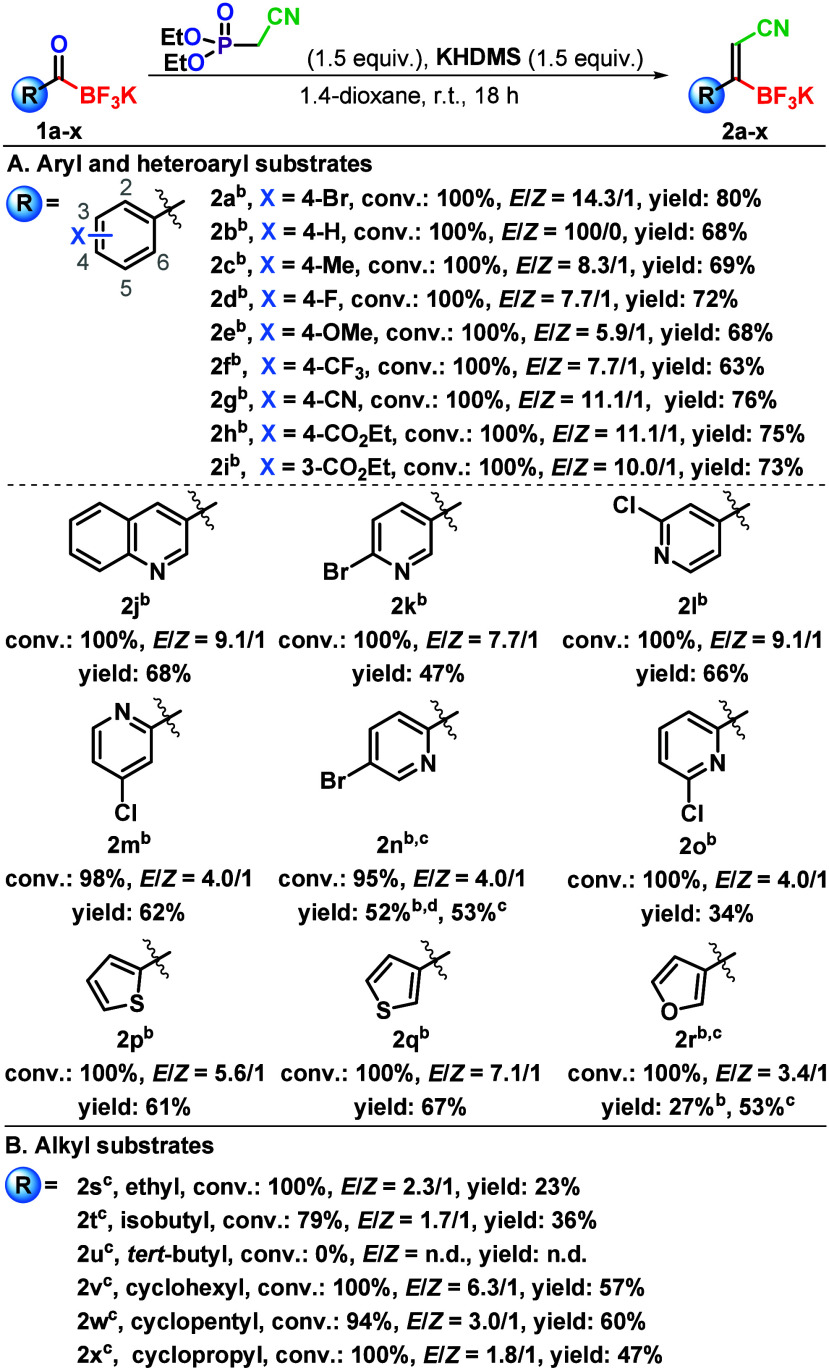
Scope of Horner–Wadsworth–Emmons Olefination of Acyltrifluoroborates **1** ^a^

a All reactions were carried out with 300 mg of substrate **1**. The conversion was determined by ^1^H NMR and was calculated as (*I*_*Z*‑**2a**_ + *I*_*E*‑**2a**_)/(*I*_*Z*‑**2a**_ + *I*_*E*‑**2a**_ + *I*_**1a**_/2) × 100%, where *I*_*E*‑**2a**_ and *I*_*Z*‑**2a**_ are integrals for olefinic protons of *E*-**2** and *Z*-**2**, respectively, and *I*_**1a**_ is the integral of the *ortho* proton of the aromatic ring. The *E*:*Z* ratio of the crude reaction mixture was determined by ^1^H NMR at the end of the reaction time and was calculated as *I*_I_/*I*_2_, where *I*_1_ and *I*_2_ are integrals for olefinic protons of *E*-**2** and *Z* **-2**, respectively. Yields reported for isolated pure *E*-**2** products. n.d. = not determined.
b Isolation using precipitation from a mixture of MeCN and a 10% KHCO_3_ solution.
c Isolation using crystallization from 2-PrOH.
d The product was not pure and contained ca. 10% of an unknown impurity. See Experimental Section for details.

The performances of heteroaromatic substrates **1j**–**1r** exhibited greater diversity. Full conversions were achieved for all heteroaromatic substrates except for **1m** and **1n**, for which conversions reached 98% and 95%, respectively. Heteroaromatic substrates **1j**–**1r** in general provided lower stereoselectivities compared to aromatic ones **1a**–**1i** (Scheme 2A). While **1j**–**1l**, **1p**, and **1q** afforded good *E*:*Z* stereoselectivities ranging from 9.1:1 (for **1j** and **1l**) to 5.6:1 (for **1p**), substrates **1m**–**1o** and **1r** provided lower *E*:*Z* stereoselectivities ranging from 4.0:1 (for **1m**–**1o**) to 3.4:1 (for **1r**). Interestingly, substrates **1j**–**1l** with an acyl moiety at the *meta* and *para* position of the pyridine ring exhibited higher stereoselectivities (9.1:1 to 7.7:1 *E*:*Z*) compared to pyridine ring-containing substrates **1m**–**1o** with an *ortho* substitution for the acyl moiety (4.0:1 *E*:*Z*). Similar trends were also observed for thiophene derivatives **1p** and **1q** (5.6:1 and 7.1:1 *E*:*Z*, respectively). Furan derivative **1r** provided the lowest stereoselectivity among all of the aromatic substrates. The isolated yields of heteroaromatic products *E*-**2j**–*E*-**2r** ranged from a moderate value of 27% (for **2r**) to a good value of 68% (for **2j**). Given the full or high conversion of heteroaromatic substrates **1j**–**1r** to products **2j**–**2r**, respectively, the lower yields observed for some products (e.g., **2k**, **2n**, **2o**, and **2r**) are attributed to a suboptimal general isolation procedure. This procedure, developed and optimized for product **2a**, does not account for the varying physicochemical properties of the other products **2**. The yield could be enhanced through further optimization of the crystallization procedure for each individual product **2** and the utilization of a second crop crystallization. Thus, an alternative workup procedure based on the recrystallization from 2-PrOH was developed and provided pure **2n** in 53% yield as well as a notably improved yield for **2r** (53%). This isolation procedure was used also for isolations of alkyl-substituted products (vide infra). Another trend observed among aromatic and heteroaromatic derivatives was a decrease in stereoselectivity with five-membered heteroaromatics, as seen when comparing phenyl-KAT **1b**, thiophenyl-KAT **1q**, and furanyl-KAT **1r** (100:0, 7.1:1, and 3.4:1 *E*:*Z*, respectively).^20^

Next, a series of aliphatic substrates **1s**–**1x** were also explored in terms of olefination with DECMP (Scheme 2B). Interesting trends were observed in this series of substrates, particularly when comparing cyclic and acyclic substrates. Substrate **1s** with an ethyl side chain provided full conversion and moderate stereoselectivity (2.3:1 *E*:*Z*) and provided an only 23% yield of *E*-**2s** after crystallization from 2-PrOH. The olefination of substrate **1t**, which contains an isobutyl side chain, resulted in a significantly lower conversion of 79% and reduced stereoselectivity (1.7:1 *E*:*Z*). This indicates that increased steric hindrance on the side chain significantly impacts the reactivity of these substrates and, to a lesser extent, their stereoselectivity. *E*-**2t** was isolated in 36% yield. Furthermore, this trend was confirmed with a *tert*-butyl side chain-containing substrate **2u**, where notably increased steric hindrance resulted in no conversion. Similar results were previously observed for the HWE olefination of ketones with a *t*-Bu substituent.^18b^ Cyclic alkyl substrates **1v**–**1x** were examined next. Cyclohexyl substrate **1v** provided full conversion and good stereoselectivity (6.3:1 *E*:*Z*) along with a 57% yield of *E*-**2v**. Next, cyclopentyl substrate **1w** afforded 94% conversion and notably lower stereoselectivity (3.0:1 *E*:*Z*) and provided a 60% yield of *E*-**2w**. Cyclopropyl substrate **1x** also afforded full conversion but the lowest overall stereoselectivity with (1.8:1 *E*:*Z*) and a 47% yield of *E*-**2x**. These results suggest that higher stereoselectivities are associated with higher conformational flexibility of the cycloalkyl substituent of substrates **1v**–**1x**.

The stereochemistry of prepared α,β-unsaturated β-boryl nitriles **2** was established using two-dimensional (2D) NOESY NMR spectroscopy. The 2D ^1^H–^1^H NOESY spectra obtained for **2a**, **2g**, **2i**, **2s**, and **2v** demonstrated that *E*-**2** was the major isomer obtained in the reaction and isolated from the mixture (see the Supporting Information Figures S3–S7).

Next, we aimed to showcase the utility of *E*-α,β-unsaturated β-boryl nitriles **2** in subsequent functionalization. First, we studied the oxidation of **2a** to β-ketonitrile **3a**.^21^ Direct oxidations of organotrifluoroborates are known to be challenging.^22^ Interestingly, NaBO_3_ in THF/H_2_O^10^ or in situ-generated hydrogen peroxide in the presence of visible light^23^ provided no reaction. However, treatment of **2a** with Oxone in acetone^24^ at 0 °C for 2 h provided β-ketonitrile **3a**, as a useful precursor of heterocyclic compounds,^21,25,26^ in 100% yield (Scheme 3A). Subsequently, **2b** did not show reactivity with PhBr under Suzuki conditions.^27^ Therefore, **2b** was first converted into **4b** in 89% yield using 2,3-dimethylbutane-2,3-diol in the presence of TMSCl/K_2_CO_3_.^28^ Suzuki coupling of **4b** with PhBr in the presence of a Pd catalyst at 50 °C after 3 h afforded **5b** ^10^ in 67% yield (Scheme 3B). In the final part of our investigation, we aimed to provide preliminary evidence of the broader scope of the olefination of KATs under the HWE reaction conditions. Thus, **1a** was reacted with triethyl phosphonoacetate in 1,4-dioxane in the presence of KHDMS at room temperature. Nevertheless, the olefination was slower compared to the one that provided nitriles **2** and gave a ca. 80% conversion in 48 h, which allowed the isolation of *E*-α,β-unsaturated β-ester **6a** in 49% yield (Scheme 3C).

**Scheme 3:**
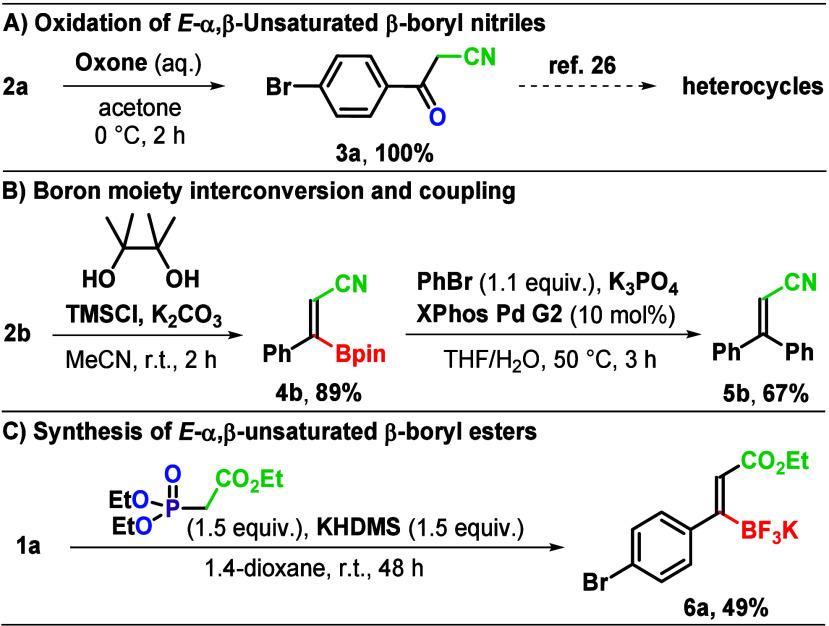
Use of *E*-α,β-Unsaturated β-Boryl Nitriles and KAT Olefination Scope Extension

In summary, we have developed a new transition-metal-free access to *E*-α,β-unsaturated β-boryl nitriles. This approach highlights the first stereoselective Horner–Wadsworth–Emmons reactions on acylboranes. Unlike previous methods, our approach avoids the use of highly toxic reagents and does not require high temperatures or cryogenic conditions. A key feature of the developed methodology is its operational simplicity, which provides title compounds using mild reaction conditions and a column chromatography-free isolation protocol. Most of the pure *E* products are obtained in 50–80% yields.

## Experimental Section

### General Information

All reactions were performed under a nitrogen atmosphere. All of the reagents and solvents purchased commercially were used without further purification. Visualization of aromatic compounds was performed by illumination with a UV lamp (254 nm). Melting points were determined using the DSC 3+ STAR^e^ System differential scanning calorimeter (Mettler Toledo). The sample was heated in a 40 μL aluminum pan with a pierced aluminum lid from 30 to 350 °C at a rate of 10 K/min. Nitrogen (purge rate of 200 mL/min) was used as the purge gas. FTIR spectra were collected with a Nicolet iS50 FTIR spectrometer (Thermo Fisher Scientific Inc.) using a KBr disk or ATR sampling technique. ^1^H, ^13^C, ^11^B, and ^19^F NMR spectra were recorded with a Bruker Avance III 500 MHz NMR spectrometer operating at 500, 126, 160, and 470 MHz, respectively. Samples were prepared by dissolving in DMSO-*d*_6_ (D, 99.8%, Merck), acetone-*d*_6_ (D, 99.8%, VWR Chemicals), or CDCl_3_ (D, 99.8%, Merck), and spectra were recorded at 298.15 K in quartz NMR tubes. Chemical shifts (δ) were expressed in parts per million with reference to the residual solvent signal (2.50 and 39.5 ppm for ^1^H and ^13^C NMR, respectively, for DMSO-*d*_6_, 2.05 ppm for ^1^H NMR for acetone-*d*_6_, and 7.26 and 77.2 ppm for ^1^H and ^13^C NMR, respectively, for CDCl_3_). Coupling constants (*J*) are given in hertz. Multiplicities are indicated as follows: s, singlet; d, doublet; t, triplet; q, quartet; quint, quintet; m, multiplet; br, broadened. Assignments of *E* and *Z* configurations for products **2a**, **2g**, **2i**, **2s**, and **2v** were made via a 2D NMR technique (^1^H–^1^H NOESY NMR). The 2D NOESY NMR spectra were acquired with 2048 data points in F2 and 512 data points in F1, with 16 transients per increment and a 2.0 s relaxation delay. The mixing time was set at 500 ms. HRMS spectra were recorded with an Agilent 6224 time-of-flight mass spectrometer equipped with a double orthogonal electrospray source at atmospheric pressure ionization (ESI) coupled to an HPLC instrument.

### Standard Procedure for the Preparation and Isolation of Substrates **2a–2x**

In a round-bottom flask closed with a septum, dry dioxane (30 mL) and diethyl cyanomethylphosphonate (DECMP, 1.5 equiv) were charged and purged with nitrogen at room temperature. Afterward, KHMDS (1 M in THF, 1.5 equiv) was added, and the reaction mixture was allowed to stir for 1 h at room temperature. Then, substrate **1** (300 mg, 1.03–1.83 mmol, 1.0 equiv) was added as a solid, and the reaction mixture was purged with nitrogen. The reaction mixture was allowed to stir for 18 h at room temperature.

#### Isolation with Precipitation from a Mixture of MeCN and a 10% Aqueous KHCO_3_ Solution (products **2a–2r**)

After 18 h, the solvent was evaporated to the final volume of ∼1 mL. To the wet residual 1.5 mL of acetonitrile was added, and 30 mL of a 10% aqueous KHCO_3_ solution was added dropwise. Then, the mixture was allowed to cool to −5 °C and stirred for 20 h. The precipitated product was filtered and washed with cold water and MTBE.

#### Isolation with Crystallization from 2-PrOH (products **2s–2x**)

After 18 h, the solvent was evaporated. To the dry residual was added isopropanol (15 mL), and the mixture was heated to reflux and stirred at reflux for 1 h using a 100 mL glass reactor inserted into a solid-state thermostat. The mixture was slowly cooled to −10 °C and stirred for 20 h. The precipitated product was filtered and washed with cold isopropanol.

### Characterization Data

Compound **2a** was prepared from 300 mg (1.03 mmol) of **1a** as a pale, off-white solid in 80% isolated yield (265 mg). DSC (10 K/min): 287.48 °C (onset), 291.11 °C (peak). IR (KBr): 3439, 3054, 2918, 2225, 1583, 1485, 1333, 1082, 1011, 979, 880, 812 cm^–1^. ^1^H NMR (500 MHz, DMSO-*d*_6_): δ 7.47–7.36 (m, 4H), 5.62 (br s, 1H). ^13^C­{^1^H} NMR (126 MHz, DMSO-*d*_6_): δ 175.2, 142.8, 130.6, 129.2, 120.7, 119.8, 99.6. ^11^B NMR (160 MHz, DMSO-*d*_6_): δ 1.21 (q, *J* = 52.7 Hz). ^19^F NMR (470 MHz, DMSO-*d*_6_): δ −137.82 (m). HRMS (ESI^–^): calcd for C_9_H_5_BBrF_3_N [M – H]^−^, 272.9692; found, 272.9701.

Compound **2b** was prepared from 300 mg (1.42 mmol) of **1b** as a pale, off-white solid in 68% isolated yield (226 mg). DSC (10 K/min): 257.16 °C (onset), 261.89 °C (peak). IR (KBr): 3441, 3022, 2227, 1584, 1336, 1098, 1084, 1012, 979, 882, 759, 702 cm^–1^. ^1^H NMR (500 MHz, DMSO-*d*_6_): δ 7.42–7.25 (m, 5H), 5.56 (br s, 1H). ^13^C­{^1^H} NMR (126 MHz, DMSO-*d*_6_): δ 176.2, 143.7, 127.7, 127.2, 127.0, 120.0, 99.0. ^11^B NMR (160 MHz, DMSO-*d*_6_): δ 1.37 (q, *J* = 51.2 Hz). ^19^F NMR (471 MHz, DMSO-*d*_6_): δ −137.46 (m). HRMS (ESI^–^): calcd for C_9_H_6_BF_3_N [M – H]^−^, 195.0587; found, 195.0590. This compound is known and has been previously characterized in the literature. The NMR spectra of this compound are consistent with those previously reported.^10^

Compound **2c** was prepared from 300 mg (1.33 mmol) of **1c** as a light-brown solid in 69% isolated yield (227 mg). DSC (10 K/min): 274.96 °C (onset), 278.02 °C (peak). IR (KBr): 3438, 3024, 2916, 2224, 1582, 1509, 1330, 1080, 1009, 984, 885, 805 cm^–1^. ^1^H NMR (500 MHz, DMSO-*d*_6_): δ 7.20 (dd, *J* = 131.0, 7.8 Hz, 4H), 5.54 (br s, 1H), 2.27 (s, 3H). ^13^C­{^1^H} NMR (126 MHz, DMSO-*d*_6_): δ 176.2, 140.7, 136.6, 128.3, 127.0, 120.1 98.01, 20.7. ^11^B NMR (160 MHz, DMSO-*d*_6_): δ 1.40 (q, *J* = 52.8 Hz). ^19^F NMR (470 MHz, DMSO-*d*_6_): δ −137.54 (m). HRMS (ESI^–^): calcd for C_10_H_8_BF_3_N [M – H]^−^, 209.0744; found, 209.0745.

Compound **2d** was prepared from 300 mg (1.30 mmol) of **1d** as a pale, off-white solid in 72% isolated yield (237 mg). DSC (10 K/min): 268.66 °C (onset), 273.98 °C (peak). IR (KBr): 3452, 3070, 2223, 1898, 1597, 1574, 1506, 1219, 1160, 1097, 1081, 977, 886, 822 cm^–1^. ^1^H NMR (500 MHz, DMSO-*d*_6_): δ 7.47 (t, *J* = 7.5 Hz, 2H), 7.09 (t, *J* = 10 Hz, 2H), 5.59 (br s, 1H). ^13^C­{^1^H} NMR (126 MHz, DMSO-*d*_6_): δ 173.9, 162.8, 160.9, 139.8, 129.0, 129.0, 119.9, 114.5, 114.3, 99.0. ^11^B NMR (160 MHz, DMSO-*d*_6_): δ 1.34 (q, *J* = 51.8 Hz). ^19^F NMR (470 MHz, DMSO-*d*_6_): δ −116.52 (s), −137.80 (m). HRMS (ESI^–^): calcd for C_9_H_5_BF_4_N [M – H]^−^, 213.0493; found, 213.0497. This compound is known and has been previously characterized in the literature. The NMR spectra of this compound are consistent with those previously reported.^10^

Compound **2e** was prepared from 300 mg (1.24 mmol) of **1e** as a white solid in 68% isolated yield (223 mg). DSC (10 K/min): 253.53 °C (onset), 257.09 °C (peak). IR (KBr): 3442, 3004, 2935, 2837, 2220, 1604, 1563, 1511, 1282, 1241, 1116, 1039, 978, 886, 820 cm^–1^. ^1^H NMR (500 MHz, DMSO-*d*_6_): δ 7.43 (d, *J* = 8.2 Hz, 2H), 6.83 (d, *J* = 8.1 Hz, 2H), 5.54 (br s, 1H), 3.74 (s, 3H). ^13^C­{^1^H} NMR (126 MHz, DMSO-*d*_6_): δ 174.6, 159.0, 135.6, 128.5, 120.4, 113.1, 96.9, 55.0. ^11^B NMR (160 MHz, DMSO-*d*_6_): δ 1.45 (q, *J* = 51.2 Hz). ^19^F NMR (471 MHz, DMSO-*d*_6_): δ −137.28 (m). HRMS (ESI^–^): calcd for C_10_H_8_BF_3_NO [M – H]^−^, 225.0693; found, 225.0695. This compound is known and has been previously characterized in the literature. The NMR spectra of this compound are consistent with those previously reported.^10^

Compound **2f** was prepared from 300 mg (1.07 mmol) of **1f** as a pale, off-white solid in 63% isolated yield (206 mg). DSC (10 K/min): 273.97 °C (onset), 276.03 °C (peak). IR (KBr): 3449, 2222, 1618, 1586, 1569, 1410, 1321, 1198, 1175, 1126, 1070, 978, 885, 824 cm^–1^. ^1^H NMR (500 MHz, DMSO-*d*_6_): δ 7.63 (d, *J* = 8.4 Hz, 2H), 7.58 (d, *J* = 8.3 Hz, 2H), 5.66 (br s, 1H). ^13^C­{^1^H} NMR (126 MHz, DMSO-*d*_6_): δ 174.3, 148.1, 127.6, 124.6, 124.5, 124.5, 119.4, 101.1. ^11^B NMR (160 MHz, DMSO-*d*_6_): δ 1.23 (q, *J* = 53.3 Hz). ^19^F NMR (471 MHz, DMSO-*d*_6_): δ −62.14, −138.21 (m). HRMS (ESI^–^): calcd for C_10_H_5_BF_6_N [M – H]^−^, 263.0461; found, 263.0465.

Compound **2g** was prepared from 300 mg (1.27 mmol) of **1g** as a pale, off-white solid in 76% isolated yield (250 mg). DSC (10 K/min): 220.06 °C (onset), 232.62 °C (peak). IR (KBr): 3406, 3046, 2226, 2174, 1603, 1500, 1331, 1104, 1065, 974, 884, 833 cm^–1^. ^1^H NMR (500 MHz, DMSO-*d*_6_): δ 7.72 (d, *J* = 8.0 Hz, 2H), 7.56 (d, *J* = 8.0 Hz, 2H), 5.68 (br s, 1H). ^13^C­{^1^H} NMR (126 MHz, DMSO-*d*_6_): δ 174.9, 148.7, 131.6, 127.8, 119.3, 119.1, 109.6, 101.6. ^11^B NMR (160 MHz, DMSO-*d*_6_): δ 1.19 (q, *J* = 50.2 Hz). ^19^F NMR (471 MHz, DMSO-*d*_6_): δ −138.05 (m). HRMS (ESI^–^): calcd for C_10_H_5_BF_3_N_2_ [M – H]^−^, 220.0540; found, 220.0545. This compound is known and has been previously characterized in the literature. The NMR spectra of this compound are consistent with those previously reported.^10^

Compound **2h** was prepared from 300 mg (1.06 mmol) of **1h** as a light-brown solid in 75% isolated yield (251 mg). DSC (10 K/min): 260.55 °C (onset), 265.11 °C (peak). IR (KBr): 3401, 2990, 2911, 2221, 1713, 1608, 1290, 1125, 1082, 986, 886, 769, 705 cm^–1^. ^1^H NMR (500 MHz, DMSO-*d*_6_): δ 7.86 (d, *J* = 8.0 Hz, 2H), 7.52 (d, *J* = 8.1 Hz, 2H), 5.65 (br s, 1H), 4.30 (q, *J* = 7.1 Hz, 2H), 1.31 (t, *J* = 7.1 Hz, 3H). ^13^C­{^1^H} NMR (126 MHz, DMSO-*d*_6_): δ 175.5, 165.7, 148.7, 128.6, 128.4, 127.2, 119.5, 100.8, 60.6, 14.2. ^11^B NMR (160 MHz, DMSO-*d*_6_): δ 1.21 (q, *J* = 65.1 Hz). ^19^F NMR (471 MHz, DMSO-*d*_6_): δ −137.94 (m). HRMS (ESI^–^): calcd for C_12_H_10_BF_3_NO_2_ [M – H]^−^, 267.0798; found, 267.0804.

Compound **2i** was prepared from 300 mg (1.06 mmol) of **1i** as a light-brown solid in 73% isolated yield (236 mg). DSC (10 K/min): 282.01 °C (onset), 286.28 °C (peak). IR (KBr): 3418, 2989, 2224, 1712, 1576, 1295, 1225, 1107, 1084, 1012, 978, 756 cm^–1^. ^1^H NMR (500 MHz, DMSO-*d*_6_): δ 8.02 (s, 1H), 7.83 (d, *J* = 7.6 Hz, 1H), 7.63 (d, *J* = 7.7 Hz, 1H), 7.42 (t, *J* = 7.8 Hz, 1H), 5.62 (br s, 1H), 4.31 (q, *J* = 7.2 Hz, 2H), 1.32 (d, *J* = 7.1 Hz, 3H). ^13^C­{^1^H} NMR (126 MHz, DMSO-*d*_6_): δ 174.9, 165.9, 144.3, 131.4, 129.5, 128.2, 127.9, 119.6, 100.1, 60.6, 14.2. ^11^B NMR (160 MHz, DMSO-*d*_6_): δ 1.22 (q, *J* = 67.3 Hz). ^19^F NMR (471 MHz, DMSO-*d*_6_): δ −138.00. HRMS (ESI^–^): calcd for C_12_H_10_BF_3_NO_2_ [M – H]^−^, 267.0798; found, 267.0811.

Compound **2j** was prepared from 300 mg (1.14 mmol) of **1j** as a light-brown solid in 68% isolated yield (220 mg). DSC (10 K/min): 264.15 °C (onset), 266.82 °C (peak). IR (KBr): 3439, 3045, 3018, 2221, 1570, 1491, 1310, 1136, 1075, 1016, 830, 753 cm^–1^. ^1^H NMR (500 MHz, DMSO-*d*_6_): δ 8.96 (d, *J* = 2.3 Hz, 1H), 8.32 (d, *J* = 2.4 Hz, 1H), 7.97 (dd, *J* = 16.9, 8.3 Hz, 2H), 7.71 (t, *J* = 7.6 Hz, 1H), 7.58 (t, *J* = 7.5 Hz, 1H), 5.89 (br s, 1H). ^13^C­{^1^H} NMR (126 MHz, DMSO-*d*_6_): δ 172.7, 149.9, 146.8, 136.4, 133.0, 129.2, 128.5, 128.4, 127.4, 126.6, 119.6, 101.0. ^11^B NMR (160 MHz, DMSO-*d*_6_): δ 1.40 (q, *J* = 52.8 Hz). ^19^F NMR (471 MHz, DMSO-*d*_6_): δ −137.87 (m). HRMS (ESI^–^): calcd for C_12_H_7_BF_3_N_2_ [M – H]^−^, 246.0696; found, 246.0703.

Compound **2k** was prepared from 300 mg (1.03 mmol) of **1k** as a light-yellow solid in 47% isolated yield (151 mg). DSC (10 K/min): 264.69 °C (onset), 271.35 °C (peak). IR (KBr): 3439, 3032, 2216, 1573, 1459, 1369, 1325, 1090, 162, 1021, 880, 818 cm^–1^. ^1^H NMR (500 MHz, DMSO-*d*_6_): δ 8.36 (m, 1H), 7.74 (m, 1H), 7.56 (d, *J* = 8.3 Hz, 1H), 5.76 (br s, 1H). ^13^C­{^1^H} NMR (126 MHz, DMSO-*d*_6_): δ 171.3, 148.2, 140.1, 138.6, 137.8, 127.2, 119.2, 101.1. ^11^B NMR (160 MHz, DMSO-*d*_6_): δ 1.13 (q, *J* = 50.3 Hz). ^19^F NMR (471 MHz, DMSO-*d*_6_): δ −138.46 (m). HRMS (ESI^–^): calcd for C_8_H_4_BBrF_3_N_2_ [M – H]^−^, 273.9645; found, 273.9658.

Compound **2l** was prepared from 300 mg (1.21 mmol) of **1l** as a pale, off-white solid in 66% isolated yield (226 mg). DSC (10 K/min): 195.4 °C (onset), 202.2 °C (peak). IR (KBr): 3444, 3056, 2217, 1586, 1527, 1463, 1378, 1129, 1065, 992, 928, 833, 748 cm^–1^. ^1^H NMR (500 MHz, DMSO-*d*_6_): δ 8.30 (d, *J* = 5.2 Hz, 1H), 7.40 (s, 1H), 7.35 (d, *J* = 5.2 Hz, 1H), 5.82 (br s, 1H). ^13^C­{^1^H} NMR (126 MHz, DMSO-*d*_6_): δ 171.5, 154.9, 150.1, 149.3, 121.8, 121.1, 118.9, 103.1. ^11^B NMR (160 MHz, DMSO-*d*_6_): δ 1.02 (q, *J* = 48.9 Hz). ^19^F NMR (471 MHz, DMSO-*d*_6_): δ −138.62 (m). HRMS (ESI^–^): calcd for C_8_H_4_BClF_3_N_2_ [M – H]^−^, 230.0150; found, 230.0159.

Compound **2m** was prepared from 300 mg (1.21 mmol) of **1m** as a light-brown solid in 62% isolated yield (203 mg). DSC (10 K/min): 283.64 °C (onset), 293.16.2 °C (peak). IR (KBr): 3447, 2211, 1566, 1542, 1324, 1072, 1004, 980, 918, 836, 744 cm^–1^. ^1^H NMR (500 MHz, DMSO-*d*_6_): δ 8.49 (d, *J* = 5.2 Hz, 1H), 7.75 (d, *J* = 2.0 Hz, 1H), 7.43 (dd, *J* = 5.2, 2.1 Hz, 1H), 6.49 (br s, 1H). ^13^C­{^1^H} NMR (126 MHz, DMSO-*d*_6_): δ 169.4, 159.5, 150.1, 142.7, 124.3, 122.9, 119.8, 102.7. ^11^B NMR (160 MHz, DMSO-*d*_6_): δ 1.37 (q, *J* = 50.1 Hz). ^19^F NMR (471 MHz, DMSO-*d*_6_): δ −137.65 (m). HRMS (ESI^–^): calcd for C_8_H_4_BClF_3_N_2_ [M – H]^−^, 230.0150; found, 230.0154.

Compound **2n** was prepared from 300 mg (1.03 mmol) of **1n** as a light-brown solid in 52% isolated yield (185 mg, which contained 10% of an unknown impurity) with a precipitation procedure from MeCN using a 10% aqueous KHCO_3_ solution and 53% isolated yield (173 mg) with crystallization from 2-PrOH. DSC (10 K/min): 176.25 °C (onset), 179.76 °C (peak). IR (KBr): 3440, 2219, 1659, 1565, 1465, 1316, 1257, 1001, 889, 820 cm^–1^. ^1^H NMR (500 MHz, DMSO-*d*_6_): δ 8.61 (d, *J* = 2.4 Hz, 3H), 7.98 (dd, *J* = 8.5, 2.5 Hz, 4H), 7.69 (d, *J* = 8.5 Hz, 4H), 6.42 (s, 2H). ^13^C­{^1^H} NMR (126 MHz, DMSO-*d*_6_): δ 170.0, 156.6, 149.3, 138.7, 126.2, 119.9, 119.6, 101.7. ^11^B NMR (160 MHz, DMSO-*d*_6_): δ 1.35 (q, *J* = 50.2 Hz). ^19^F NMR (471 MHz, DMSO-*d*_6_): δ −137.72 (m). HRMS (ESI^–^): calcd for C_8_H_4_BBrF_3_N_2_ [M – H]^−^, 273.9645; found, 273.9649.

Compound **2o** was prepared from 300 mg (1.21 mmol) of **1o** as a pale, light-brown solid in 34% isolated yield (111 mg). DSC (10 K/min): 230.11 °C (onset), 232.88 °C (peak). IR (KBr): 3444, 2221, 1575, 1550, 1438, 1309, 1078, 1026, 997, 796 cm^–1^. ^1^H NMR (500 MHz, DMSO-*d*_6_): δ 7.76 (dd, *J* = 37.5, 7.7 Hz, 2H), 7.38 (d, *J* = 7.8 Hz, 1H), 6.35 (br s, 1H). ^13^C­{^1^H} NMR (126 MHz, DMSO-*d*_6_): δ 169.4, 158.7, 149.3, 139.9, 123.7, 123.4, 119.7, 102.4. ^11^B NMR (160 MHz, DMSO-*d*_6_): δ 1.32 (q, *J* = 49.4 Hz). ^19^F NMR (471 MHz, DMSO-*d*_6_): δ −137.64. HRMS (ESI^–^): calcd for C_8_H_4_BClF_3_N_2_ [M – H]^−^, 230.0150; found, 230.0157.

Compound **2p** was prepared from 300 mg (1.38 mmol) of **1p** as a light-brown solid in 61% isolated yield (207 mg). DSC (10 K/min): 269.36 °C (onset), 274.70 °C (peak). IR (KBr): 3443, 3076, 2219, 1564, 1331, 1209, 1068, 988, 810, 798 cm^–1^. ^1^H NMR (500 MHz, DMSO-*d*_6_): δ 7.42 (m, 2H), 7.01 (s, 1H), 5.69 (br s, 1H). ^13^C­{^1^H} NMR (126 MHz, DMSO-*d*_6_): δ 166.2, 146.0, 129.0, 127.8, 126.6, 120.0, 95.6. ^11^B NMR (160 MHz, DMSO-*d*_6_): δ 1.16 (q, *J* = 49.2 Hz). ^19^F NMR (471 MHz, DMSO-*d*_6_): δ −138.22 (m). HRMS (ESI^–^): calcd for C_7_H_4_BF_3_NS [M – H]^−^, 201.0151; found, 201.0157.

Compound **2q** was prepared from 300 mg (1.38 mmol) of **1q** as a light-brown solid in 67% isolated yield (221 mg). DSC (10 K/min): 268.56 °C (onset), 276.57 °C (peak). IR (KBr): 3451, 3128, 2222, 1578, 1318, 1180, 1078, 982, 853, 783 cm^–1^. ^1^H NMR (500 MHz, DMSO-*d*_6_): δ 7.64 (s, 1H), 7.37 (d, *J* = 30.5 Hz, 2H), 5.80 (br s, 1H). ^13^C­{^1^H} NMR (126 MHz, DMSO-*d*_6_): δ 168.6, 143.9, 126.6, 125.0, 124.9, 120.4, 96.9. ^11^B NMR (160 MHz, DMSO-*d*_6_): δ 1.39 (q, *J* = 52.1 Hz). ^19^F NMR (471 MHz, DMSO-*d*_6_): δ −138.10 (m). HRMS (ESI^–^): calcd for C_7_H_4_BF_3_NS [M – H]^−^, 201.0151; found, 201.0154.

Compound **2r** was prepared from 300 mg (1.48 mmol) of **1r** as a light-brown solid in 27% isolated yield (92 mg) with a precipitation procedure from MeCN using a 10% aqueous KHCO_3_ solution and 53% isolated yield (182 mg) with crystallization from 2-PrOH. DSC (10 K/min): 232.29 °C (onset), 239.62 °C (peak). IR (KBr): 3437, 3181, 2207, 1581, 1512, 1390, 1320, 1163, 1102, 1017, 972, 813, 800 cm^–1^. ^1^H NMR (500 MHz, DMSO-*d*_6_): δ 7.76 (s, 1H), 7.55 (s, 1H), 6.72 (s, 1H), 5.69 (br s, 1H). ^13^C­{^1^H} NMR (126 MHz, DMSO-*d*_6_): δ 165.2, 143.8, 143.0, 127.3, 120.5, 107.9, 95.5. ^11^B NMR (160 MHz, DMSO-*d*_6_): δ 1.32 (q, *J* = 51.4 Hz). ^19^F NMR (471 MHz, DMSO-*d*_6_): δ −138.93 (m). HRMS (ESI^–^): calcd for C_7_H_4_BF_3_NO [M – H]^−^, 185.0380; found, 185.0386.

Compound **2s** was prepared from 300 mg (1.83 mmol) of **1s** as a pale, off-white solid in 23% isolated yield (80 mg). DSC (10 K/min): 230.11 °C (onset), 243.80 °C (peak). IR (KBr): 3440, 2960, 2934, 2220, 1603, 1344, 1166, 1001, 971, 920, 827 cm^–1^. ^1^H NMR (500 MHz, DMSO-*d*_6_): δ 5.14 (br s, 1H), 2.10 (q, *J* = 7.4 Hz, 2H), 0.90 (t, *J* = 7.5 Hz, 3H). ^13^C­{^1^H} NMR (126 MHz, DMSO-*d*_6_): δ 184.1, 120.1, 95.2, 29.7, 12.5. ^11^B NMR (160 MHz, DMSO-*d*_6_): δ 1.14 (q, *J* = 52.2 Hz). ^19^F NMR (471 MHz, DMSO-*d*_6_): δ −141.72 (m). HRMS (ESI^–^): calcd for C_5_H_6_BF_3_N [M – H]^−^, 147.0587; found, 147.0589.

Compound **2t** was prepared from 300 mg (1.56 mmol) of **1t** as a light-yellow solid in 36% isolated yield (122 mg). DSC (10 K/min): 255.97 °C (onset), 261.29 °C (peak). IR (KBr): 3439, 2957, 2867, 2218, 2110, 2089, 1598, 1172, 1005, 976 cm^–1^. ^1^H NMR (500 MHz, DMSO-*d*_6_): δ 5.12 (br s, 1H), 1.92 (d, *J* = 6.9 Hz, 2H), 1.87 (dt, *J* = 13.5, 6.6 Hz, 1H), 0.76 (d, *J* = 6.4 Hz, 6H). ^13^C­{^1^H} NMR (126 MHz, DMSO-*d*_6_): δ 180.7, 119.9, 97.4, 47.8, 25.9, 22.8. ^11^B NMR (160 MHz, DMSO-*d*_6_): δ 1.06 (q, *J* = 55.7 Hz). ^19^F NMR (471 MHz, DMSO-*d*_6_): δ −141.16 (m). HRMS (ESI^–^): calcd for C_7_H_10_BF_3_N [M – H]^−^, 175.0900; found, 175.0903.

Compound **2v** was prepared from 300 mg (1.38 mmol) of **1v** as a light-yellow solid in 57% isolated yield (194 mg). DSC (10 K/min): 209.19 °C (onset), 216.35 °C (peak). IR (KBr): 3387, 2931, 2850, 2220, 1653, 1586, 1119, 1022, 976, 812 cm^–1^. ^1^H NMR (500 MHz, DMSO-*d*_6_): δ 5.13 (br s, 1H), 2.12 (td, *J* = 10.1, 5.8 Hz, 1H), 1.65 (m, 5H), 1.10 (m, 6H). ^13^C­{^1^H} NMR (126 MHz, DMSO-*d*_6_): δ 186.8, 120.6, 94.8, 44.2, 31.7, 26.5, 26.0. ^11^B NMR (160 MHz, DMSO-*d*_6_): δ 1.21 (q, *J* = 56.7 Hz). ^19^F NMR (471 MHz, DMSO-*d*_6_): δ −140.32 (m). HRMS (ESI^–^): calcd for C_9_H_12_BF_3_N [M – H]^−^, 201.1057; found, 201.1062.

Compound **2w** was prepared from 300 mg (1.47 mmol) of **1w** as a pale, off-white solid in 60% isolated yield (200 mg). DSC (10 K/min): 305.20 °C (onset), 309.86 °C (peak). IR (KBr): 3439, 2954, 2867, 2219, 1596, 1318, 1145, 977, 824 cm^–1^. ^1^H NMR (500 MHz, DMSO-*d*_6_): δ 5.17 (br s, 1H), 1.50 (m, 9H). ^13^C­{^1^H} NMR (126 MHz, DMSO-*d*_6_): δ 184.3, 120.3, 94.6, 47.3, 31.3, 24.7. ^11^B NMR (160 MHz, DMSO-*d*_6_): δ 1.17 (q, *J* = 53.2 Hz). ^19^F NMR (471 MHz, DMSO-*d*_6_): δ −139.68 (m). HRMS (ESI^–^): calcd for C_8_H_10_BF_3_N [M – H]^−^, 187.0900; found, 187.0904.

Compound **2x** was prepared from 300 mg (1.70 mmol) of **1x** as a pale-white solid in 47% isolated yield (160 mg). DSC (10 K/min): 252.84 °C (onset), 254.71 °C (peak). IR (KBr): 3435, 3080, 3004, 2218, 1600, 1317, 1156, 1104, 973, 811 cm^–1^. ^1^H NMR (500 MHz, DMSO-*d*_6_): δ 5.03 (br s, 1H), 1.47 (m, 1H), 0.65 (m, 4H). ^13^C­{^1^H} NMR (126 MHz, DMSO-*d*_6_): δ 183.6, 120.4, 93.0, 18.2. ^11^B NMR (160 MHz, DMSO-*d*_6_): δ 0.97 (q, *J* = 51.9 Hz). ^19^F NMR (471 MHz, DMSO-*d*_6_): δ −139.81 (m). HRMS (ESI^–^): calcd for C_6_H_6_BF_3_N [M – H]^−^, 159.0587; found, 159.0591.

**Synthesis of 3-(4-bromophenyl)-3-oxopropanenitrile (3a)**. A 10 mL vial with a septum, inserted into a solid-state thermostat, was charged with **2a** (100 mg, 0.319 mmol) and 3 mL of acetone. The mixture was allowed to cool to 0 °C. A syringe was charged with a solution of Oxone (215 mg dissolved in 1 mL of water, 0.350 mmol, 1.1 equiv), which was slowly added to the vial. The reaction mixture was allowed to stir for 2 h at 0 °C, after aqueous HCl (0.1 M, 956 μL, 0.3 equiv) was added. The reaction mixture was allowed to heat to room temperature and extracted with CH_2_Cl_2_ (3 × 3 mL). Combined organic phases were washed with 10% aqueous NaCl (5 mL) and dried with Na_2_SO_4_. The solvent was evaporated to obtain a solid white product (71 mg, 100% yield). DSC (10 K/min): 161.15 °C (onset), 162.09 °C (peak). IR (ATR): 2950, 2920, 2253, 1684, 1584, 1394, 1328, 1218, 1182, 1070, 1001, 929 cm^–1^. ^1^H NMR (500 MHz, CDCl_3_): δ 7.79 (d, *J* = 8.6 Hz, 2H), 7.68 (d, *J* = 8.6 Hz, 2H), 4.04 (s, 2H). ^13^C­{^1^H} NMR (126 MHz, CDCl_3_): δ 184.1, 133.2, 132.7, 130.5, 130.0, 113.5, 29.5. HRMS (ESI^+^): calcd for C_9_H_6_BrNO [M + H]^+^, 223.9706; found, 223.9706. This compound is known and has been previously characterized in the literature. The NMR spectra of this compound are consistent with those previously reported.^21^

**Synthesis of (*E*)-3-phenyl-3-(4,4,5,5-tetramethyl-1,3,2-dioxaborolan-2-yl)­acrylonitrile (4b)**. A round-bottom flask was charged with **2b** (100 mg, 0.425 mmol), potassium carbonate (147 mg, 2.5 equiv, 1.06 mmol), 2,3-dimethylbutane-2,3-diol (53 mg, 1.05 equiv, 0.447 mmol), and dry acetonitrile (5 mL). The suspension was stirred for 5 min at room temperature, after chlorotrimethylsilane (92 mg, 108 μL, 2 equiv, 0.851 mmol) was added slowly, and the reaction mixture was allowed to stir for 2 h at room temperature. Subsequently, CH_2_Cl_2_ (5 mL) was added, and the mixture was filtered. The solvent was evaporated to obtain yellow oily product, which was dissolved in CH_2_Cl_2_ (1 mL), filtered through a silica plug, and washed with CH_2_Cl_2_. The solvent was evaporated to obtain a solid white product (97 mg, 89% yield). DSC (10 K/min): 221.89 °C (onset), 228.09 °C (peak). IR (ATR): 2979, 2933, 2215, 1383, 1373, 1331, 1216, 1137, 850 cm^–1^. ^1^H NMR (500 MHz, CDCl_3_): δ 7.47–7.40 (m, 2H), 7.40–7.34 (m, 3H), 6.02 (s, 1H), 1.41 (s, 12H). ^13^C­{^1^H} NMR (126 MHz, CDCl_3_): δ 155.69, 138.11, 129.96, 128.97, 127.20, 117.68, 106.61, 85.46, 24.88. ^11^B NMR (160 MHz, CDCl_3_): δ 29.76. HRMS (ESI^+^): calcd for C_15_H_18_BNO_2_ [M + H]^+^, 255.154; found, 255.154. This compound is known and has been previously characterized in the literature. The NMR spectra of this compound are consistent with those previously reported.^10^

**Synthesis of 3,3-diphenylacrylonitrile (5b)**. In a drybox under nitrogen XPhos Pd G2 (29 mg, 0.1 equiv, 0.037 mmol) and **4b** (95 mg, 1 equiv, 0.372 mmol) were charged in a three-neck round-bottom flask, which was closed with a septum and a nitrogen balloon. Through the septum were added THF (3 mL), 0.6 M aqueous potassium phosphate (1.024 mL, 1.65 equiv, 0.614 mmol), and bromobenzene (64 mg, 1.1 equiv, 0.409 mmol). The reaction mixture was allowed to stir at 50 °C for 3 h. Then, the reaction mixture was allowed to cool to room temperature. Water (5 mL) was added, and the mixture was extracted with CH_2_Cl_2_ (3 × 5 mL). Combined organic phases were washed with 10% aqueous NaCl (5 mL). The solvent was evaporated, and the product was further purified by silica gel column chromatography (2% ethyl acetate/heptane solvent system) to give the product as a colorless oil (51 mg, 67% yield). IR (ATR): 3057, 2924, 2853, 2212, 1592, 1568, 1444, 13551078, 827 cm^–1^. ^1^H NMR (500 MHz, CDCl_3_): δ 7.48–7.42 (m, 6H), 7.38 (t, *J* = 7.7 Hz, 2H), 7.30 (dt, *J* = 7.1, 1.4 Hz, 2H), 5.74 (s, 1H). ^13^C­{^1^H} NMR (126 MHz, CDCl_3_): δ 163.3, 139.1, 137.2, 130.5, 130.2, 129.7, 128.8, 128.7, 128.6, 118.0, 95.0. (ESI^+^): calcd for C_15_H_11_N [M + H]^+^, 206.0964; found, 206.0963. This compound is known and has been previously characterized in the literature. The NMR spectra of this compound are consistent with those previously reported.^10^

**Synthesis of compound (6a)**. A round-bottom flask closed with a septum was charged with dry 1,4-dioxane (30 mL) and triethyl phosphonoacetate (307 μL, 1.5 equiv) and purged with nitrogen at room temperature. Then, 1 M KHMDS in THF (1.55 mL, 1.5 equiv) was added, and the reaction mixture was allowed to stir for 1 h at room temperature. Then, substrate **1** (300 mg, 1.03 mmol) was added as a solid, and the reaction mixture was purged with nitrogen. The reaction mixture was allowed to stir for 48 h at room temperature. Then, 200 μL of the reaction mixture was separately evaporated and dissolved in DMSO-*d*_6_ to determine conversion of the reaction with ^1^H NMR spectroscopy (80% conversion determined). Then, the solvent was evaporated from the reaction mixture to a final volume of ∼1 mL. To the wet residual was added 1.5 mL of acetonitrile, and 30 mL of a 10% aqueous KHCO_3_ solution was added dropwise. Then, the mixture was allowed to cool to −5 °C and stirred for 20 h. The precipitated product was filtered and washed with cold water and MTBE to afford an off-white solid (183 mg, 49% yield). DSC (10 K/min): 261.99 °C (onset), 265.38 °C (peak). IR (ATR): 3650, 3529, 2995, 2900, 1720, 1597, 1482, 1252, 1193, 964 cm^–1^. ^1^H NMR (500 MHz, DMSO-*d*_6_): δ 7.39 (d, *J* = 8.0 Hz, 2H), 7.26 (d, *J* = 8.1 Hz, 2H), 5.87 (s, 1H), 4.03 (q, *J* = 7.1 Hz, 2H), 1.20 (t, *J* = 7.1 Hz, 3H). ^13^C­{^1^H} NMR (126 MHz, DMSO-*d*_6_): δ 169.1, 160.1, 145.5, 130.2, 129.1, 123.9, 118.8, 58.9, 14.1. ^11^B NMR (160 MHz, DMSO-*d*_6_): δ 1.49 (s).^29^ ^19^F NMR (471 MHz, DMSO-*d*_6_): δ −136.57 (m). HRMS (ESI^–^): calcd for C_11_H_11_BBrF_3_O_2_ [M – H]^−^, 319.9951; found, 319.9965.

## Supplementary Material



## Data Availability

The data underlying this study are available in the published article and its Supporting Information.

## Notes

Experimental procedures for screening experiments, IR spectra, and NMR spectra (PDF)

## Notes

This manuscript is dedicated to Professor Dr. Miha Japelj (University of Ljubljana, Slovenia) on the occasion of his 90th birthday.
