# Supplementary material for: Application of the Horner–Wadsworth–Emmons Olefination in the Construction of E‑α,β-Unsaturated β‑Boryl Nitriles
Source: J Org Chem. 2025 May 21;90(22):7498–506. doi: 10.1021/acs.joc.4c02915 (PMC12150335; doi:10.1021/acs.joc.4c02915)

Supporting Information

Application of the Horner–Wadsworth–Emmons Olefination in the Construction of E-α,β-Unsaturated β-Boryl Nitriles

Kaja Gosak,[a,b] Martin Črnugelj, [a] and Zdenko Časar*[a,c]

a Lek Pharmaceuticals d.d., Sandoz Development Center Slovenia, Verovškova ulica 57, 1526 Ljubljana, Slovenia

b University of Ljubljana, Faculty of Chemistry and Chemical Technology, Večna pot 113, SI-1000 Ljubljana, Slovenia

c University of Ljubljana, Faculty of Pharmacy, Aškerčeva cesta 7, SI-1000 Ljubljana, Slovenia

Table of Contents

1. General Information S2
2. Preparation of Potassium Acyltrifluoroborates (KATs) S4
3. Preparation of *E*-α,β-unsaturated β-boryl nitriles S5
   3.1. Screening and Optimisation of HWE reaction conditions S5
   3.2. Optimisation of Isolation S9
   3.2.1. Optimisation of Isolation with Precipitation form MeCN and 10% $KHCO_3$ solution S9
   3.2.2. Optimisation of Isolation with Crystallisation from 2-PrOH for Alkyl Substrates S10
   3.3. Determination of stereoselectivity of α,β-unsaturated β-boryl nitriles S12
   3.4. General Procedure for the Synthesis of *E*-α,β-unsaturated β-boryl nitriles S16
4. References S19
5. NMR Spectra S20

1. General Information

All reagents were purchased from commercial vendors (Merck-Sigma Aldrich, TCI, Acros Organics, Fluka, Alfa Aesar) and were used as supplied unless noted otherwise.

For screening and optimisation of HWE reaction following reagents and solvents were used: Tetrahydrofuran (THF), 2-methyl tetrahydrofuran (2-MeTHF), acetonitrile (MeCN), *tert*-butyl methyl ether (MTBE), dimethoxyethane (DME), 1,4-dioxane and cyclopentyl methyl ether (CPME) were supplied as dry solvents from commercial vendors (Merck-Sigma Aldrich, Acros Organics). Methanol (MeOH), ethanol (EtOH), isopropanol (*i*-PrOH), *tert*-butanol (*t*-BuOH), 1-butanol (1-BuOH), 2-butanol (2-BuOH), *n*-pentanol, dimethylacetamide (DMAc), Dimethylformamide (DMF), 3-methyl-1-butanol, acetonitile, *tert*-butyl methyl ether, ethyl acetate were supplied from Merck-Sigma Aldrich or J. T. Baker. Lithium hexamethyldisilazide (LiHMDS), sodium hexamethyldisilazide (NaHMDS) and potassium hexamethyldisilazide (KHMDS) were supplied from Sigma Aldrich as 1 M solution in THF. Lithium diisopropylamide (LDA) was supplied from Sigma Aldrich as 2.0 M in THF/heptane/ethylbenzene. Diethyl cyanomethylphosphonate (DECMP) was supplied from Sigma Aldrich, however, diphenyl cyanomethylphosphonate (DPCMP) was synthesized according to the known literature procedure.[S1] 18-crown-6 as well as inorganic bases potassium carbonate (K_2CO_3), cesium carbonate (Cs_2CO_3), sodium hydrogen carbonate ($NaHCO_3$), potassium phosphate (K_3PO_4), potassium hydrogencarbonate ($KHCO_3$) were supplied from Sigma Aldrich.

For further functionalisation of *E*-α,β-unsaturated β-boryl nitriles **2** following reagents and solvents were used: Acetone, dry acetonitile, tetrahydrofurane, dry 1,4-dioxane and dichloromethane were supplied from commercial vendors (Merck-Sigma Aldrich, Acros Organics). Reagents such as Oxone®, pinacol (2,3-dimethylbutane-2,3-diol), chlorotrimethylsilane (TMSCl), bromobenzene (PhBr), triethyl phosphonoacetate as well as catalyst XPhos Pd G2, were supplied from Merck-Sigma Aldrich.

Visualization of aromatic compounds was performed by illumination with a UV lamp (254 nm). Melting points were determined using the DSC 3+ STAR^e System differential scanning calorimeter (Mettler Toledo). The sample was heated in a 40 µL aluminum pan with a pierced aluminum lid from 30 to 350 °C at a rate of 10 K/min. Nitrogen (purge rate of 200 mL/min) was used as the purge gas. FTIR spectra were collected with a Nicolet™ iS50 FTIR spectrometer (Thermo Fisher Scientific Inc.) using a KBr disk or ATR sampling technique. ^{1}H, ^{13}C, ^{11}B, and ^{19}F NMR spectra were recorded with a Bruker Avance III 500 MHz NMR spectrometer operating at 500, 126, 160, and 470 MHz, respectively. Samples were prepared by dissolving in DMSO-d_6 (D, 99.8 %, Merck), acetone-d_6 (D, 99.8%, VWR Chemicals), or $CDCl_3$ (D, 99.8%, Merck), and spectra were recorded at 298.15 K in quartz NMR tubes. Chemical shifts (δ) were expressed in parts per million with reference to the residual solvent signal (2.50 and 39.5 ppm

for ^{1}H and ^{13}C NMR, respectively, for DMSO-d_6, 2.05 ppm for ^{1}H NMR for acetone-d_6 and 7.26 ppm and 77.2 ppm for ^{1}H and ^{13}C NMR, respectively, for $CDCl_3$). Coupling constants (J) are given in hertz. Multiplicities are indicated as follows: s, singlet; d, doublet; t, triplet; q, quartet; quint, quintet; m, multiplet; br, broadened. Assignments of *E* and *Z* configuration for products **2a**, **2g**, **2i**, **2s** and **2v** were made via a 2D NMR technique (^{1}H–^{1}H NOESY NMR). The 2D NOESY NMR spectra were acquired with 2048 data points in F2 and 512 data points in F1, with 16 transients per increment and a 2.0 s relaxation delay. The mixing time was set at 500 ms. HRMS spectra were recorded with an Agilent 6224 time-of-flight mass spectrometer equipped with a double orthogonal electrospray source at atmospheric pressure ionization (ESI) coupled to an HPLC instrument.

2. Preparation of Potassium Acyltrifluoroborates (KATs)

The following KATs **1a**, **1e**, **1g-r** were purchased from Sigma Aldrich. KATs **1a**–**1d**, **1f** and **1s**–**1x** were synthesized according to the literature procedure[S1,S2]. The following KATs were already synthesized and described in literature (**1a**[S2], **1b**[S2], **1c**[S2], **1d**[S2], **1f**[S2], **1s**[S3], **1t**[S4], **1u**[S2] , **1v**[S2], **1w**[S3], **1x**[S3]). Their spectroscopic data corresponded to those that had been reported previously and are not repeated here.

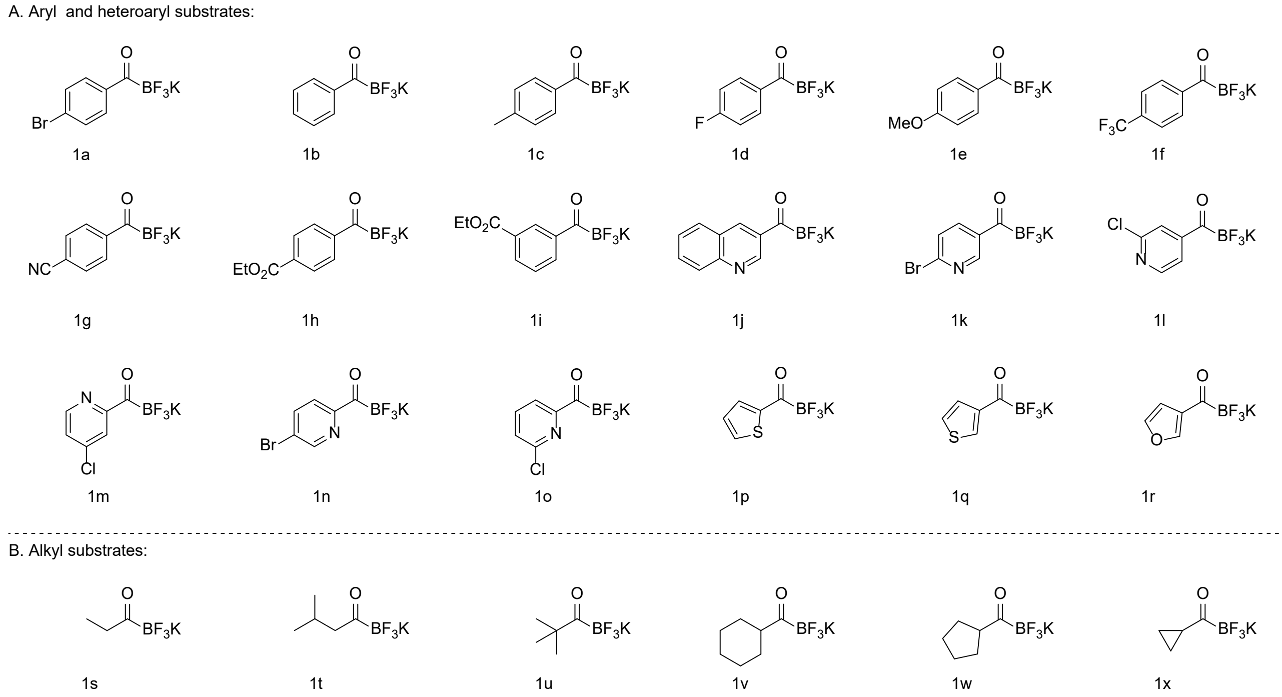

Figure S1. List of KATs used in this study.

3. Preparation of *E*-α,β-unsaturated β-boryl nitriles

3.1. Screening and Optimisation of HWE reaction conditions

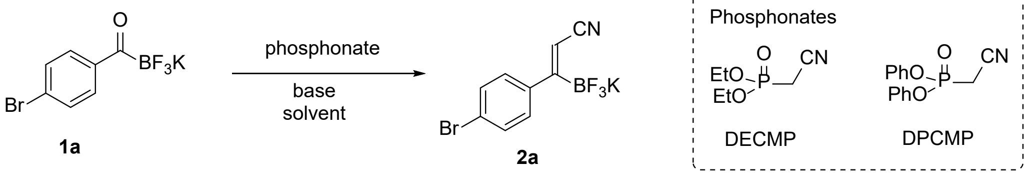

Scheme S1. Screening of condition for HWE reaction on substrate **1a**

General procedures for screening of reaction condition:

Procedure A (entries 1-13 Table S1):
In the vial with 20 mg of **1a** (0.069 mmol), 2 mL of solvent was added. After base and phosphonate was added. The vial was closed and the reaction mixture was heated to the certain temperature and was allowed to stir overnight. After, the solvent was evaporated, ethyl acetate was added and the mixture was extracted with water and brine. Finally, ethyl acetate was evaporated.

Procedure B (entries 14-34 Table S1 and entries 1-17 Table S2):
In the vial 2 mL of dry solvent and phosphonate was added, vial was purged with nitrogen and closed. The mixture was allowed to cool to the certain temperature. Base was added and the mixture was stirred for 1 h. Afterwards, the vial was opened to add 20 mg of **1a** (0.069 mmol), purged with nitrogen, and closed. The mixture was allowed to stir overnight at the certain temperature. After, the solvent was evaporated, ethyl acetate was added and the mixture was extracted with water and brine. Finally, ethyl acetate was evaporated.

Table S1. Conditions screening of HWE reaction

Entry[a]	Phosphonate (equiv.)	Base (equiv.)	Solvent	T [°C]	*Z*-**2a** [%][b]	*E*-**2a** [%][b]	Conversion [%][c]
1	DPCMP (2)	K_2CO_3 (5)	*i*-PrOH	reflux	25.0	75.0	24
2	DECMP (2)	K_2CO_3 (5)	*i*-PrOH	reflux	38.6	61.4	100
3	DECMP (2)	K_2CO_3 (5)	*t*-BuOH	reflux	24.8	75.2	100
4	DECMP (2)	K_2CO_3 (5)	*n*-pentanol	reflux	0	0	No reaction
5	DECMP (2)	K_2CO_3 (5)	DMAc	reflux	0	0	No reaction
6	DECMP (2)	K_2CO_3 (5)	DMF	reflux	0	0	Traces
7	DECMP (2)	K_2CO_3 (5)	2-BuOH	reflux	35.1	64.9	89
8	DECMP (2)	K_2CO_3 (5)	3-methyl-1-butanol	reflux	0	0	Traces
9	DECMP (3)	K_2CO_3 (5)	*i*-PrOH	reflux	40.8	59.2	100
10	DECMP (3)	Cs_2CO_3 (5)	*i*-PrOH	reflux	38.8	61.2	100
11	DECMP (3)	$NaHCO_3$ (5)	*i*-PrOH	reflux	41.3	58.7	100
12	DECMP (3)	K_3PO_4 (5)	*i*-PrOH	reflux	36.5	63.5	100
13	DECMP (3)	$KHCO_3$ (5)	*i*-PrOH	reflux	47.2	52.8	100
14	DECMP (1.3)	LiHMDS (1.2)	THF	-20	57.4	42.6	100
15	DECMP (1.3)	NaHMDS (1.2)	THF	-20	45.0	55.0	95
16	DECMP (1.3)	KHMDS (1.2)	THF	-20	10.5	89.5	100
17	DPCMP (1.3)	LiHMDS (1.2)	THF	-20	64.9	35.1	87
18	DPCMP (1.3)	NaHMDS (1.2)	THF	-20	54.3	45.7	93

Entry[a]	Phosphonate (equiv.)	Base (equiv.)	Solvent	T [°C]	*Z*-**2a** [%][b]	*E*-**2a** [%][b]	Conversion [%][c]
19[d]	DPCMP (1.2)	KHMDS (1.2)	THF	-20	46.1	53.9	12
20[d]	DECMP (1.3)	LiHMDS (1.2)	THF	-20	73.5	26.5	100
21	DECMP (1.3)	KHMDS (1.2)	THF	-20	63.7	36.3	86
22	DECMP (1.3)	LDA (1.2)	THF	-20	48.8	51.2	100
23	DPCMP (1.3)	LDA (1.2)	THF	-20	0	0	Traces
24	DECMP (1.3)	LiHMDS (1.2)	2-MeTHF	-20	36.7	63.3	100
25	DECMP (1.3)	NaHMDS (1.2)	2-MeTHF	-20	33.8	66.2	100
26	DECMP (1.3)	LiHMDS (1.2)	MeCN	-20	68.0	32.0	100
27	DECMP (1.3)	NaHMDS (1.2)	MeCN	-20	63.7	36.3	100
28	DECMP (1.3)	KHMDS (1.2)	MeCN	-20	58.5	41.5	100
29	DPCMP (1.2)	LiHMDS (1.2)	MeCN	-20	78.1	21.9	93
30	DPCMP (1.2)	NaHMDS (1.2)	MeCN	-20	56.2	43.8	83
31	DECMP (1.3)	KHMDS (1.2)	MTBE	-20	10.9	89.1	69[e]
32	DECMP (1.3)	KHMDS (1.2)	DME	-20	38.9	61.1	100
33	DECMP (1.3)	KHMDS (1.2)	1,4-dioxane	15	5.2	94.8	100
34	DECMP (1.3)	KHMDS (1.2)	CPME	-20	9.3	90.7	88[e]

[a] Reactions were conducted by using **1a** (20 mg, 0.069 mmol) at 0.01 g/mL concentration after the isolation the product were analyzed with ^{1}H NMR in acetone-d_6. [b] The percentage of formed *Z*- and *E*-isomer was calculated using ratio of integrals for olefinic protons of *Z*-**2a** and *E*-**2a** determined by ^{1}H NMR with a formula $I_{Z\text{-}2a}/(I_{Z\text{-}2a}+I_{E\text{-}2a})\times100\%$ for *Z*-**2a** and $I_{E\text{-}2a}/(I_{Z\text{-}2a}+I_{E\text{-}2a})\times100\%$ for *E*-**2a**. [c] Conversion was calculated using ^{1}H NMR integrals for olefinic protons of *Z*-**2a** and *E*-**2a** and aromatic doublet of **1a** (dived by 2 since it integrates for 2H) and was calculated using following formula $(I_{Z\text{-}2a}+ I_{E\text{-}2a})/(I_{Z\text{-}2a}+I_{E\text{-}2a}+(I_{1a}/2))\times100\%$. [d] addition of 18-crown-6 (1.5 equiv.). [e] Low solubility of **1a** observed.

The model substrate **1a** was chosen for the study and the isolated products were analysed with ^{1}H NMR. We first preforformed preliminary experiments with K_2CO_3 as a base. When DPCMP was used as a reagent, low conversion was observed, only 24% (Table S1, entry 1), however, in the case of DECMP a mixture of both isomers were isolated and with full conversion (Table S1, entry 2). It is known that the isomer which is predominantly formed in HWE rection is *E*-isomer. However, in our case reactions preformed with inorganic bases and with DECMP gave mostly mixtures of *E* and *Z*-isomers of **2a** (Table S1, entries 2-13). Highest stereoselectivity up to 75.2% of *E*-**2a** was achieved with potassium carbonate in *t*-butanol (Table S1, entry 4). The reaction did not occur with potassium carbonate in *n*-pentanol, *N*,*N*-dimethylacetamide, *N*,*N*-dimethylformamide (Table S1, entries 4-6) and 3-methyl-1-butanol (Table S1, entry 8). To achieve higher stereoselectivity LiHMDS, NaHMDS and KHMDS bases were used in tetrahydrofuran (THF) at -20 °C (Table S1, entries 14-16). The reaction performed with KHMDS gave highest stereoselectivity, 89.5% of *E*-**2a** (Table S1, entry 16). When DECMP was replaced with DPCMP, the reaction was not as stereoselective, moreover, full conversion was not afforded (Table S1, entries 17-19). Addition of 18-crown-6 improved stereoselectivity in case of LiHMDS (Table S1, entry 20), however, the stereoselectivity dropped in the case KHMDS was used as a base (Table S1, entry 21). If HMDS bases were replaced with LDA, the reaction was not stereoselective with DECMP (Table S1, entry 22), besides, in case of DPCMP there was no reaction (entry 23). Performing the reaction in 2-methyl tetrahydrofuran (2-MeTHF) as a solvent, did not have a greater impact on the stereoselectivity (Table S1, entries 24-25). On the contrary, when THF was

replaced with acetonitrile (MeCN), slightly higher percentages of *Z*-**2a** were formed (Table S1, entries 26-30), although the highest selectivity 78.1% of *Z*-**2a** was obtained with DPCMP in the presence of LiHMDS (Table S1, entry 29). Considering the experiment described in Table S1, entry 15, which by far afforded the highest percentage of *E*-**2a**, the effect of different ether solvents was further explored (Table S1, entries 31-34). Notably, up to 94.8% of *E*-**2a** was obtained in 1,4-dioxane at 15 °C (Table S1, entry 33). The experiment gave satisfactory results, therefore further optimisation was performed.

Table S2. Optimisation of HWE reaction conditions (temperature, equivalents of reagents and concentration)

Entry[a]	DECMP equiv.	KHMDS equiv.	T [°C]	*Z*-**2a** [%][b]	*E*-**2a** [%][b]	Concentration [g/mL]	Conversion [%][c]
1	1.3	1.2	15	5.2	94.8	0.01	100
2	1.3	1.2	23	5.7	94.3	0.01	100
3	1.3	1.2	30	6.7	93.3	0.01	100
4	1.3	1.2	40	6.0	94.0	0.01	100
5	1.05	1.05	23	0.0	100	0.01	60
6[d]	1.1	1.1	23	2.9	97.1	0.01	63
7[d]	1.2	1.2	23	3.8	96.2	0.01	71
8[d]	1.3	1.3	23	2.9	97.1	0.01	77
9[d]	1.4	1.4	23	4.8	95.2	0.01	94
10[d]	1.4	1.5	23	3.9	96.1	0.01	93
11[d]	1.5	1.5	23	5.7	94.3	0.01	100
12	1.4	1.5	23	16.1	83.9	0.02	100
13	1.4	1.5	23	11.7	88.3	0.04	100
14	1.4	1.5	23	11.6	88.4	0.08	100
15	1.4	1.5	23	5.2	94.8	0.005	100
16	1.4	1.5	23	9.3	90.7	0.002	86
17	1.4	1.5	23	7.8	92.2	0.001	46

[a] Reactions were conducted by using **1a** (20 mg, 0.069 mmol) at 0.01 g/mL concentration after the isolation the product were analyzed with ^{1}H NMR in acetone-d_6. [b] The percentage of formed *Z*- and *E*-isomer was calculated using ratio of integrals for olefinic protons of *Z*-**2a** and *E*-**2a** determined by ^{1}H NMR with a formula $I_{Z\text{-}2a}/(I_{Z\text{-}2a}+I_{E\text{-}2a})\times100\%$ for *Z*-**2a** and $I_{E\text{-}2a}/(I_{Z\text{-}2a}+I_{E\text{-}2a})\times100\%$ for *E*-**2a**. [c] Conversion was calculated using ^{1}H NMR integrals for olefinic protons of *Z*-**2a** and *E*-**2a** and aromatic doublet of **1a** (dived by 2, since it integrates for 2H) and was calculated using following formula $(I_{Z\text{-}2a}+I_{E\text{-}2a})/(I_{Z\text{-}2a}+I_{E\text{-}2a}+(I_{1a}/2))\times100\%$. [d] Additional extraction of water phases with ethyl acetate were performed and all organic phases were combined to extract all **1b** and **2b**.

With the optimal solvent and reagent, the reactions were exposed to different temperatures from 15 – 40 °C and no major impact on the stereoselectivity was observed (Table S2, entries 1-4). In further studies of the effect of different equivalents of DECMP and KHMDS (Table S2, entries 5-11), the stereoselectivity was not severely impacted, however the full conversion was achieved with 1.5 equivalents of both DECMP and KHMDS (Table S2, entry 11). In parallel, we studied the effect of the concentration on conversion and stereoselectivity (Table S2, entry 12-17). With higher concentration of the reaction mixture, stereoselectivity of the reaction was reduced (Table S2, entry 12-14). However, on the other hand, with dilution of the reaction mixtures, stereoselectivity was not greatly affected, instead it had an impact on decreasing of conversion of the reaction.

As a result of this optimization study, optimal conditions were determined to be as follows: 1.5 equiv. of DECMP, 1.5 equiv. of 1 M KHMDS in THF, dry 1,4-dioxane at 0.01 g/mL, 23 °C, 20 h (Table S2, entry 11).

3.2. Optimisation of Isolation

In all **2a** products, which were isolated previously via extraction with ethyl acetate and washed with water and brine, impurities (mostly residual DECMP) were present in the ^{1}H NMR spectra. In addition, isolated products were mostly oily. Further optimisation of extraction, such as replacing ethyl acetate with isopropyl acetate or isobutyl acetate; or additional washing of organic phase with brine or different concentration of water solution of potassium hydrogen carbonate; did not bring any promising results in isolation of the pure product. Nevertheless, when experiments on larger scale (300 mg) and with different substrates were conducted, it was observed that not all products **2** are soluble in ethyl acetate, moreover, some remained in the water phase and over the night, the product precipitate from the water phases. Therefore, the idea of isolation of the products **2** via precipitation arose.

3.2.1. Optimisation of Isolation with Precipitation form MeCN and 10% $KHCO_3$ solution

For the studies of precipitation process, we performed reactions under optimized reaction conditions. After the reaction completion, the product was isolated as written in the Table **S3**.

Table S3: Optimisation of isolation of aryl products **2**.

Entry[a]	Substrate	Optimisation of precipitation	*Z*-**2** [%][b]	*E*-**2** [%][b]	Purity [%]	Yield [%]
1	**1g**	Half of the 1,4-dioxane was evaporated from the reaction mixture, 15 mL of 10% water solution of $KHCO_3$ was added and the mixture was stirred on an ice bath for 2 h. Precipitated product was filtered and washed with cold water and cold MTBE and dried.	0	100	86	41.5
2	**1a**	The reaction mixture was evaporated to the dry residual, 1 mL of 1,4-dioxane was added, after 20 mL of 10% water solution of $KHCO_3$ was added and stirred at 0 °C for 20 h. Precipitated product was filtered and washed with cold water and cold MTBE and dried.	0	100	97.7	69.7
3	**1a**	The reaction mixture was evaporated to the dry residual, 1 mL of acetonitrile was added, after 20 mL of 10% water solution of $KHCO_3$ was added and stirred at 0 °C for 20 h. Precipitated product was filtered and washed with cold water and cold MTBE and dried.	0	100	97	72.8
4	**1a**	The reaction mixture was evaporated to remain wet residual, 0.5 mL of acetonitrile was added, after 20 mL of 10% water solution of $KHCO_3$ was added and stirred at 0 °C for 20 h. Precipitated product was filtered and washed with cold water and cold MTBE and dried.	0	100	100	75
5[c]	**1a**	The reaction mixture was evaporated to remain wet residual (~1 mL), 1.5 mL of acetonitrile was added, after 30 mL of 10% water solution of $KHCO_3$ was added and stirred at 0 °C for 20 h. Precipitated product was filtered and washed with cold water and cold MTBE and dried.	0	100	100	80

[a] Reactions were conducted under optimised reaction condition by using **1a** (100 mg, 0.344 mmol) or **1g** (100 mg, 0.422 mmol) at 0.01 g/mL concentration and reactions were left over night. Next day the isolation was performed as described in the table above. [b] The percentage of formed *Z*- and *E*-isomer was calculated using ratio of integrals for olefinic protons of *Z*-**2** and *E*-**2** determined by ^{1}H NMR with a formula $I_{Z\text{-}2}/(I_{Z\text{-}2}+I_{E\text{-}2})\times100\%$ for *Z*-**2** and $I_{E\text{-}2}/(I_{Z\text{-}2}+I_{E\text{-}2})\times100\%$ for *E*-**2**. [c]Reaction was conducted under optimised reaction condition by using **1a** (300 mg, 1.03 mmol) 0.01 g/mL

concentration and reaction was left over night. Next day the isolation was performed as described in the table above. White solid product was isolated in 80% yield (265 mg).

Pure *E*-isomers were isolated under all attempts of the isolation with precipitation. The excess of 1,4-dioxane in the reaction mixture influence on lower precipitation of the product and consequently on lower yield 41.5% (Table S3, entry 1). In addition, we noticed that it was crucial that 1,4-dioxane was not fully evaporated from the reaction mixture, since this influenced on lower purity of the isolated product (Table S3, entry 2). However, after the addition of acetonitrile, the wet residual of the reaction mixture was easily mixed. After the addition of the 10% aqueous solution of $KHCO_3$, the precipitation of the product slowly initiated. The optimal conditions of isolation were performed on 300 mg scale of **1a** obtaining pure *E*-**2a** in 80% yield (Table S3, entry 5).

3.2.2. Optimisation of Isolation with Crystallisation from 2-PrOH for Alkyl Substrates

On contrary to the aromatic products **2**, we were not able to isolate alkyl products **2** with the precipitation from acetonitrile and 10% $KHCO_3$ solution, since all were mostly soluble in water. In addition, also extractions were not the option, since a side product of DECMP, potassium salt of diethyl phosphate is soluble in water. Moreover, attempts with the flash chromatography were not successful, since coelution of phosphate was observed and also it is already known that trifluoroborates are not stable on silica. After few unsuccessful isolations we tried to find a solvent in which potassium diethyl phosphate would be soluble and the alkyl products **2** would potentially crystallise. Different alcohols were explored. The reactions were performed under optimised reaction conditions and after, 1,4-dioxane was fully evaporated and different alcohols were added. The mixture was stirred at room temperature for 1 h and then slowly cooled down to -10 °C and stirred for 20 h.

Table S4: Optimisation of isolation of alkyl products **2.**

Entry[a]	Substrate	Alcohol	*Z*-**2** [%][b]	*E*-**2** [%][b]	Purity [%]	Yield [%]
1	1v	methanol	0	100	93	4.5
2	1v	ethanol	0	100	95	9.9
3	1v	isopropanol	0	100	100	39.8
4	1v	1-butanol	0	100	50	51.6
5[c]	1v	isopropanol	2	98	84	55.3
6[d]	1v	isopropanol	3	97	97	56.9

[a] Reactions were conducted by using **1v** (100 mg, 0.458 mmol) at 0.01 g/mL concentration and reactions were left over night. Next day the isolation was performed as described above. [b] The percentage of formed *Z*- and *E*-isomer was calculated using ratio of integrals for olefinic protons of *Z*-**2** and *E*-**2** determined by ^{1}H NMR with a formula $I_{Z\text{-}2}/(I_{Z\text{-}2}+I_{E\text{-}2})\times100\%$ for *Z*-**2** and $I_{E\text{-}2}/(I_{Z\text{-}2}+I_{E\text{-}2})\times100\%$ for *E*-**2**. [c] 300 mg experiment (**1v**, 1.38 mmol). [d] 300 mg experiment (**1v**, 1.38 mmol), after isopropanol was added to the evaporated residual, the mixture was heated to the reflux and then cooled to -10 °C.

The lowest yield was afforded with crystallisation in methanol and ethanol (Table S4, entries 1 and 2). The highest purity and yield of *E*-**2v** was achieved in isopropanol (Table S4, entry-3), while in case of 1-butanol additional impurities were isolated (Table S4, entry 4). In the scale up experiment of the isolation on 300 mg scale (entry 5) in isopropanol, lower purity (84%) of the product was observed. Further optimisation with the inclusion of heating of the isopropanol mixture to the reflux followed by slow cooling to -10 °C, pure product **2v** was isolated with only 3% of *Z*-**2v**.

3.3. Determination of stereoselectivity of α,β-unsaturated β-boryl nitriles

For determination of the stereochemistry of the formed α,β-unsaturated β-boryl nitriles **2a**, the 2D NOESY spectrum of mixture of obtained products **2a** was studied (Figures S2-S3). The two-dimensional ^{1}H-^{1}H NOESY spectra recorded in DMSO-d_6 for the mixture of compounds *E*-**2a/***Z*-**2a** revealed the cross peak between the olefinic proton (NC-C**H**=C(*p*-Br-Ph)BF_3K) located at 5.60 ppm and the ortho proton on the aromatic substituent located at 7.36 ppm for the major isomer, which was also obtained in pure form after isolation. These observations support the *E*-geometry of the main isolated product. The absence of nOe between the same protons located at 5.57 ppm in minor isomer present in the mixture, is indicative of its *Z*-geometry (Figures S2-S3). Similar observations were obtained for additional aromatic products **2g** and **2i** on reaction mixtures and isolated pure aliphatic products **2s** and **2v** (vide infra) (Figures S4-S7).

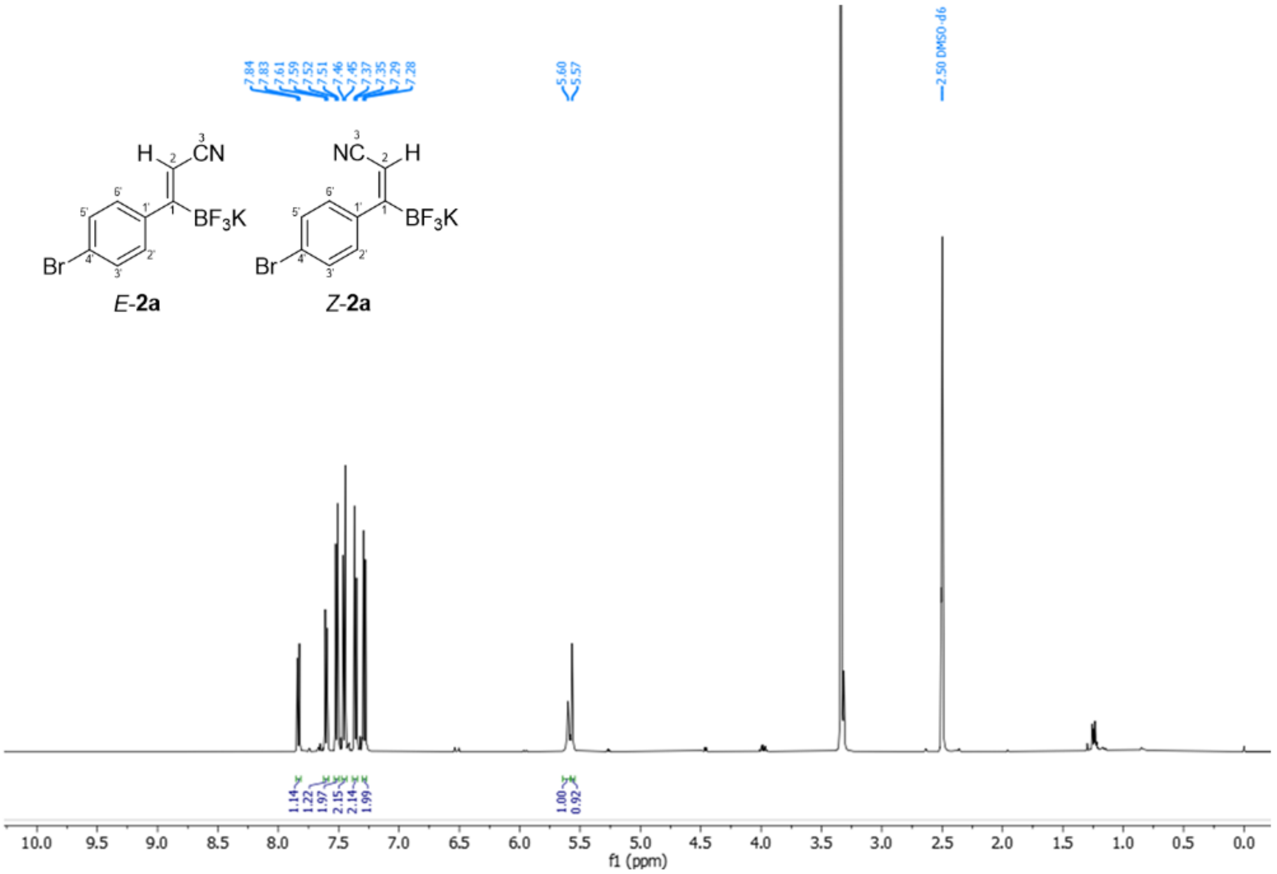

Figure S2: ^{1}H-NMR (500 MHz, DMSO-d_6) of the mixture of *Z*-**2a** and *E*-**2a**

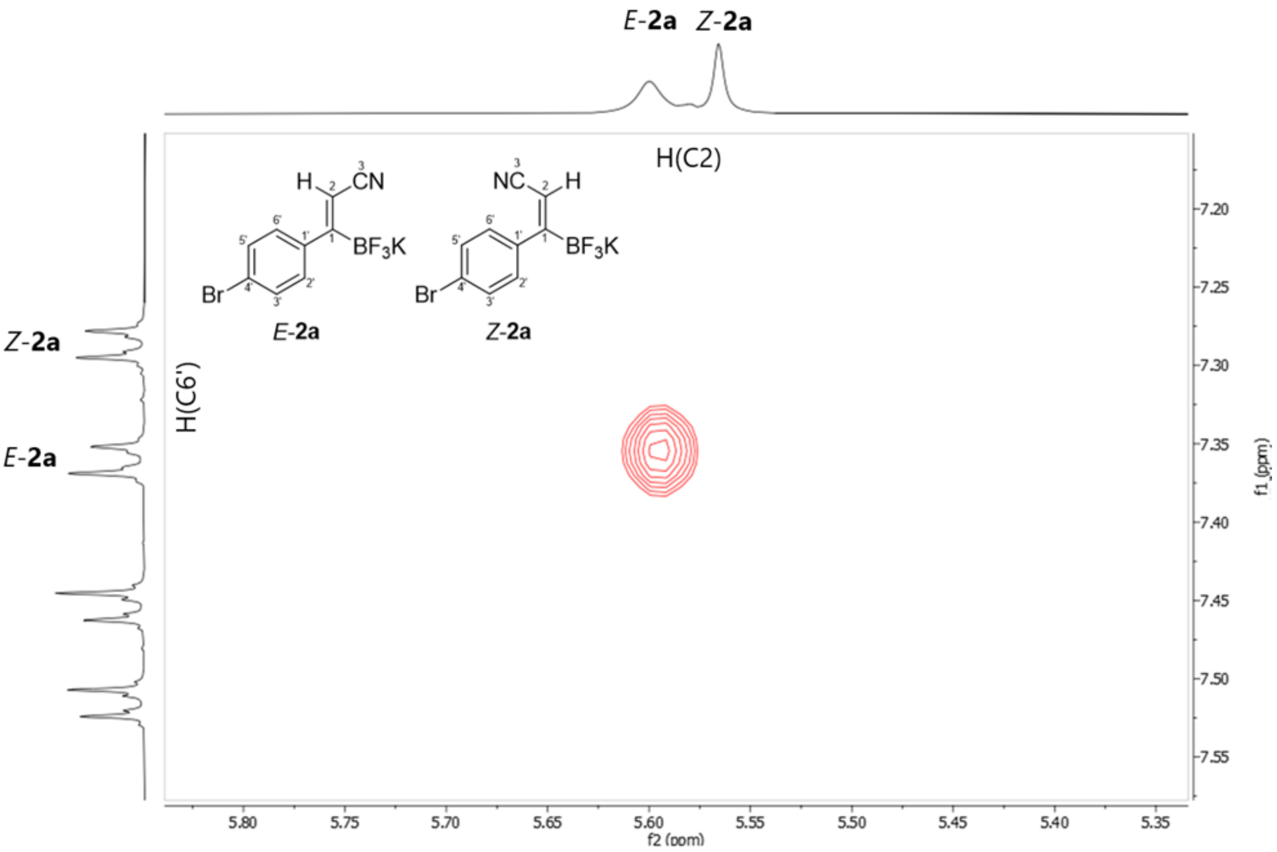

Figure S3: ^{1}H-^{1}H NOESY spectrum (DMSO-d_6) of the mixture of *Z*-**2a** and *E*-**2a**.

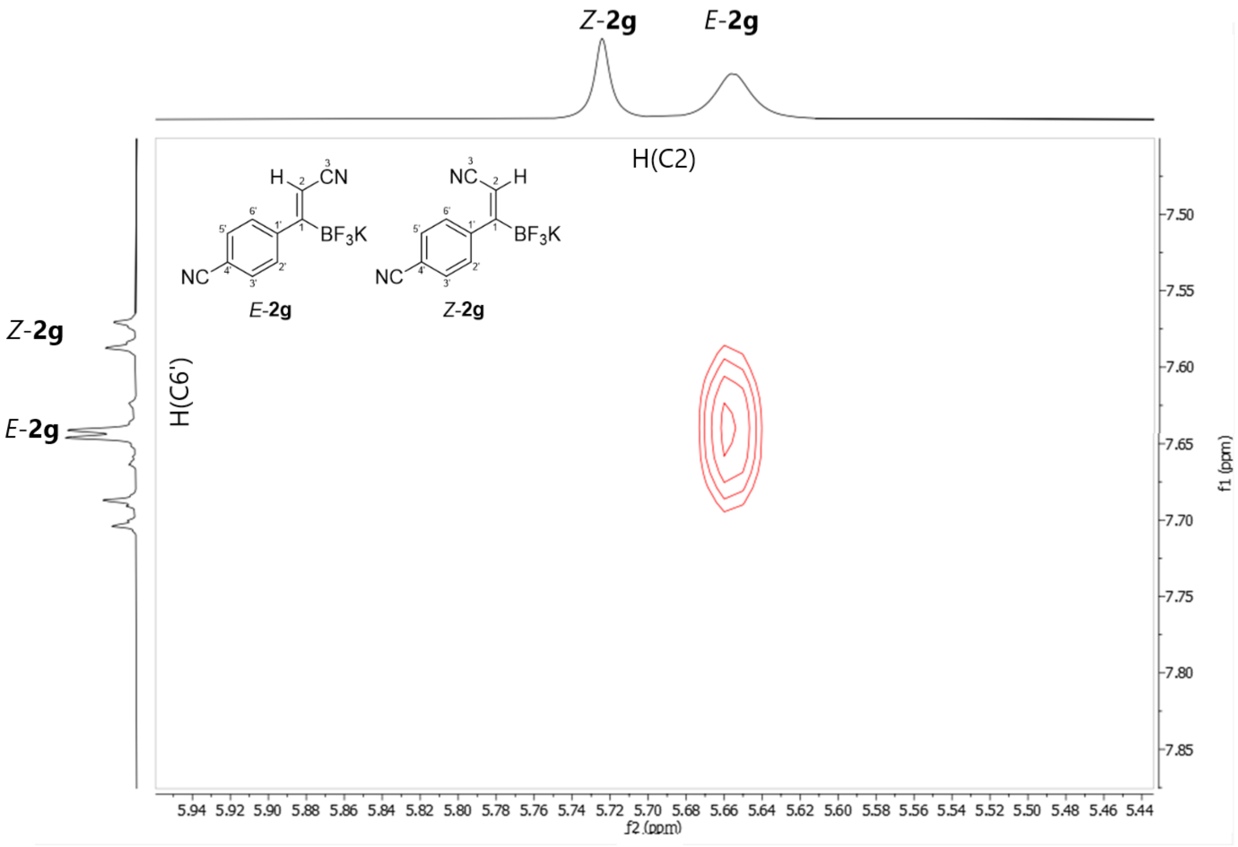

Figure S4: ^{1}H-^{1}H NOESY spectrum (acetone-d_6) of the mixture of *Z*-**2g** and *E*-**2g**.

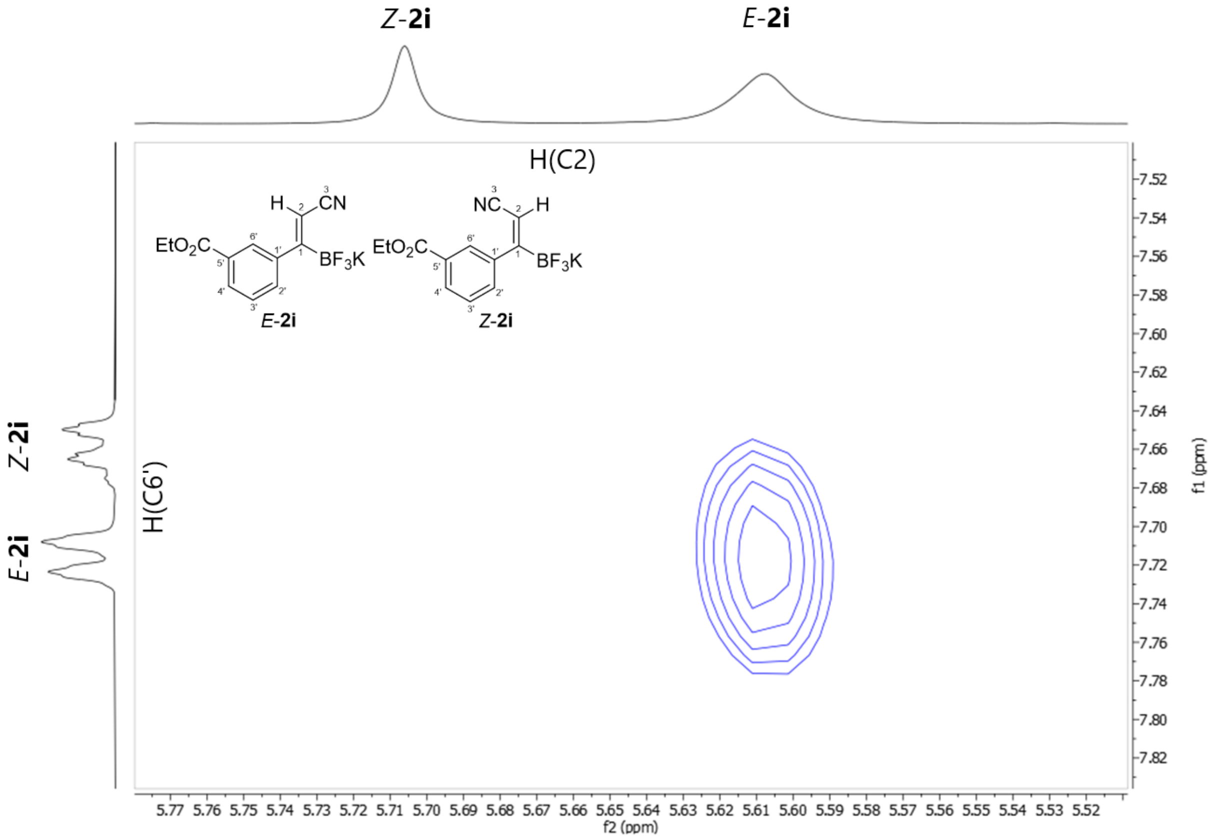

Figure S5: ^{1}H-^{1}H NOESY spectrum (acetone-d_6) of the mixture of *Z*-**2i** and *E*-**2i**.

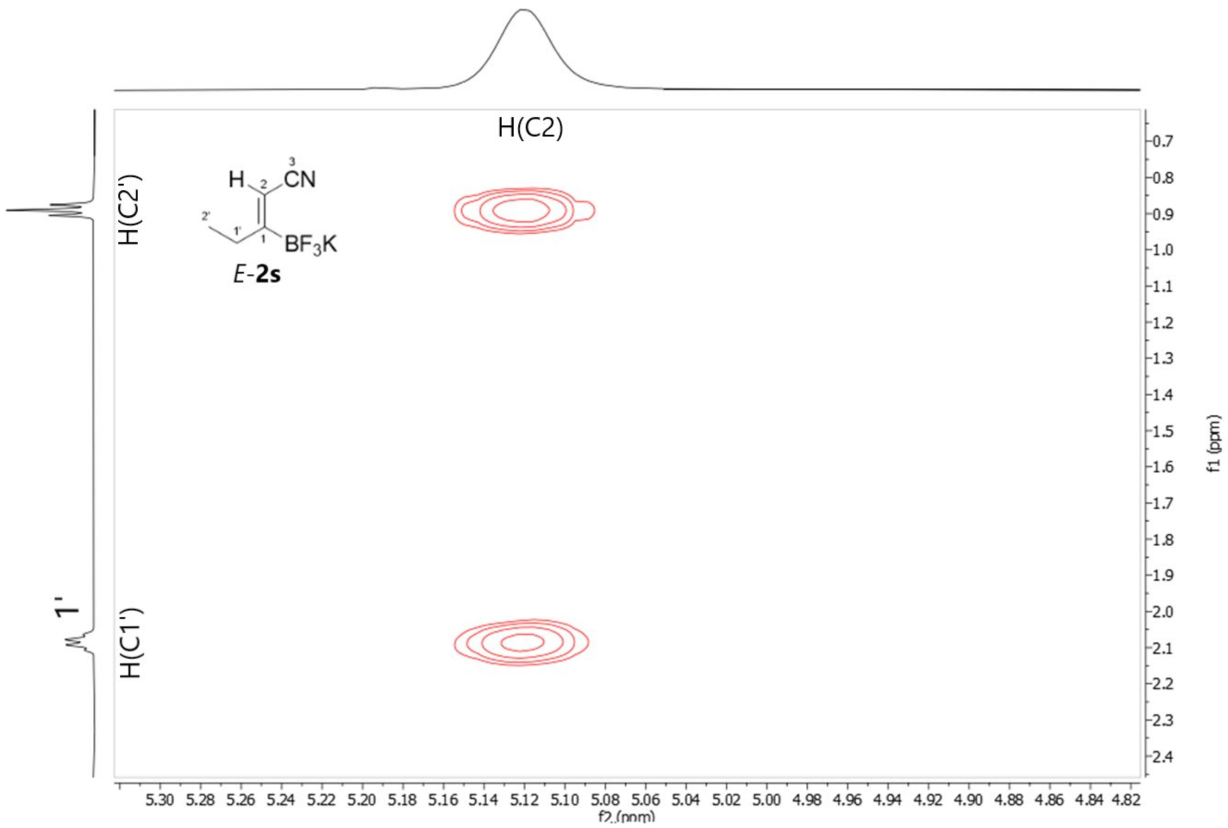

Figure S6: ^{1}H-^{1}H NOESY spectrum (DMSO-d_6) of the *E*-**2s.**

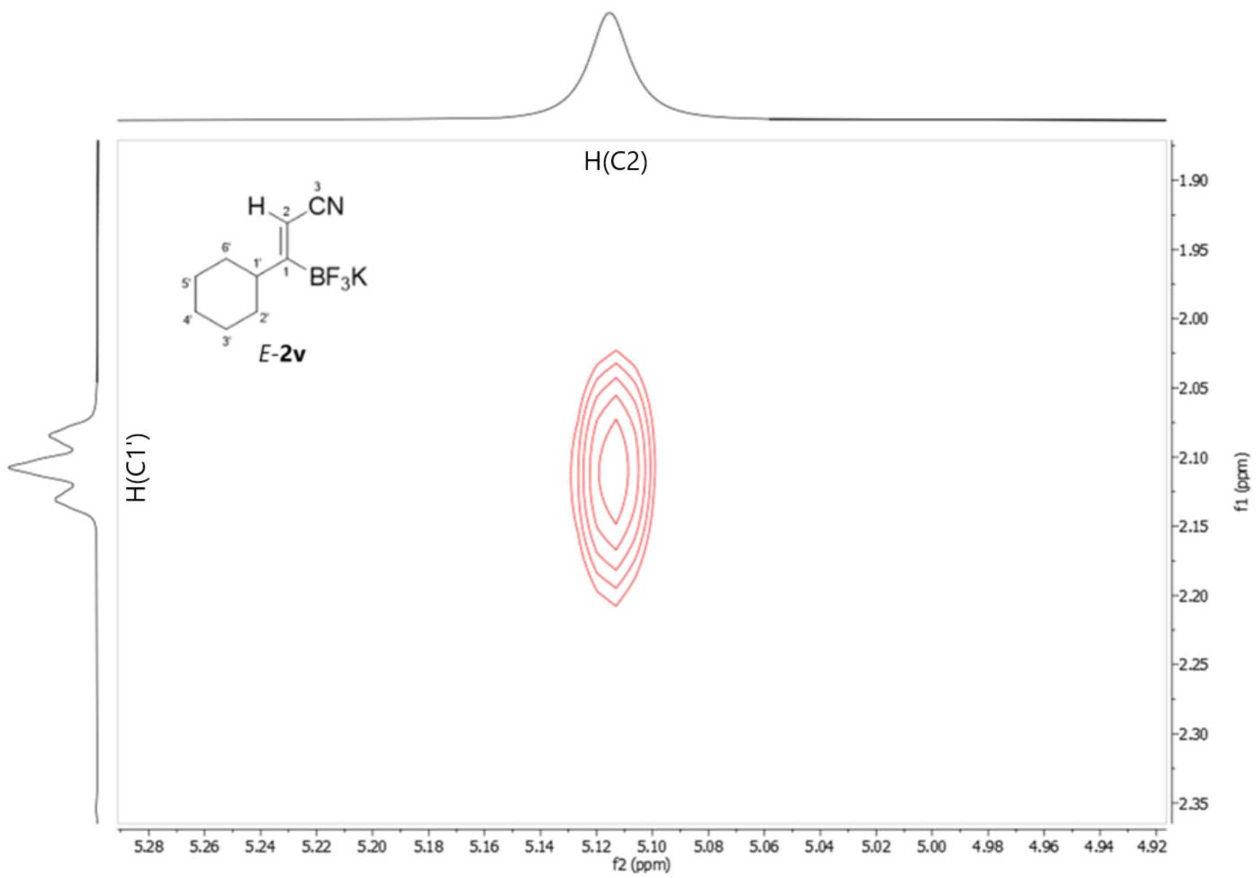

Figure S7: ^{1}H-^{1}H NOESY spectrum (DMSO-d_6) of the *E*-**2v**

3.4. General Procedure for the Synthesis of *E*-α,β-unsaturated β-boryl nitriles

In the round bottom flask closed with septum, dry 1,4-dioxane (30 mL) and DECMP (1.5 equiv) was charged and purged with nitrogen at room temperature. Afterwards, KHMDS (1M in THF, 1.5 equiv) was added, and the reaction mixture was allowed to stir for 1 h at room temperature. Then the substrate **1** (300 mg) was added as a solid, and the reaction mixture was purged with nitrogen. The reaction mixture was allowed to stir for 18 h at room temperature. After, 200 µL of the reaction mixture was separately evaporated and dissolved in *d*-DMSO, to determine conversion of the reaction with ^{1}H NMR.

A) Isolation with precipitation form MeCN and 10% $KHCO_3$ mixture (products **2a-r**).

After 18 h solvent was evaporated to the final volume of ~1mL. To the wet residual 1.5 mL of acetonitrile was added and dropwise 30 mL of 10% $KHCO_3$ solution was added. After, the mixture was allowed to cool down to -5 °C and stirred for 20 h. Precipitated product was filtrated and washed with cold water and MTBE.

B) Isolation with crystallisation form 2-PrOH (products **2s-x**).

After 18 h the solvent was evaporated. To the dry residual 15 mL isopropanol was added and the mixture was heated to reflux and stirred at reflux for 1 h. The mixture was slowly cooled to -10 °C and stirred for 20 h. Precipitated product was filtrated and washed with cold isopropanol.

Table S5: Results of the synthesis of *E*-α,β-unsaturated β-boryl nitriles

N	Product	Conversion[a] [%]	Reaction mixture					Isolated product					Isolation method	Purity [%]	Yield [%]
			Z-**2**[b]	*E*-**2**[b]	Ratio *Z*-**2** : *E*-**2**[c]	*Z*-**2** [%][d]	*E*-**2** [%][d]	*Z*-**2**[b]	*E*-**2**[b]	Ratio *Z*-**2** : *E*-**2**[c]	*Z*-**2** [%][d]	*E*-**2** [%][d]			
1	**2a**	100	0.07	1	1:14.3	7	93	0	1	0:1	0	100	A	98	80
2	**2b**	100	0	1	0:100	0	100	0	1	0:1	0	100	A	100	68
3	**2c**	100	0.12	1	1:8.3	11	89	0	1	0:1	0	100	A	100	69
4	**2d**	100	0.13	1	1:7.7	12	88	0	1	0:1	0	100	A	100	72
5	**2e**	100	0.17	1	1:5.9	15	85	0	1	0:1	0	100	A	100	68
6	**2f**	100	0.13	1	1:7.7	12	88	0	1	0:1	0	100	A	100	63
7*	**2g**	100	0.09	1	1:11.1	8	92	0	1	0:1	0	100	A	100	76

N	Product	Conversion[a] [%]	Reaction mixture					Isolated product					Isolation method	Purity [%]	Yield [%]
			Z-**2**[b]	*E*-**2**[b]	Ratio *Z*-**2** : *E*-**2**[c]	*Z*-**2** [%][d]	*E*-**2** [%][d]	*Z*-**2**[b]	*E*-**2**[b]	Ratio *Z*-**2** : *E*-**2**[c]	*Z*-**2** [%][d]	*E*-**2** [%][d]			
8	**2h**	100	0.09	1	1:11.1	8	92	0	1	0:1	0	100	A	97	75
9	**2i**	100	0.10	1	1:10	9	91	0	1	0:1	0	100	A	100	73
10	**2j**	100	0.11	1	1:9.1	10	90	0	1	0:1	0	100	A	100	68
11	**2k**	100	0.13	1	1:7.7	12	88	0	1	0:1	0	100	A	100	47
12	**2l**	100	0.11	1	1:9.1	10	90	0.05	1	1:20	5	95	A	95	66
13	**2m**	98	0.25	1	1:4	20	80	0	1	0:1	0	100	A	100	62
14	**2n**	95	0.25	1	1:4	20	80	0	1	0:1	0	100	A	90	52
15	**2n**	98	0.28	1	1:3.6	22	78	0	1	0:1	0	100	B	100	53
16	**2o**	100	0.25	1	1:4	20	80	0	1	0:1	0	100	A	100	34
17	**2p**	100	0.18	1	1:5.6	15	85	0.02	1	1:50	2	98	A	98	61
18	**2q**	100	0.14	1	1:7.1	12	88	0	1	0:1	0	100	A	100	67
19	**2r**	100	0.29	1	1:3.4	22	78	0.02	1	1:50	2	98	A	98	27
20	**2r**	100	0.24	1	1:4.3	19	81	0	1	0:1	0	100	B	97	53
21	**2s**	100	0.41	1	1:2.4	29	71	0	1	0:1	0	100	B	100	23
22	**2t**	79	0.59	1	1:1.7	37	63	0	1	0:1	0	100	B	100	36
23	**2u**	0	/	/	/	/	/	/	/	/	/	/	B	/	/
24	**2v**	100	0.16	1	1:6.3	14	86	3.13	97	1:31	3	97	B	97	57
25	**2w**	94	0.33	1	1:3	25	75	0	1	0:1	0	100	B	100	60
26	**2x**	100	0.58	1	1:1.8	37	63	0	1	0:1	0	100	B	97	47

[a] Conversion was calculated using ^{1}H NMR integrals for olefinic protons of *Z*-**2a** and *E*-**2a** and aromatic doublet of **1a** (dived by 2 since it integrates for 2H) and was calculated using following formula $(I_{Z\text{-}2a}+ I_{E\text{-}2a})/(I_{Z\text{-}2a}+I_{E\text{-}2a}+(I_{E\text{-}1a}/2))\times100\%$. [b] 1H NMR integrals for olefinic protons of *Z*-**2** and *E*-**2** determined by ^{1}H NMR. [c] Ratio of integrals for olefinic protons of *Z*-**2** and *E*-**2** determined by ^{1}H NMR. [d] The percentage of formed *Z*- and *E*-isomer was calculated using ratio of integrals for olefinic protons of *Z*-**2** and *E*-**2** determined by ^{1}H NMR with a formula $I_{Z\text{-}2}/(I_{Z\text{-}2}+I_{E\text{-}2})\times100\%$ for *Z*-**2** and $I_{E\text{-}2}/(I_{Z\text{-}2}+I_{E\text{-}2})\times100\%$ for *E*-**2**.

A. Aryl and heteroaryl substrates:

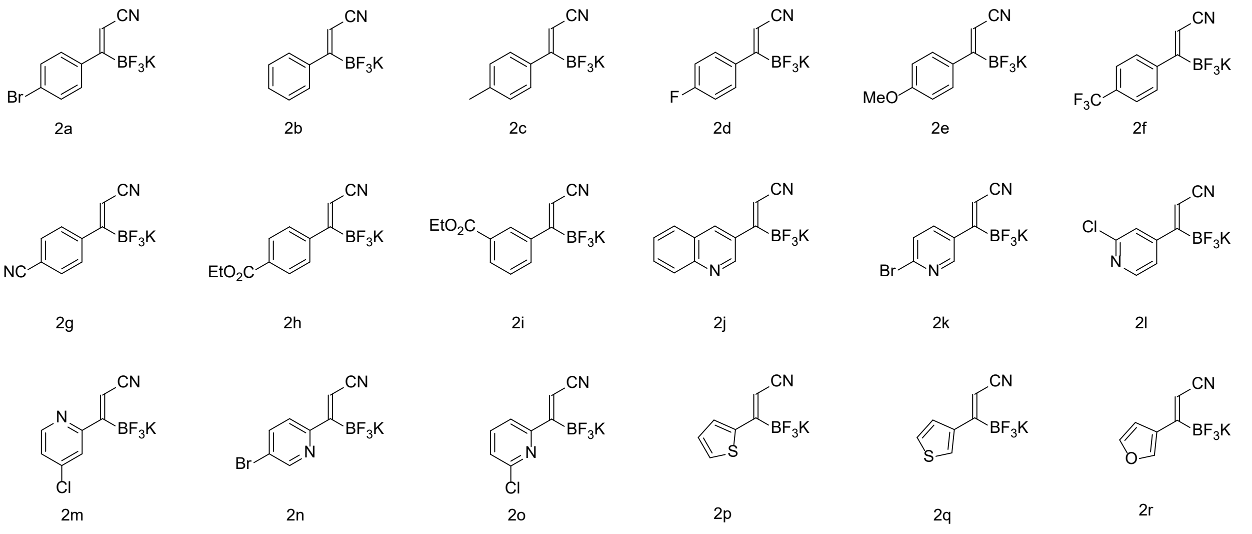

B. Alkyl substrates:

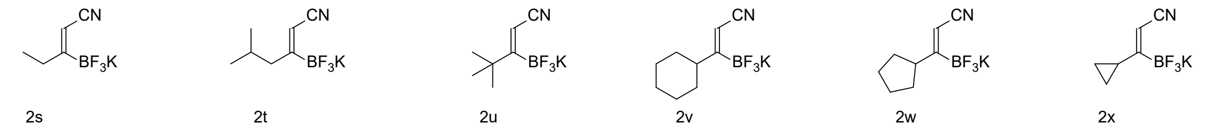

Figure S8 List of synthesized products **2**

4. References

S1. Zhang, T. Y.; O'Toole, J. C.; Dunigan, J. M. An efficient and practical synthesis of diphenyl cyanomethylenephosphonate: Applications to the stereoselective synthesis of cis-α,β-unsaturated nitriles. *Tetrahedron Lett.* **1998**, *39*, 1461–1464. https://doi.org/10.1016/S0040-4039(97)10836-X

S2. Nakahara M.; Kurahayashi K.; Hanaya, K.; Sugai, T.; Higashibayashi, S. One-Step Synthesis of Acylborons from Acyl Chlorides through Copper-Catalyzed Borylation with Polystyrene-Supported PPh_3 Ligand. *Org. Lett.* **2022**, *24*, 5596–5601. https://doi.org/10.1021/acs.orglett.2c02305

S3. Liu, S. M.; Wu, D.; Bode, J. W. One-Step Synthesis of Aliphatic Potassium Acyltrifluoroborates (KATs) from Organocuprates. *Org. Lett.* **2018**, *20*, 2378–2381. https://doi.org/10.1021/acs.orglett.8b00720

S4. Šterman, A.; Košmrlj, J.; Žigart, N.; Gobec, S.; Sosič, I.; Časar, Z. Catalytic Approach to Diverse α-Aminoboronic Acid Derivatives by Iridium-Catalyzed Hydrogenation of Trifluoroborate-Iminiums *Adv*. *Synth*. *Catal*. **2021**, *363*, 2396–2402. https://doi.org/10.1002/adsc.202001350

5. NMR Spectra

(*E*)-3-(4-bromophenyl)-3-(trifluoro-λ[4]-boraneyl)acrylonitrile, potassium salt (2a)

^{1}H-NMR (500 MHz, acetone-d_6) of the reaction mixture of **2a**:

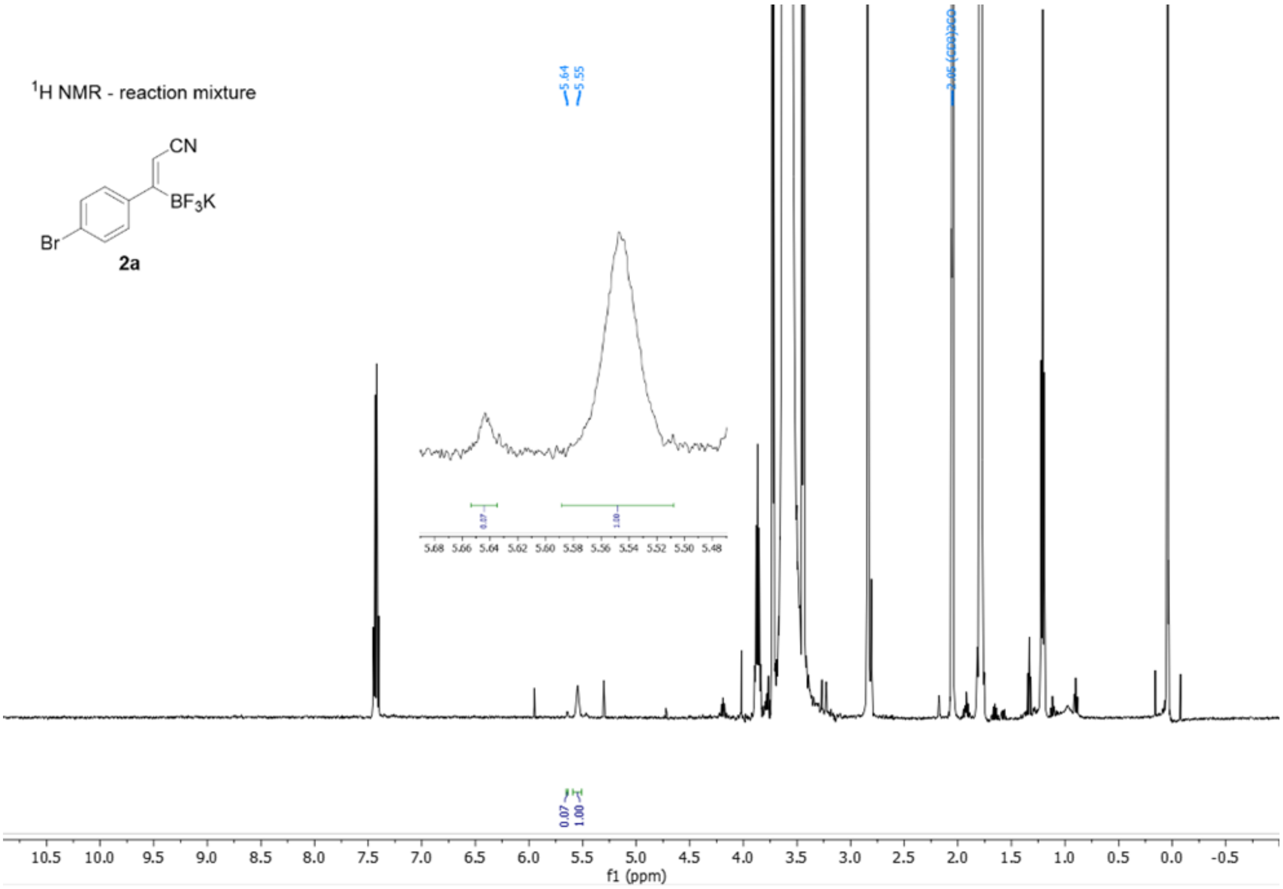

^{1}H-NMR (500 MHz, DMSO-d_6) of **2a**:

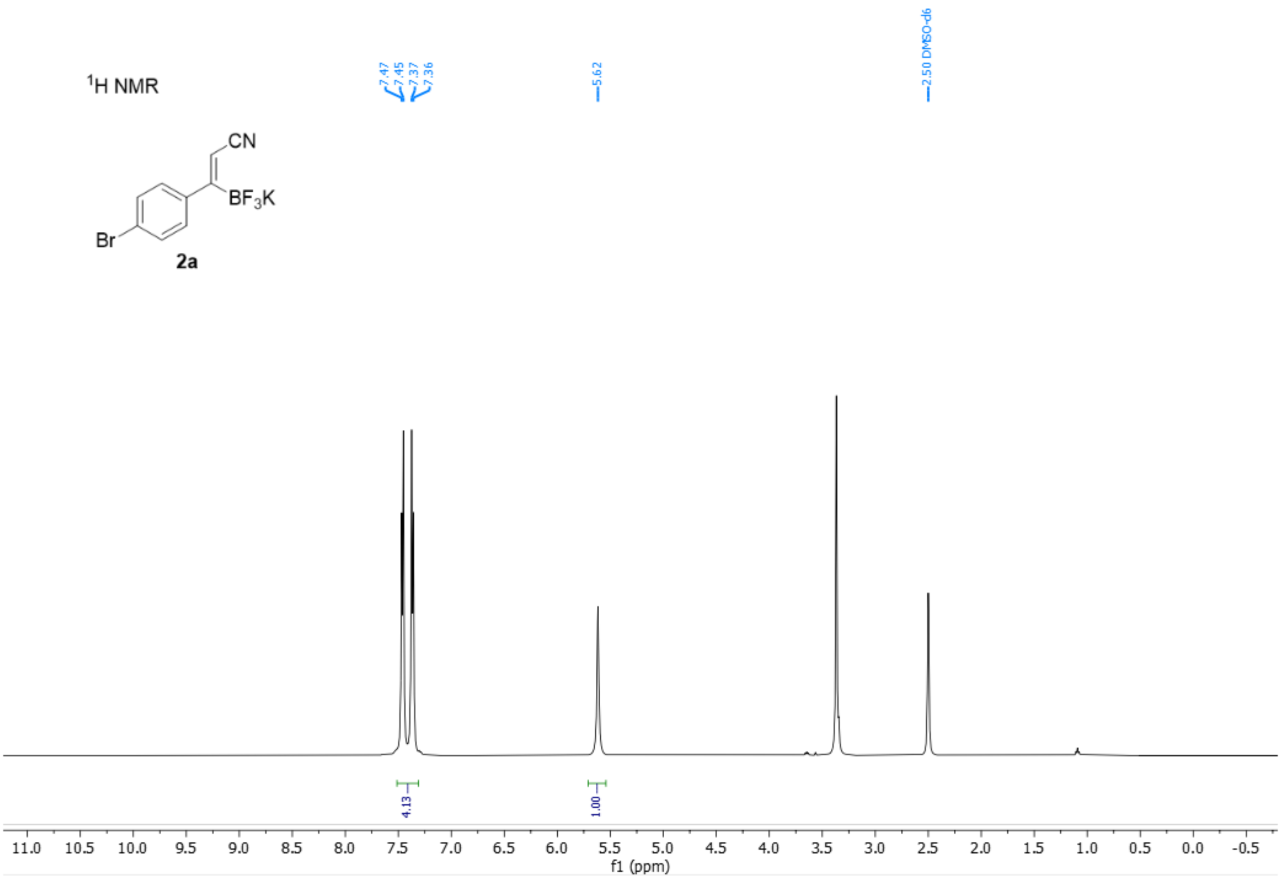

^{13}C{^{1}H}-NMR (126 MHz, DMSO-d_6) of **2a**:

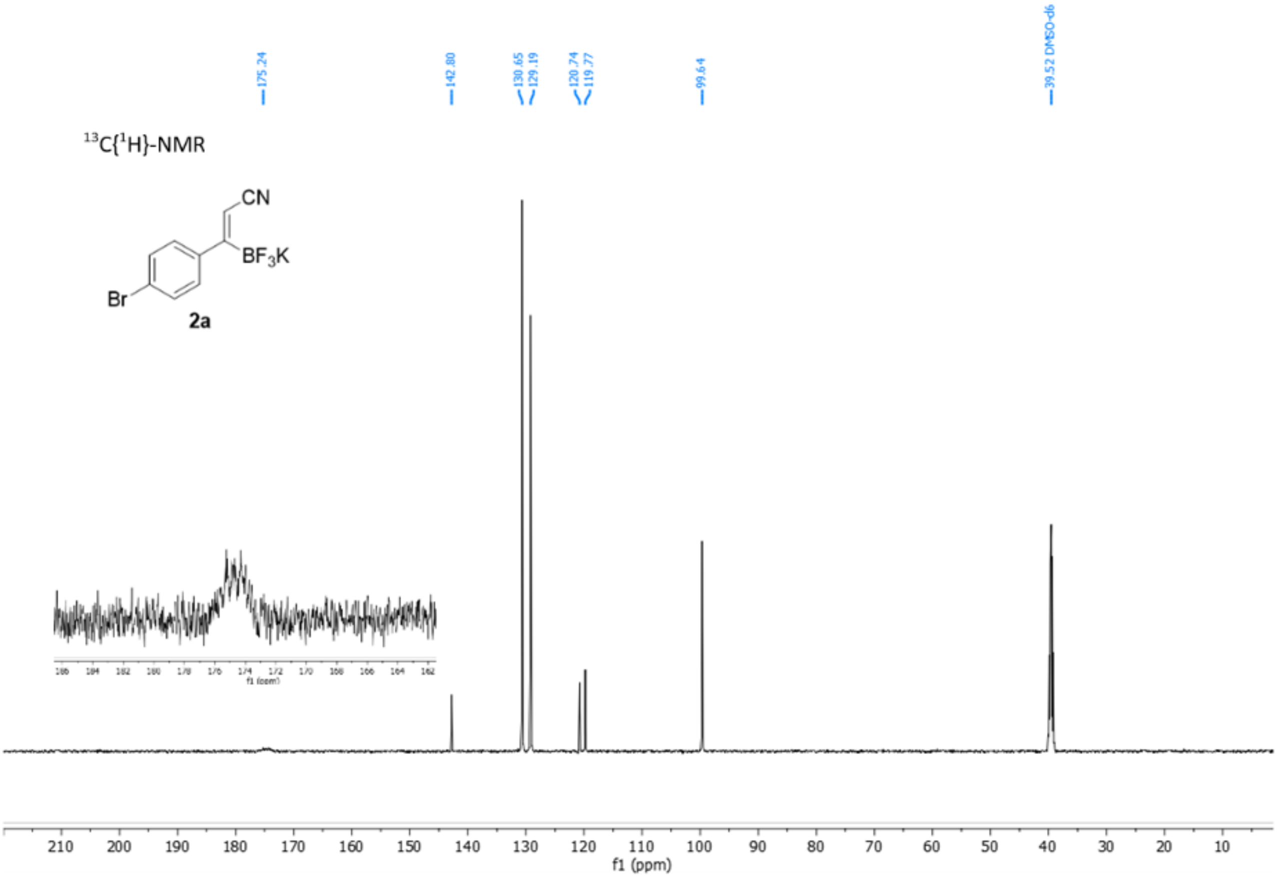

^{11}B-NMR (160 MHz, DMSO-d_6) of **2a**:

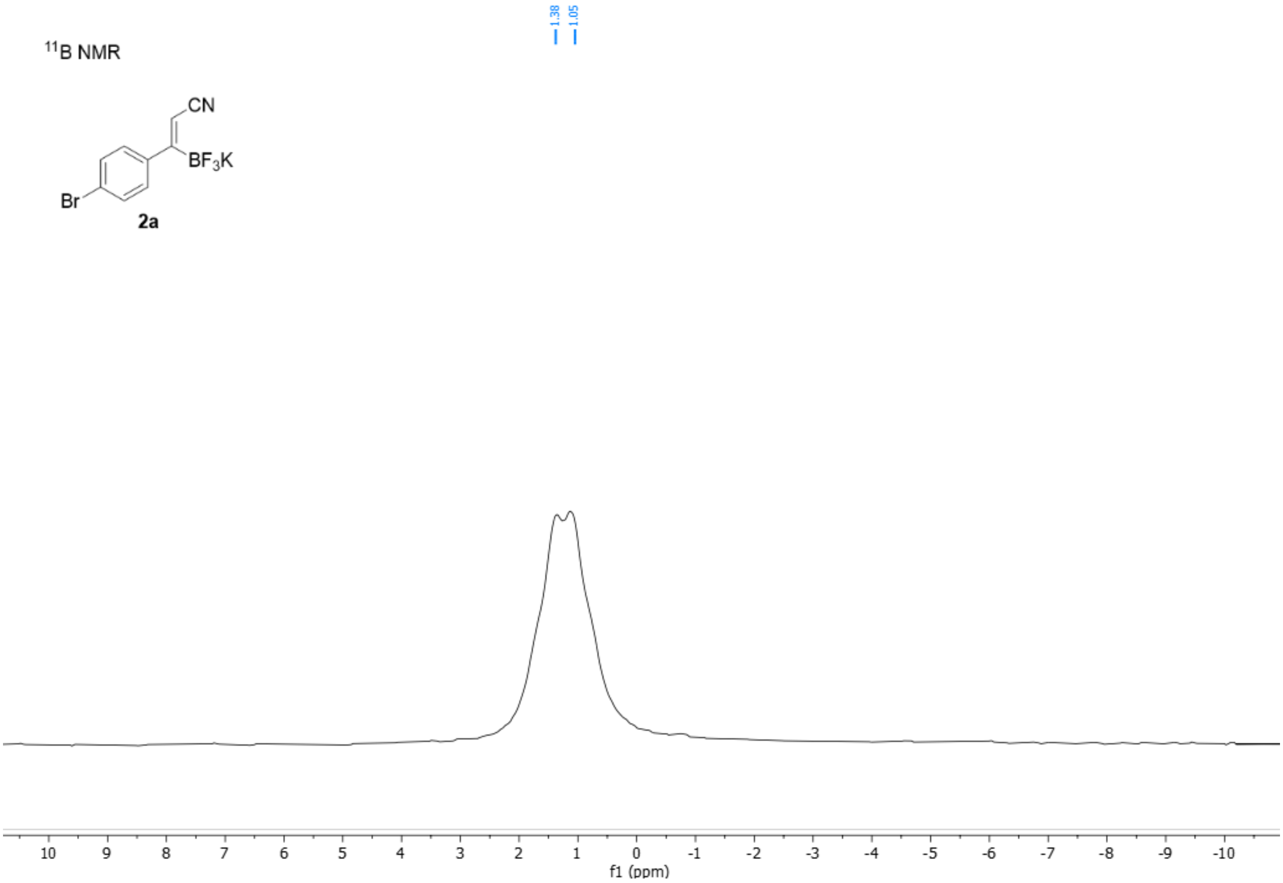

^{19}F-NMR (470 MHz, DMSO-d_6) of **2a**:

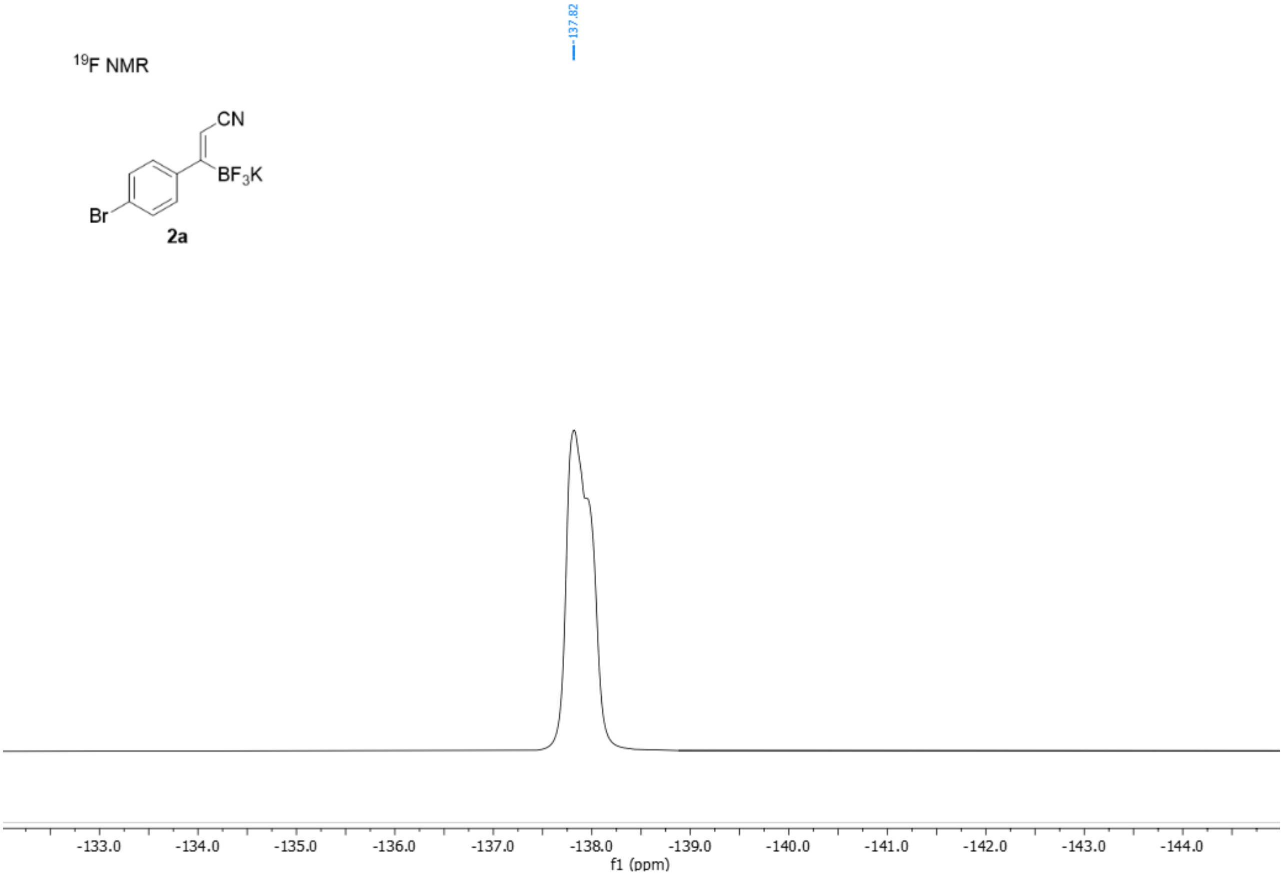

(*E*)-(2-cyano-1-phenylvinyl)trifluoroborate, potassium salt (2b)

^{1}H-NMR (500 MHz, DMSO-d_6) of the reaction mixture of **2b**:

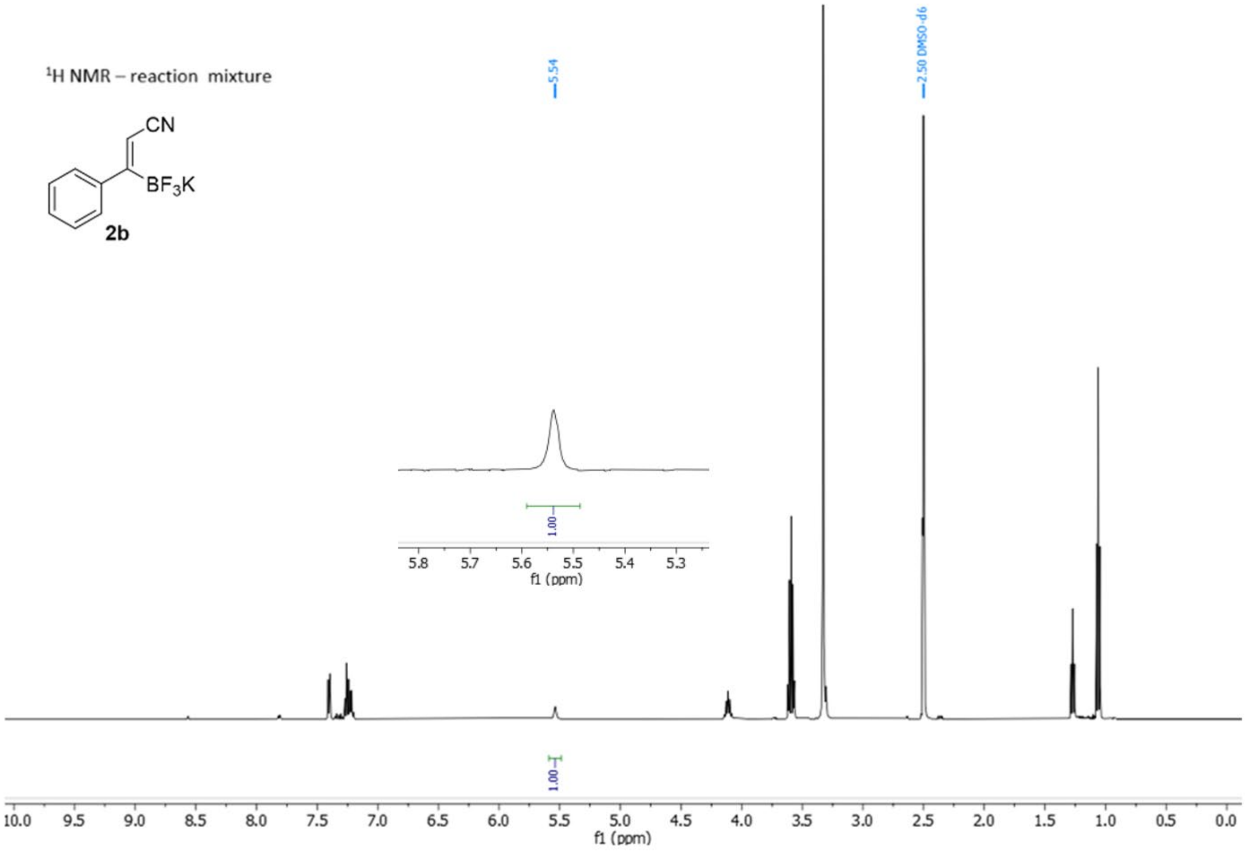

^{1}H-NMR (500 MHz, DMSO-d_6) of **2b**:

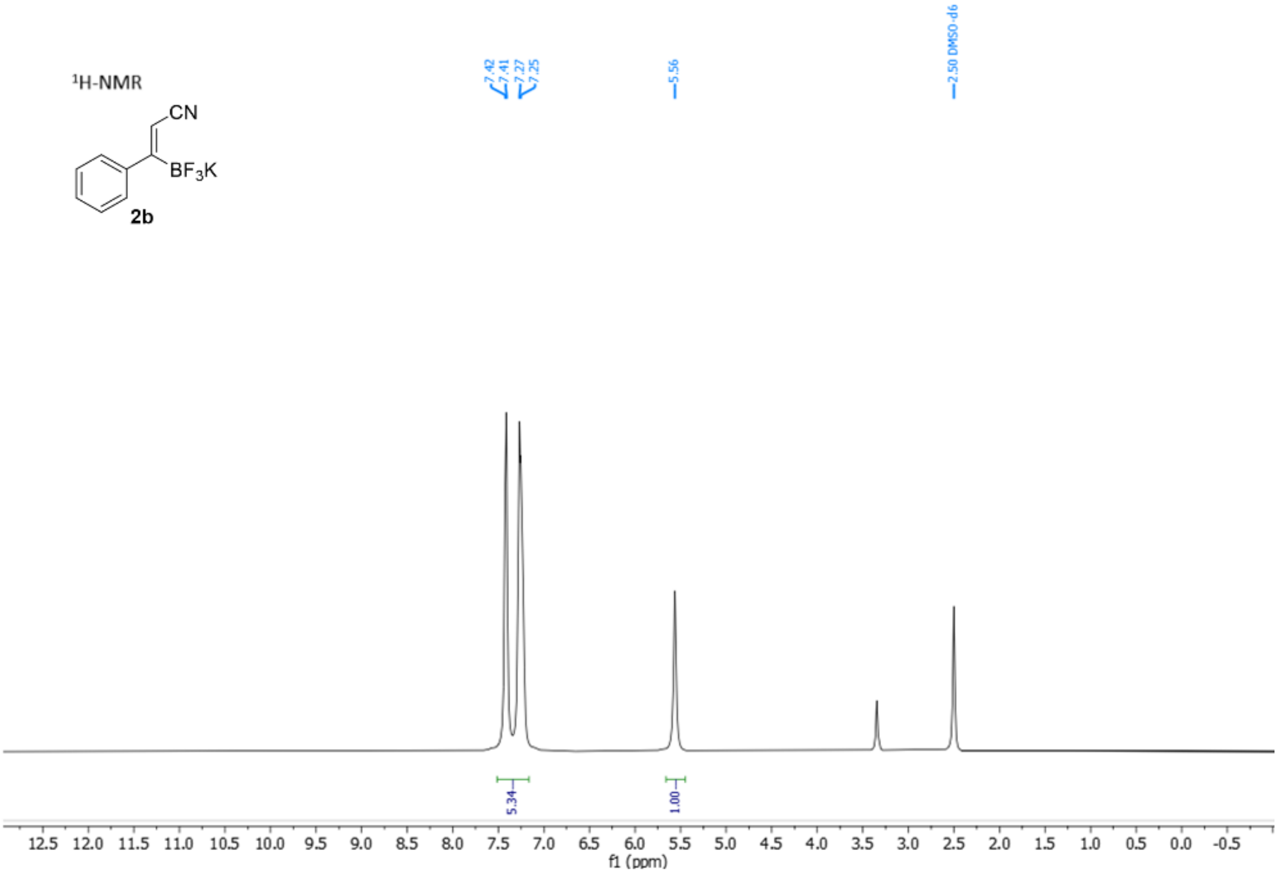

$^{13}C\{^1H\}$-NMR (126 MHz, DMSO-d_6) of **2b**:

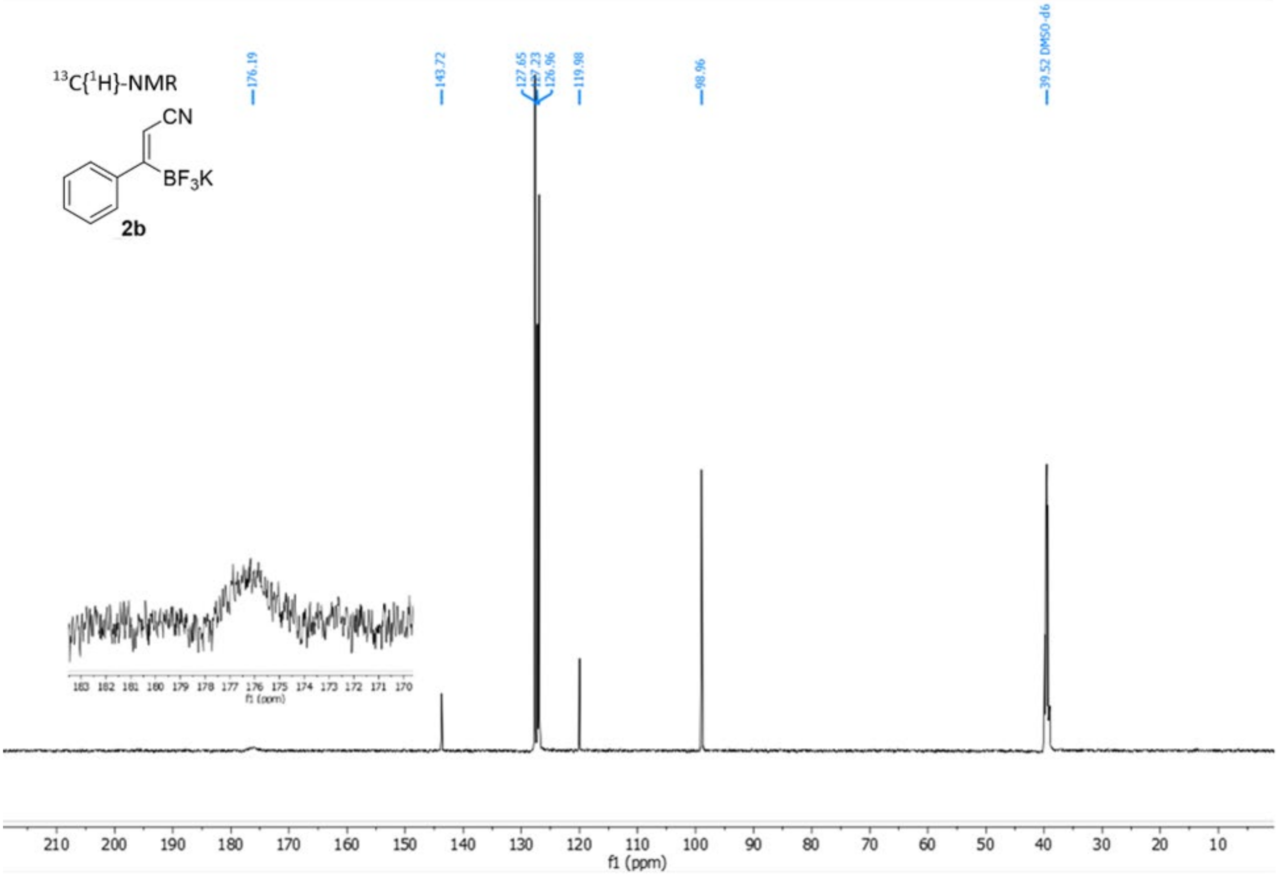

^{11}B-NMR (160 MHz, DMSO-d_6) of **2b**:

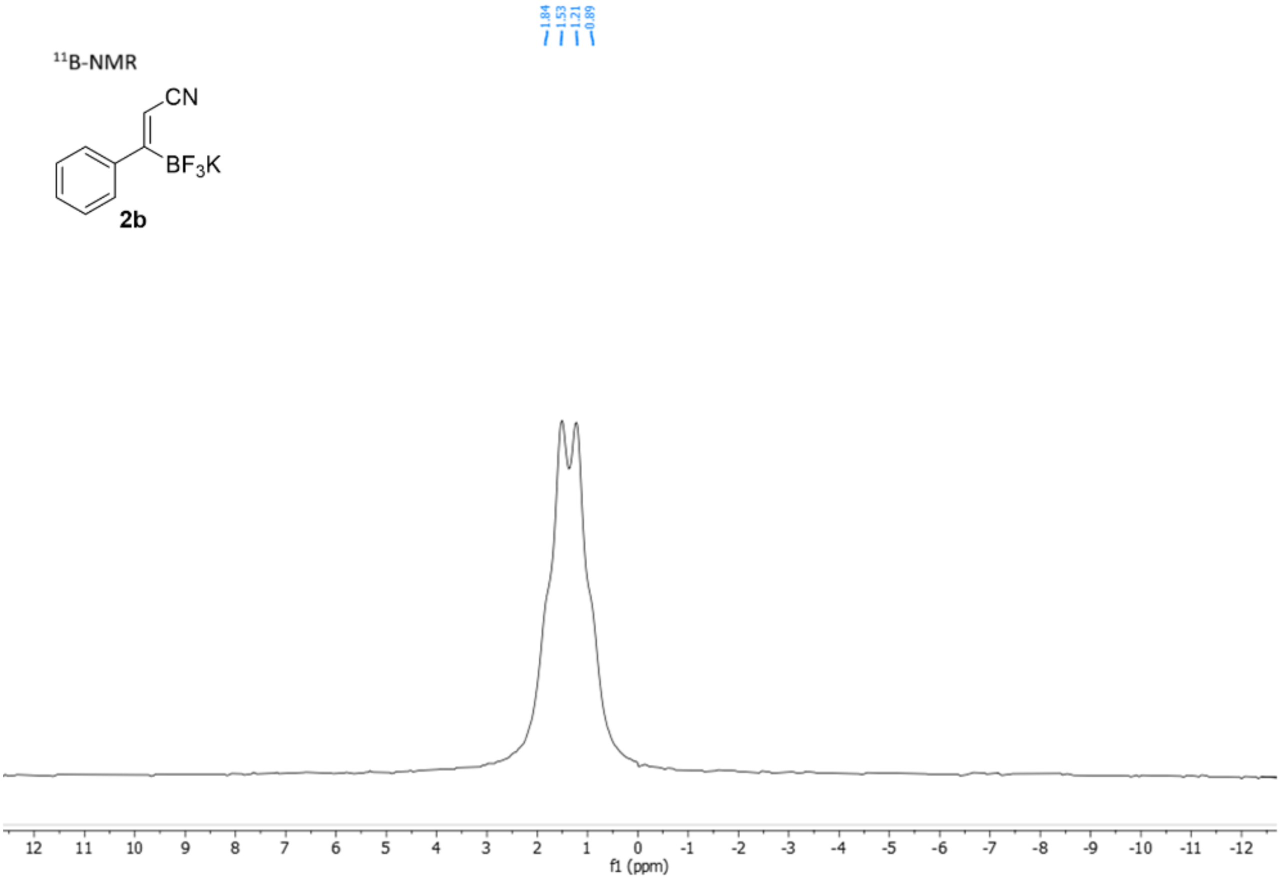

^{19}F-NMR (470 MHz, DMSO-d_6) of **2b**:

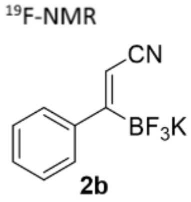

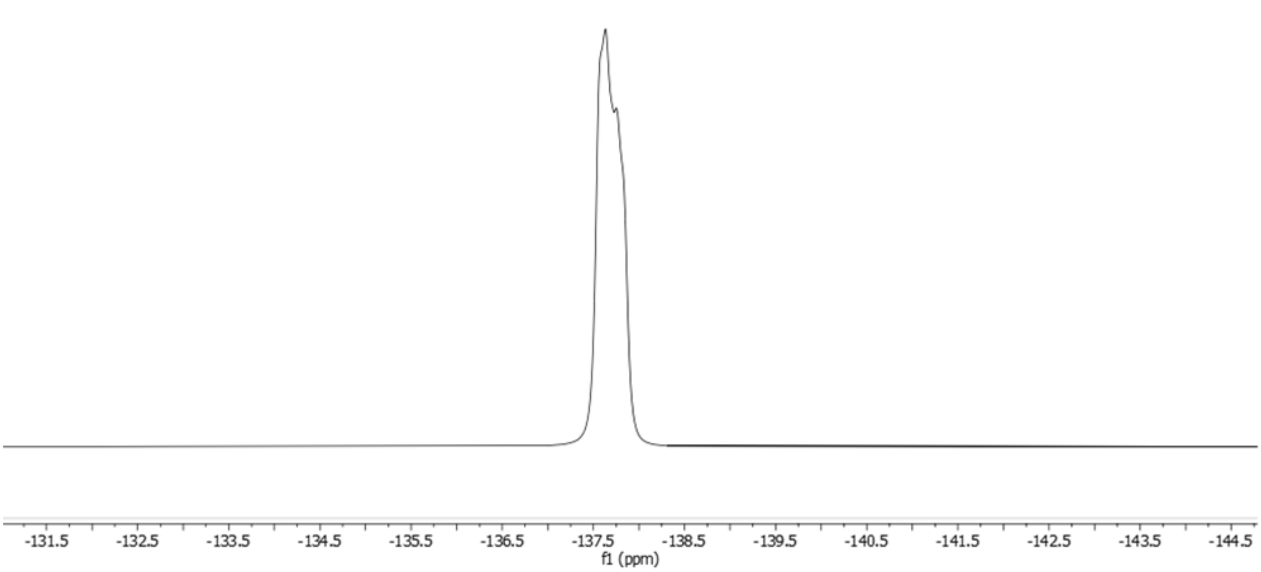

(*E*)-3-(p-tolyl)-3-(trifluoro-λ[4]-boraneyl)acrylonitrile, potassium salt (2c)

^{1}H-NMR (500 MHz, DMSO-d_6) of the reaction mixture of **2c**:

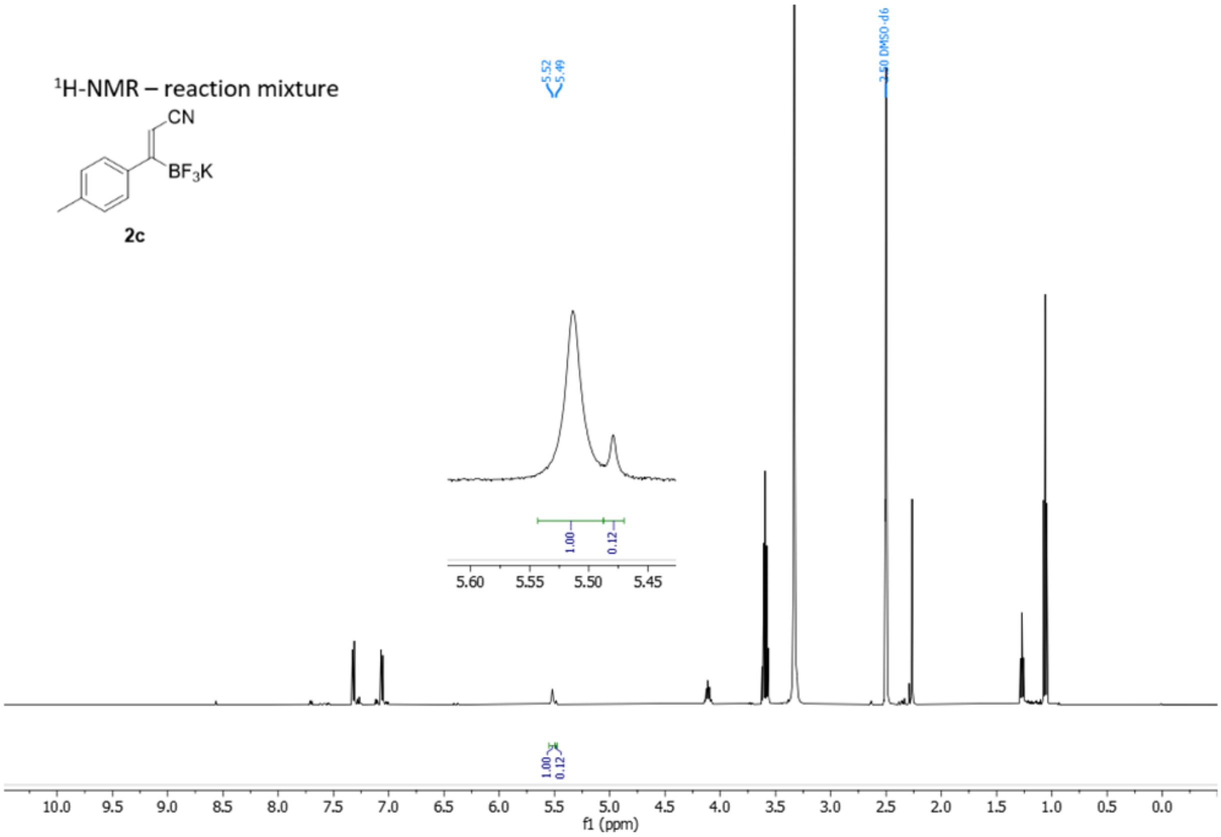

^{1}H-NMR (500 MHz, DMSO-d_6) of **2c**:

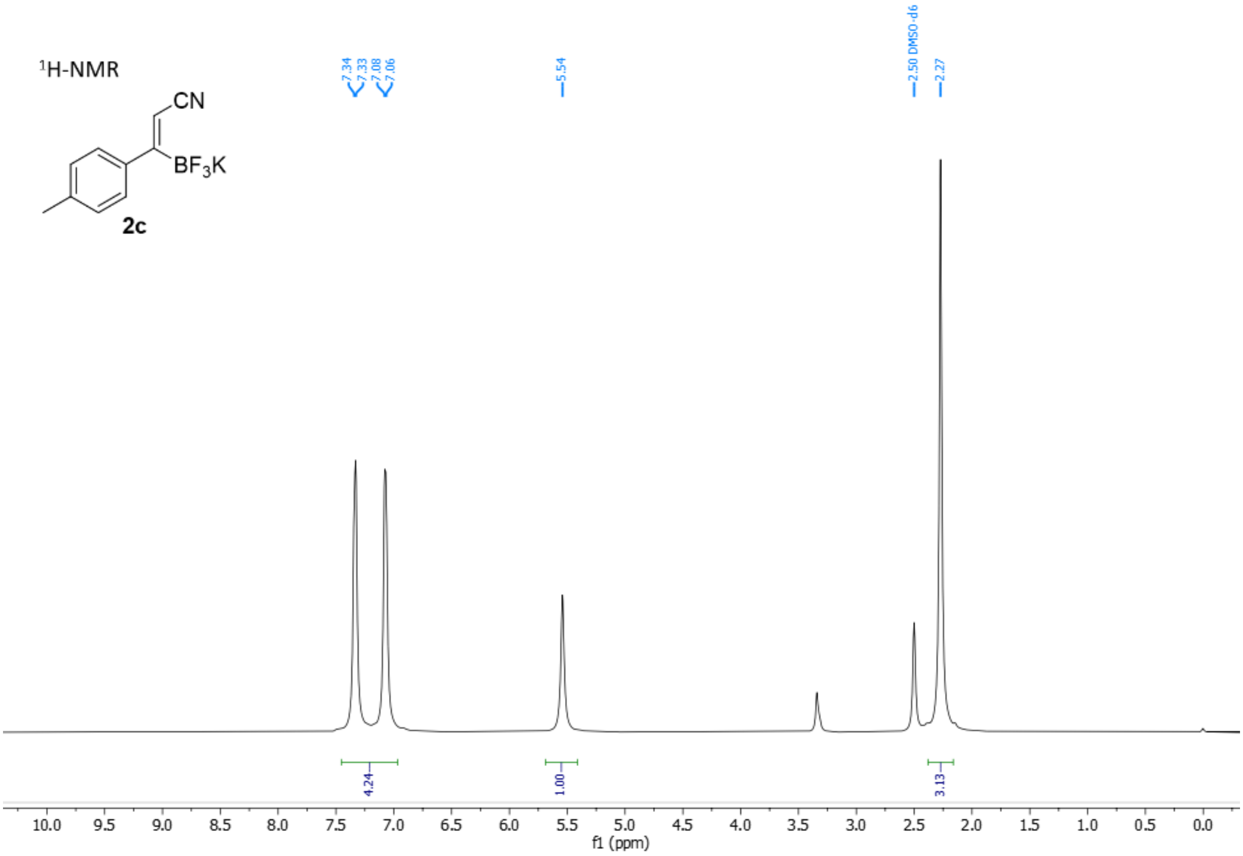

$^{13}C\{^1H\}$-NMR (126 MHz, DMSO-d_6) of **2c**:

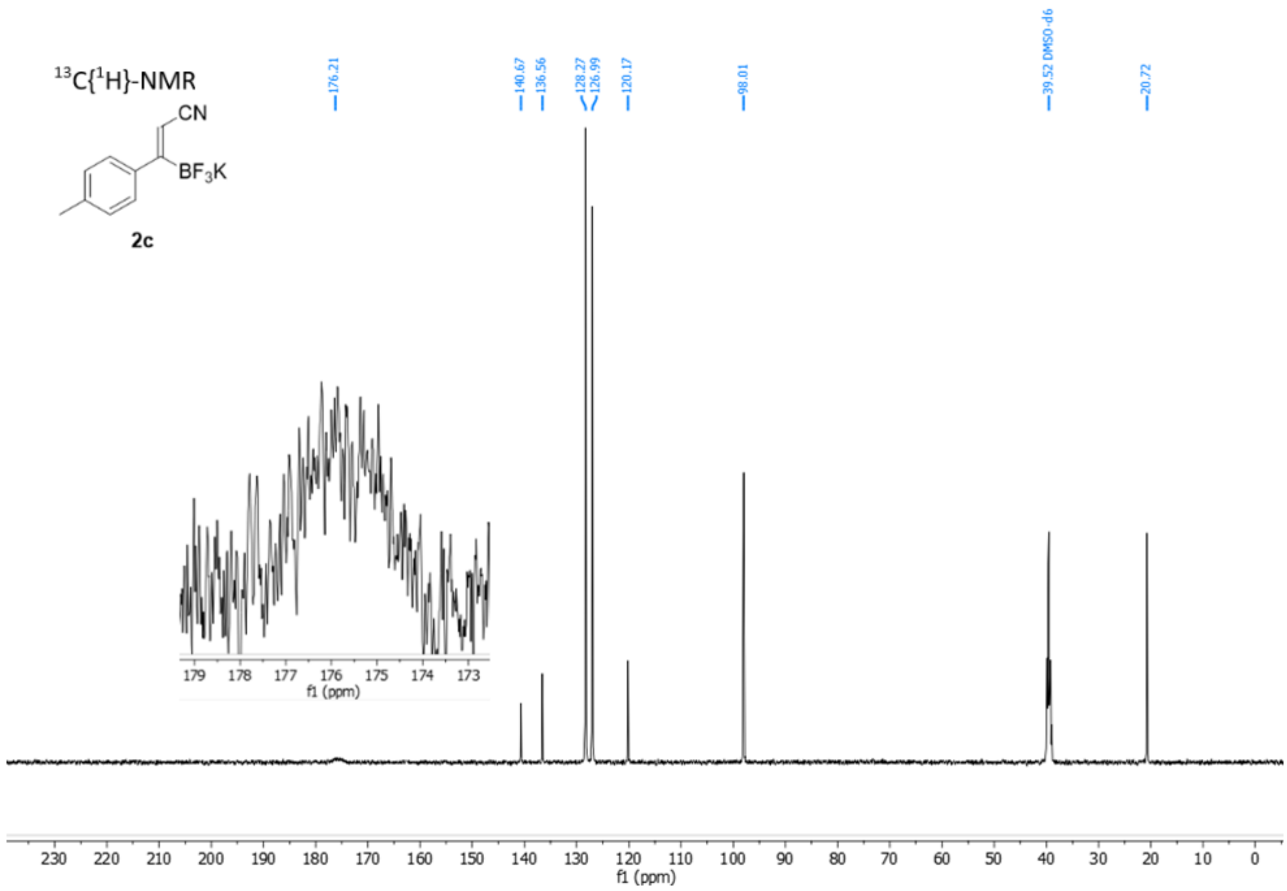

^{11}B-NMR (160 MHz, DMSO-d_6) of **2c**:

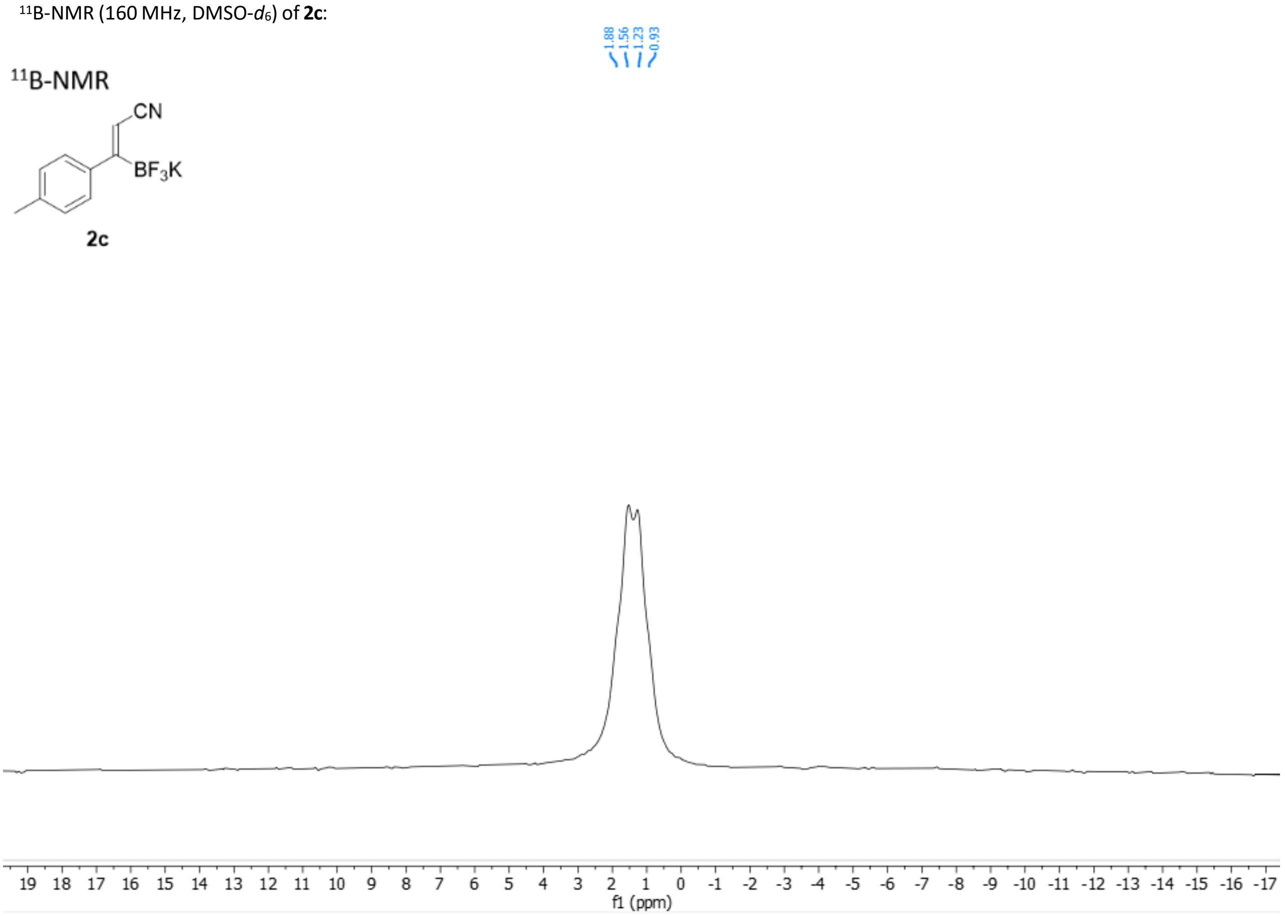

^{19}F-NMR (470 MHz, DMSO-d_6) of **2c**:

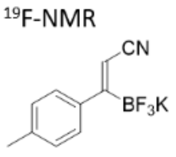

2c

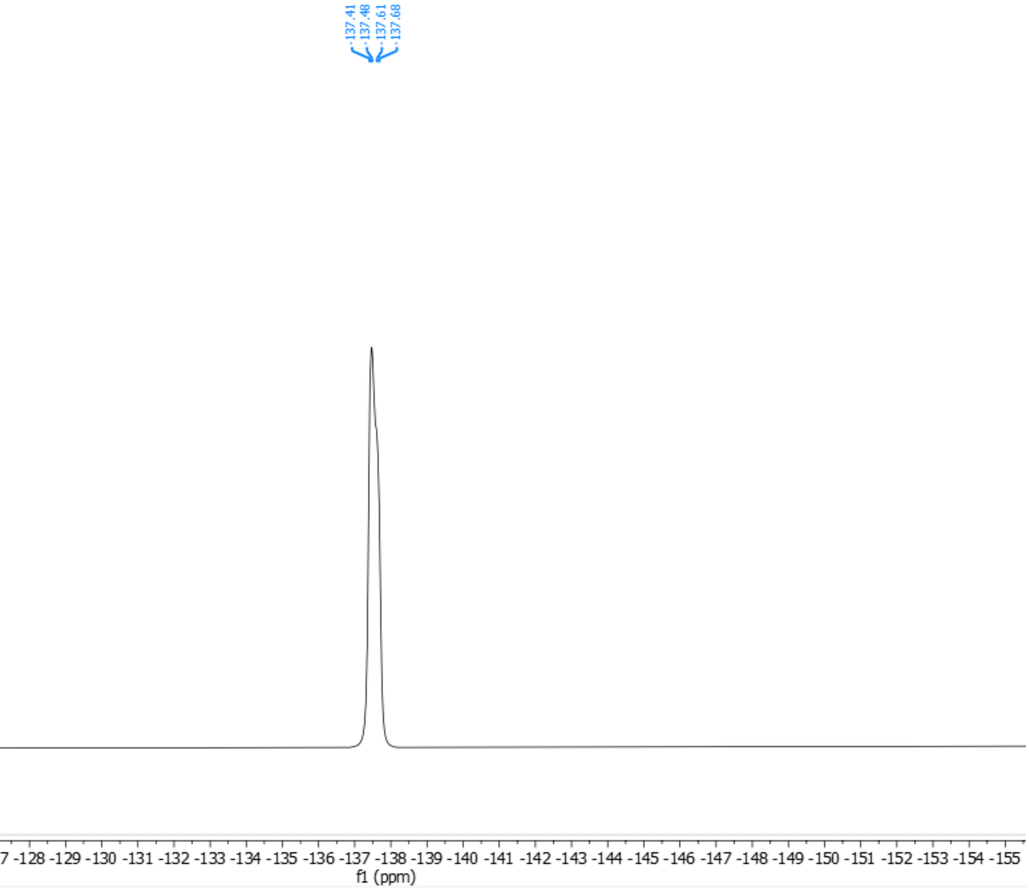

(*E*)-3-(4-fluorophenyl)-3-(trifluoro-λ^4-boraneyl)acrylonitrile, potassium salt (2d)

^{1}H-NMR (500 MHz, DMSO-d_6) of the reaction mixture of **2d**:

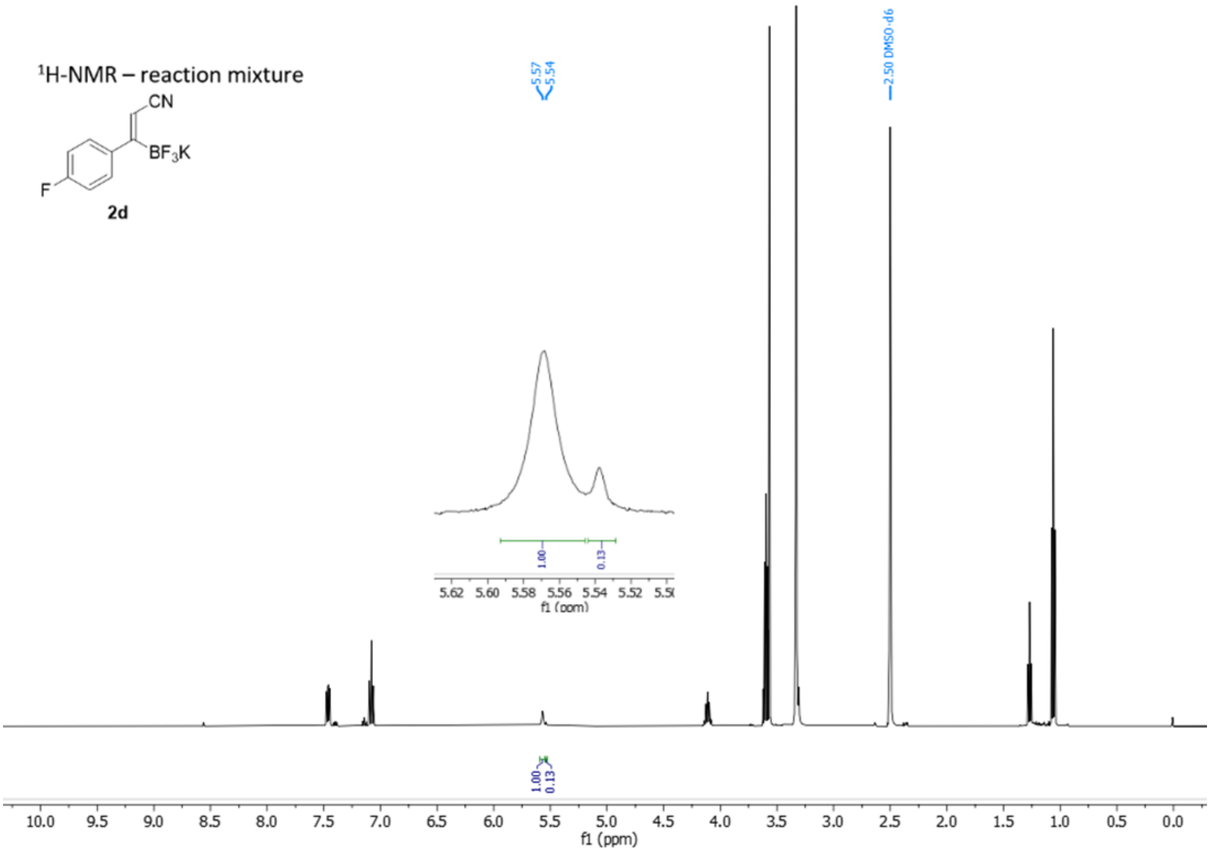

^{1}H-NMR (500 MHz, DMSO-d_6) of **2d**:

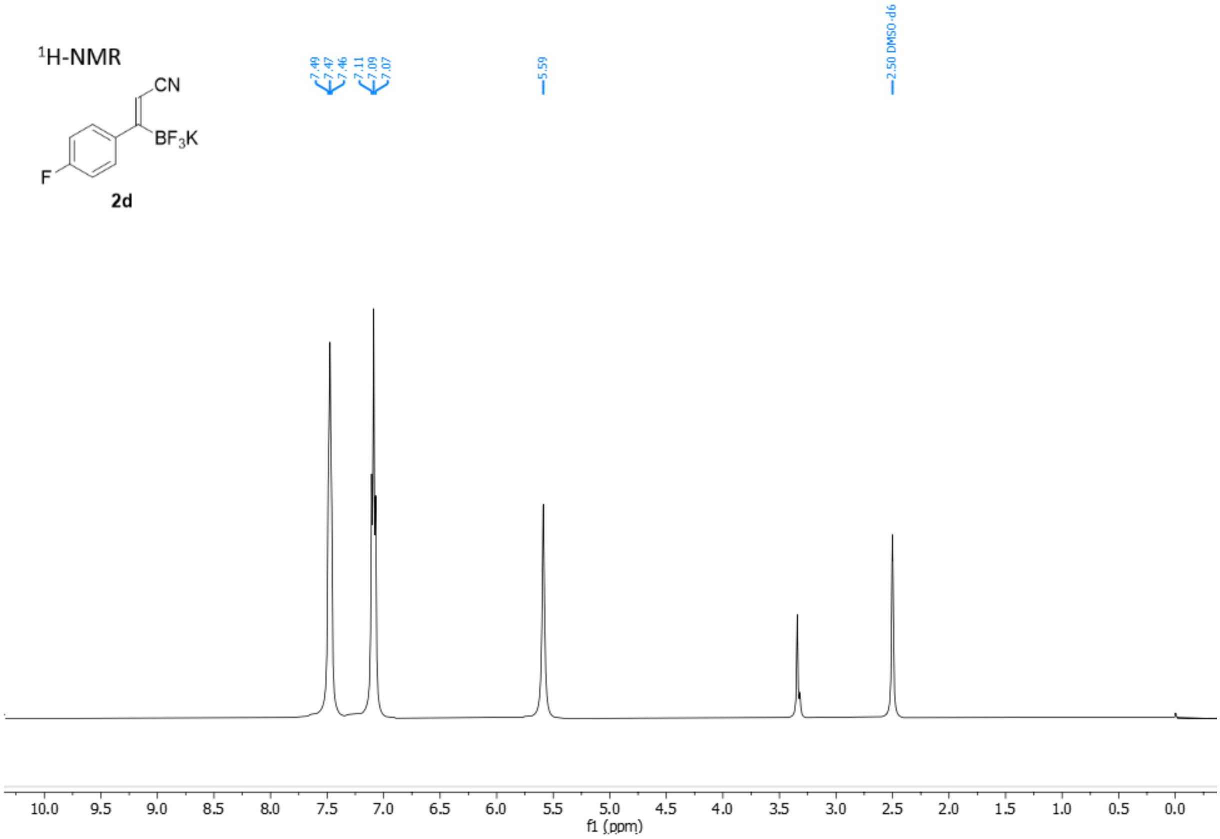

$^{13}C\{^{1}H\}$-NMR (126 MHz, DMSO-d_6) of **2d**:

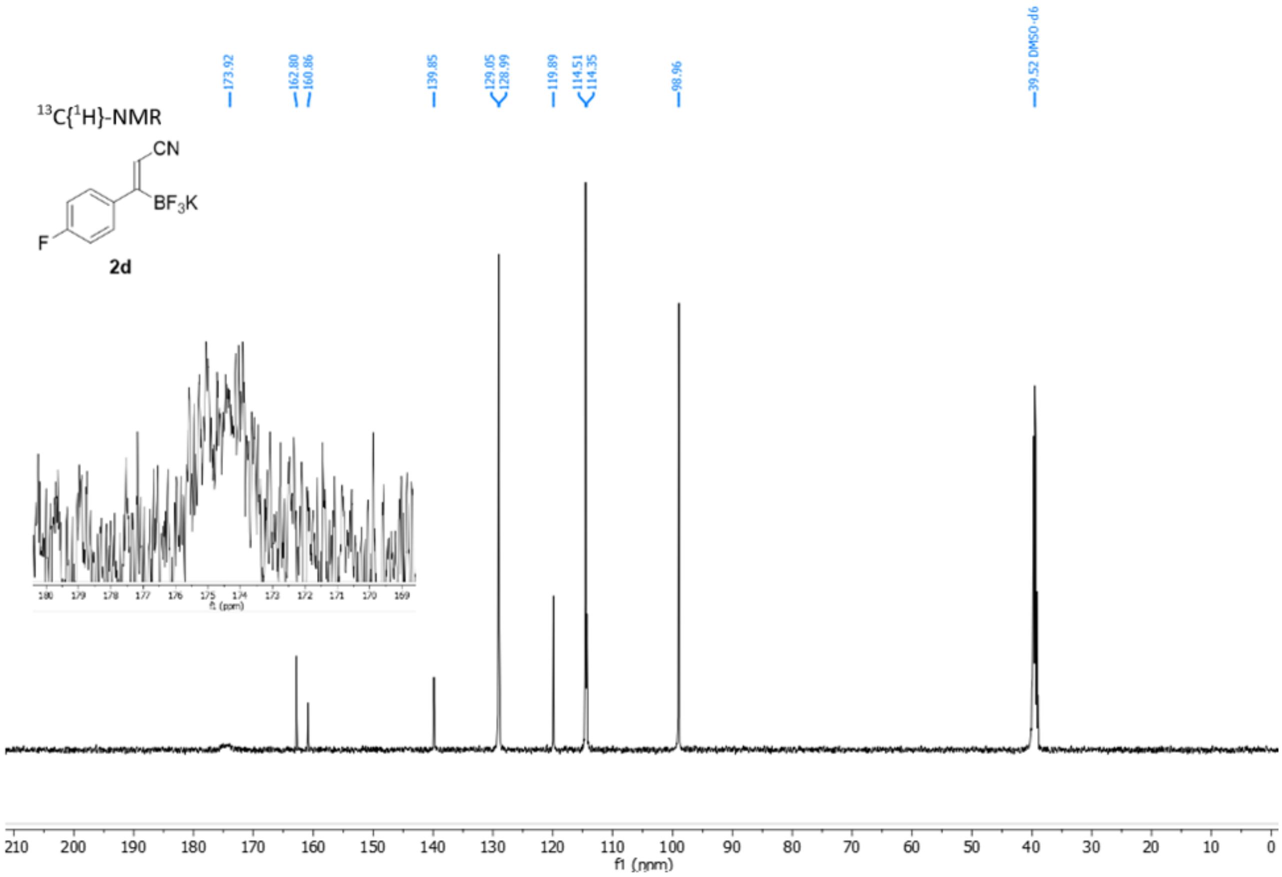

^{11}B-NMR (160 MHz, DMSO-d_6) of **2d**:

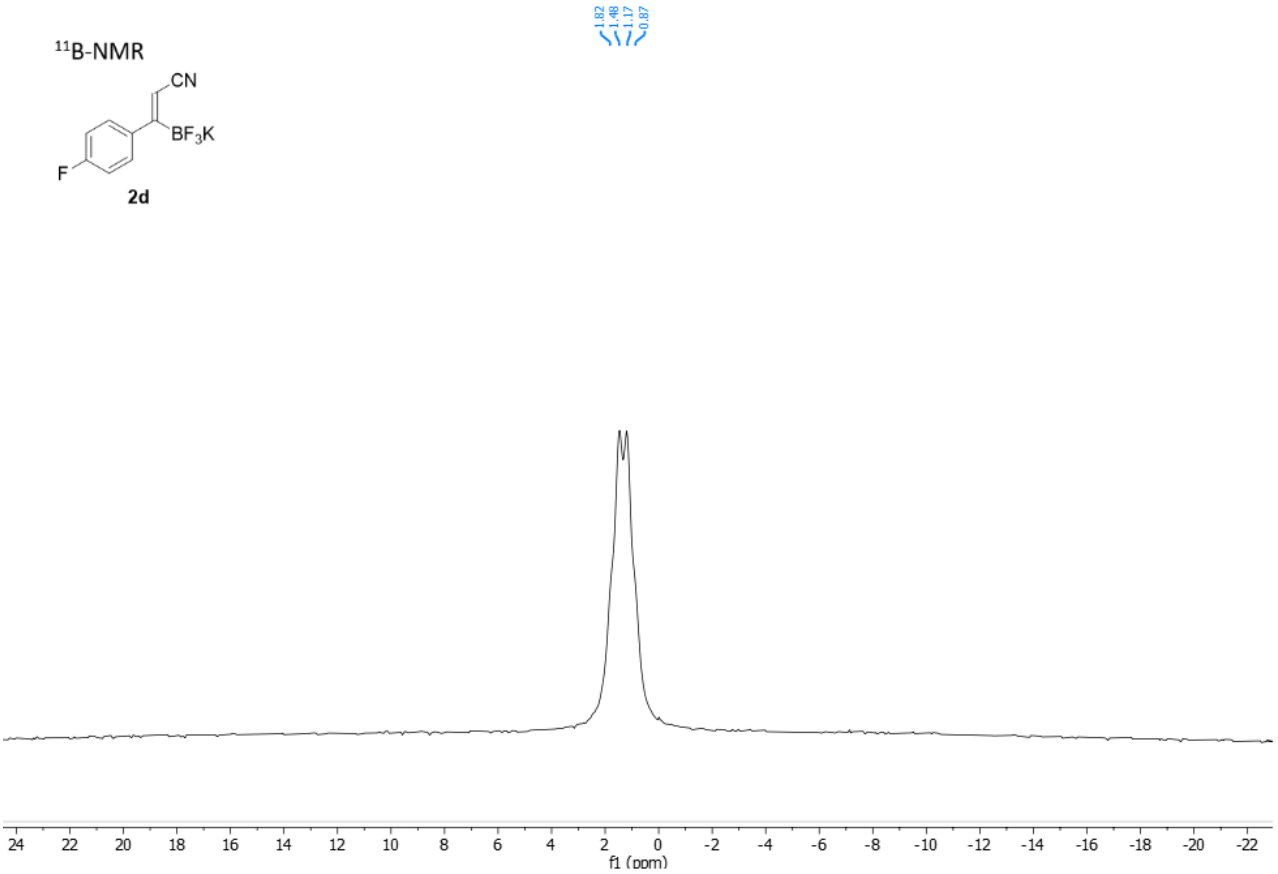

^{19}F-NMR (470 MHz, DMSO-d_6) of **2d**:

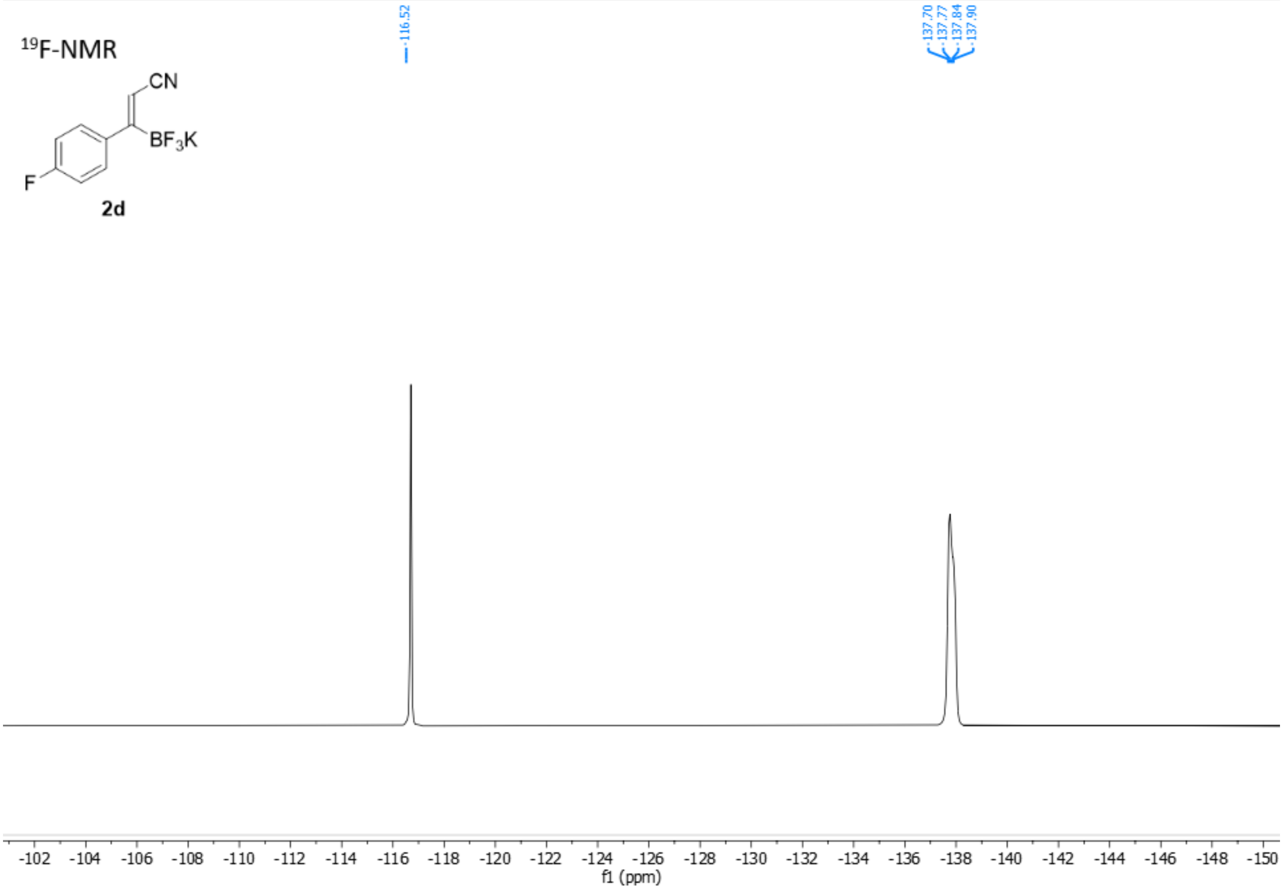

(E)-3-(4-methoxyphenyl)-3-(trifluoro-λ⁴-boraneyl)acrylonitrile, potassium salt (2e)

^{1}H-NMR (500 MHz, DMSO-d_6) of the reaction mixture of **2e**:

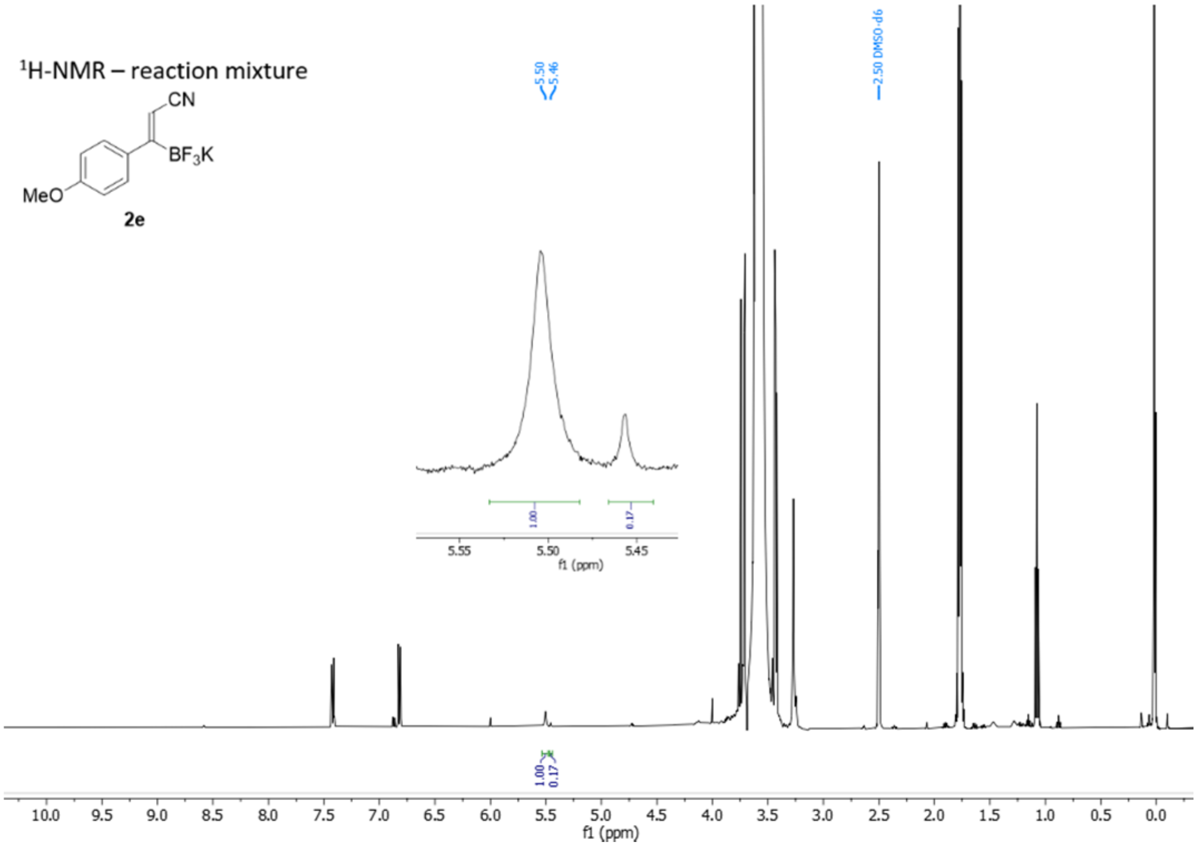

^{1}H-NMR (500 MHz, DMSO-d_6) of **2e**:

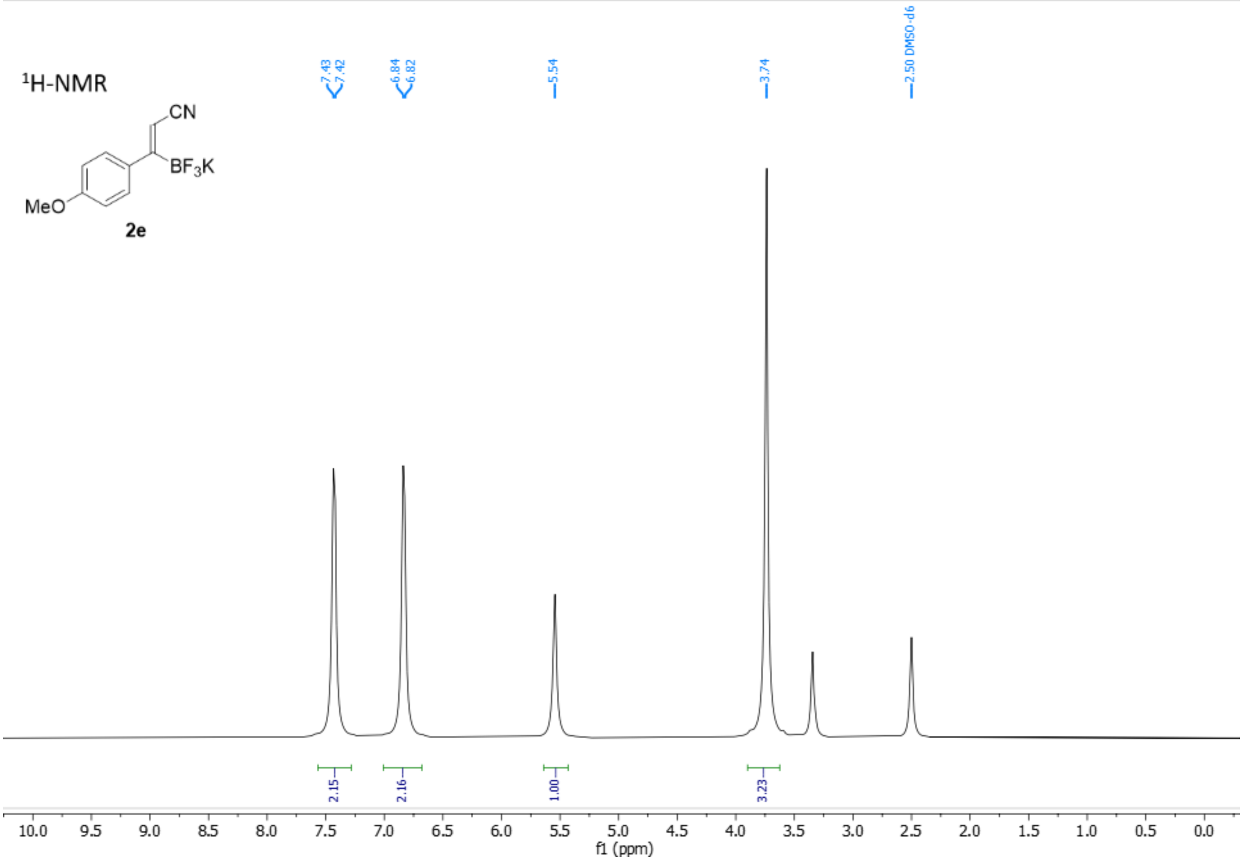

$^{13}C\{^1H\}$-NMR (126 MHz, DMSO-d_6) of **2e**:

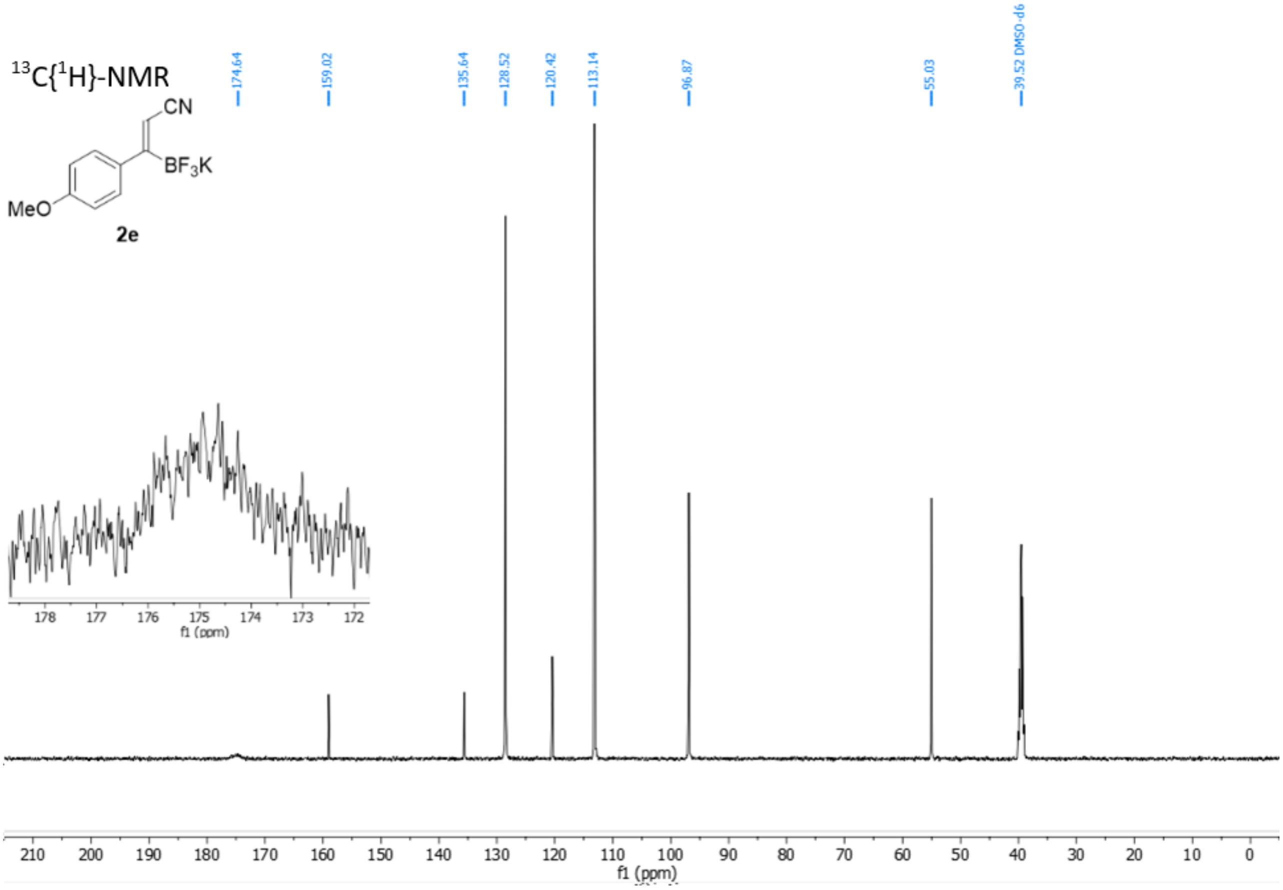

^{11}B-NMR (160 MHz, DMSO-d_6) of **2e**:

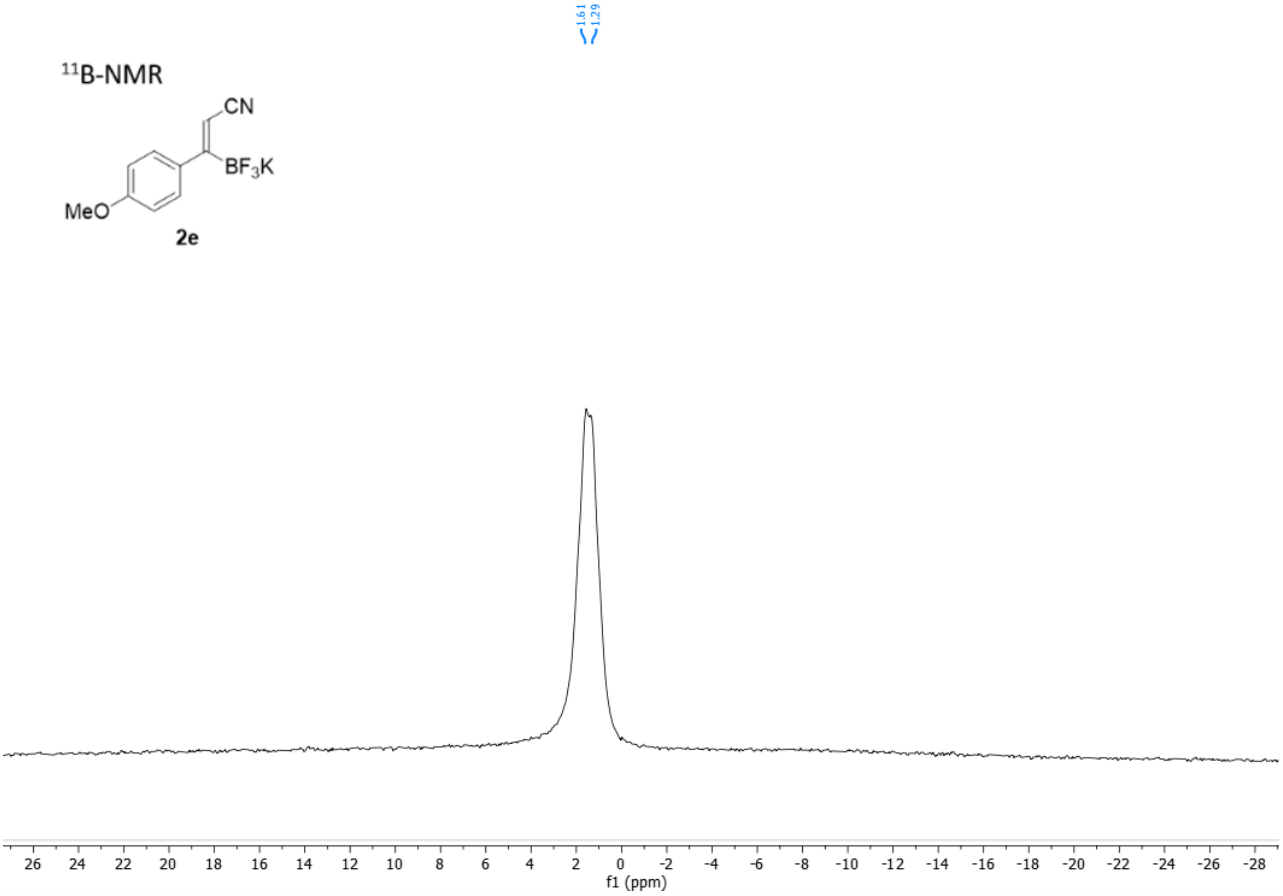

^{19}F-NMR (470 MHz, DMSO-d_6) of **2e**:

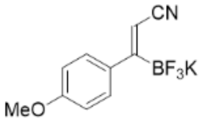

2e

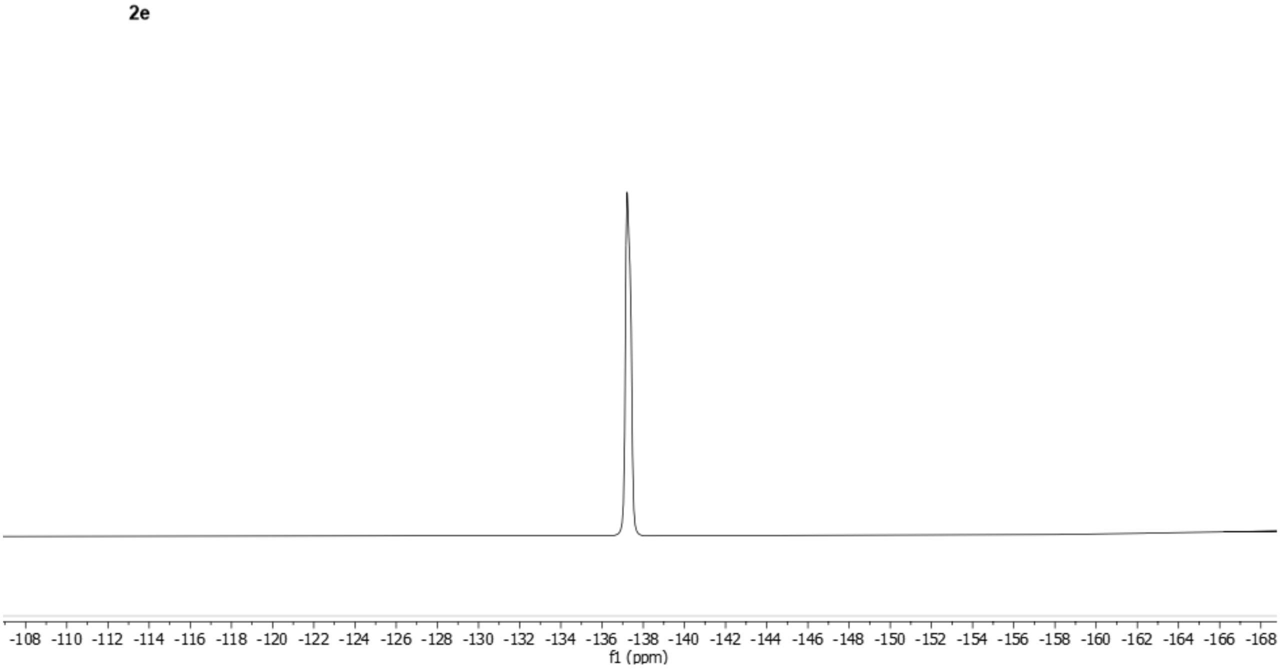

(E)-3-(trifluoro-λ^4-boraneyl)-3-(4-(trifluoromethyl)phenyl)acrylonitrile, potassium salt (2f)

^{1}H-NMR (500 MHz, DMSO-d_6) of the reaction mixture of **2f**:

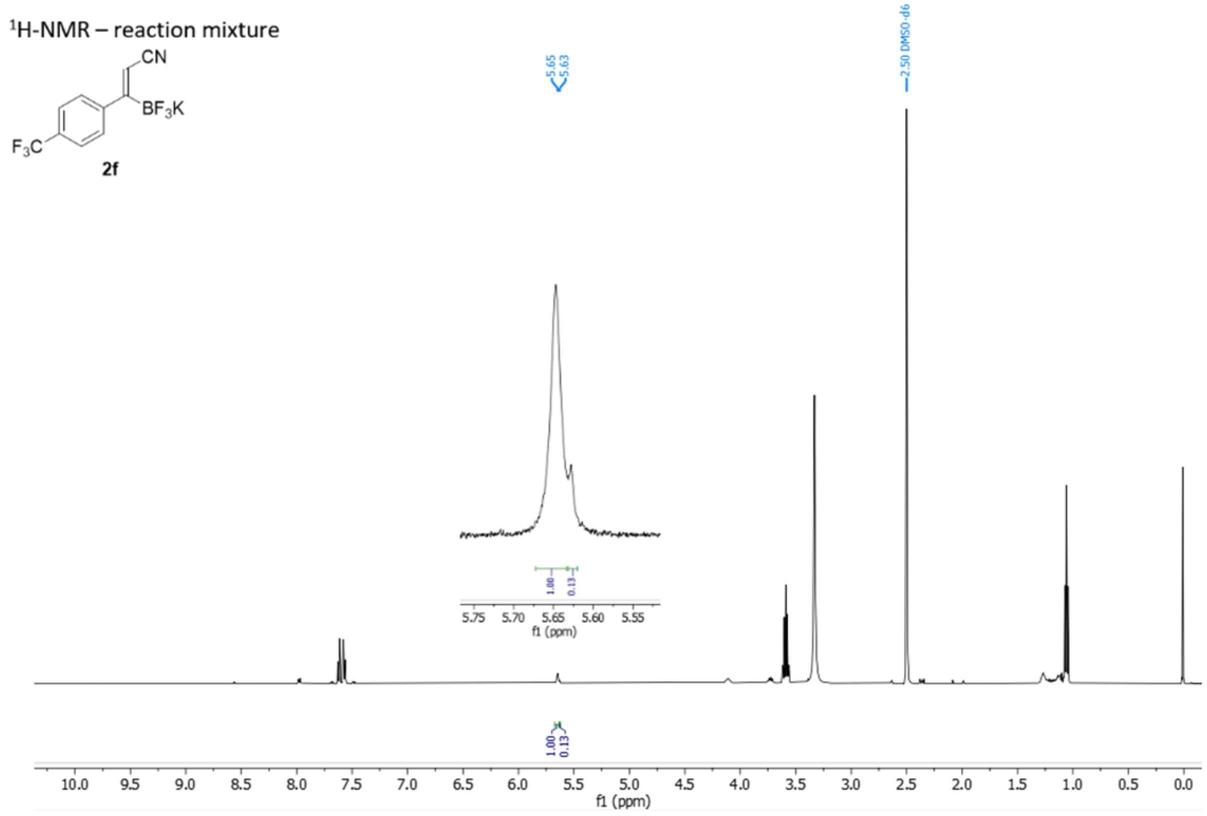

^{1}H-NMR (500 MHz, DMSO-d_6) of **2f**:

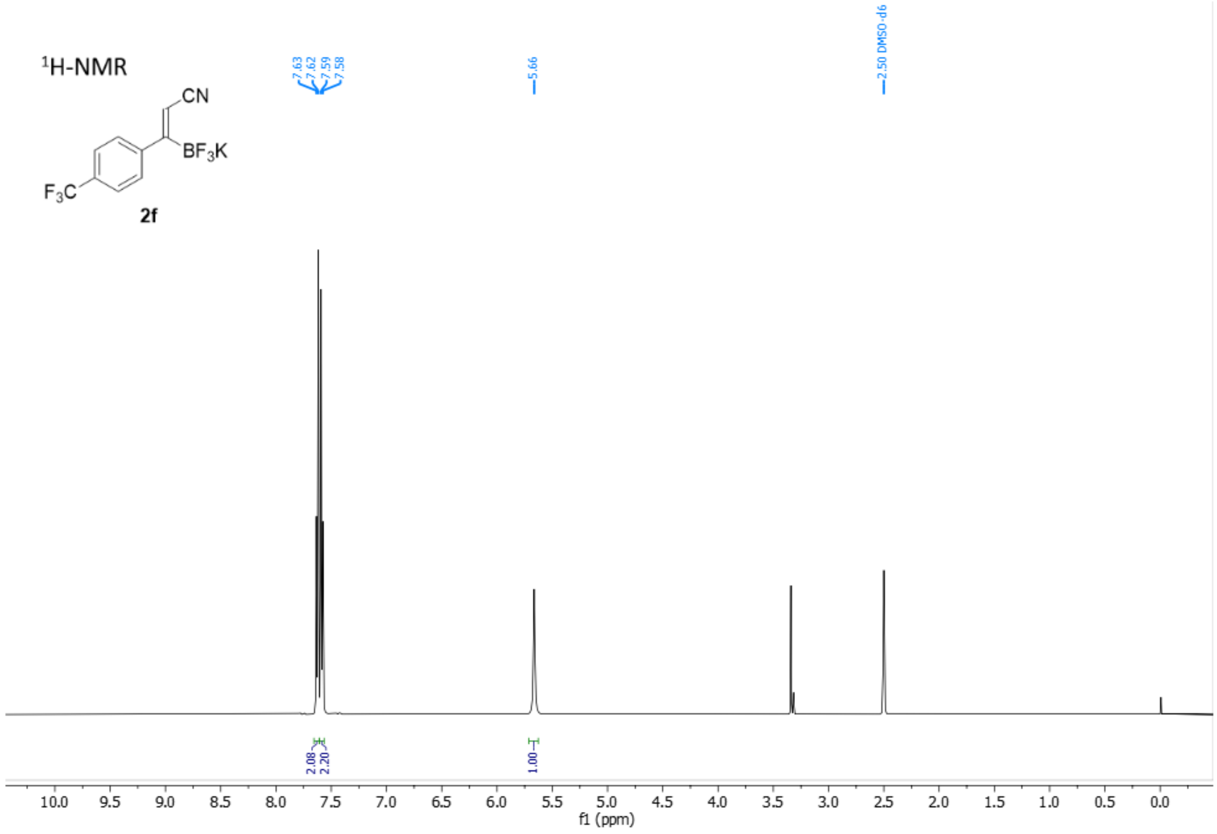

$^{13}C\{^1H\}$-NMR (126 MHz, DMSO-d_6) of **2f**:

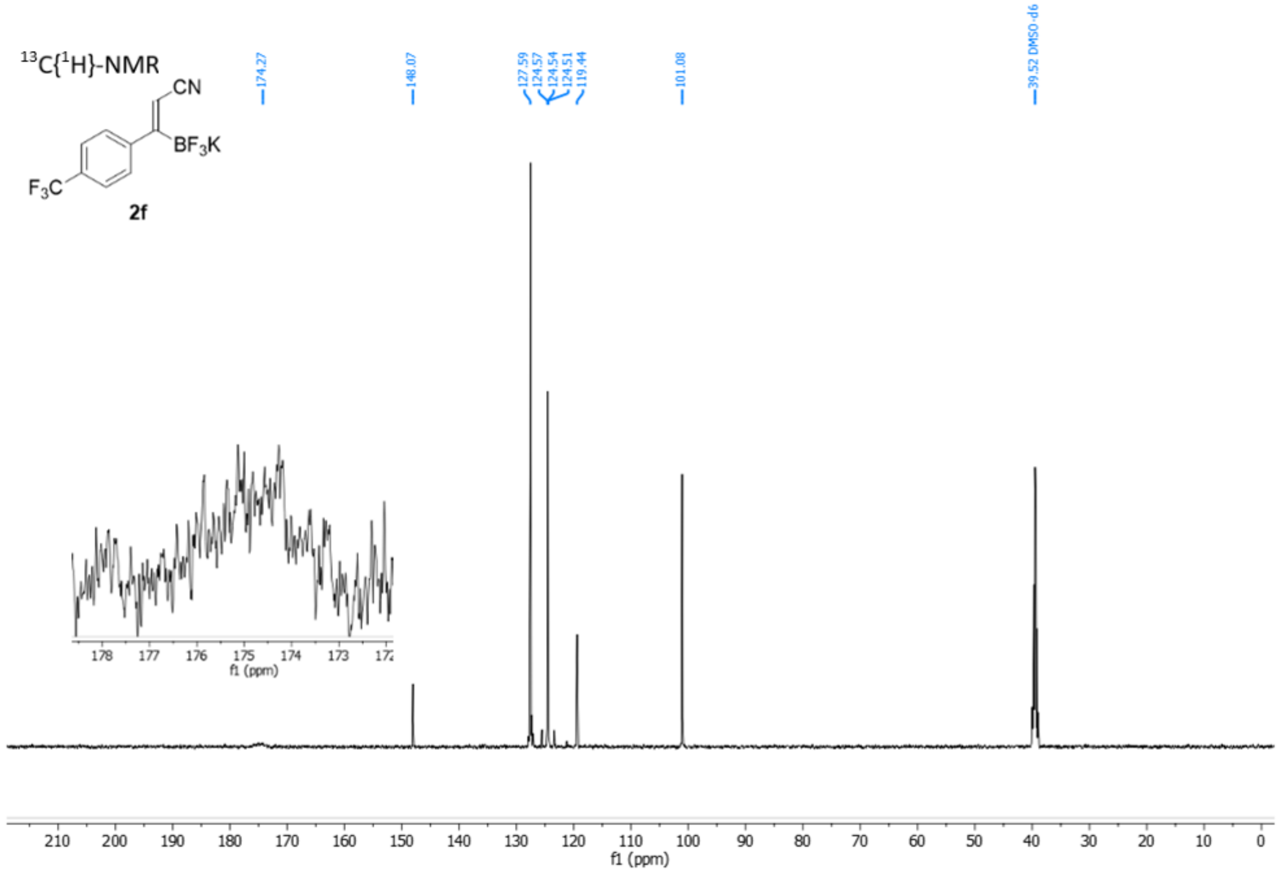

^{11}B-NMR (160 MHz, DMSO-d_6) of **2f**:

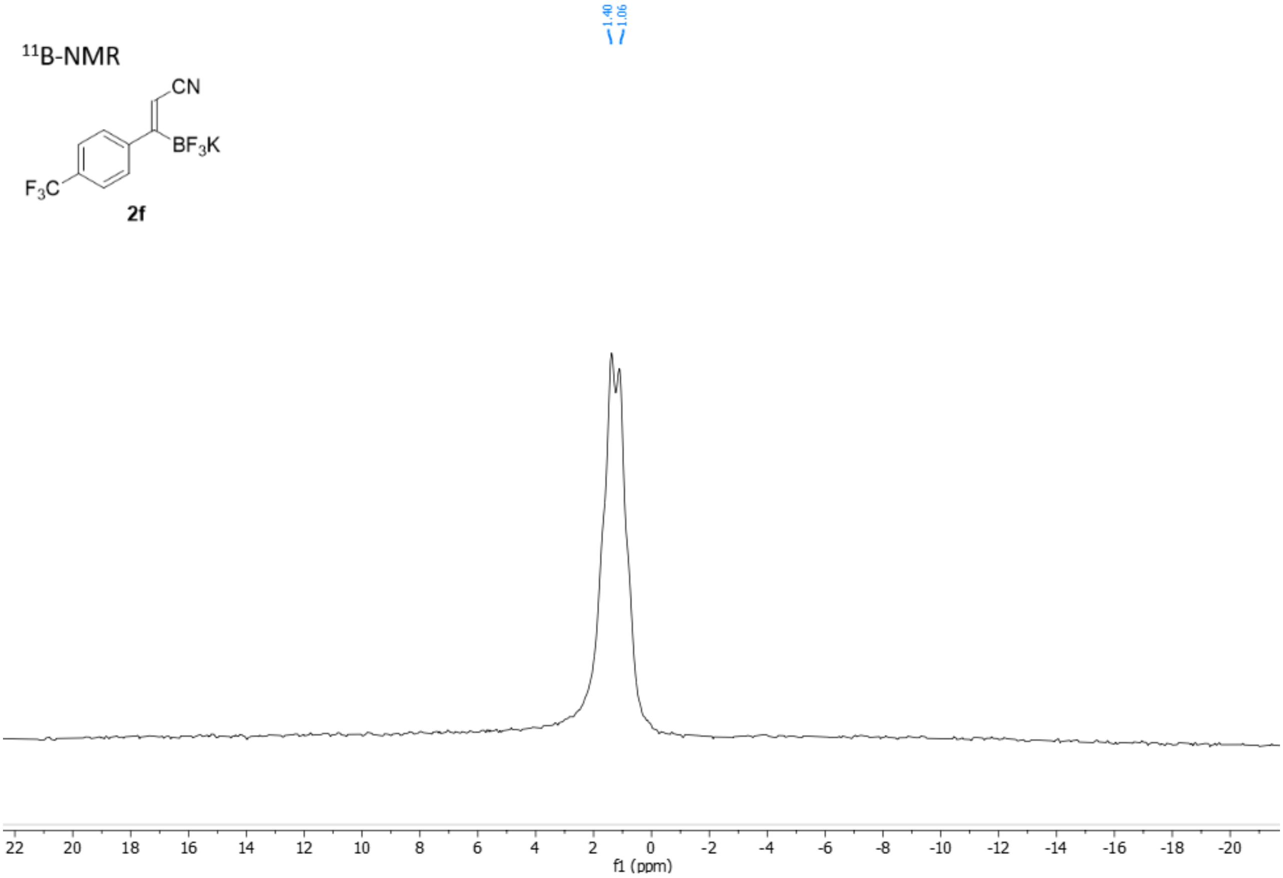

^{19}F-NMR (470 MHz, DMSO-d_6) of **2f**:

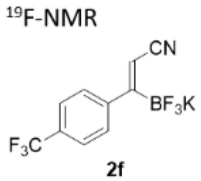

2f

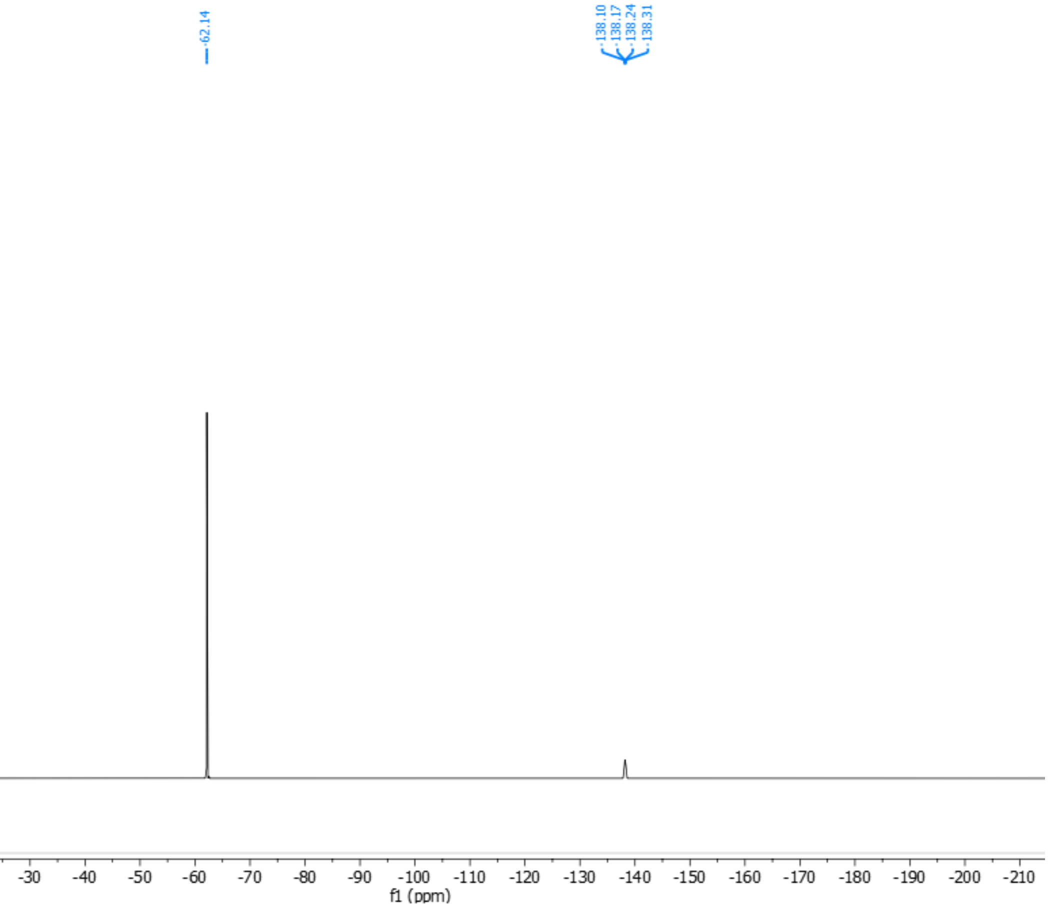

(*E*)-4-(2-cyano-1-(trifluoro-λ⁴-boraneyl)vinyl)benzonitrile, potassium salt (2g)

^{1}H-NMR (500 MHz, DMSO-d_6) of the reaction mixture of **2g**:

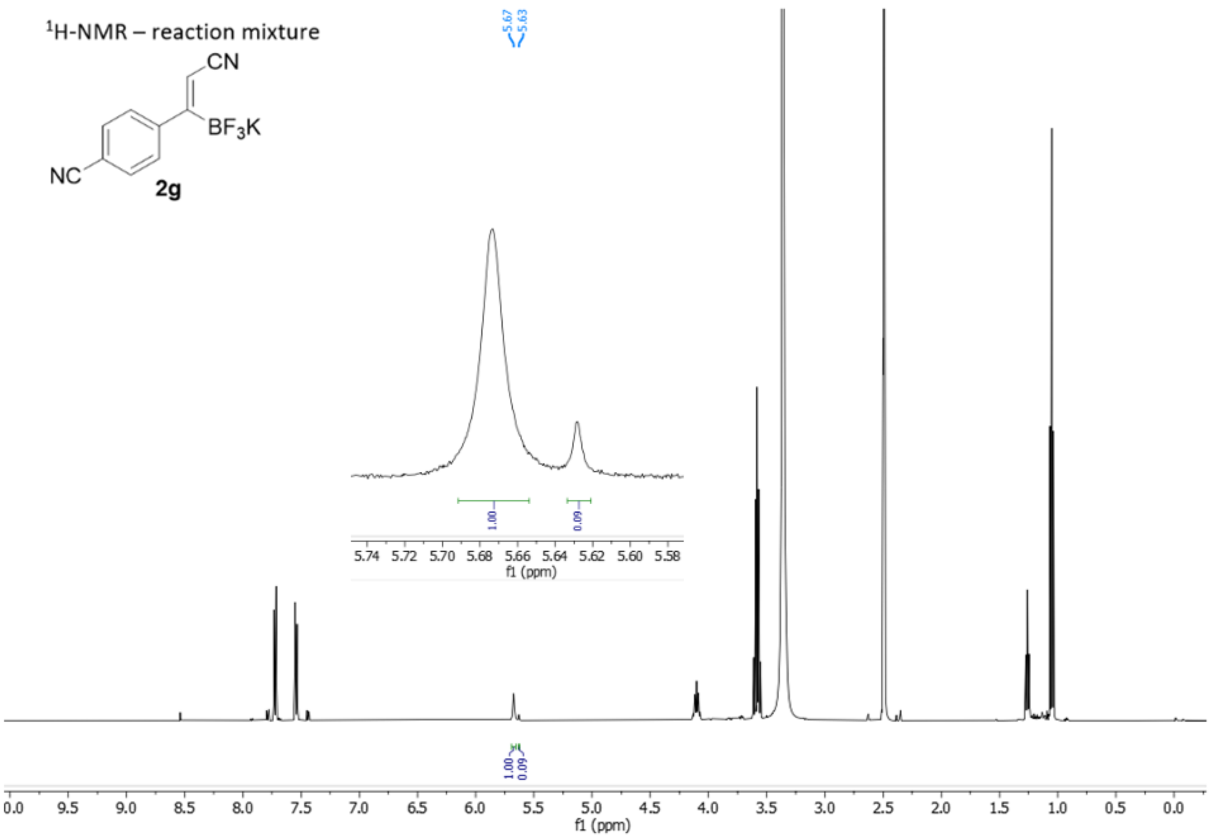

^{1}H-NMR (500 MHz, DMSO-d_6) of **2g**:

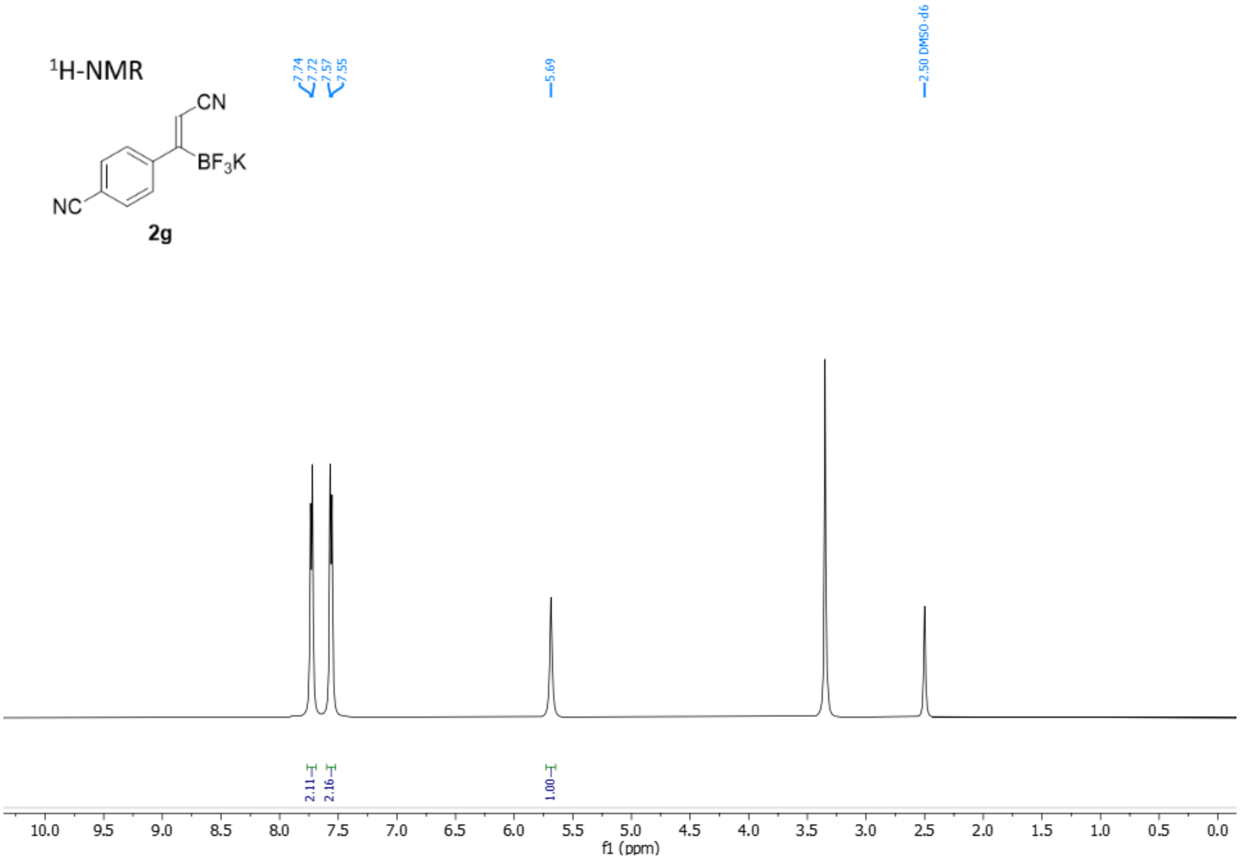

$^{13}C\{^1H\}$-NMR (126 MHz, DMSO-d_6) of **2g**:

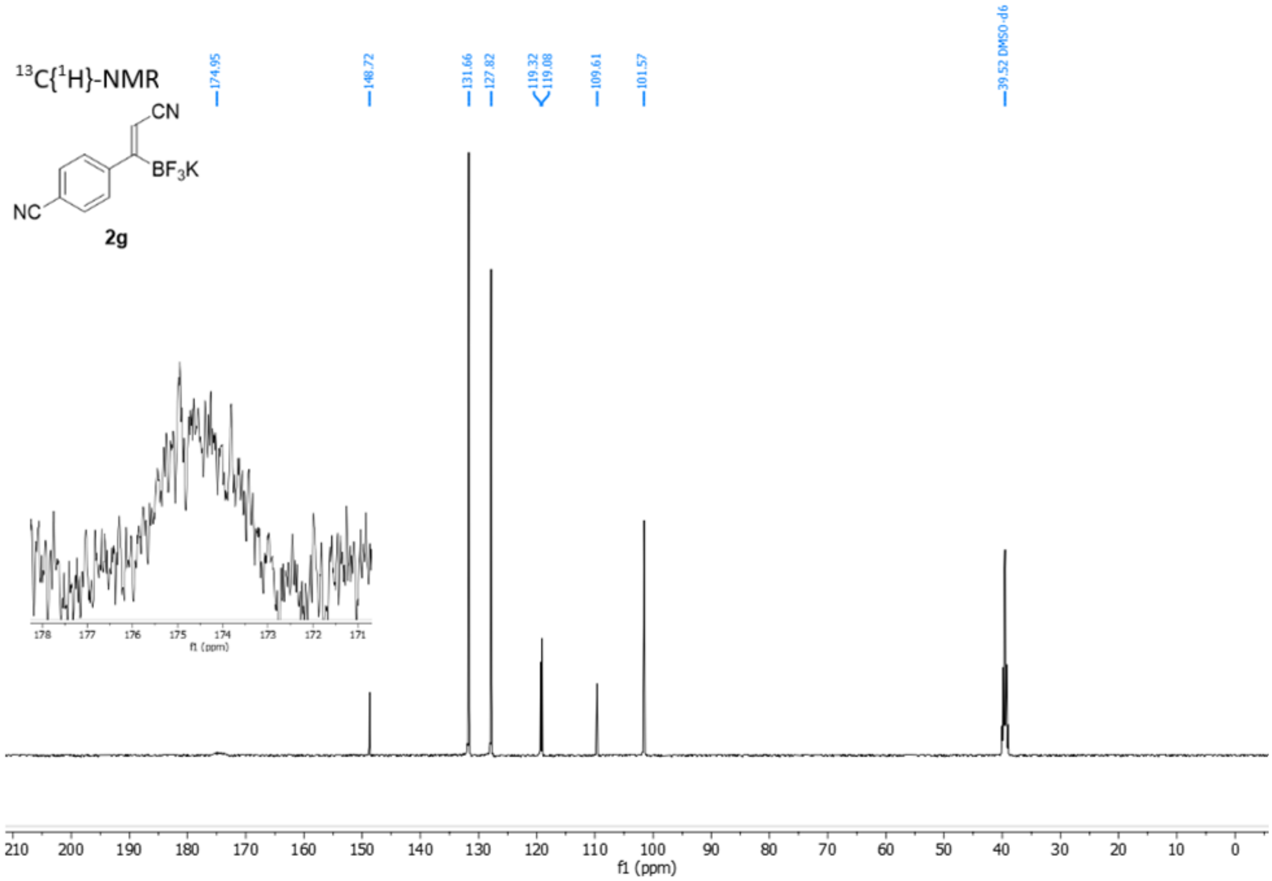

^{11}B-NMR (160 MHz, DMSO-d_6) of **2g**:

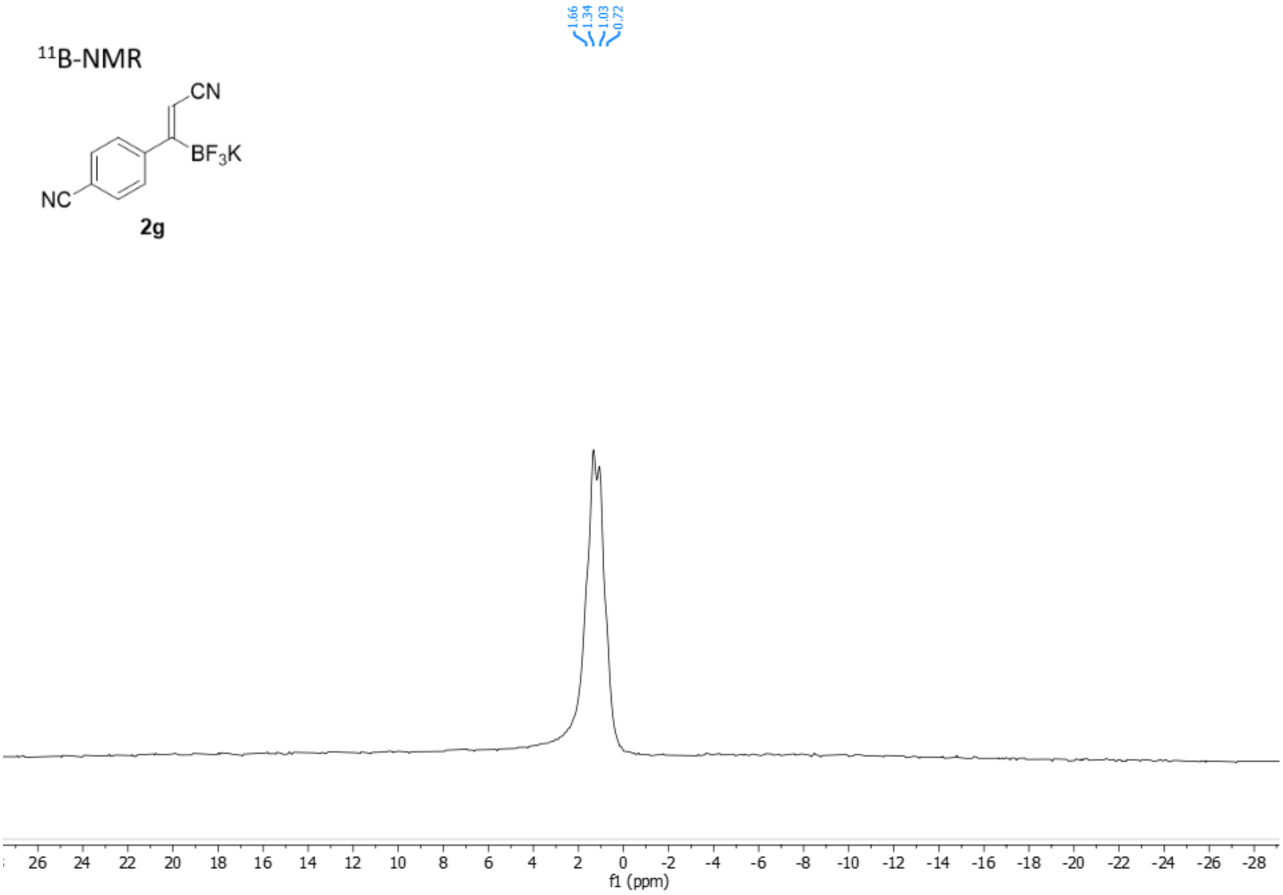

^{19}F-NMR (470 MHz, DMSO-d_6) of **2g**:

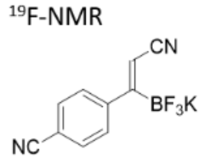

2g

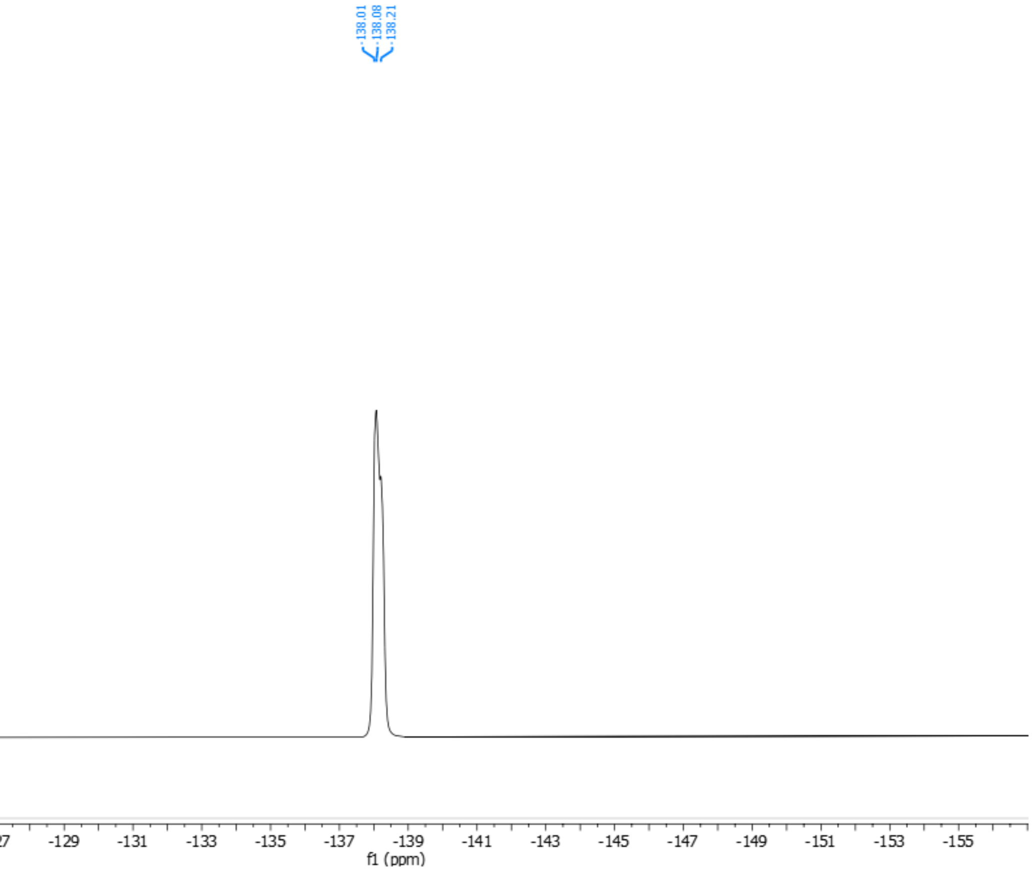

Ethyl (*E*)-4-(2-cyano-1-(trifluoro-λ^4-boraneyl)vinyl)benzoate, potassium salt (2h)

^{1}H-NMR (500 MHz, DMSO-d_6) of the reaction mixture of **2h**:

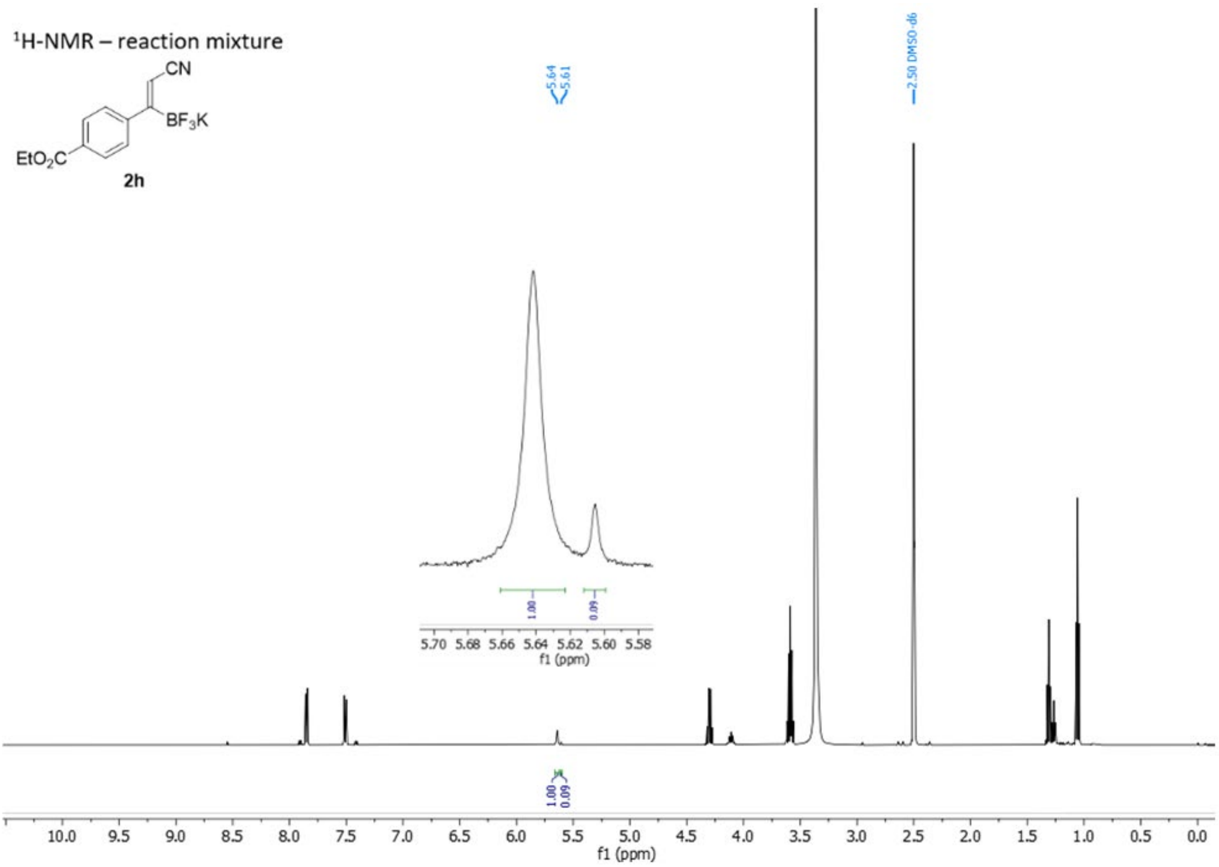

^{1}H-NMR (500 MHz, DMSO-d_6) of **2h**:

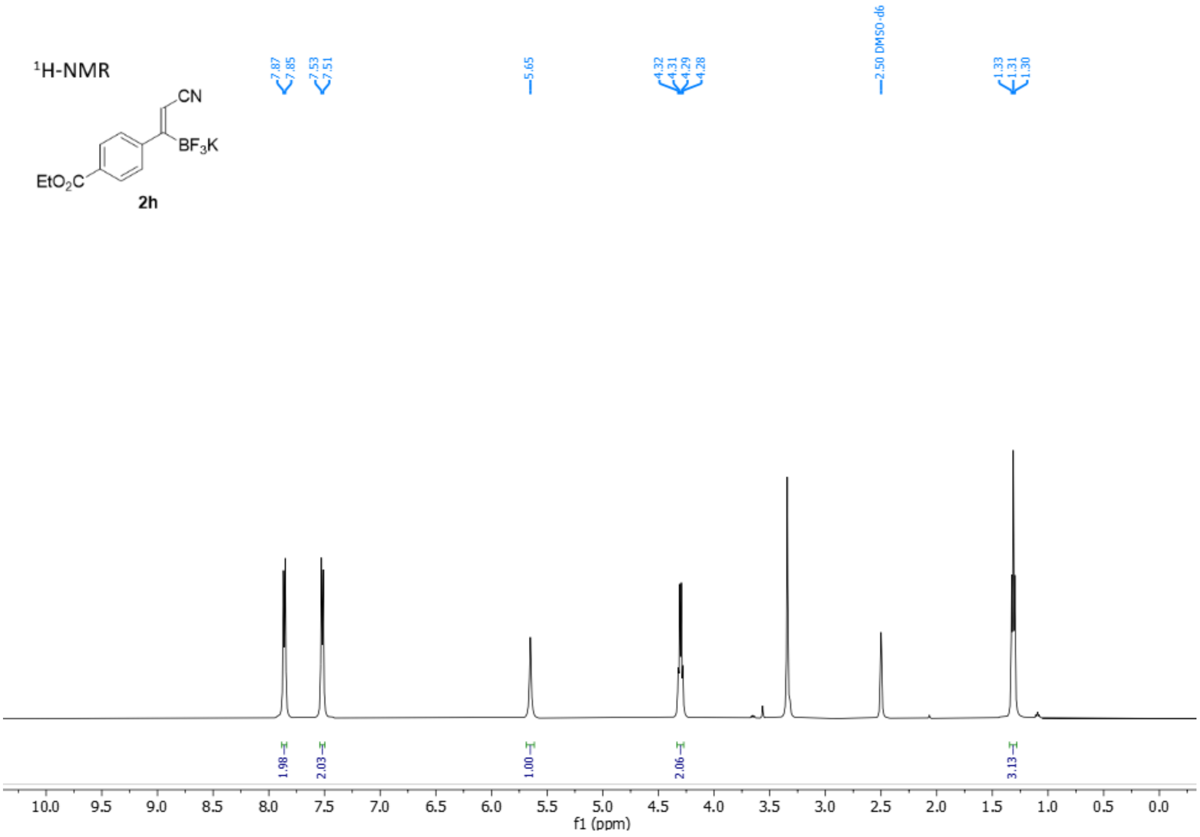

$^{13}C\{^1H\}$-NMR (126 MHz, DMSO-d_6) of **2h**:

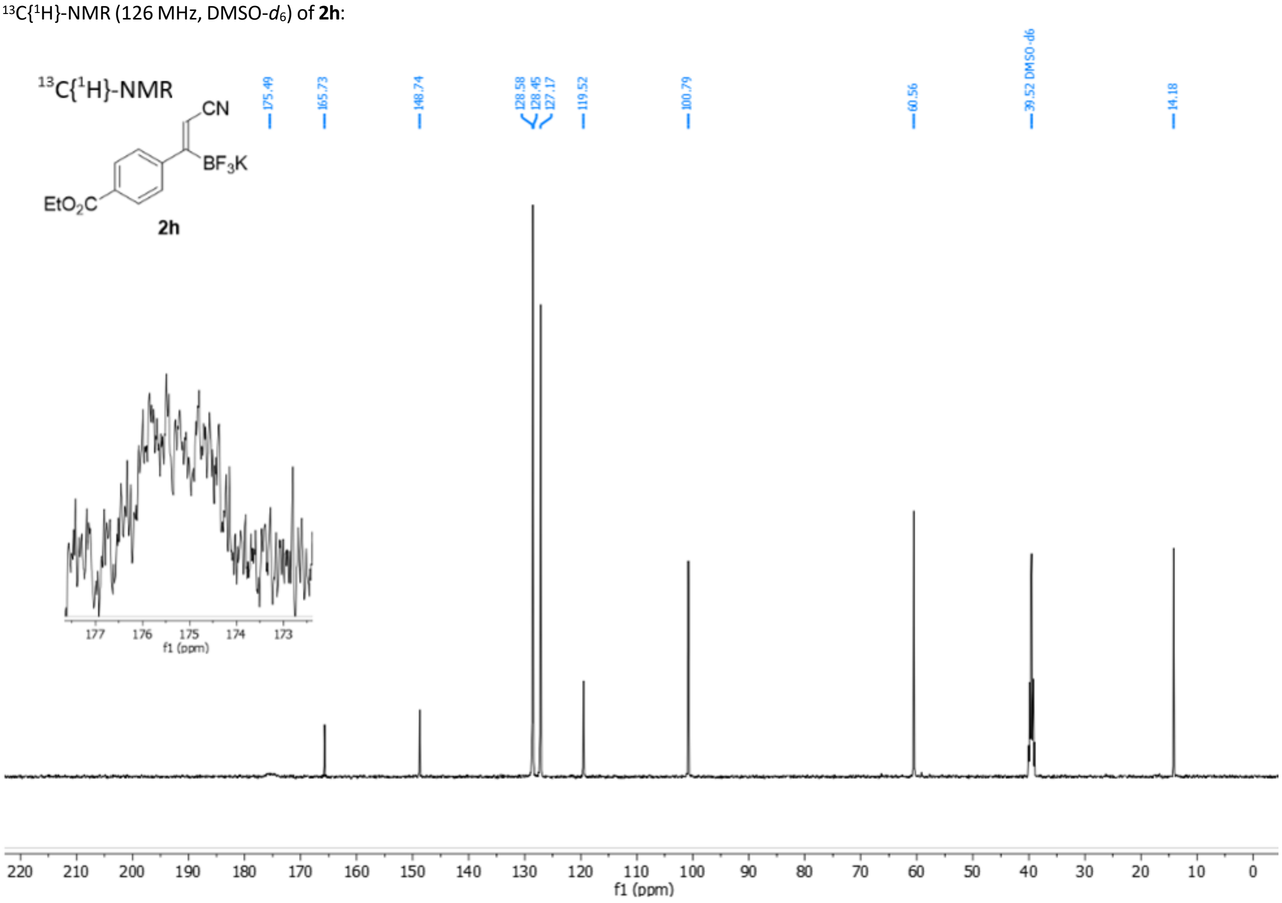

^{11}B-NMR (160 MHz, DMSO-d_6) of **2h**:

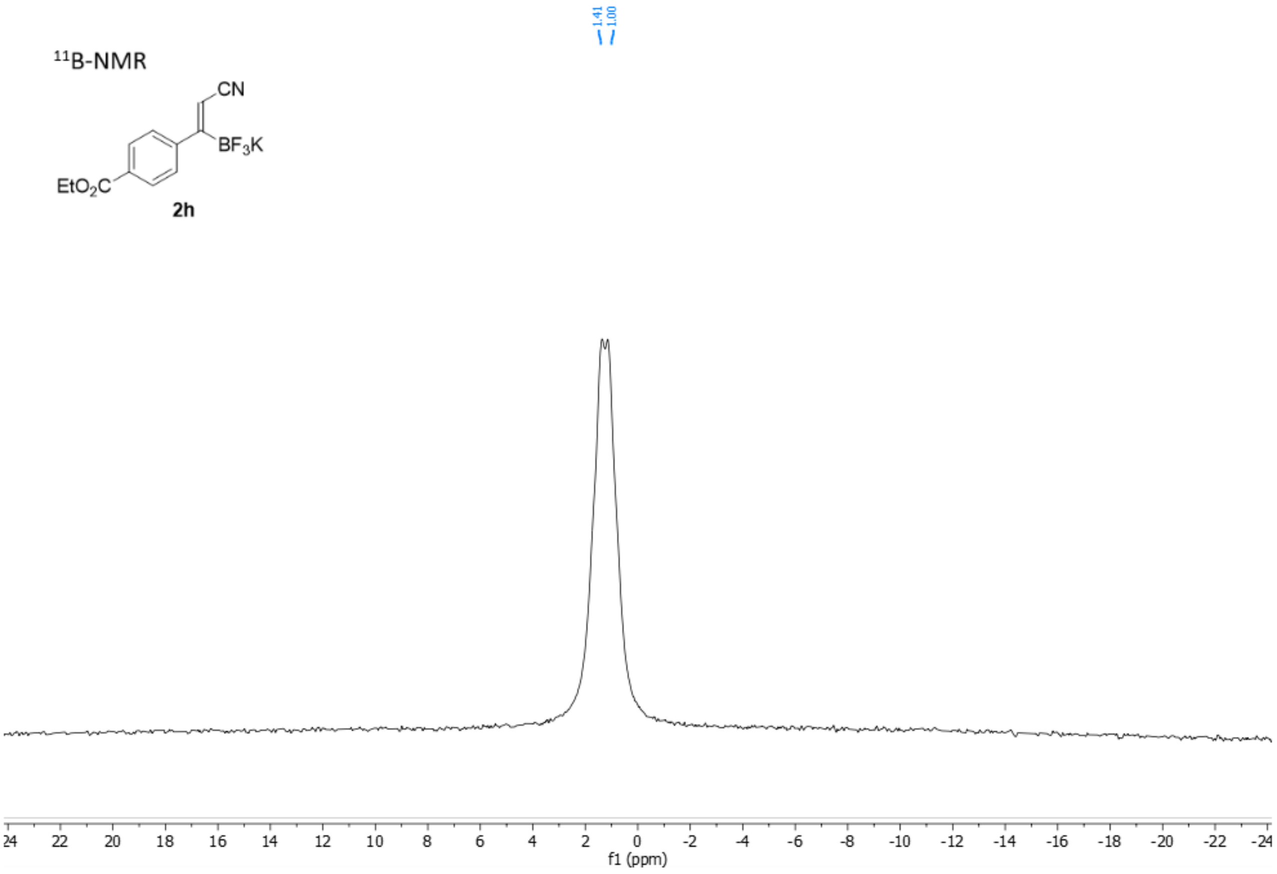

^{19}F-NMR (470 MHz, DMSO-d_6) of **2h**:

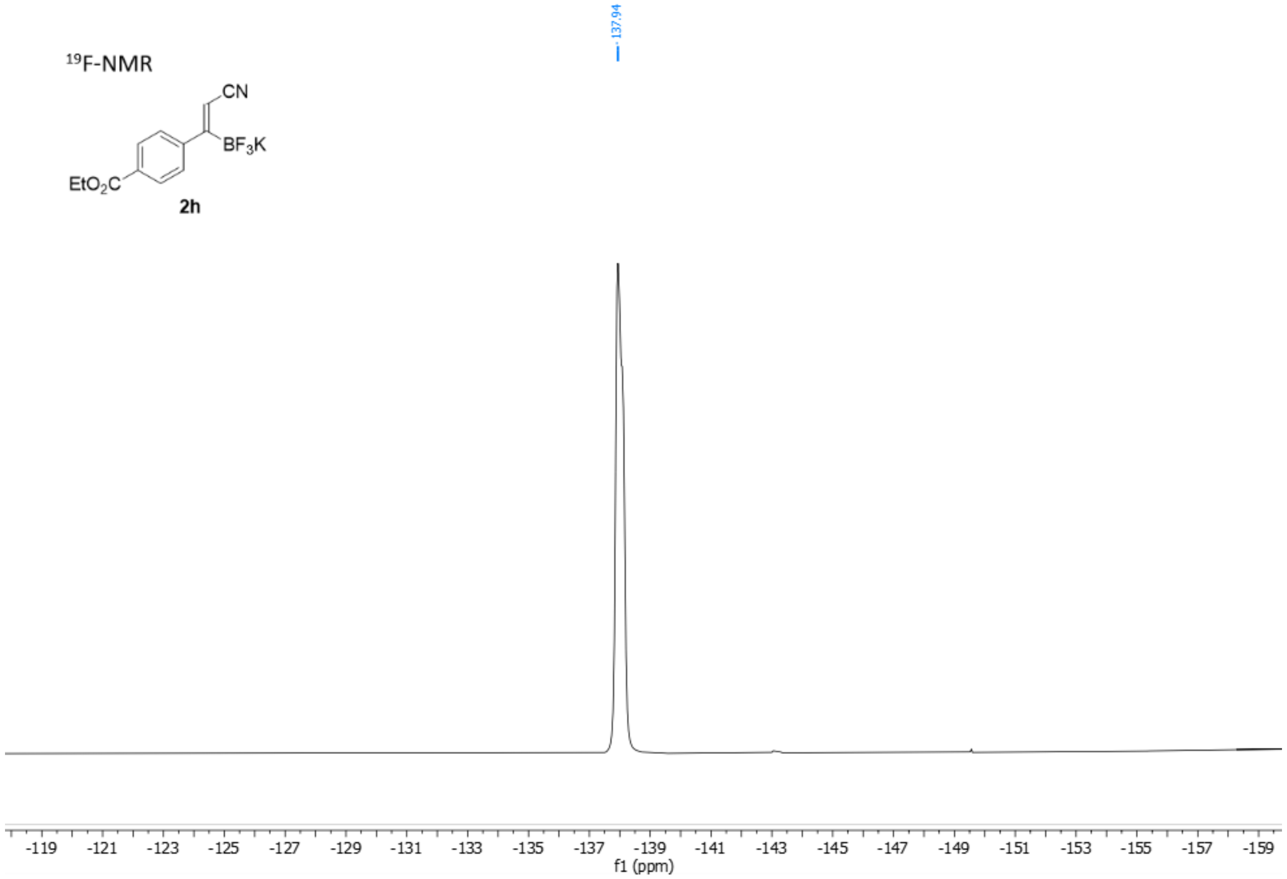

Ethyl (*E*)-3-(2-cyano-1-(trifluoro-λ^4-boraneyl)vinyl)benzoate, potassium salt (2i)

^{1}H-NMR (500 MHz, DMSO-d_6) of the reaction mixture of **2i**:

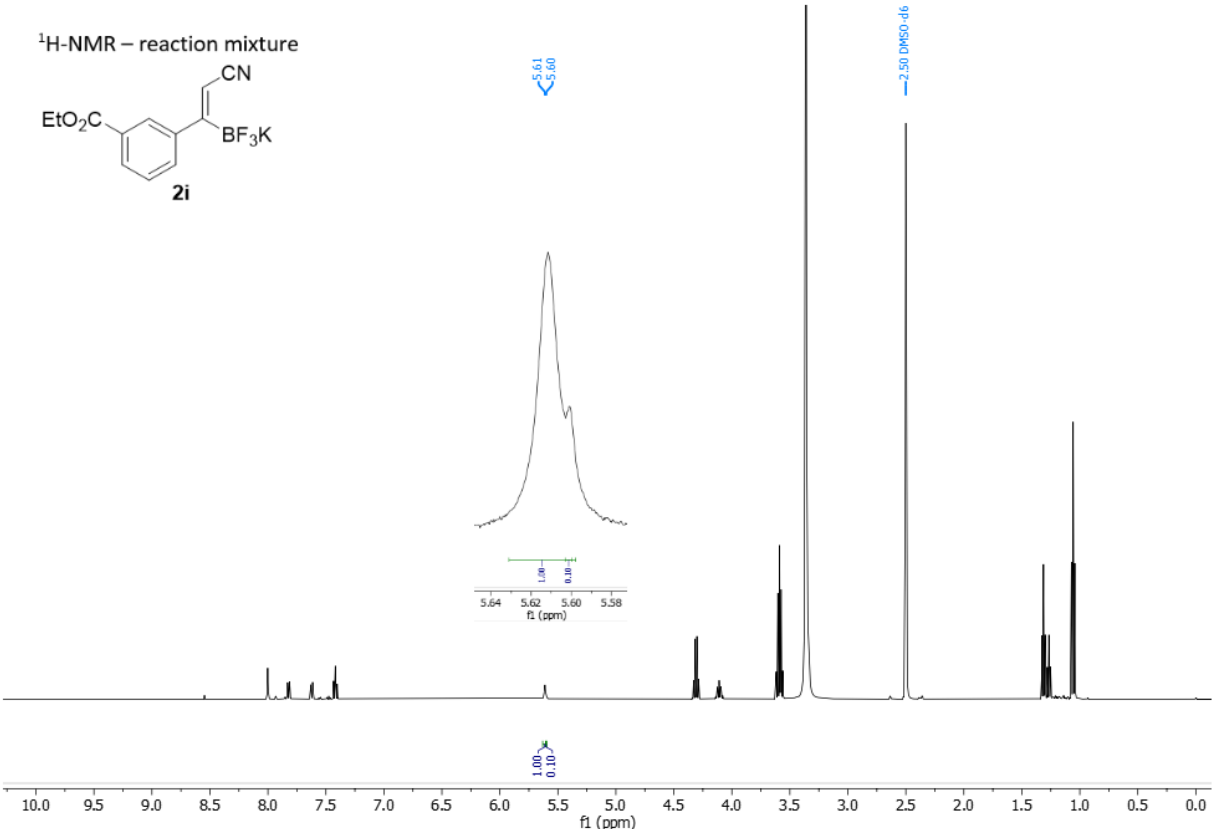

^{1}H-NMR (500 MHz, DMSO-d_6) of **2i**:

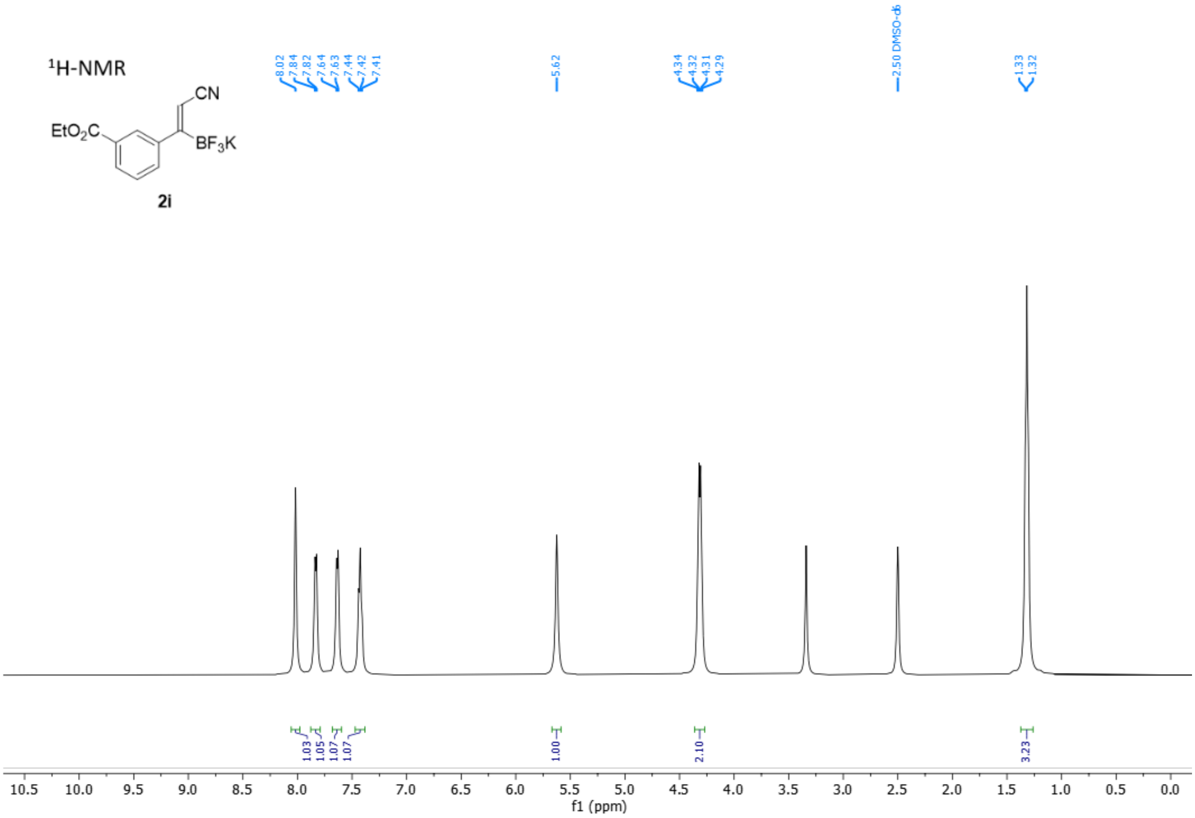

$^{13}C\{^1H\}$-NMR (126 MHz, DMSO-d_6) of **2i**:

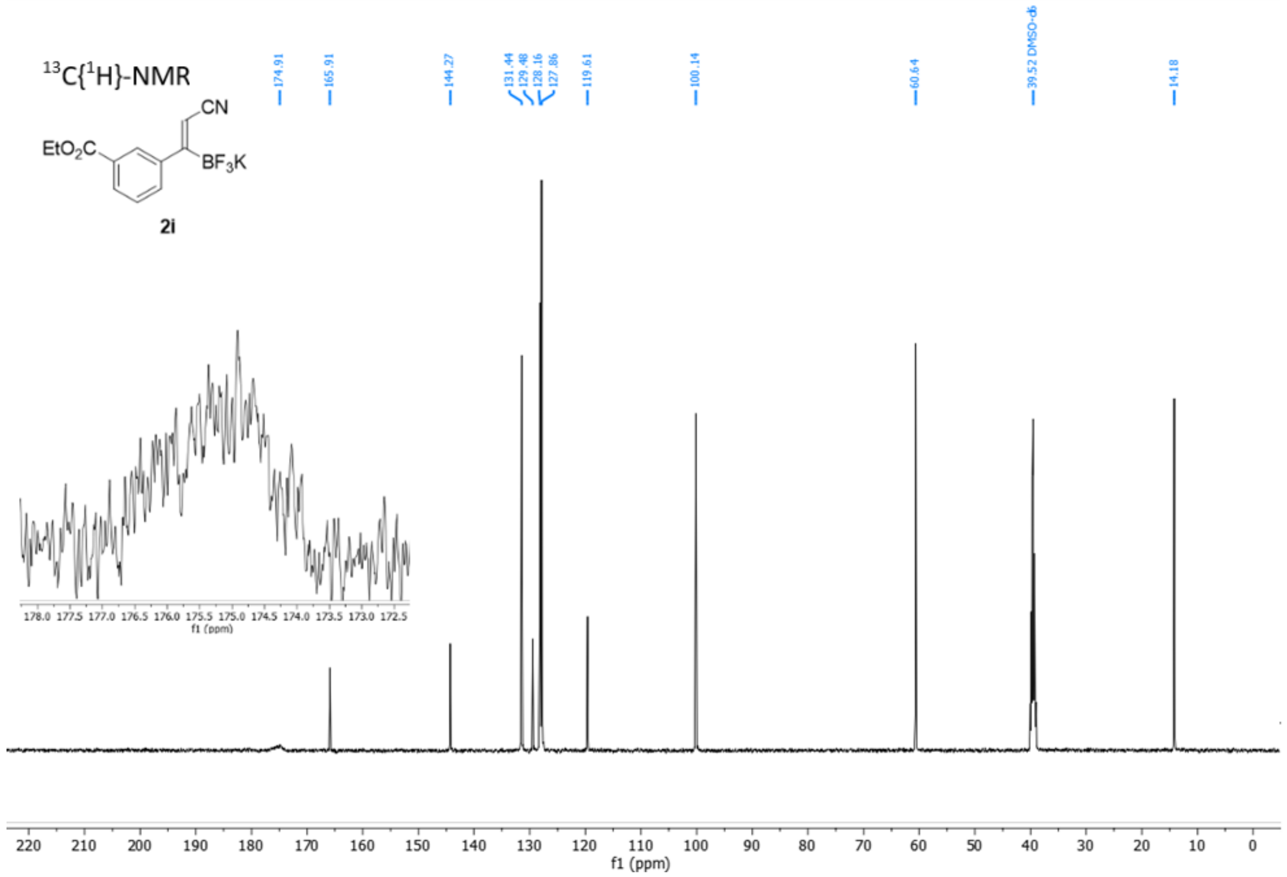

^{11}B-NMR (160 MHz, DMSO-d_6) of **2i**:

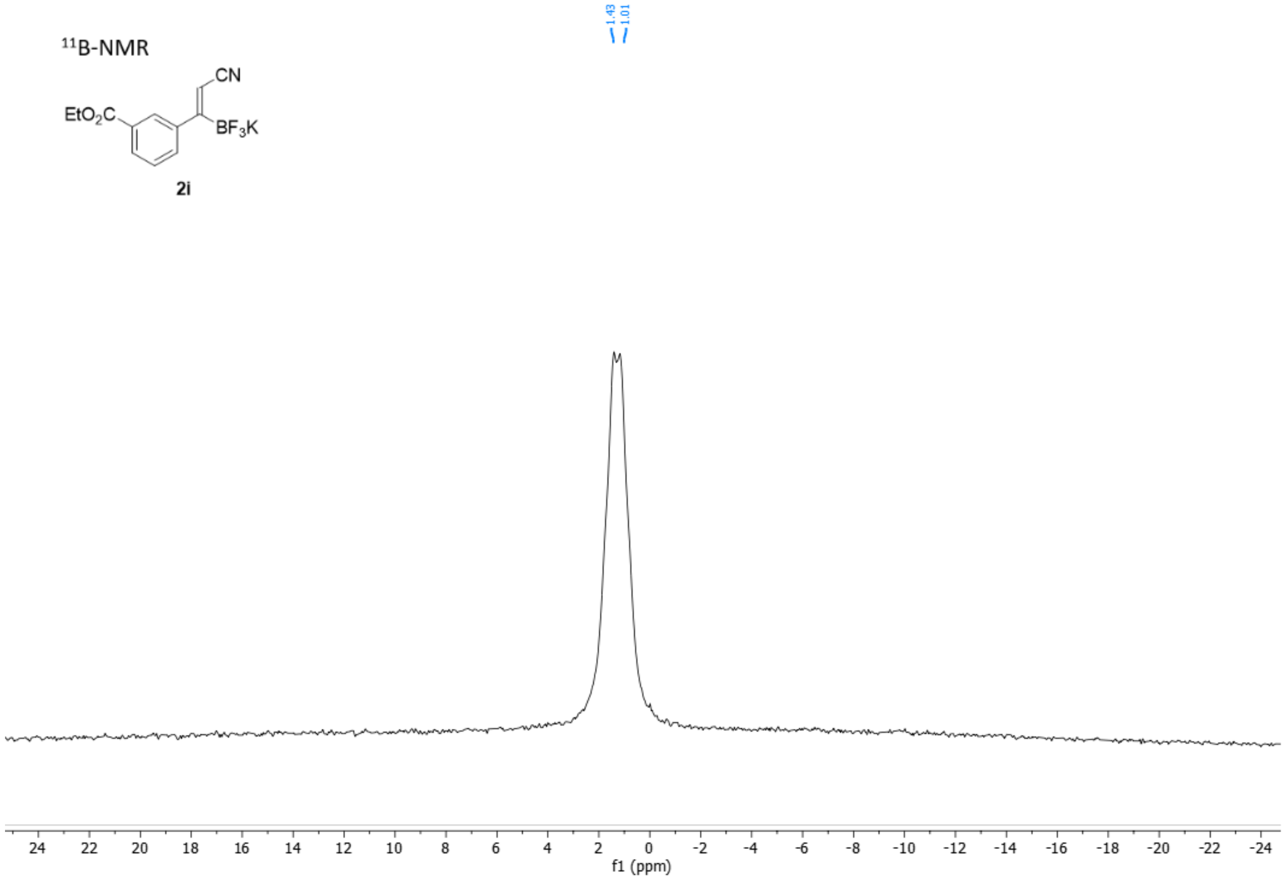

^{19}F-NMR (470 MHz, DMSO-d_6) of **2i**:

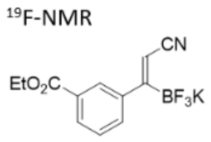

2i

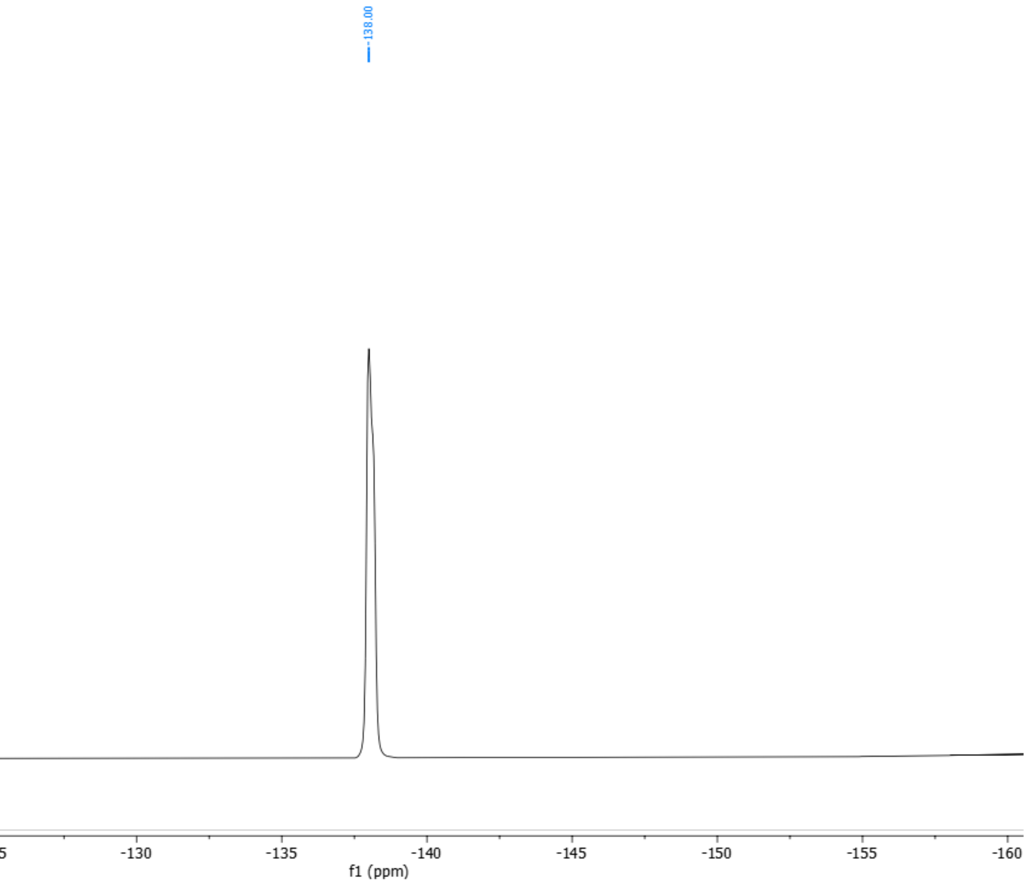

(*E*)-3-(quinolin-3-yl)-3-(trifluoro-λ^4-boraneyl)acrylonitrile, potassium salt (2j)

^{1}H-NMR (500 MHz, DMSO-d_6) of the reaction mixture of **2j**:

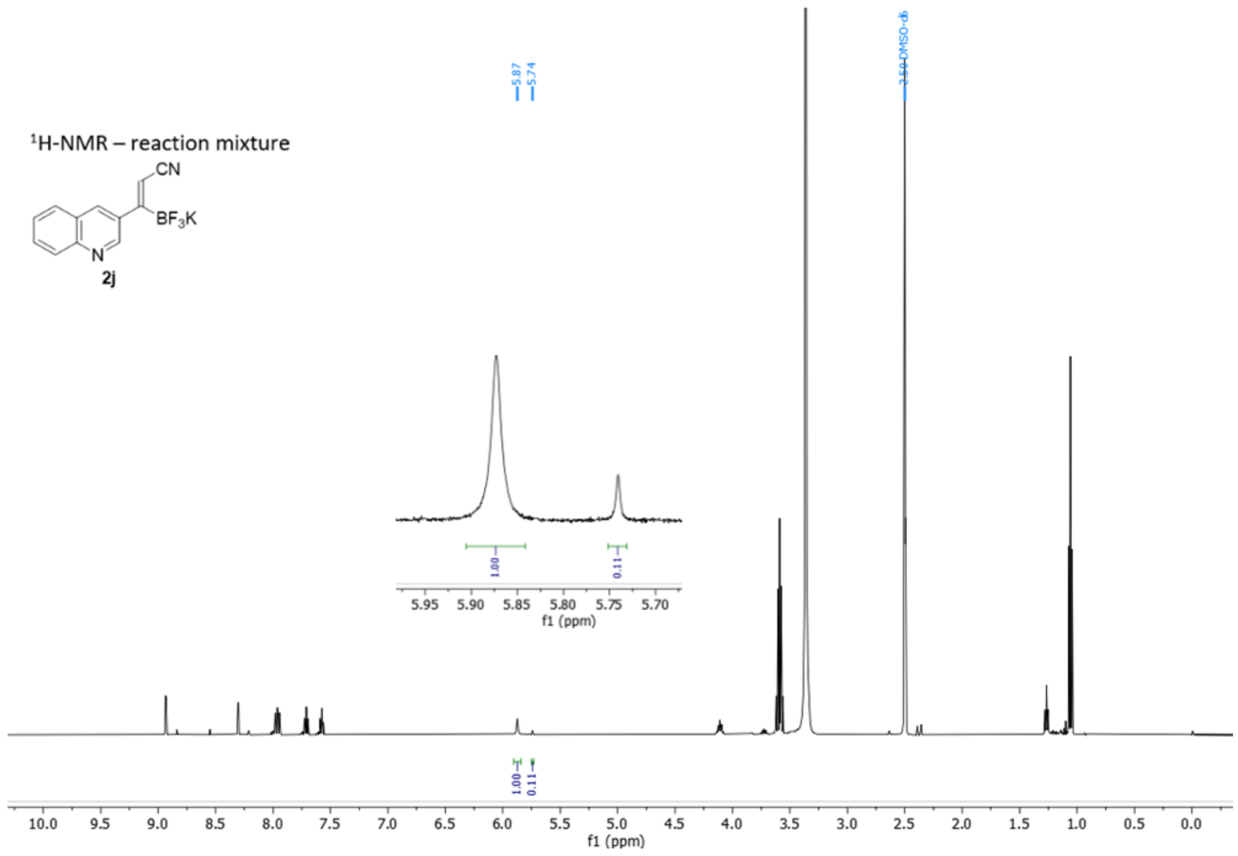

^{1}H-NMR (500 MHz, DMSO-d_6) of **2j**:

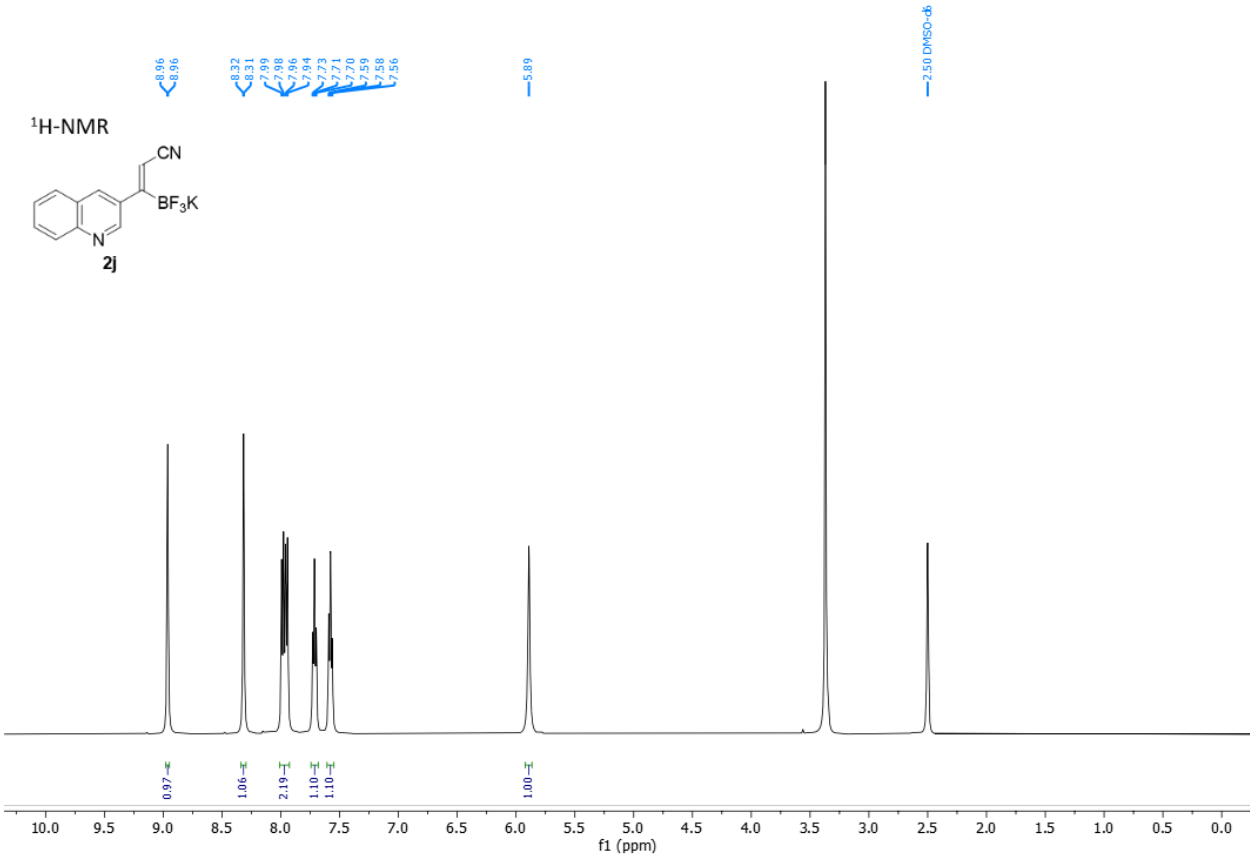

$^{13}C\{^1H\}$-NMR (126 MHz, DMSO-d_6) of **2j**:

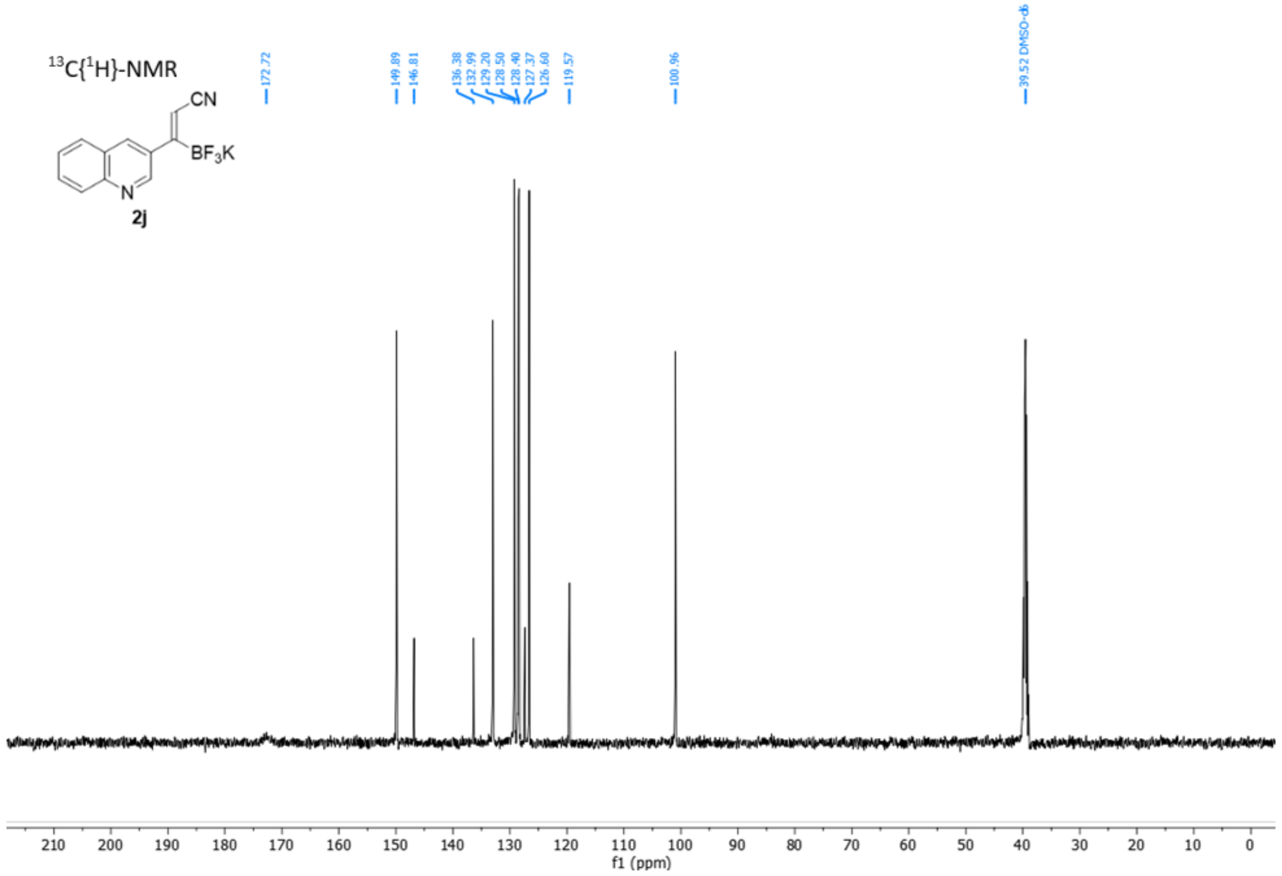

^{11}B-NMR (160 MHz, DMSO-d_6) of **2j**:

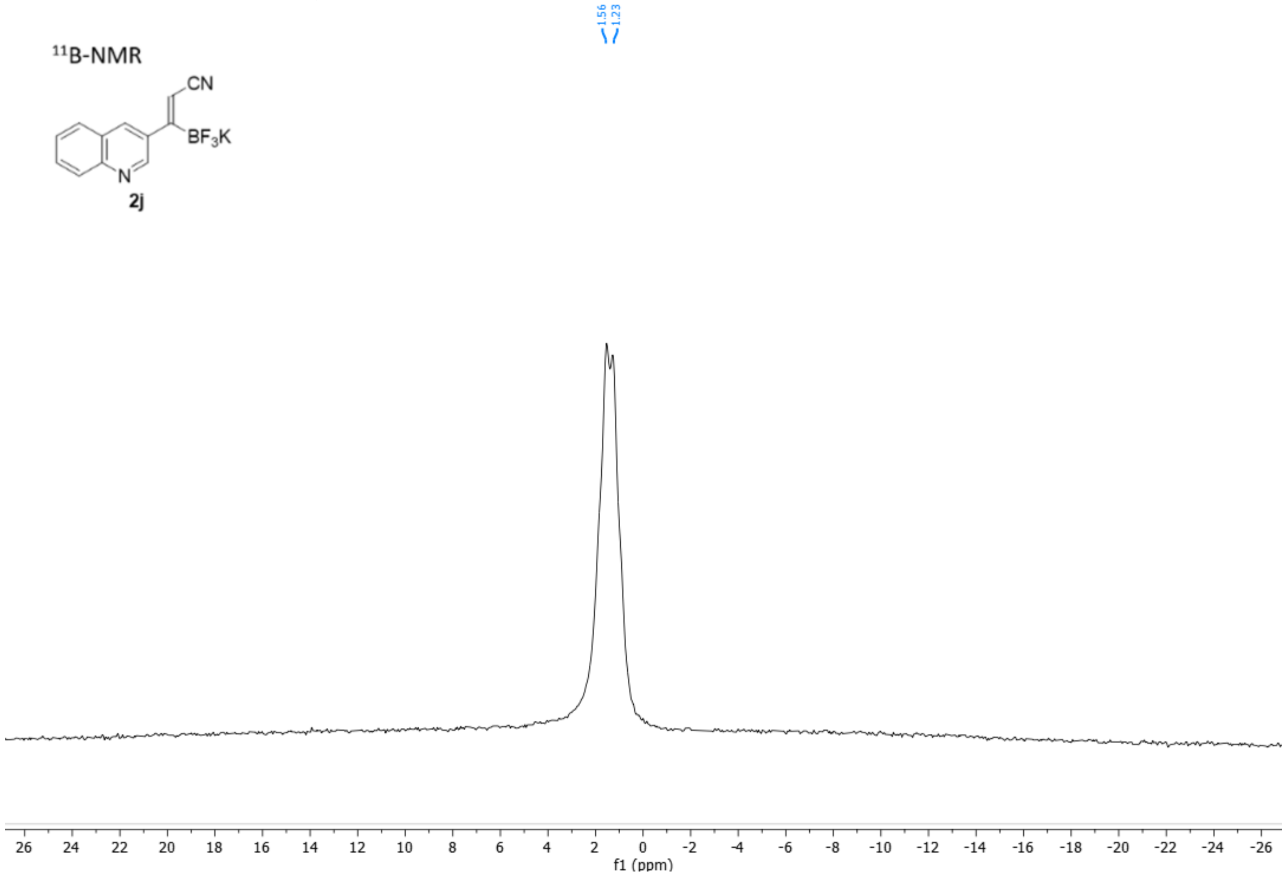

^{19}F-NMR (470 MHz, DMSO-d_6) of **2j**:

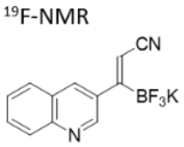

2j

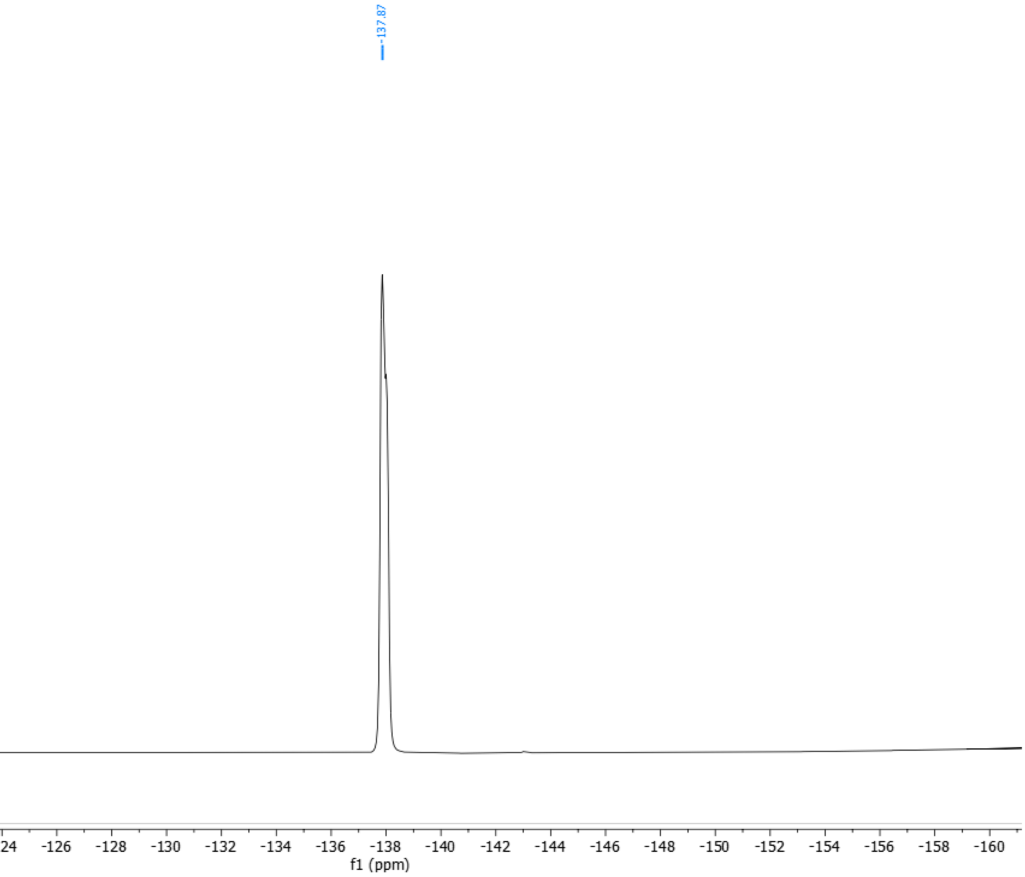

(*E*)-3-(6-bromopyridin-3-yl)-3-(trifluoro-λ⁴-boraneyl)acrylonitrile, potassium salt (2k)

^{1}H-NMR (500 MHz, DMSO-d_6) of the reaction mixture of **2k**:

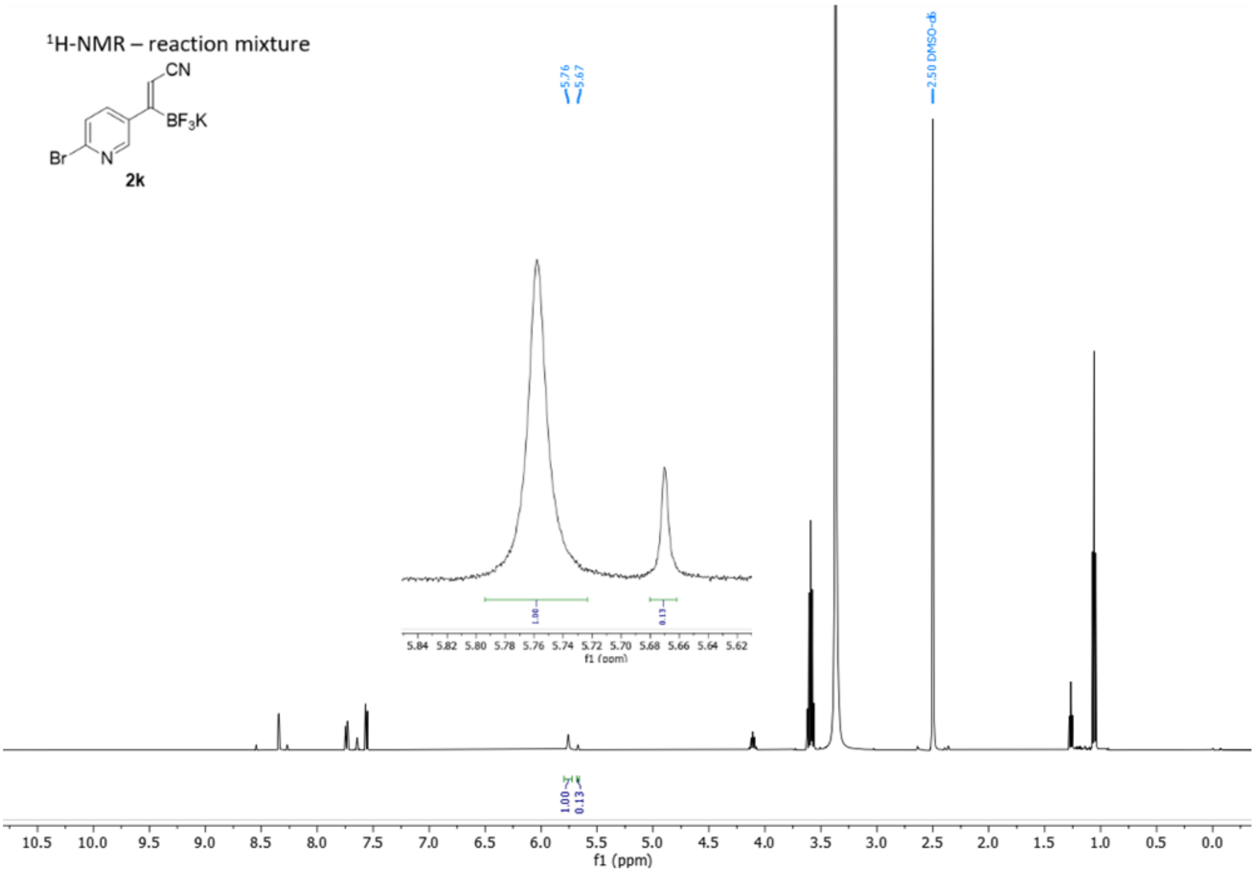

^{1}H-NMR (500 MHz, DMSO-d_6) of **2k**:

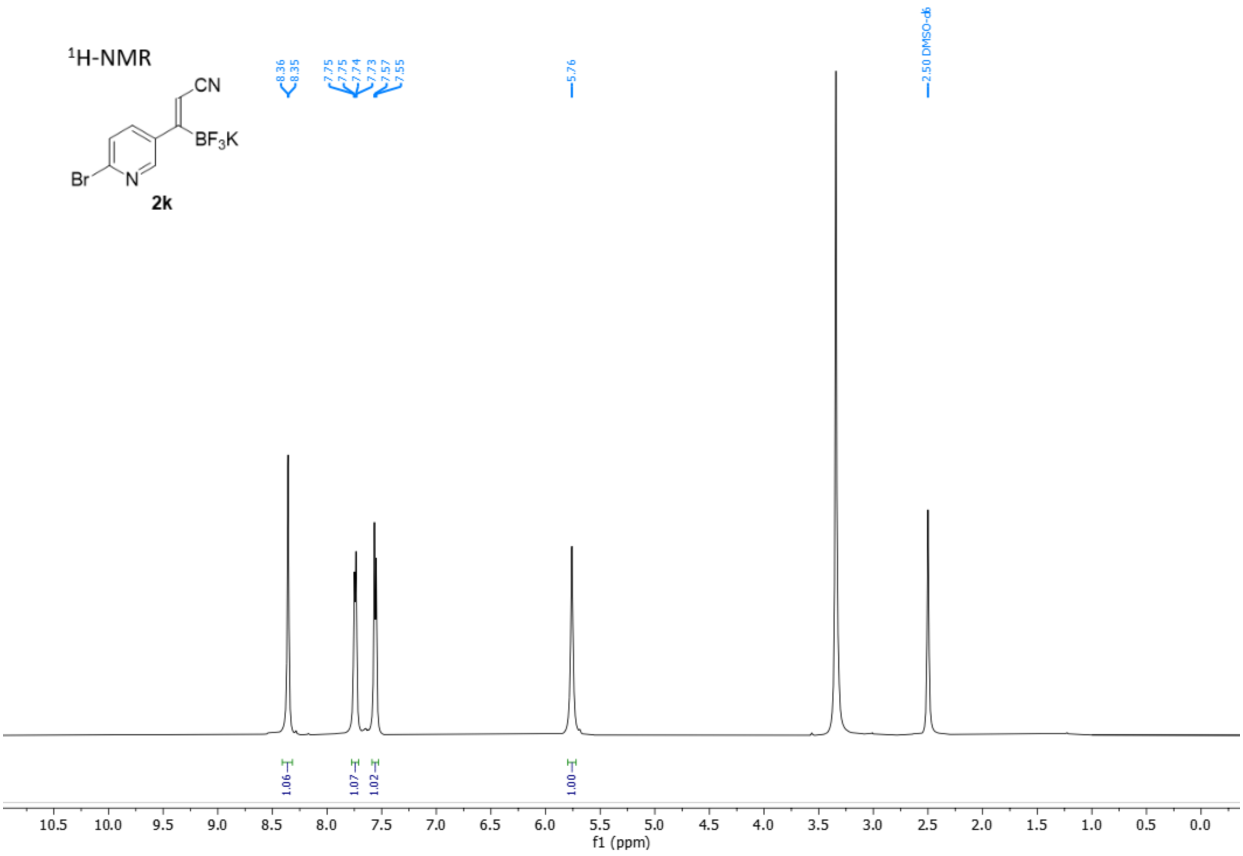

$^{13}C\{^1H\}$-NMR (126 MHz, DMSO-d_6) of **2k**:

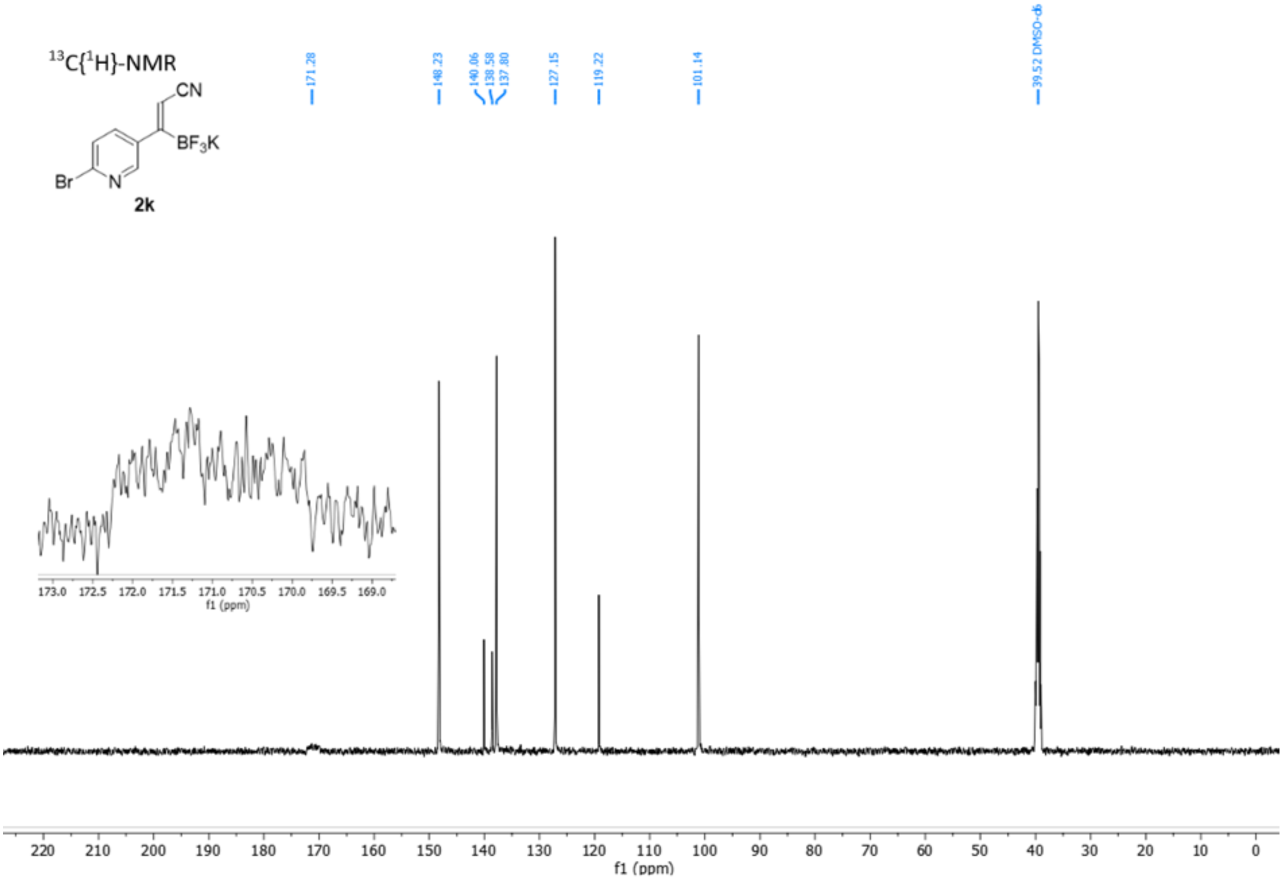

^{11}B-NMR (160 MHz, DMSO-d_6) of **2k**:

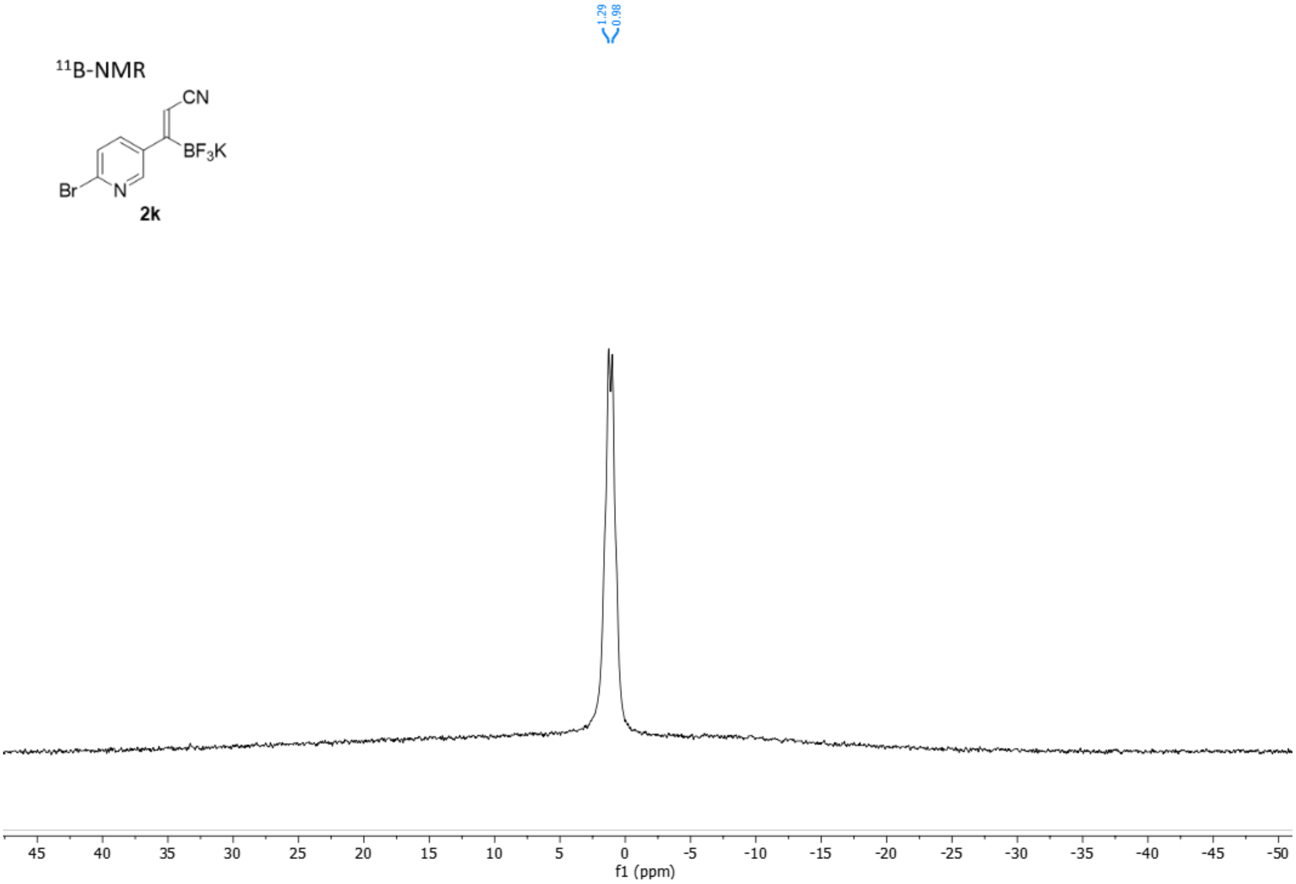

^{19}F-NMR (470 MHz, DMSO-d_6) of **2k**:

^{19}F-NMR

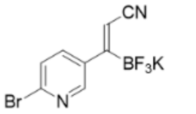

2k

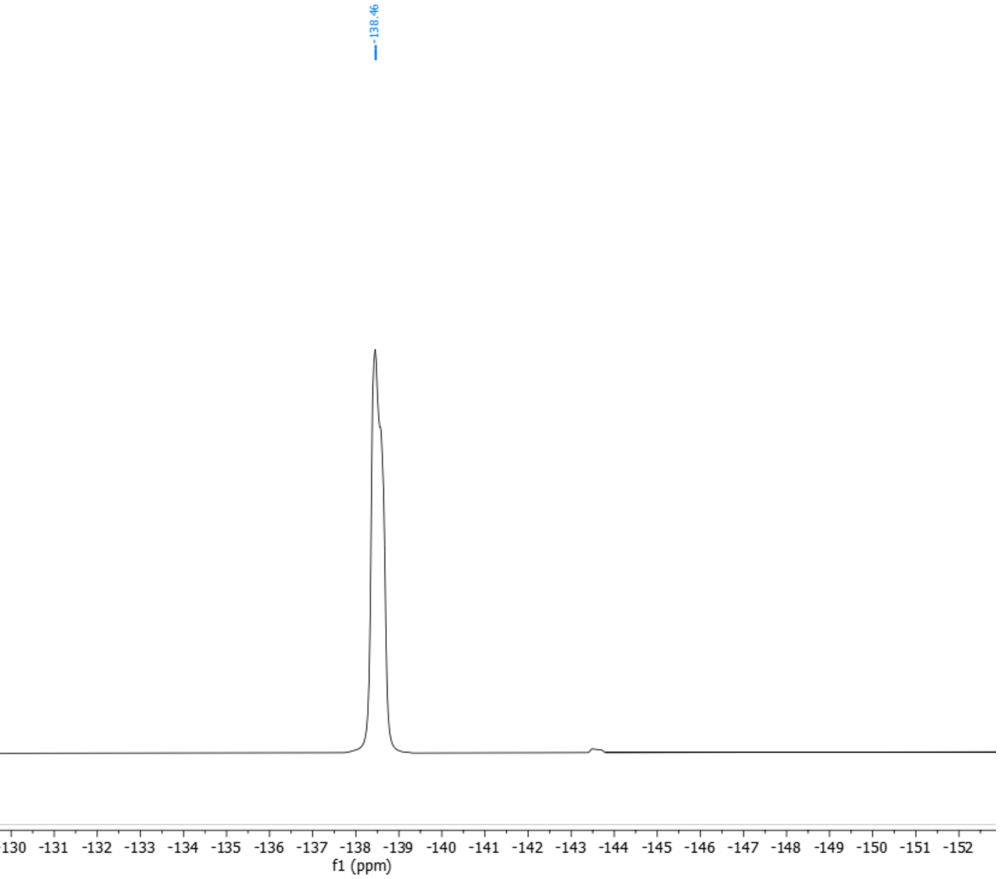
-138.46
-124 -125 -126 -127 -128 -129 -130 -131 -132 -133 -134 -135 -136 -137 -138 -139 -140 -141 -142 -143 -144 -145 -146 -147 -148 -149 -150 -151 -152
f1 (ppm)

(*E*)-3-(2-chloropyridin-4-yl)-3-(trifluoro-λ[4]-boraneyl)acrylonitrile, potassium salt (2l)

^{1}H-NMR (500 MHz, DMSO-*d*$_6$) of the reaction mixture of **2l**:

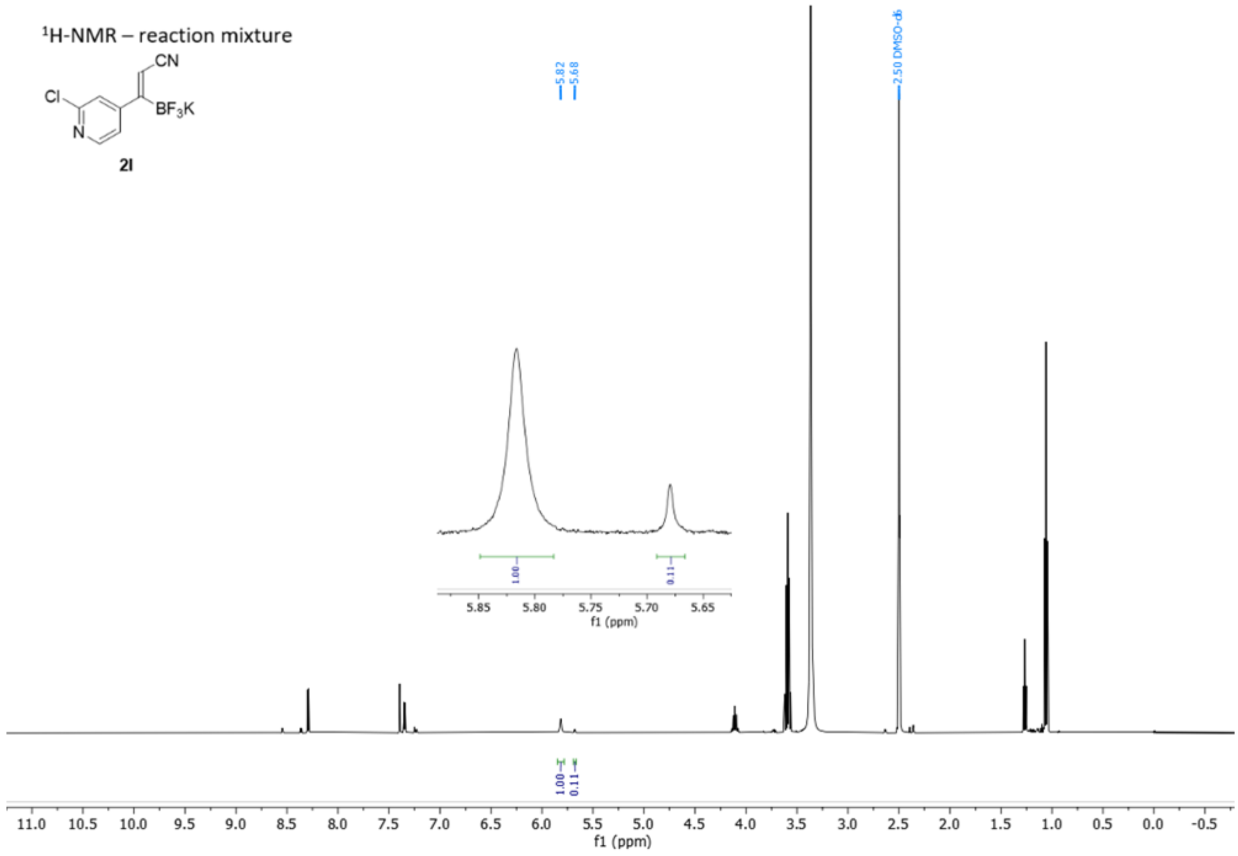

^{1}H-NMR (500 MHz, DMSO-*d*$_6$) of **2l**:

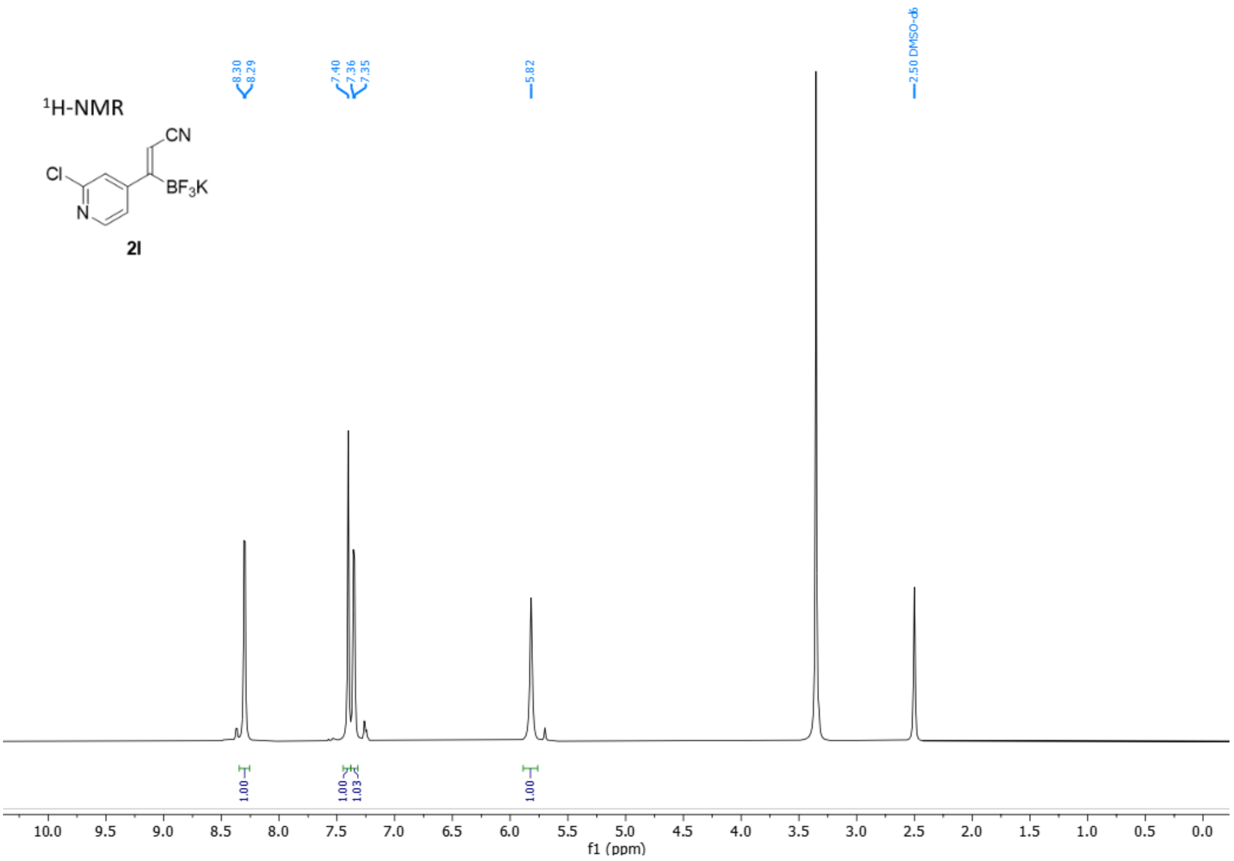

$^{13}C\{^1H\}$-NMR (126 MHz, DMSO-d_6) of **2l**:

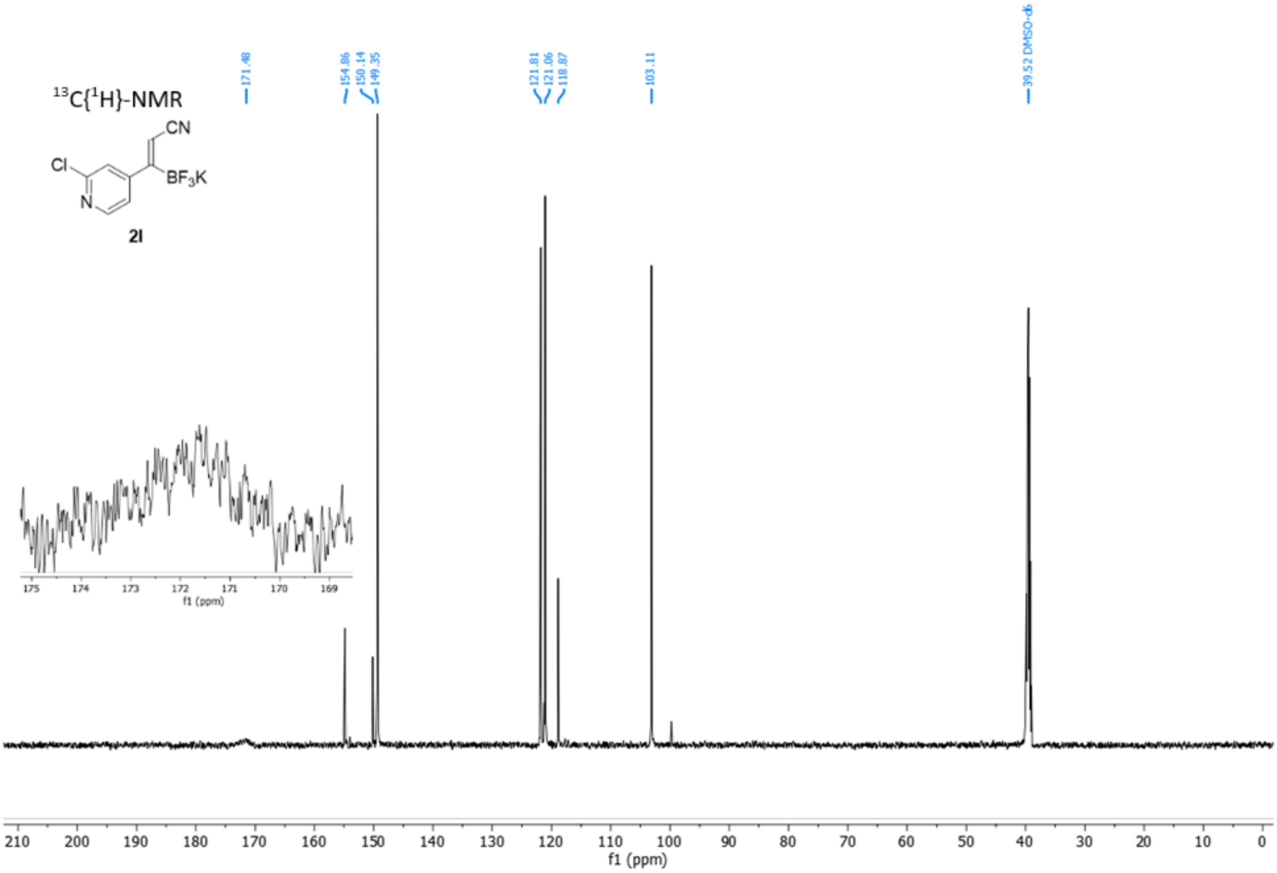

^{11}B-NMR (160 MHz, DMSO-d_6) of **2l**:

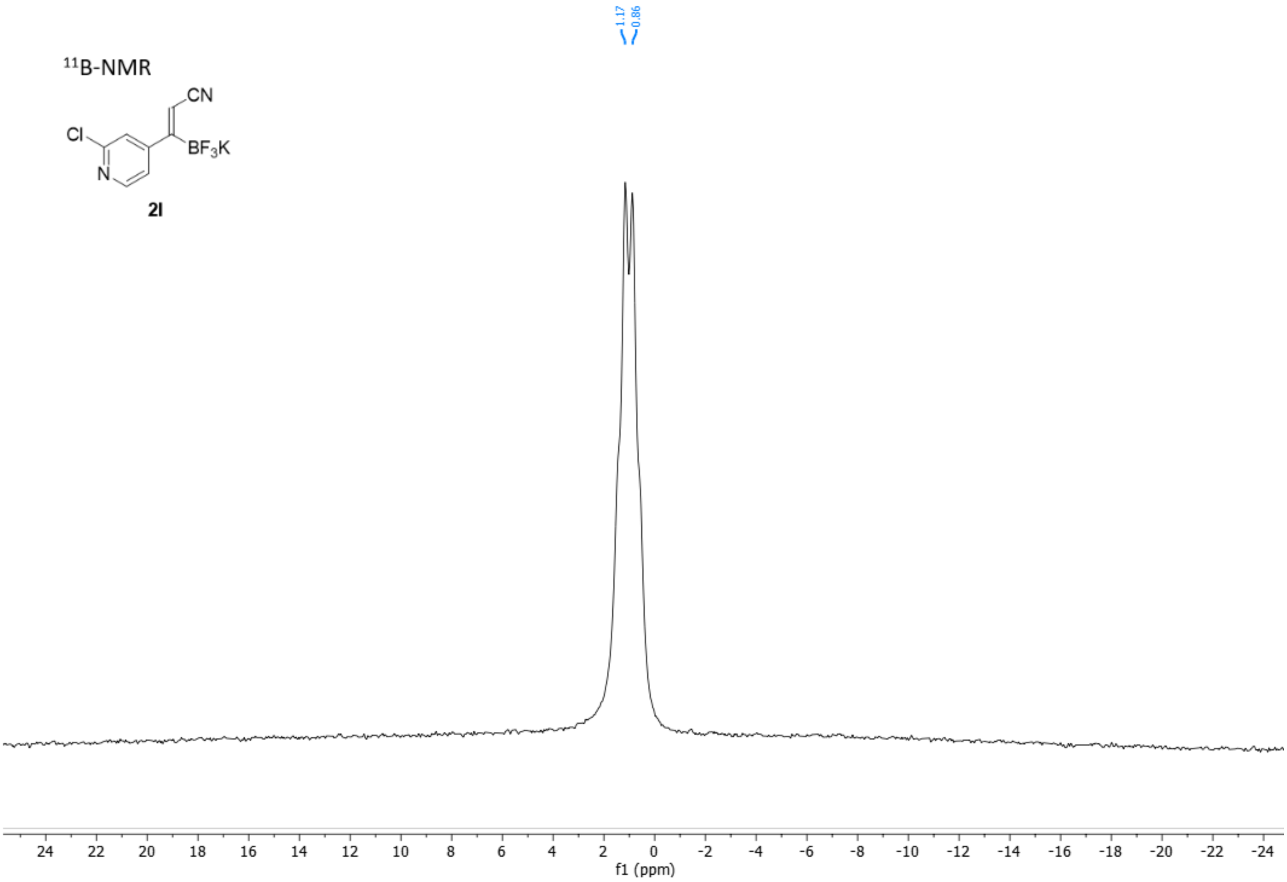

^{19}F-NMR (470 MHz, DMSO-d_6) of **2l**:

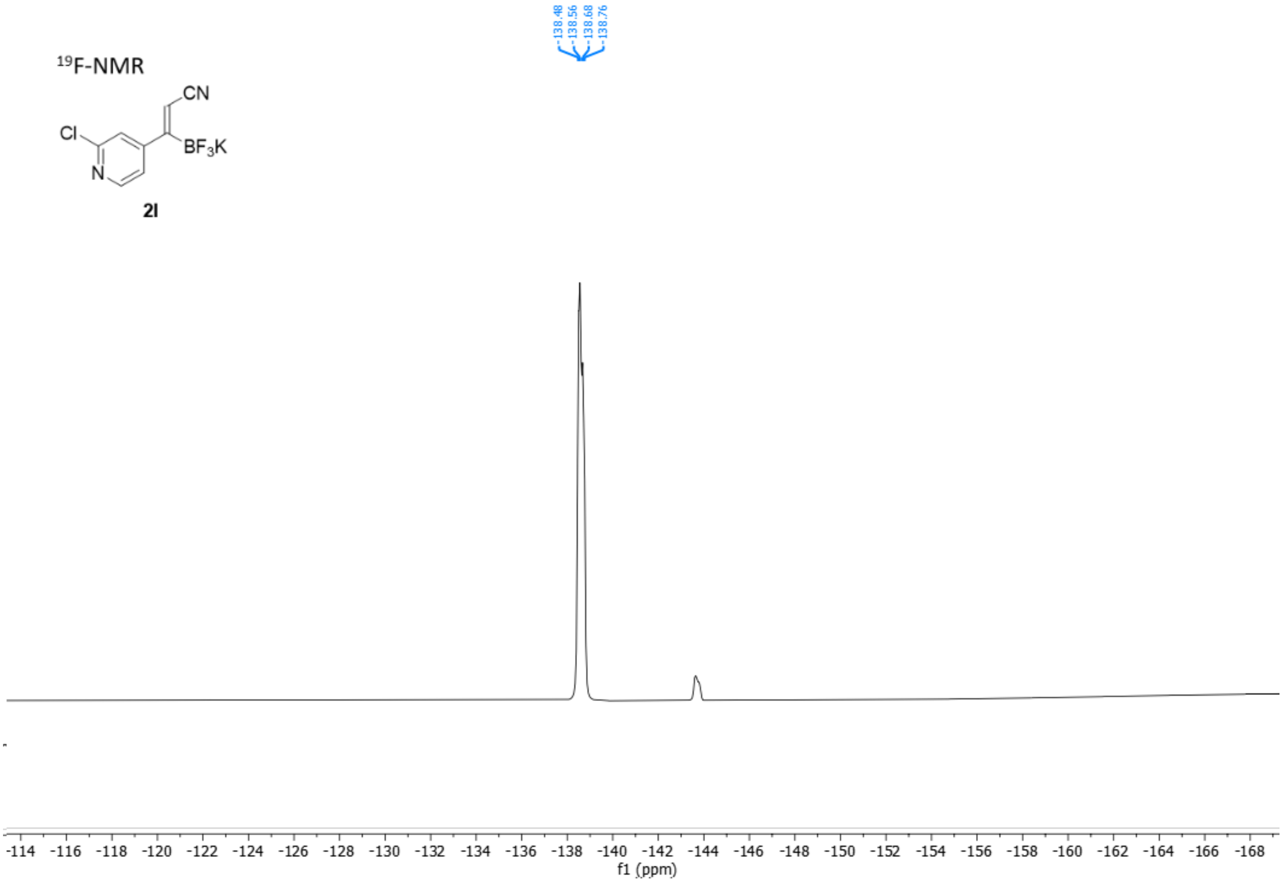

(*E*)-3-(4-chloropyridin-2-yl)-3-(trifluoro-λ[4]-boraneyl)acrylonitrile, potassium salt (2m)

^{1}H-NMR (500 MHz, DMSO-d_6) of the reaction mixture of **2m**:

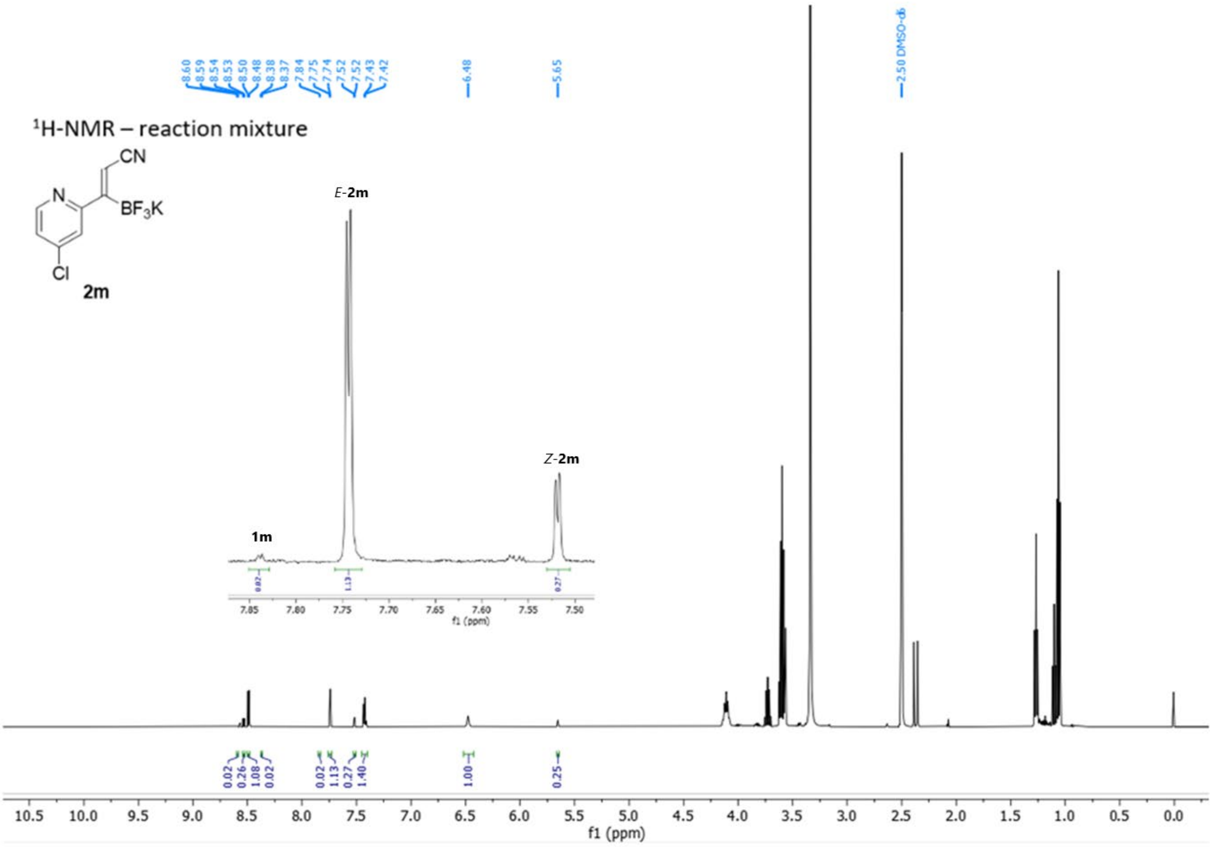

^{1}H-NMR (500 MHz, DMSO-d_6) of **2m**:

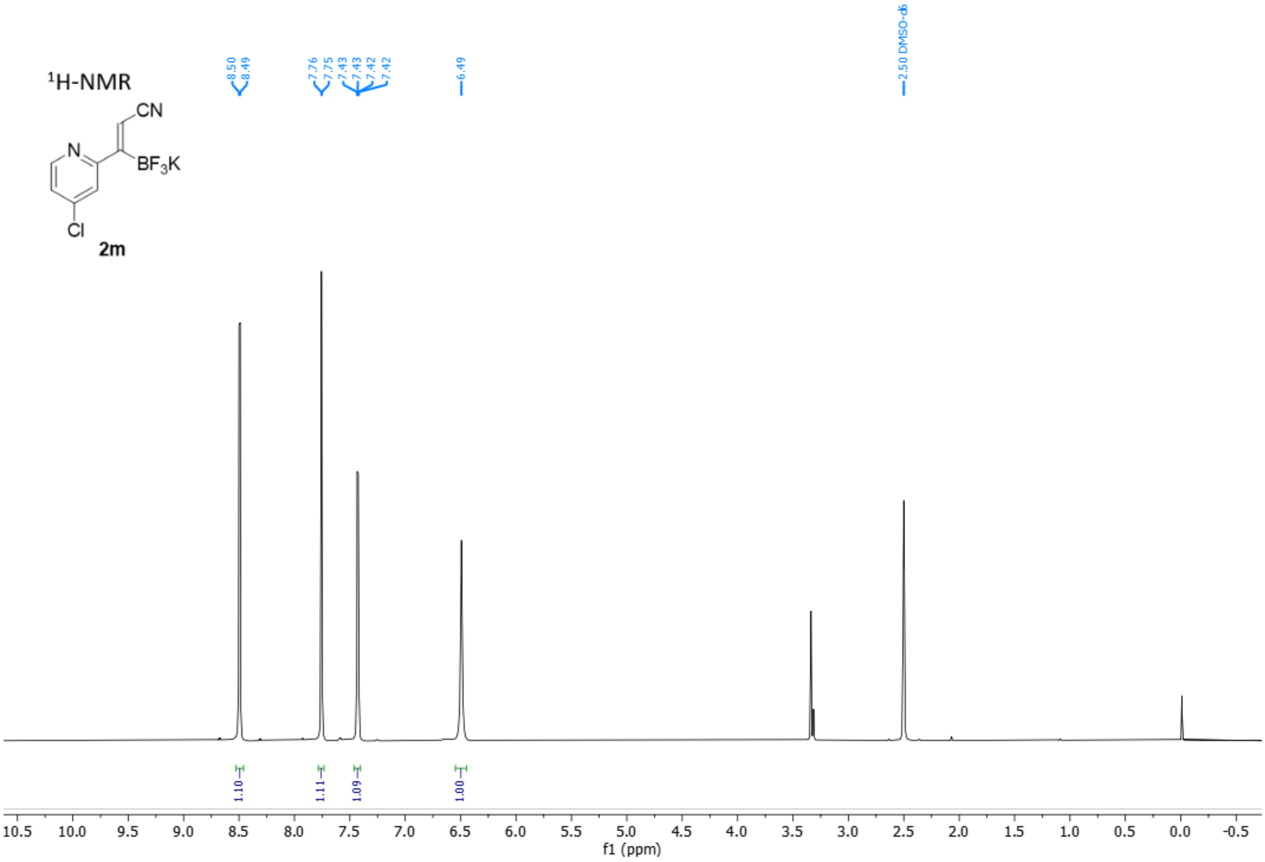

$^{13}C\{^1H\}$-NMR (126 MHz, DMSO-d_6) of **2m**:

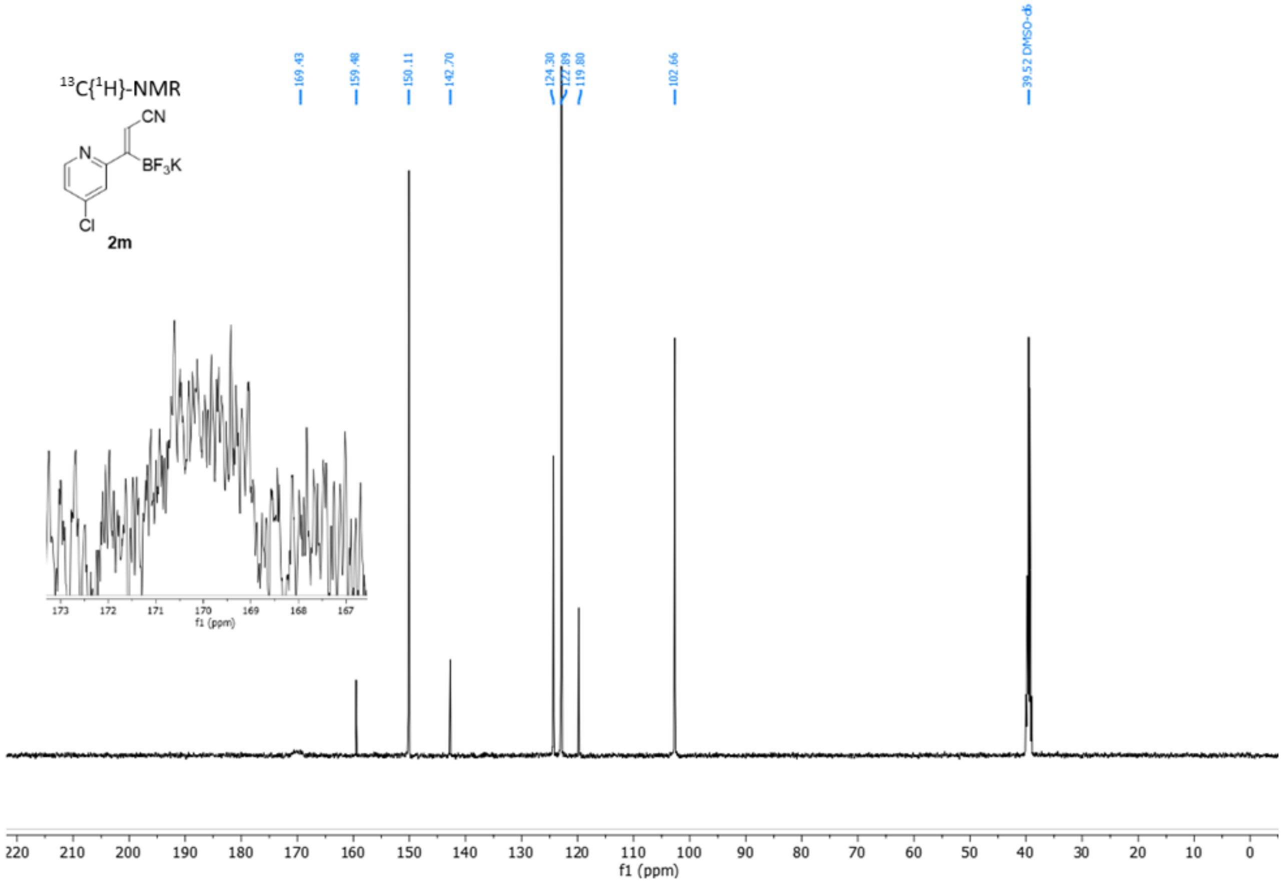

^{11}B-NMR (160 MHz, DMSO-d_6) of **2m**:

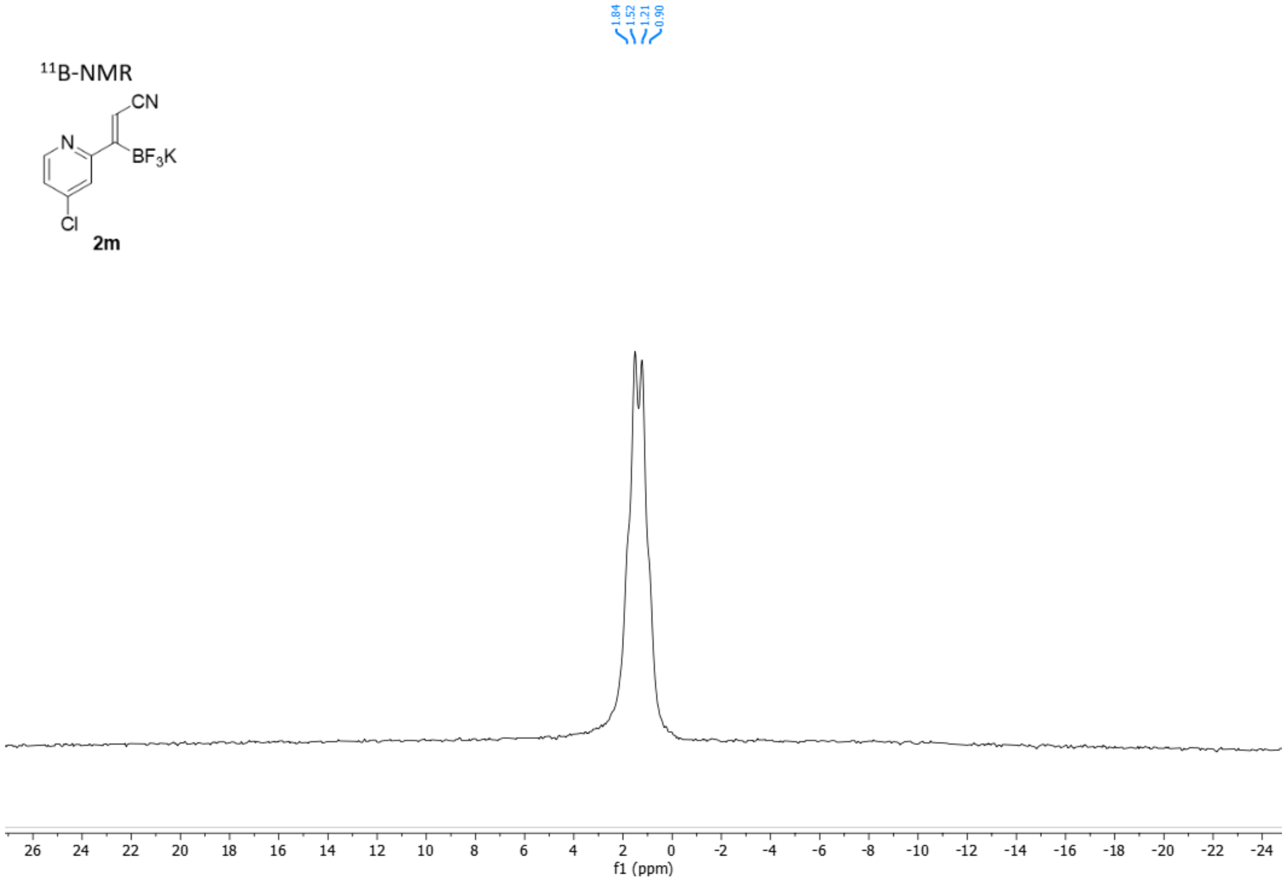

^{19}F-NMR (470 MHz, DMSO-d_6) of **2m**:

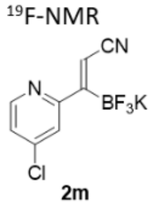

2m

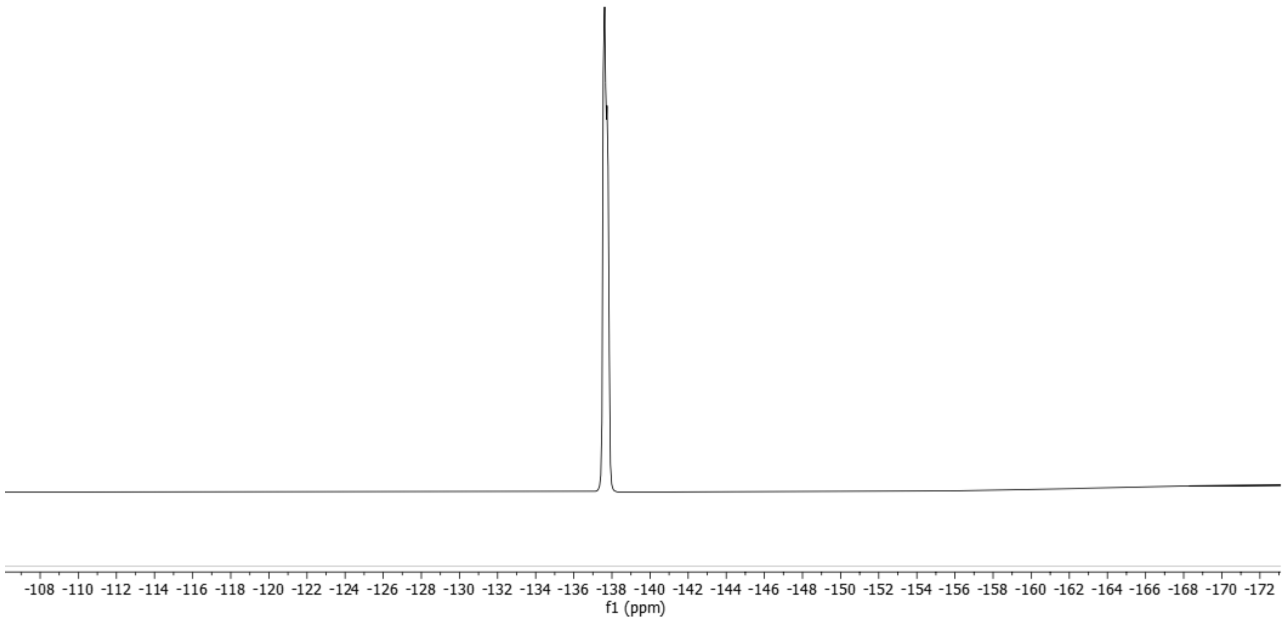

(*E*)-3-(5-bromopyridin-2-yl)-3-(trifluoro-λ4-boraneyl)acrylonitrile, potassium salt (2n)

^{1}H-NMR (500 MHz, DMSO-d_6) of the reaction mixture of **2n** before the isolation according to the procedure A:

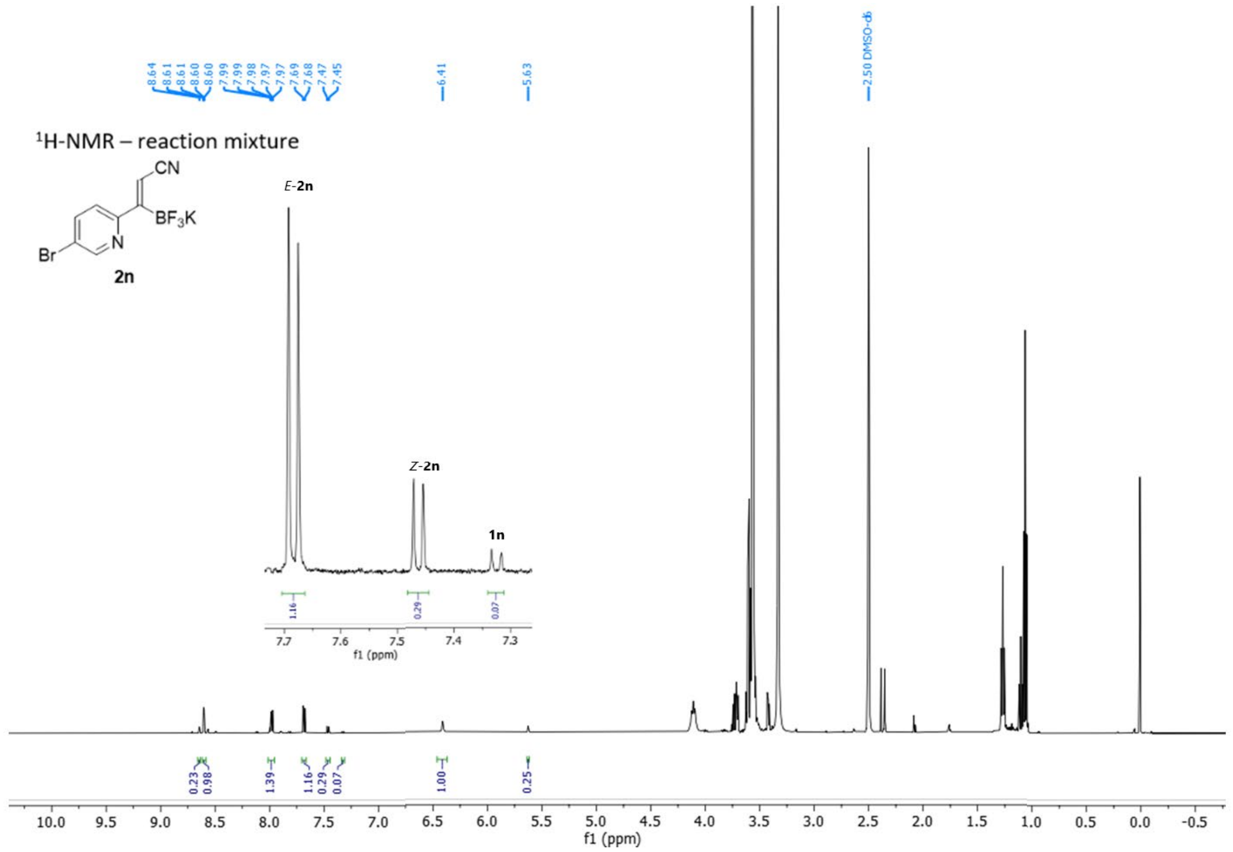

^{1}H-NMR (500 MHz, DMSO-d_6) of the reaction mixture of **2n** before the isolation according to the procedure B:

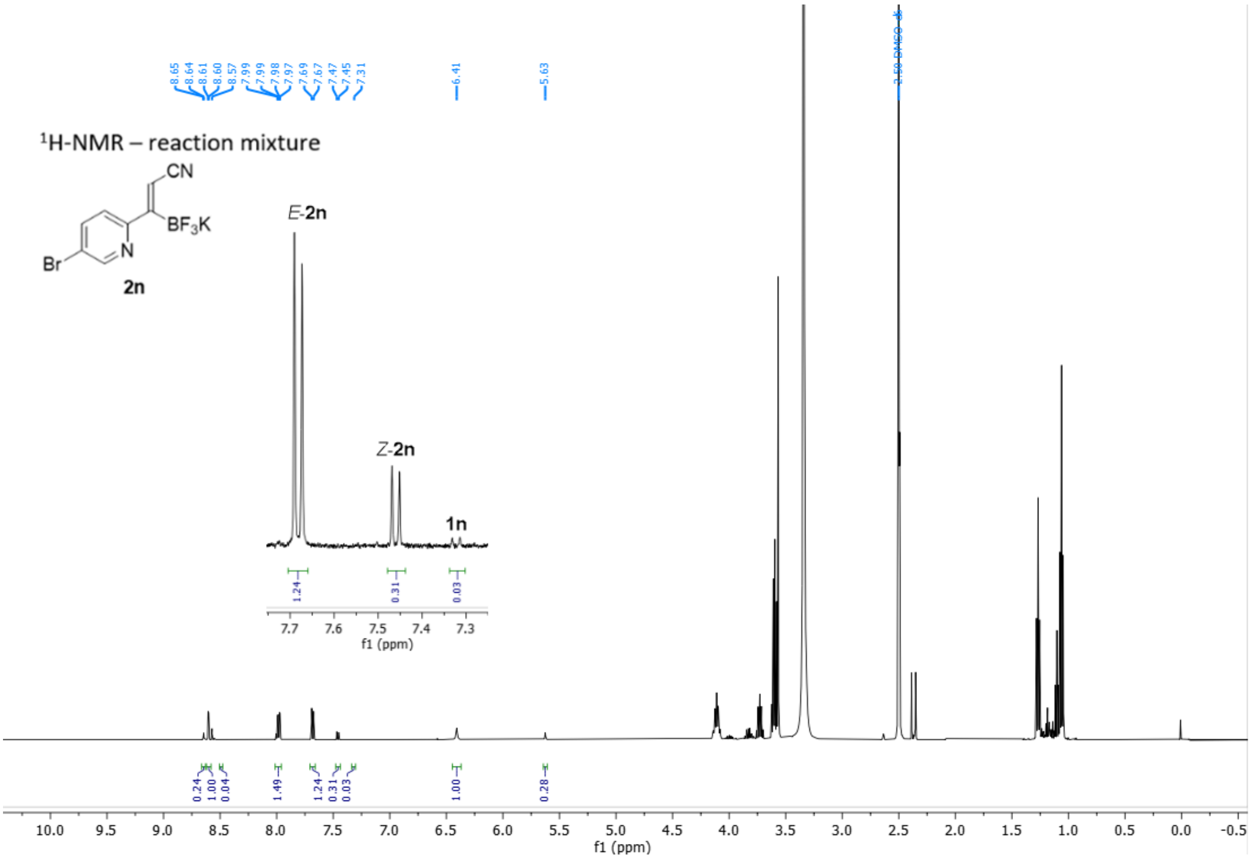

^{1}H-NMR (500 MHz, DMSO-d_6) of **2n** isolated according to procedure A, containing 10% of unkown impurity.

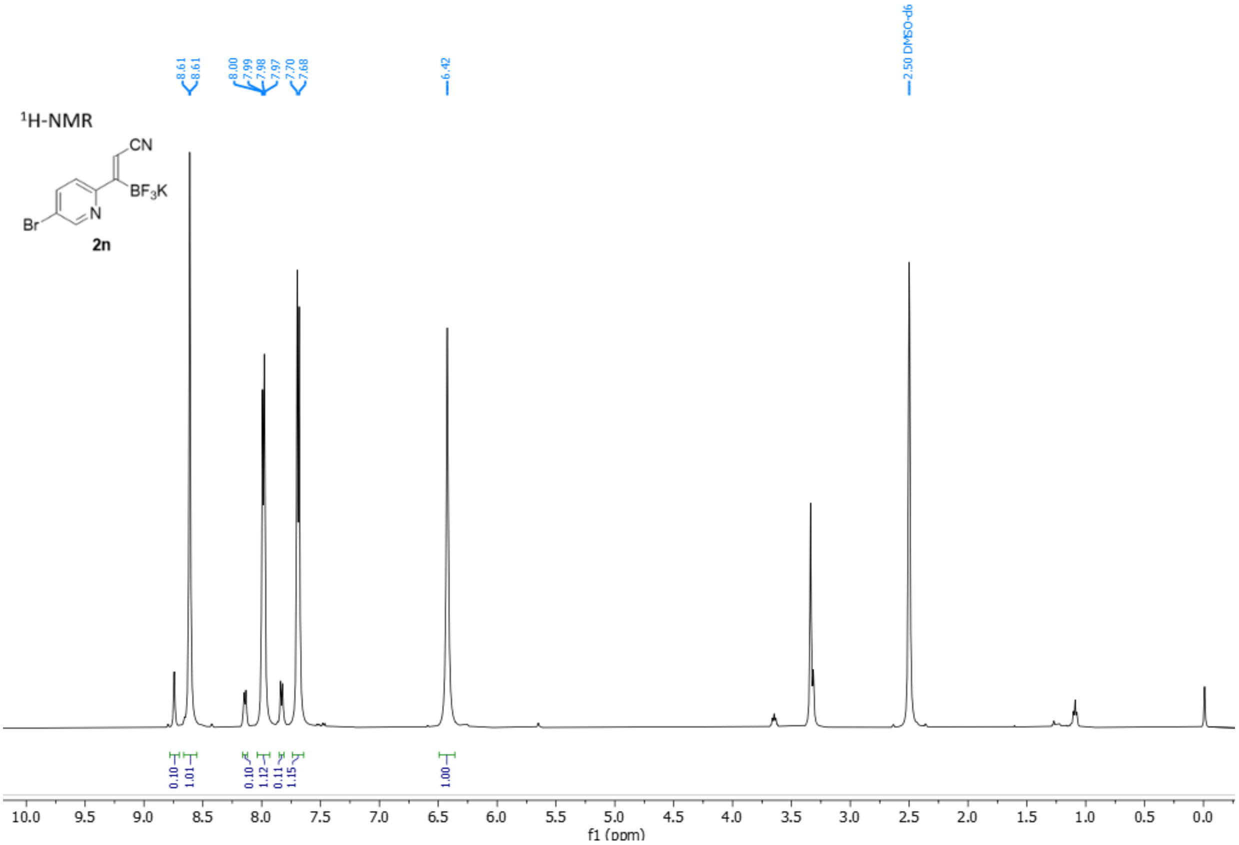

^{1}H-NMR (500 MHz, DMSO-d_6) of **2n** isolated according to procedure B:

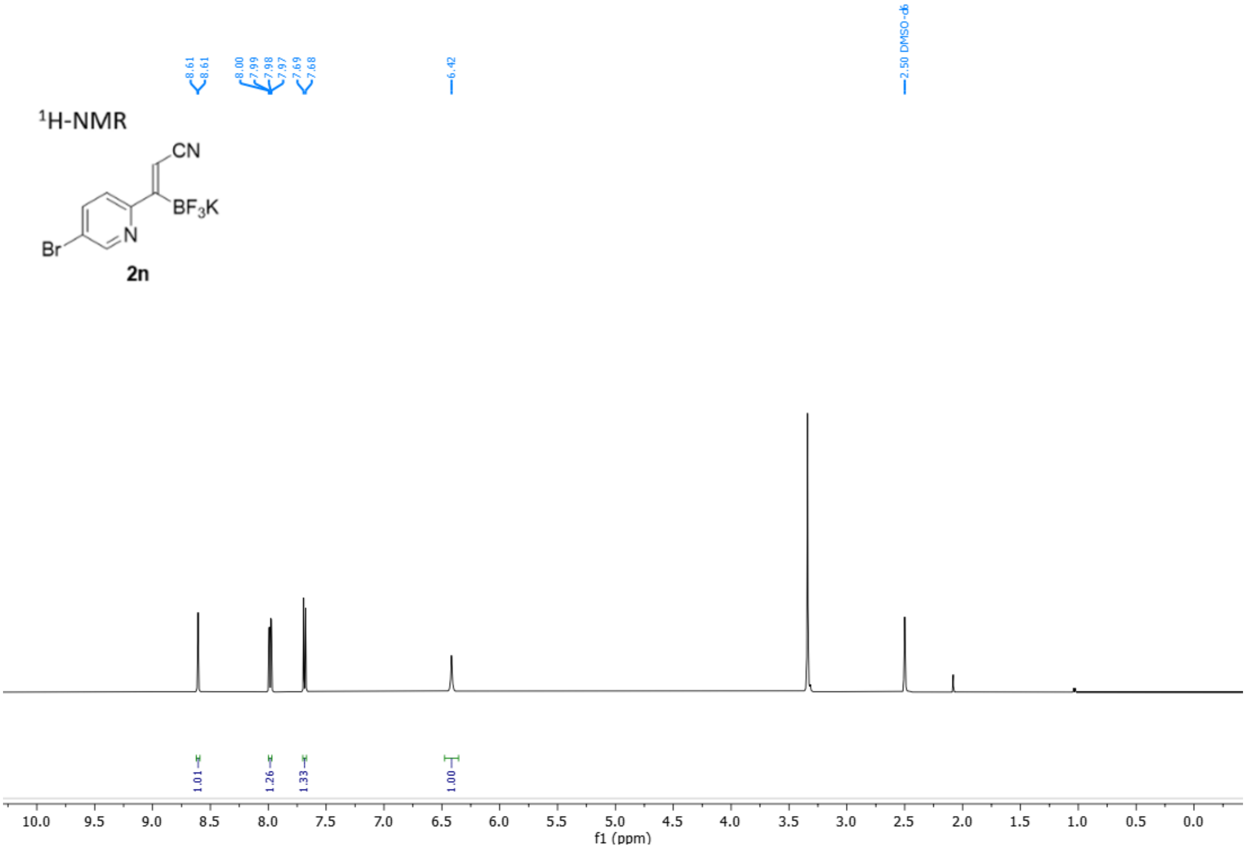

^{13}C{^{1}H}-NMR (126 MHz, DMSO-d_6) of **2n**:

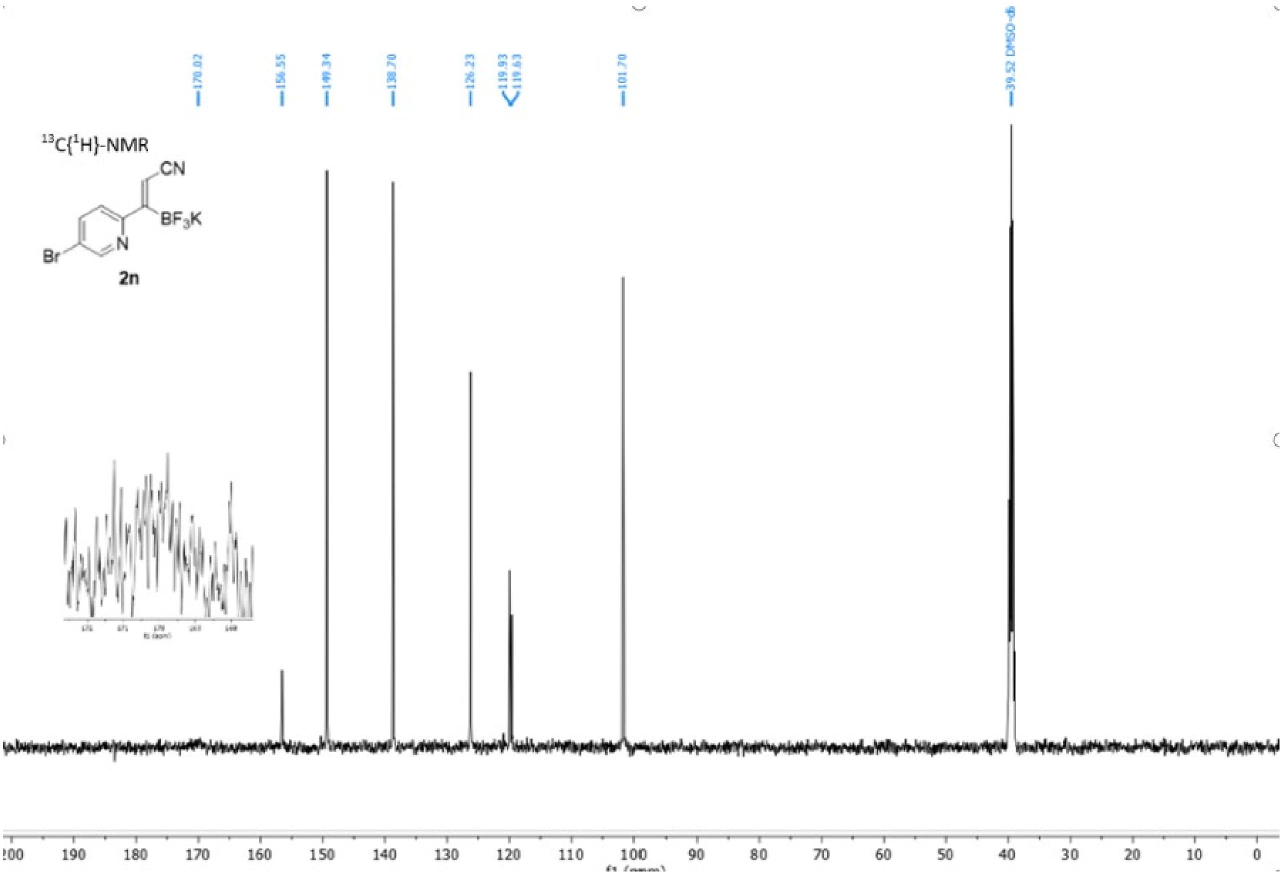

^{11}B-NMR (160 MHz, DMSO-d_6) of **2n**:

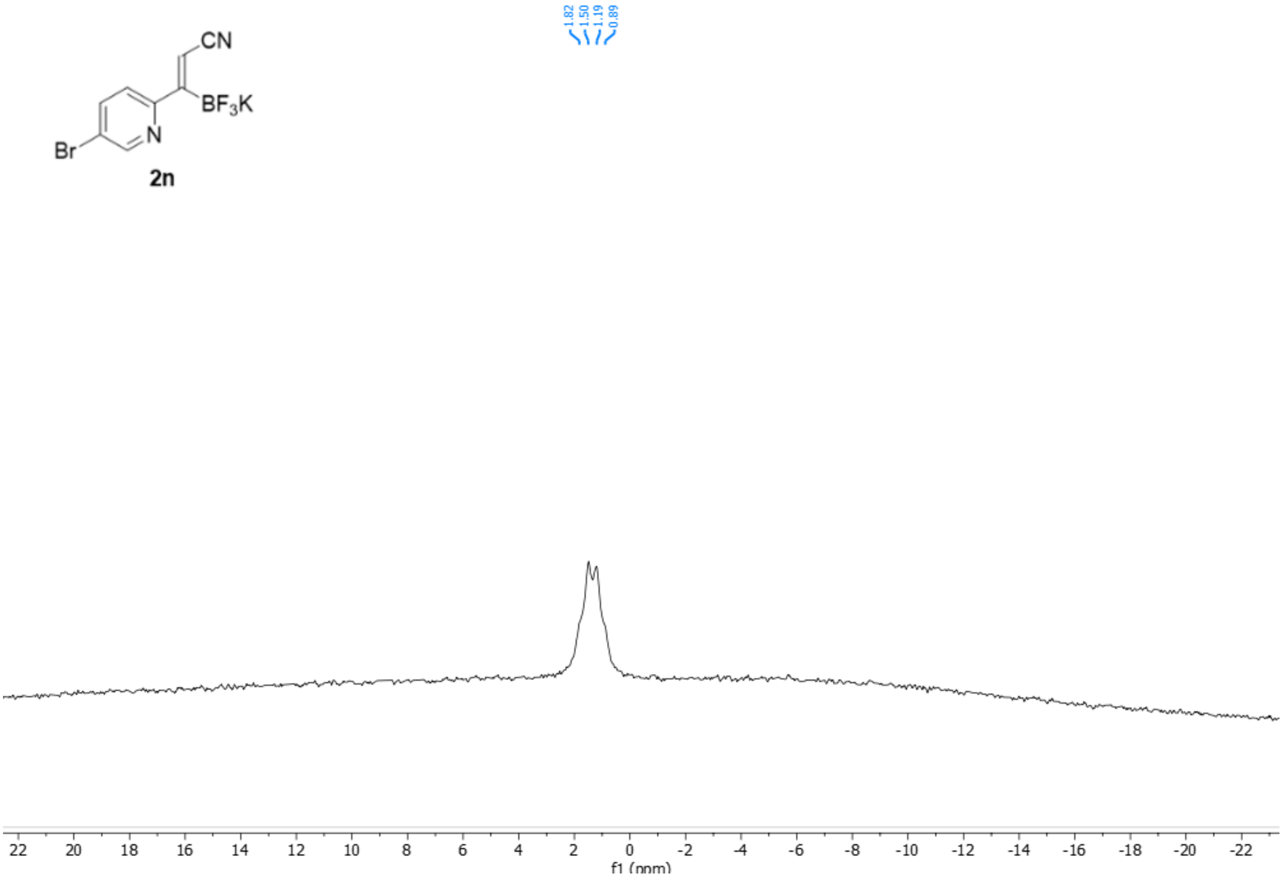

^{19}F-NMR (470 MHz, DMSO-d_6) of **2n**:

^{19}F-NMR

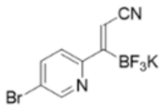

2n

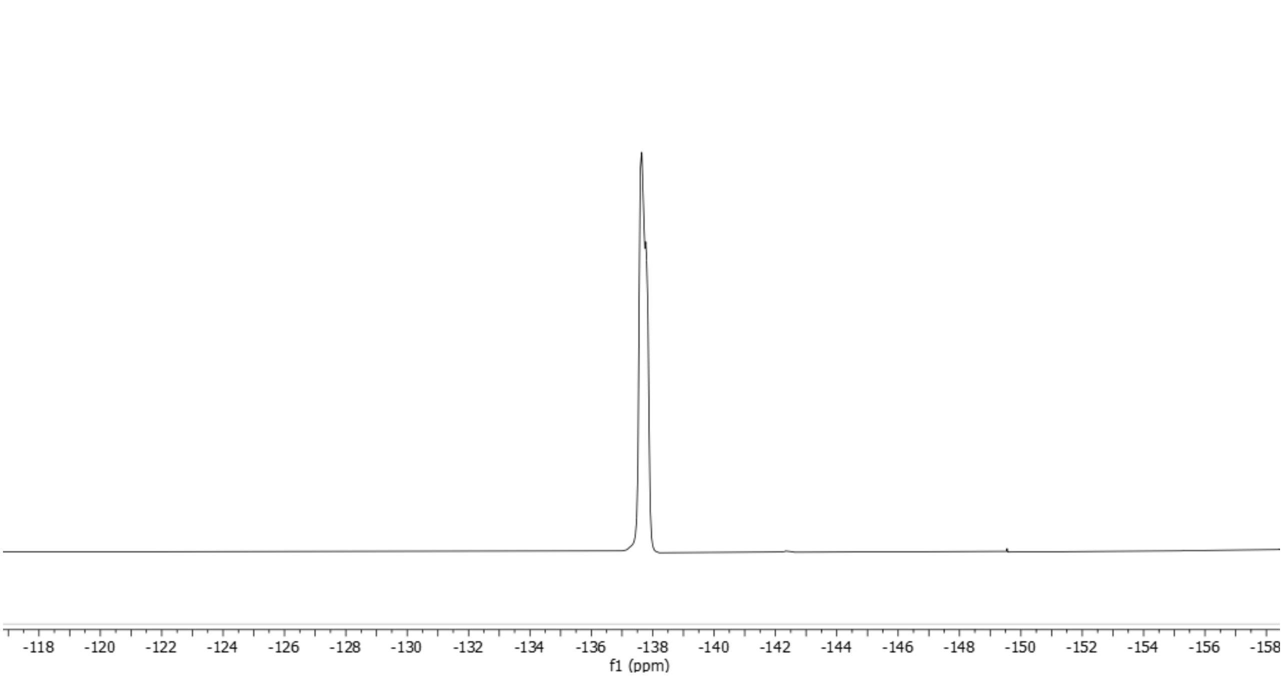

(E)-3-(6-chloropyridin-2-yl)-3-(trifluoro-λ⁴-boraneyl)acrylonitrile, potassium salt (2o)

^{1}H-NMR (500 MHz, DMSO-d_6) of the reaction mixture of **2o**:

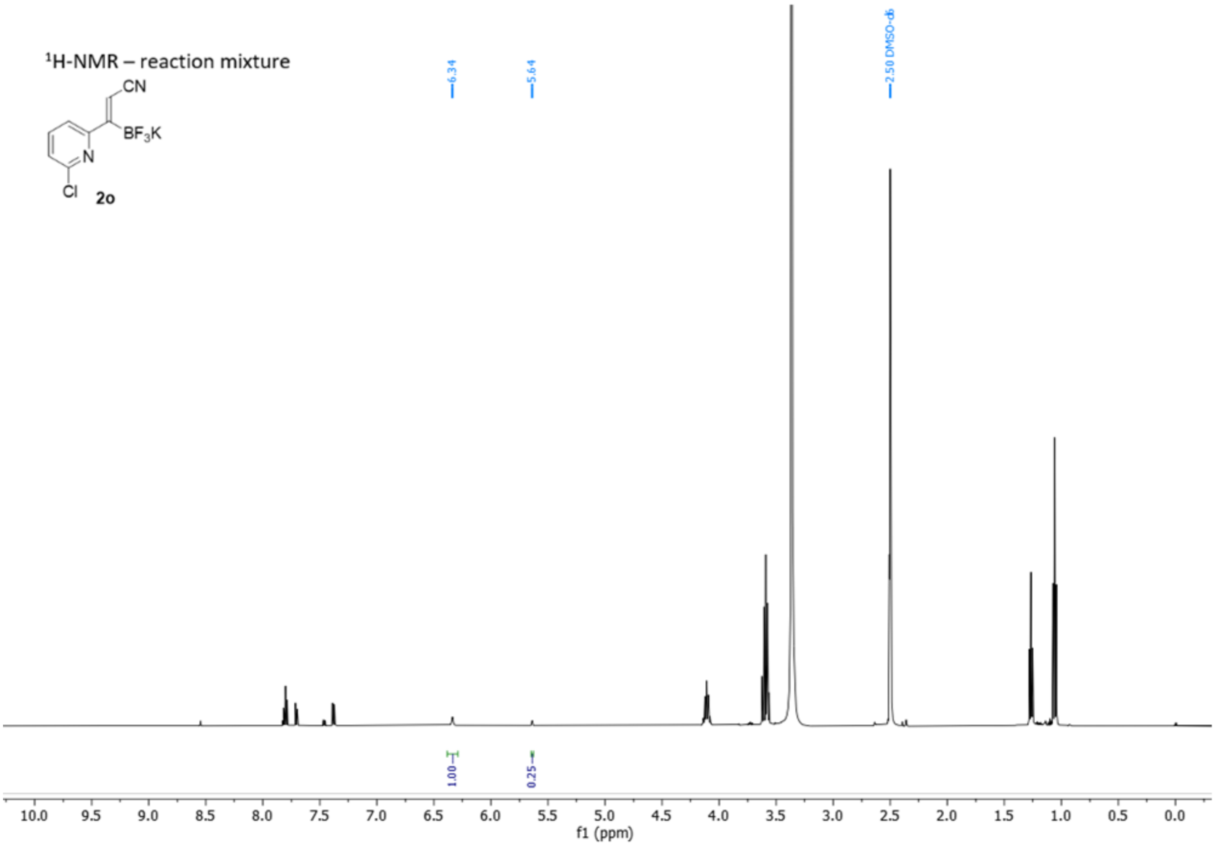

^{1}H-NMR (500 MHz, DMSO-d_6) of **2o**:

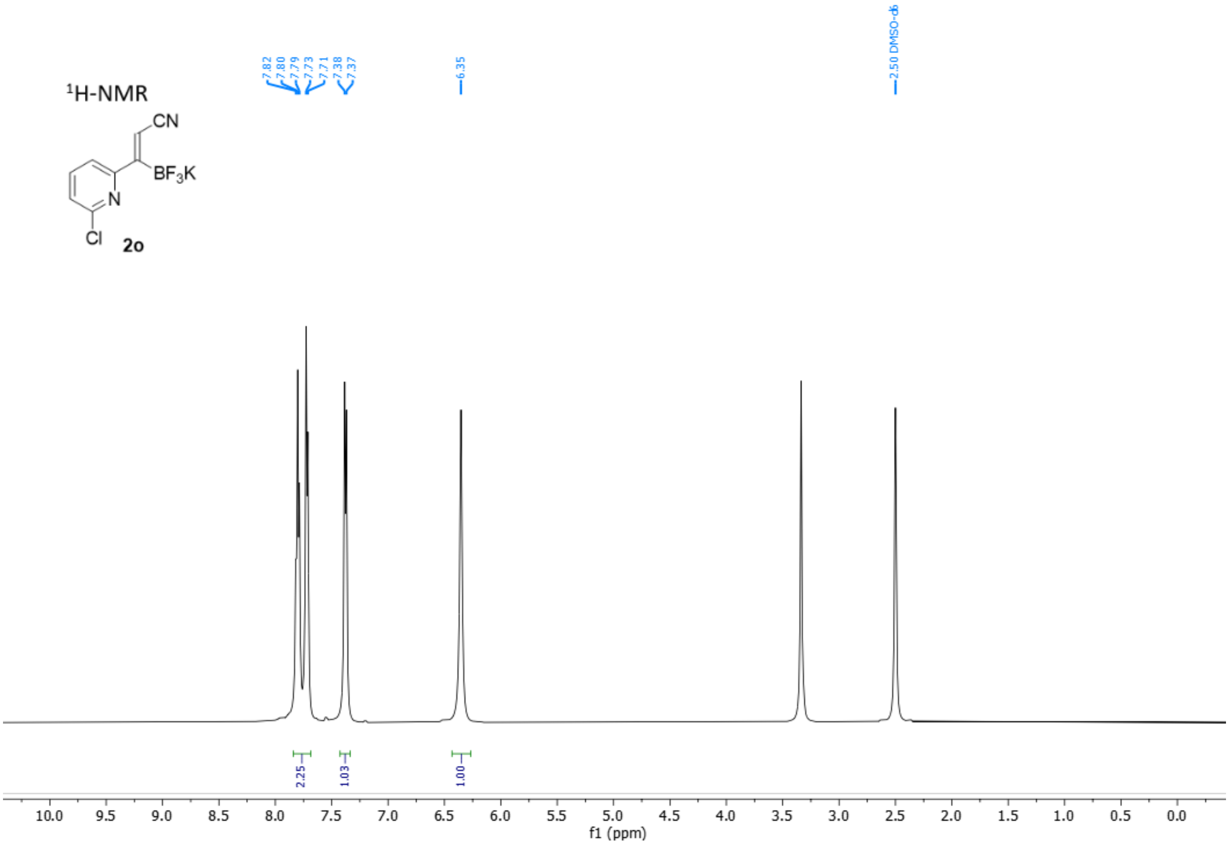

$^{13}C\{^1H\}$-NMR (126 MHz, DMSO-d_6) of **2o**:

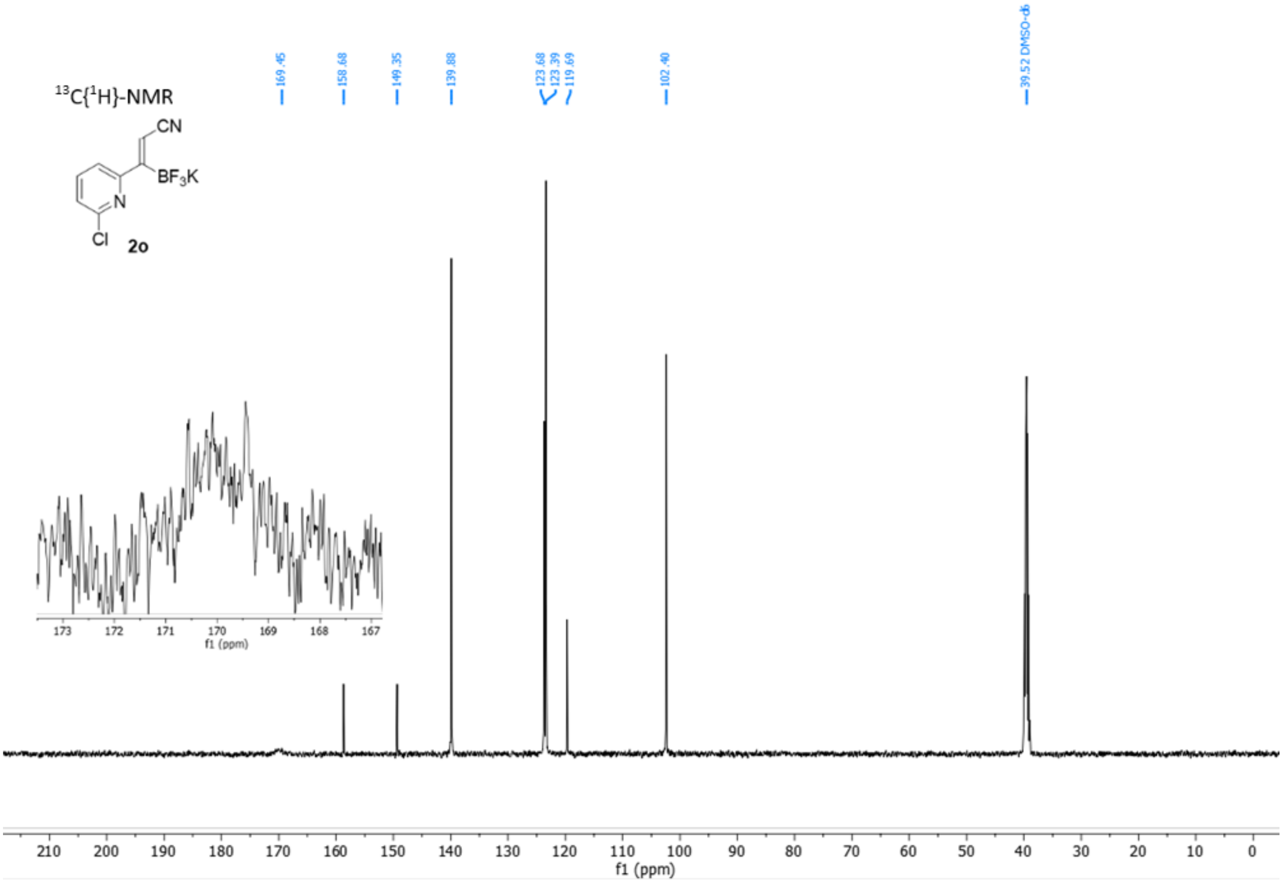

^{11}B-NMR (160 MHz, DMSO-d_6) of **2o**:

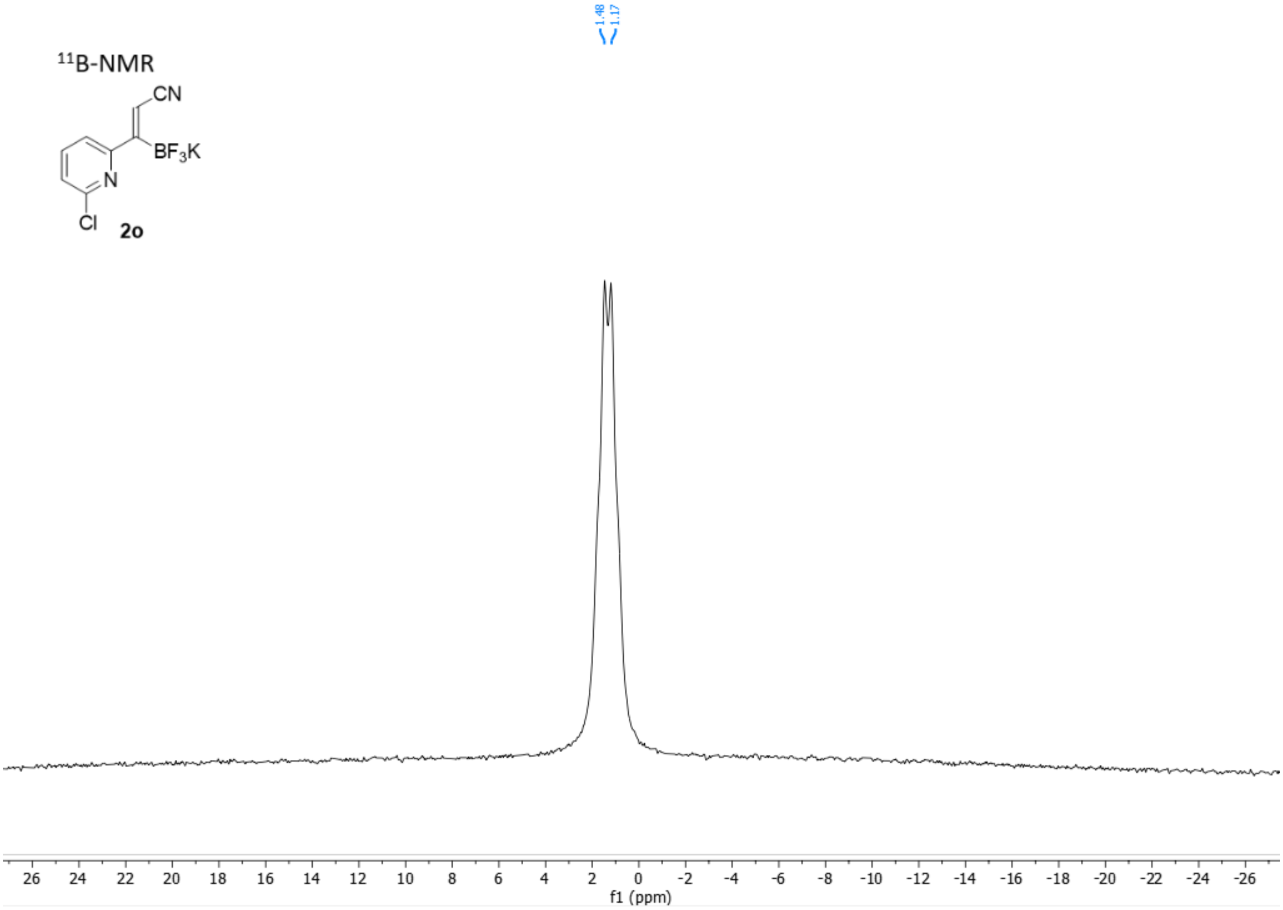

^{19}F-NMR (470 MHz, DMSO-d_6) of **2o**:

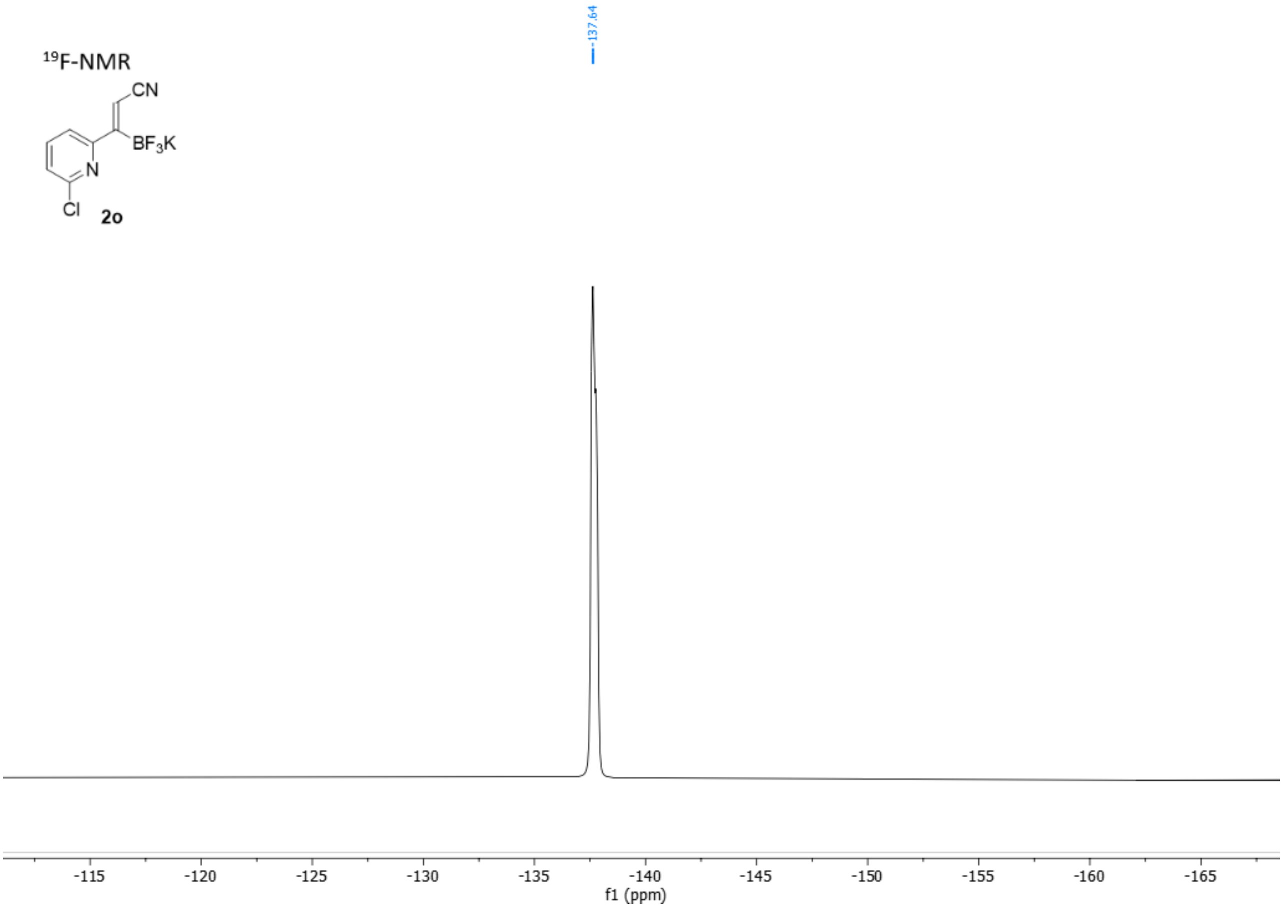

(E)-3-(thiophen-2-yl)-3-(trifluoro-λ[4]-boraneyl)acrylonitrile, potassium salt (2p)

^{1}H-NMR (500 MHz, DMSO-d_6) of the reaction mixture of **2p**:

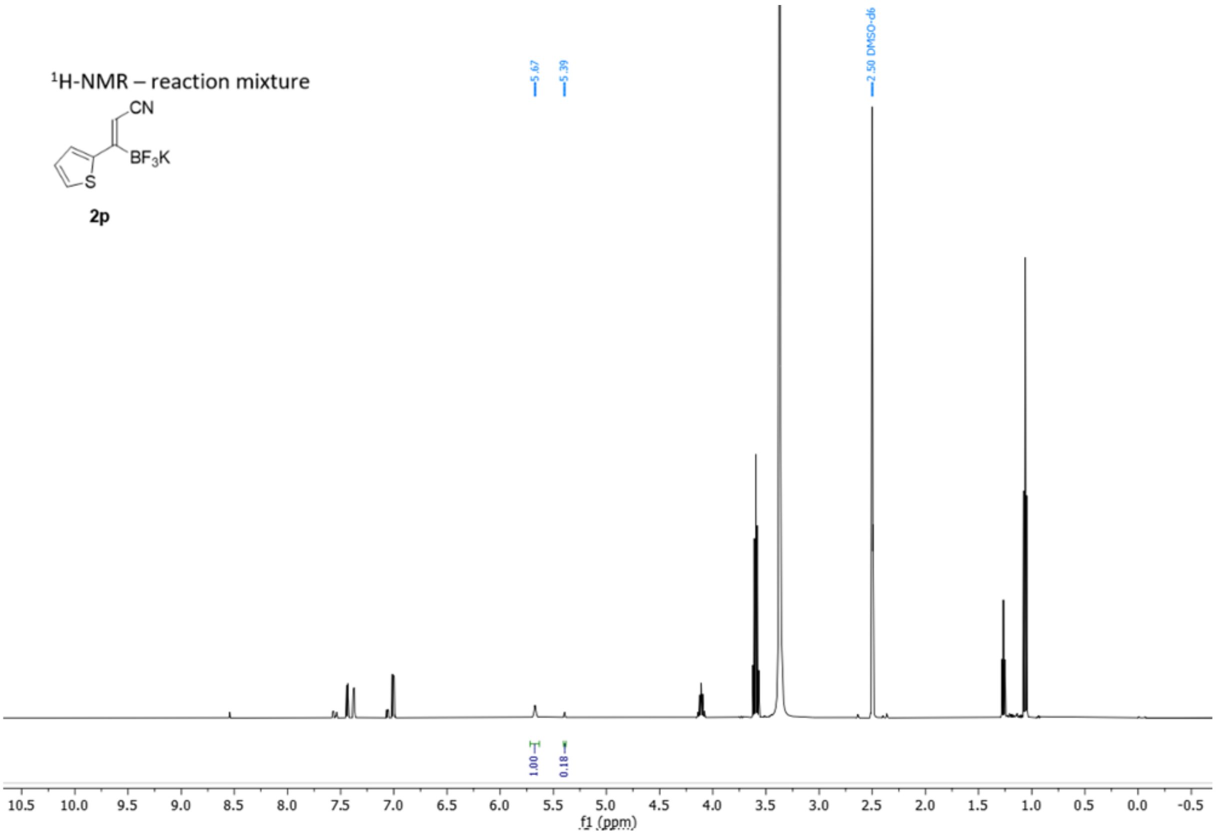

^{1}H-NMR (500 MHz, DMSO-d_6) of **2p**:

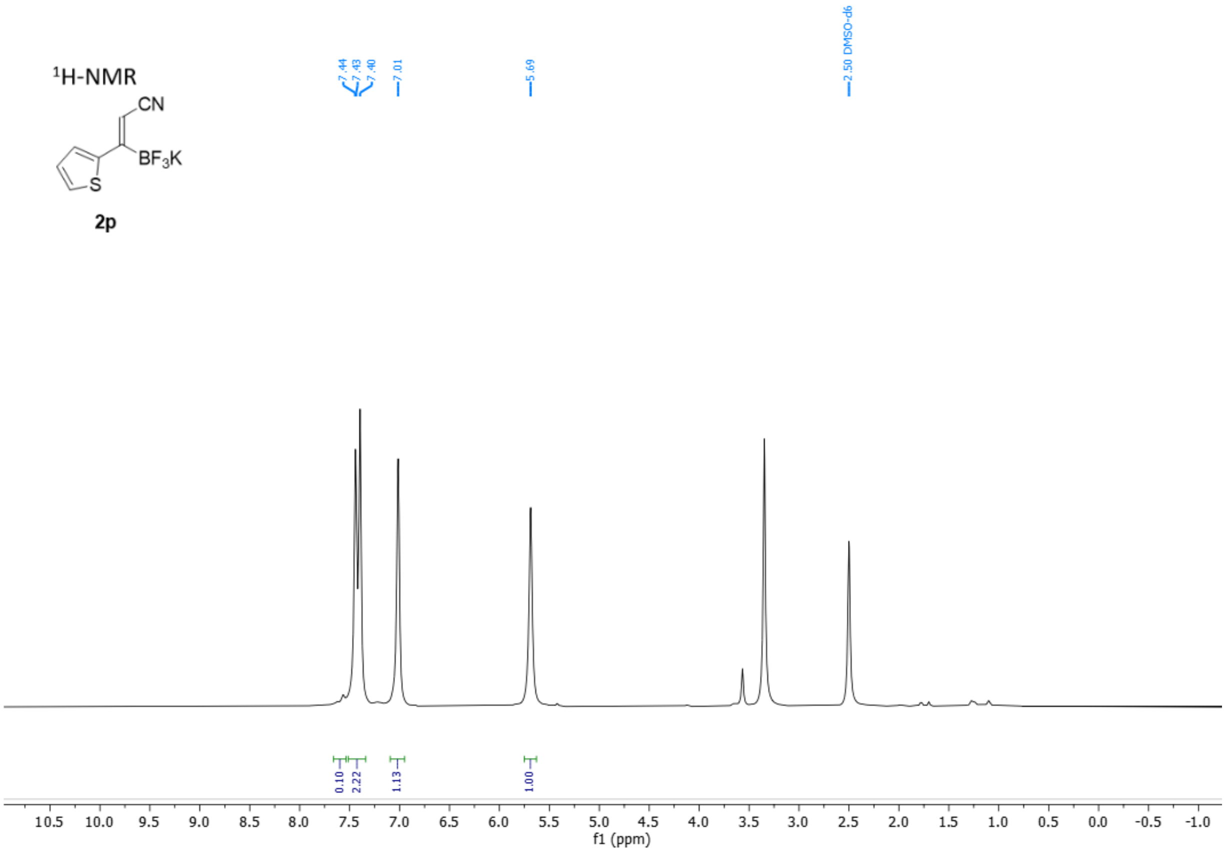

$^{13}C\{^{1}H\}$-NMR (126 MHz, DMSO-d_6) of **2p**:

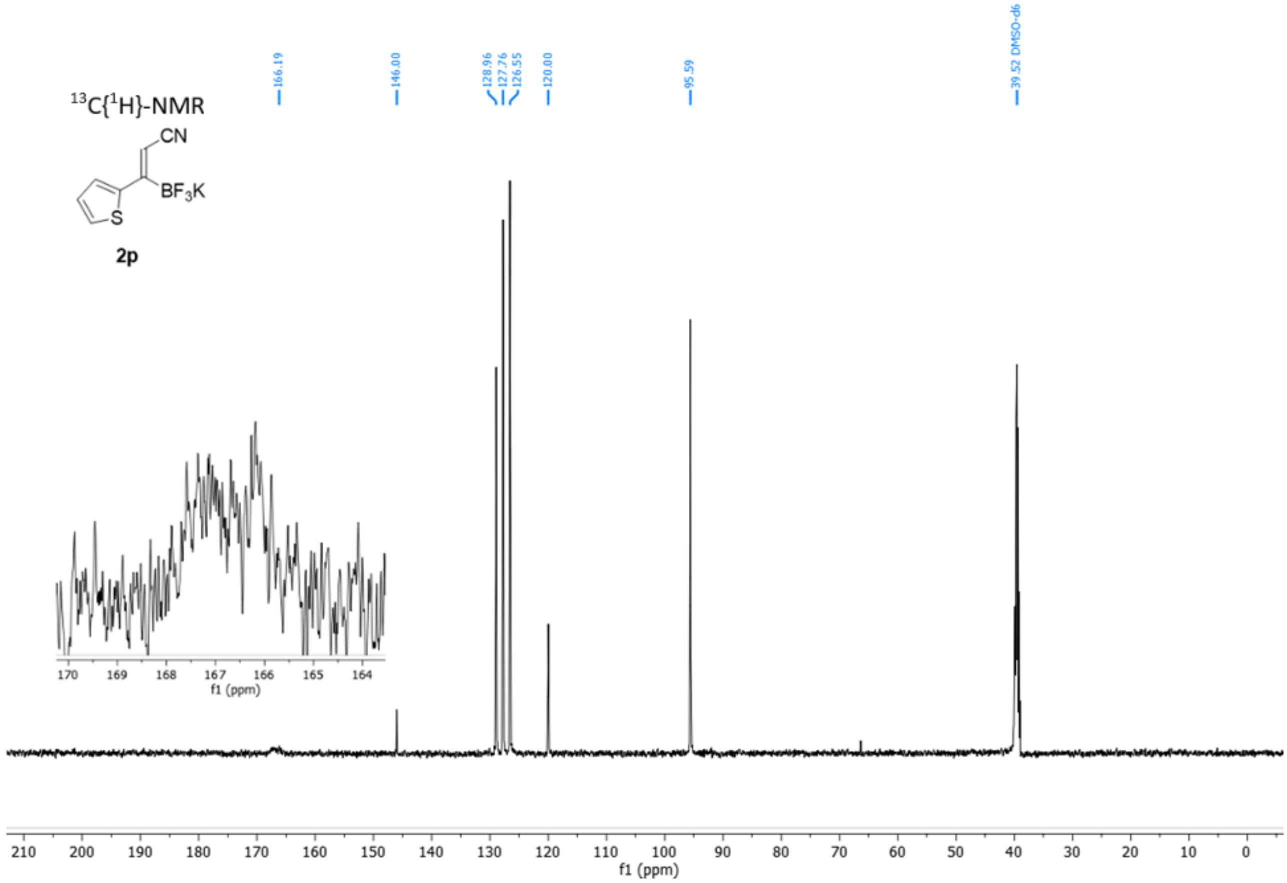

^{11}B-NMR (160 MHz, DMSO-d_6) of **2p**:

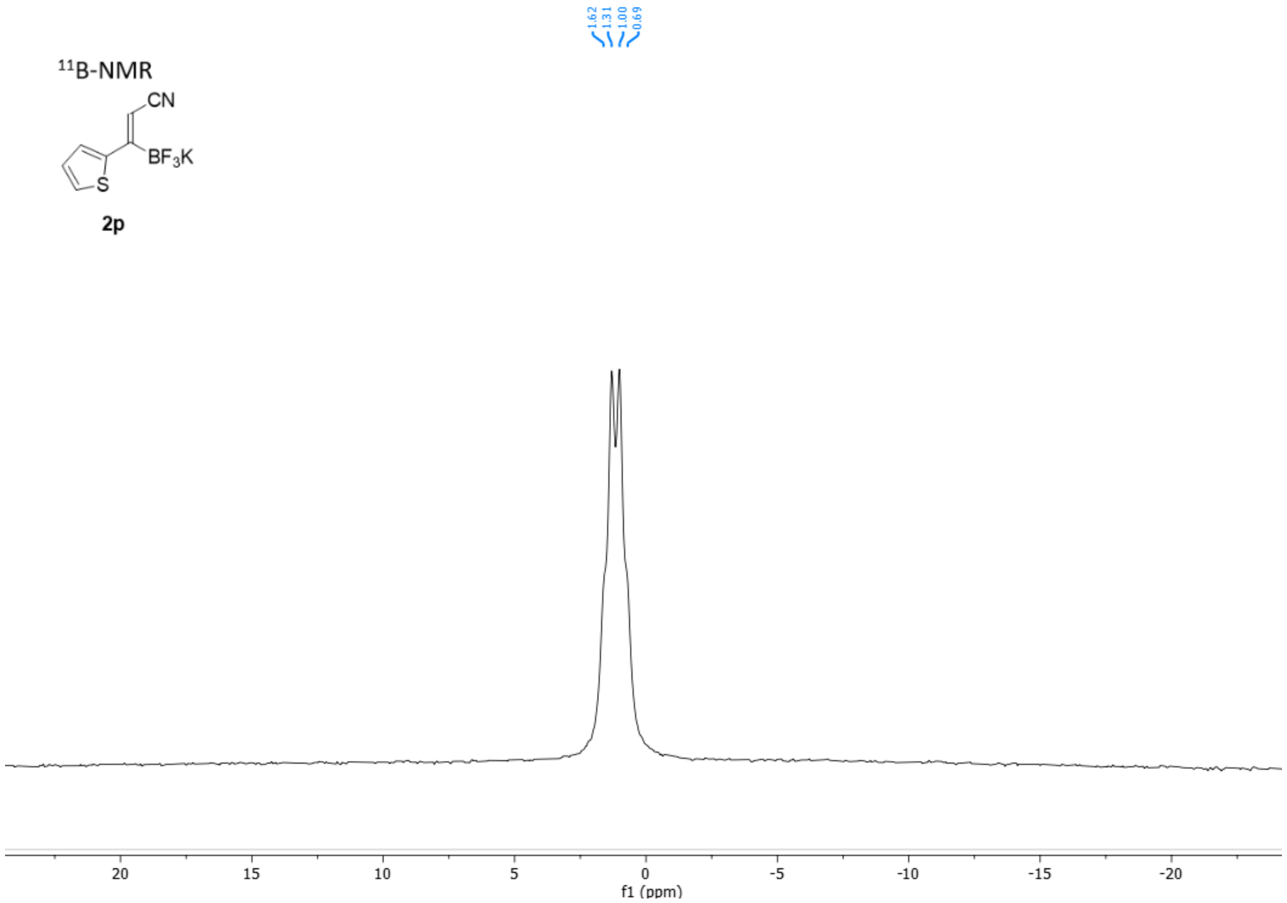

^{19}F-NMR (470 MHz, DMSO-d_6) of **2p**:

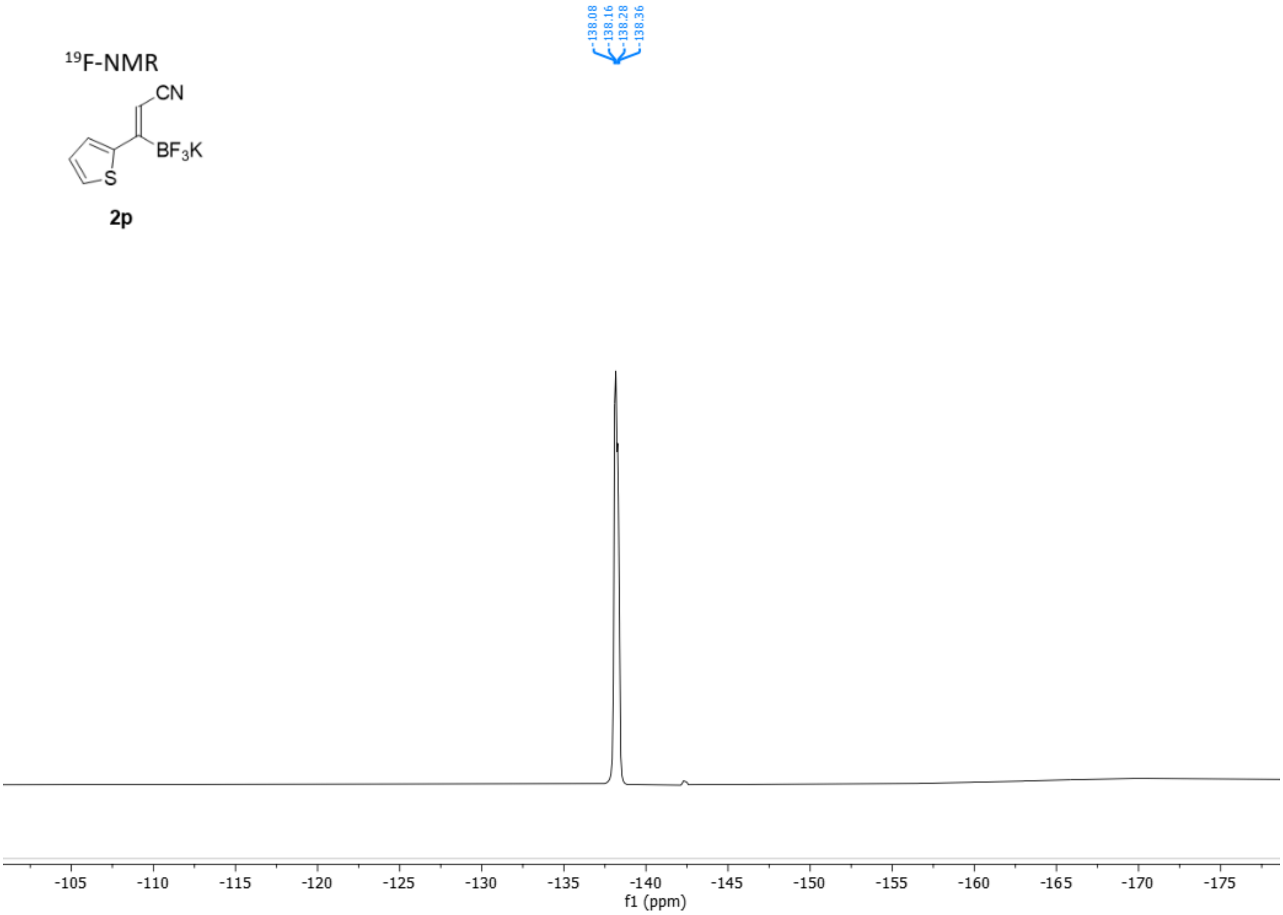

(E)-3-(thiophen-3-yl)-3-(trifluoro-λ⁴-boraneyl)acrylonitrile, potassium salt (2q)

^{1}H-NMR (500 MHz, DMSO-d_6) of the reaction mixture of **2q**:

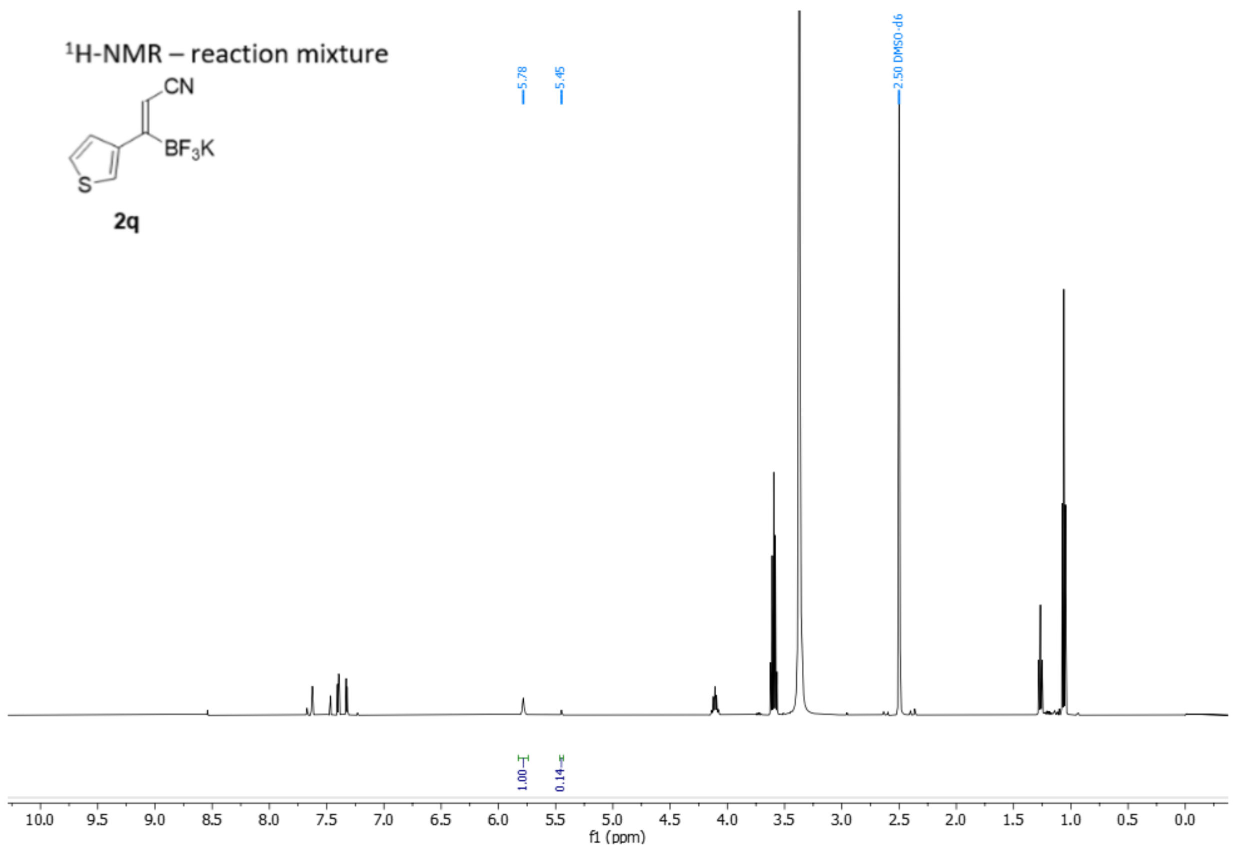

^{1}H-NMR (500 MHz, DMSO-d_6) of **2q**:

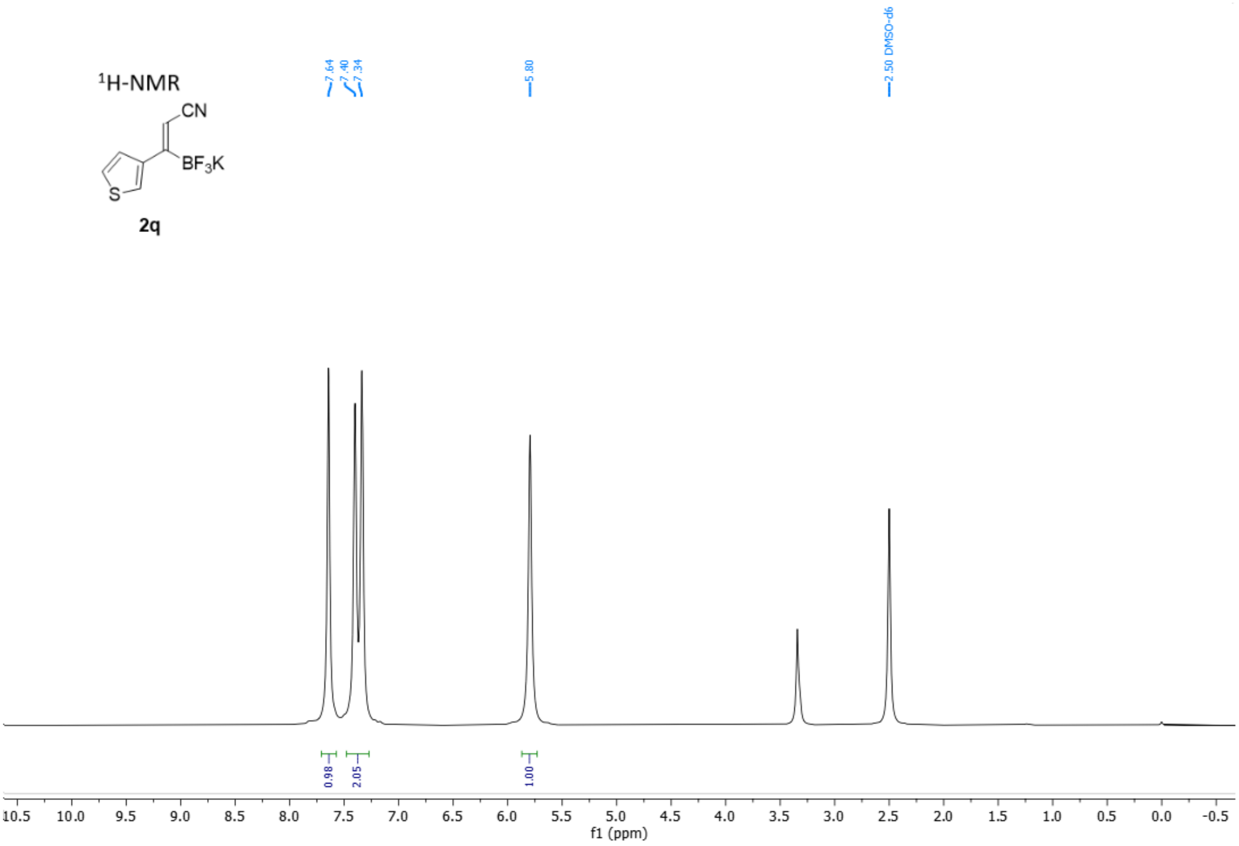

$^{13}C\{^1H\}$-NMR (126 MHz, DMSO-d_6) of **2q**:

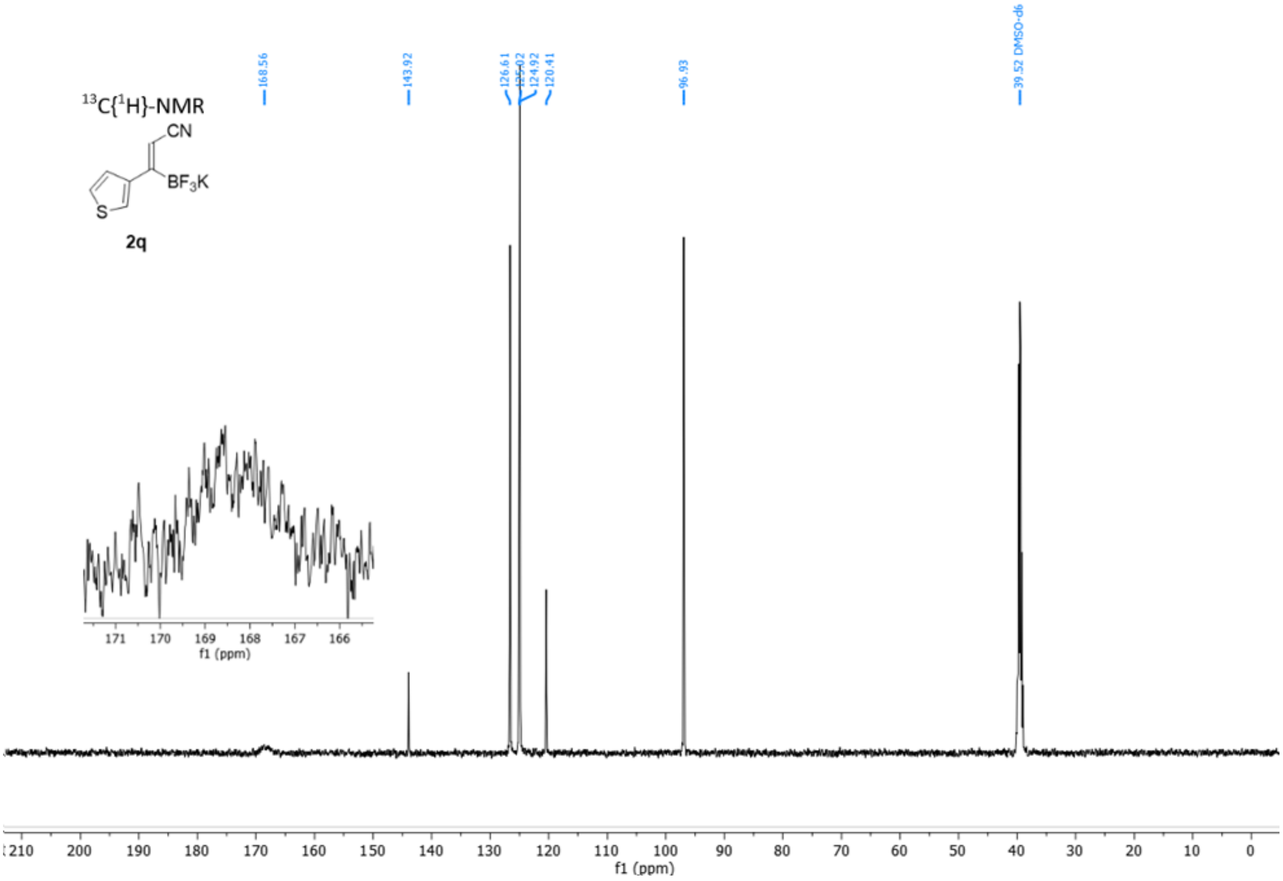

^{11}B-NMR (160 MHz, DMSO-d_6) of **2q**:

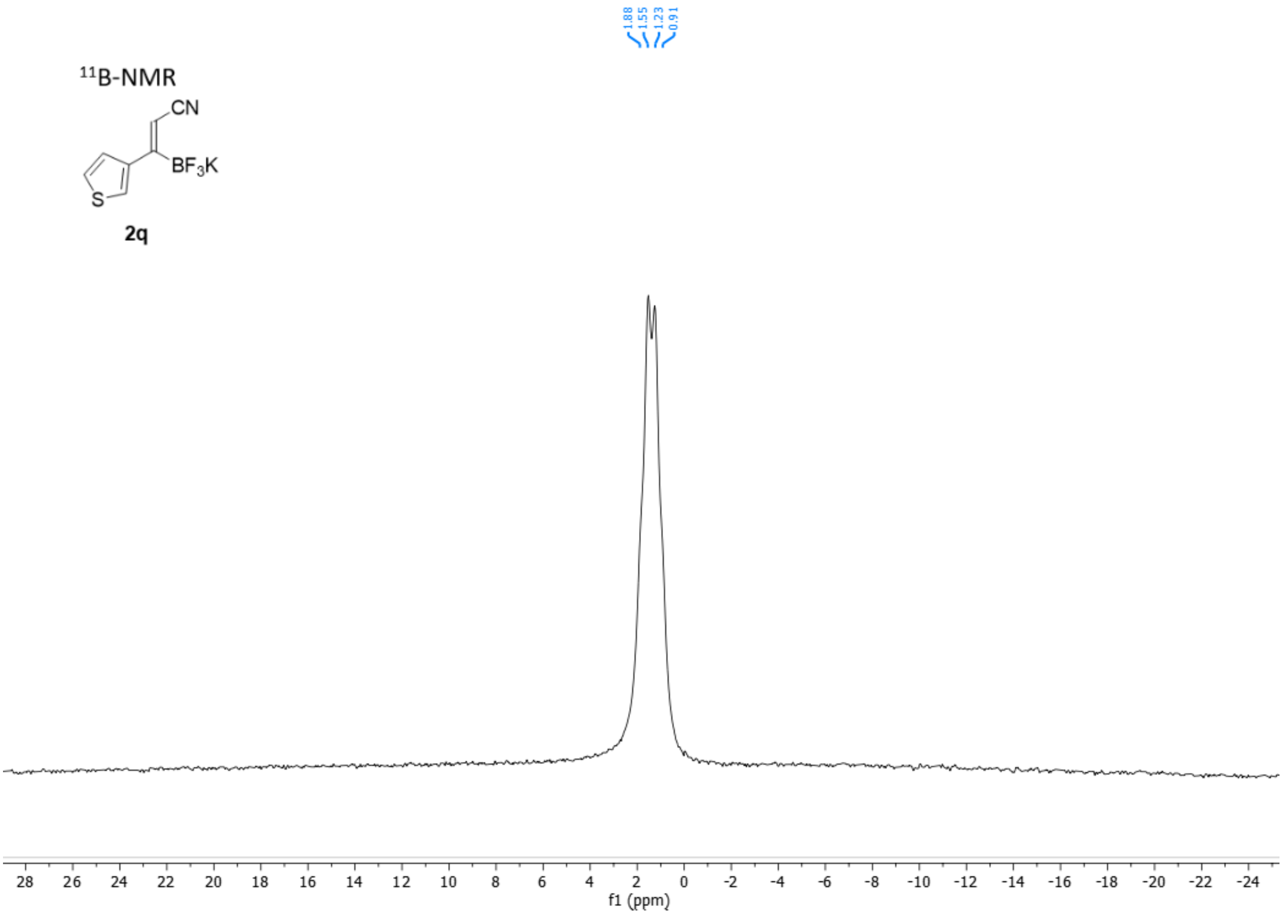

^{19}F-NMR (470 MHz, DMSO-d_6) of **2q**:

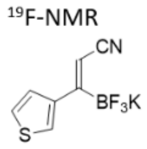

2q

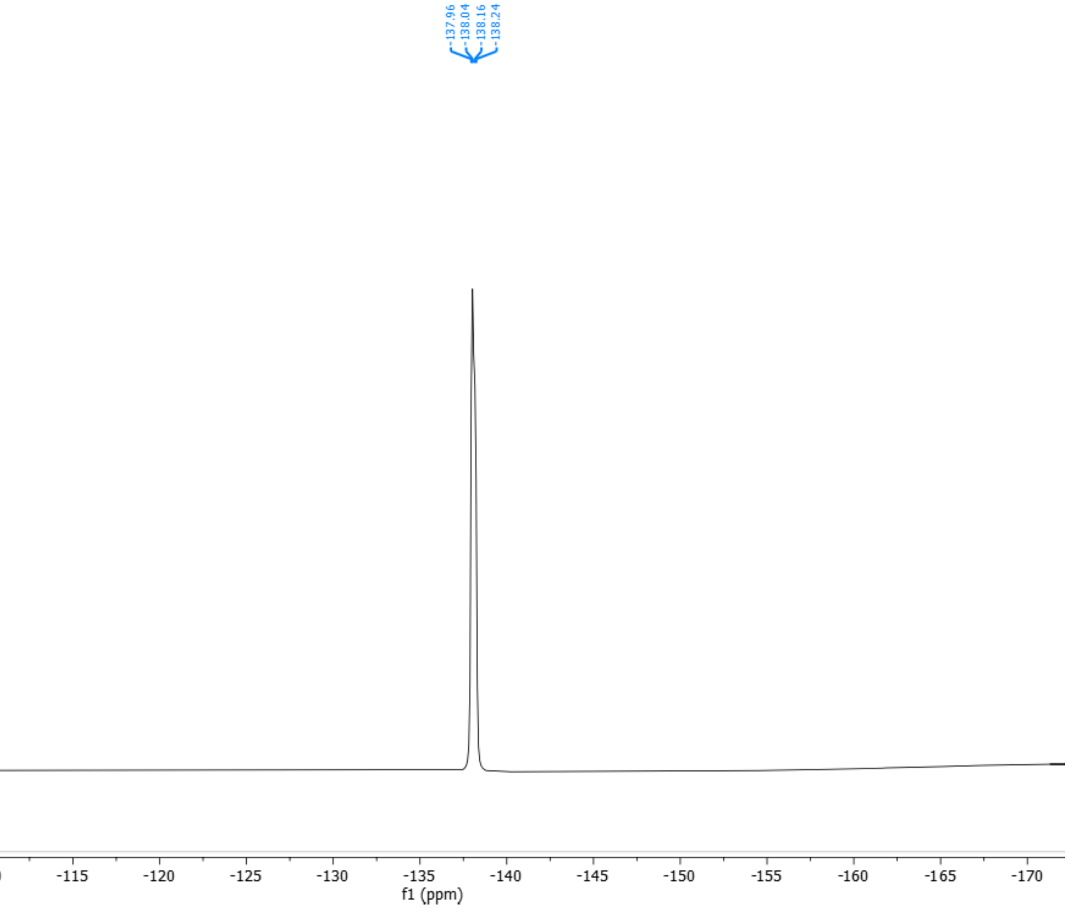

(*E*)-3-(furan-3-yl)-3-(trifluoro-λ⁴-boraneyl)acrylonitrile, potassium salt (2r)

^{1}H-NMR (500 MHz, DMSO-d_6) of the reaction mixture of **2r** before the isolation according to the procedure A:

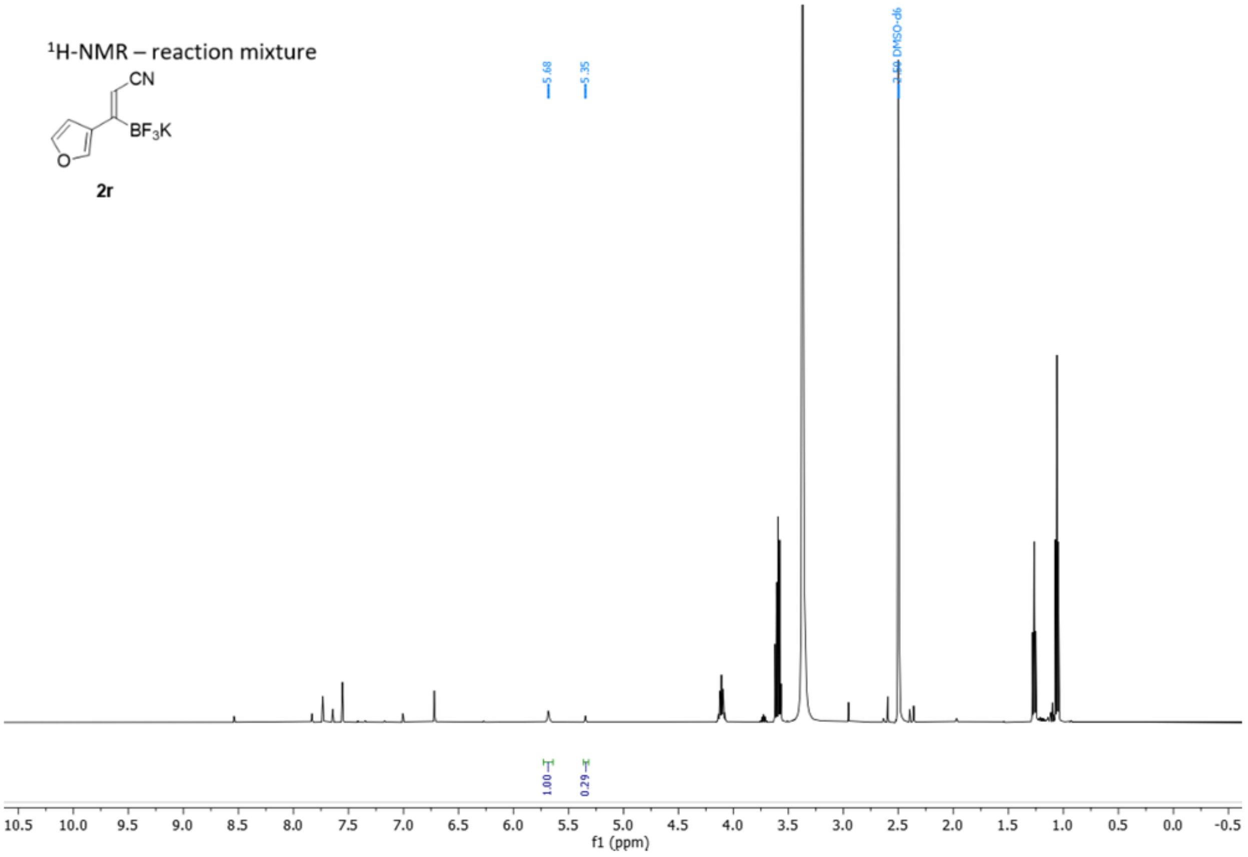

^{1}H-NMR (500 MHz, DMSO-d_6) of the reaction mixture of **2r** before the isolation according to the procedure B:

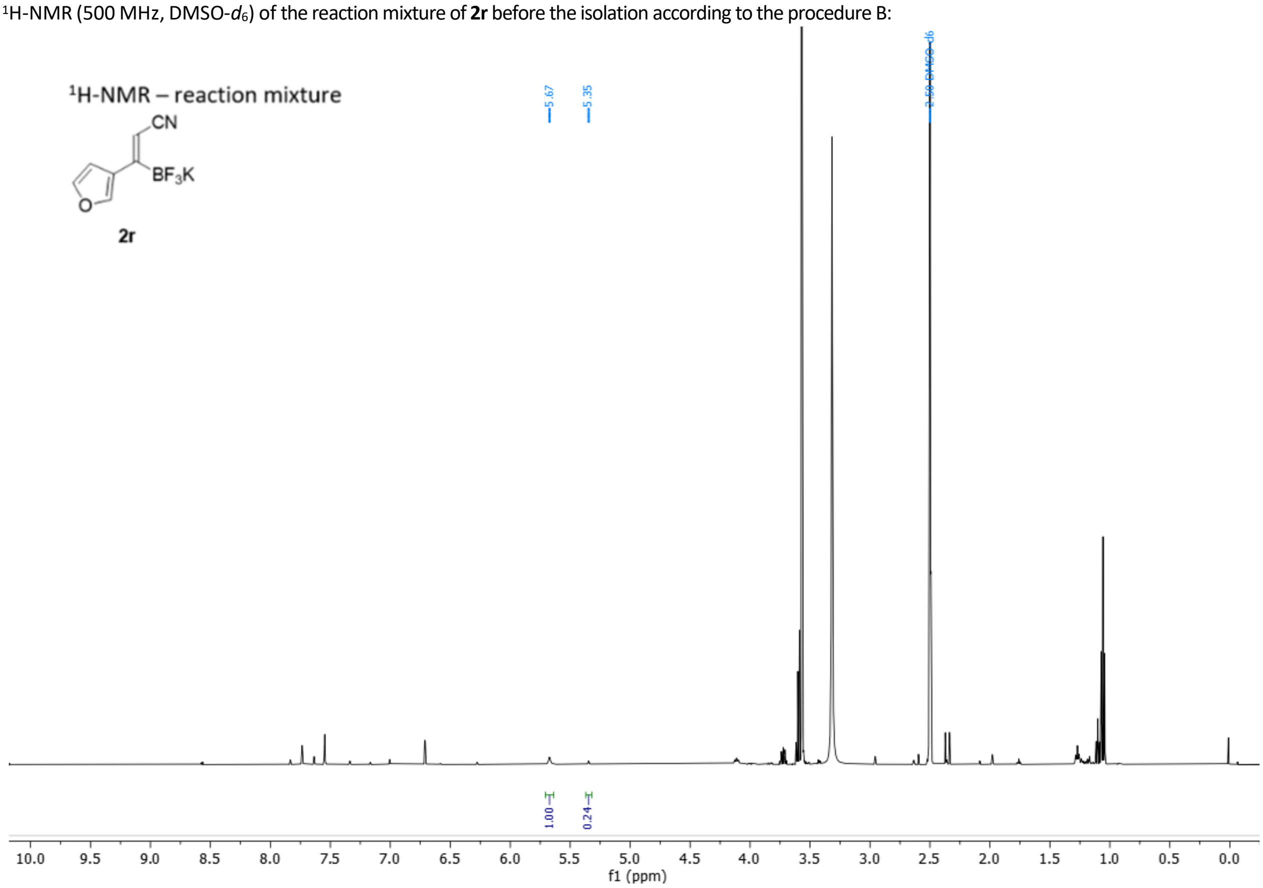

^{1}H-NMR (500 MHz, DMSO-d_6) of **2r**:

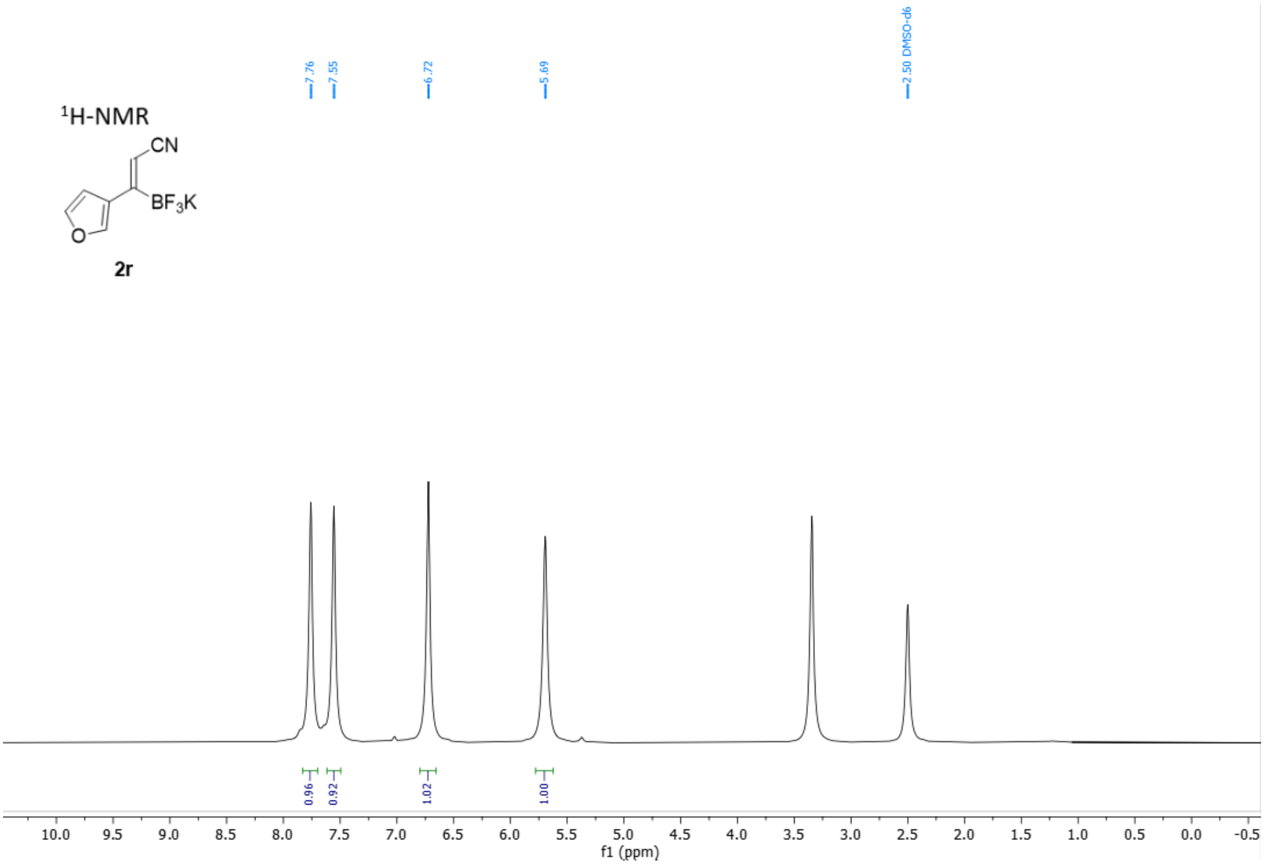

^{13}C{^{1}H}-NMR (126 MHz, DMSO-d_6) of **2r**:

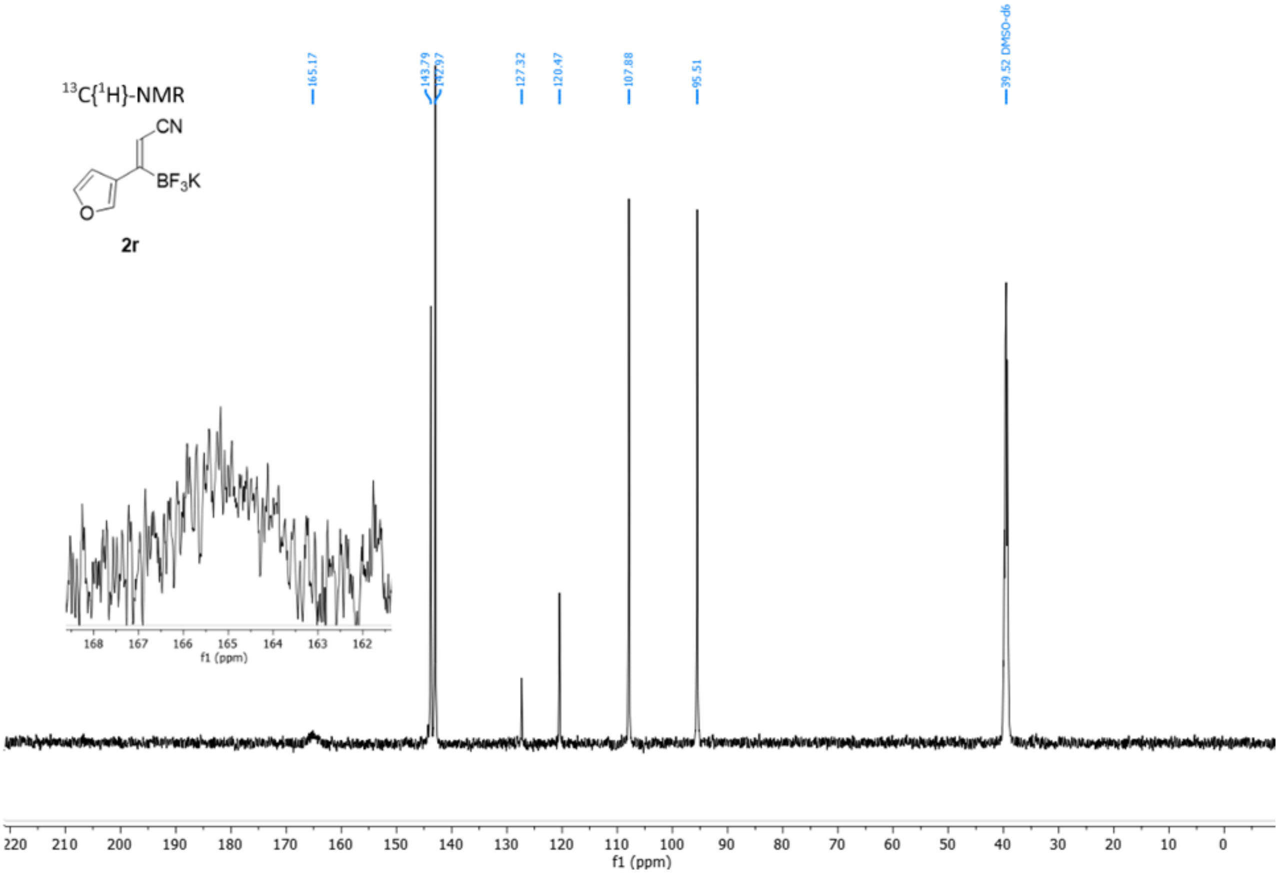

^{11}B-NMR (160 MHz, DMSO-d_6) of **2r**:

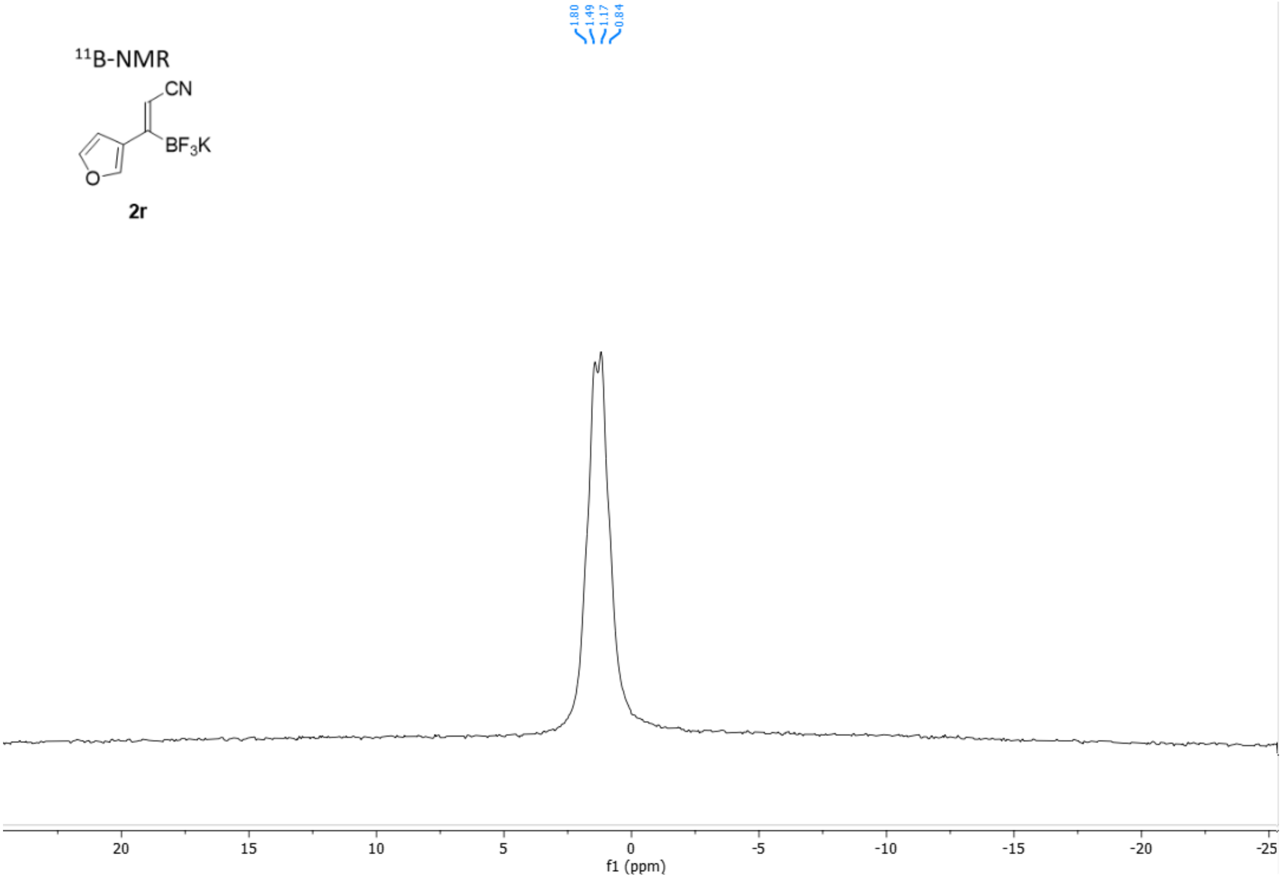

^{19}F-NMR (470 MHz, DMSO-d_6) of **2r**:

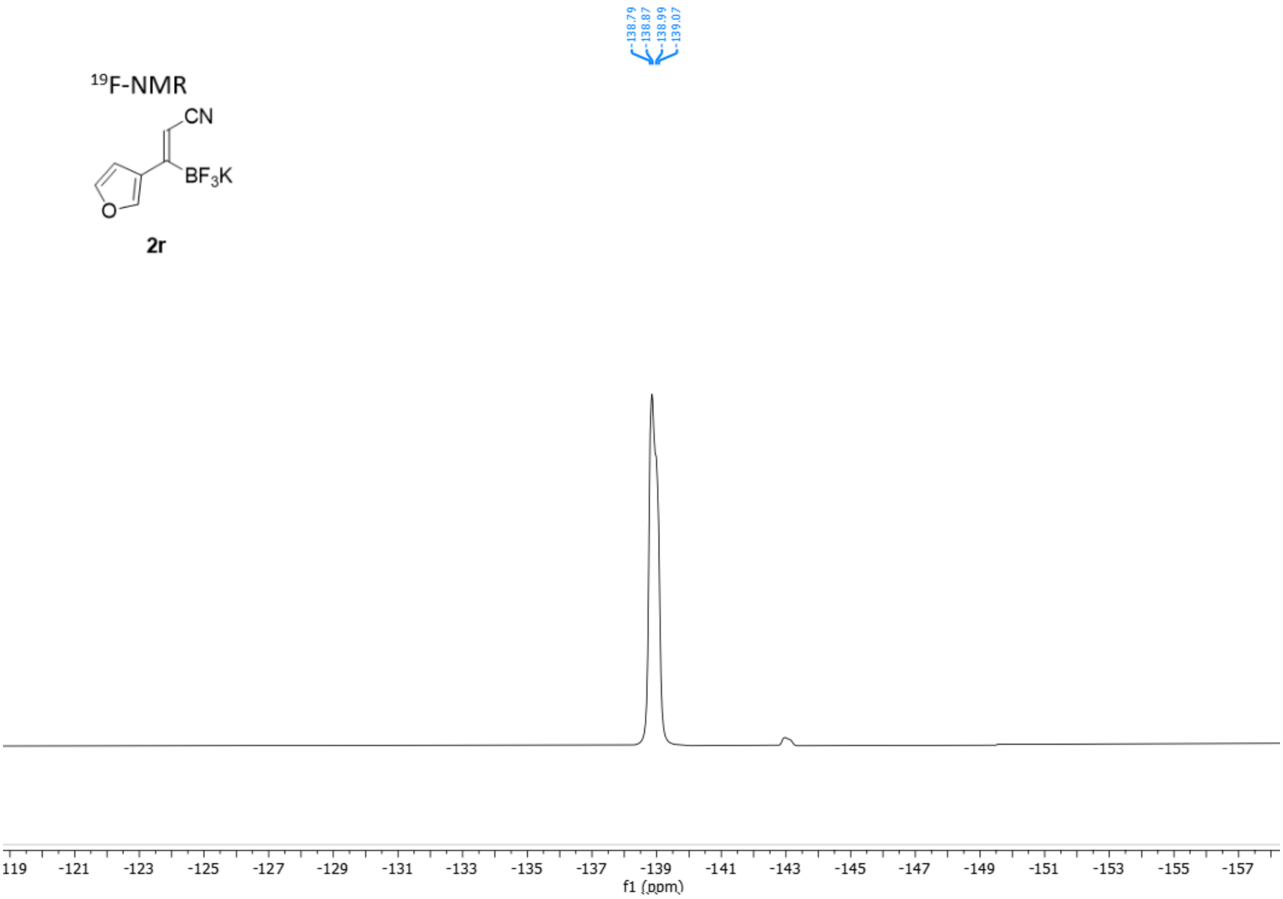

(*E*)-3-(trifluoro-λ^4-boraneyl)pent-2-enenitrile, potassium salt (2s)

^{1}H-NMR (500 MHz, DMSO-d_6) of the reaction mixture of **2s**:

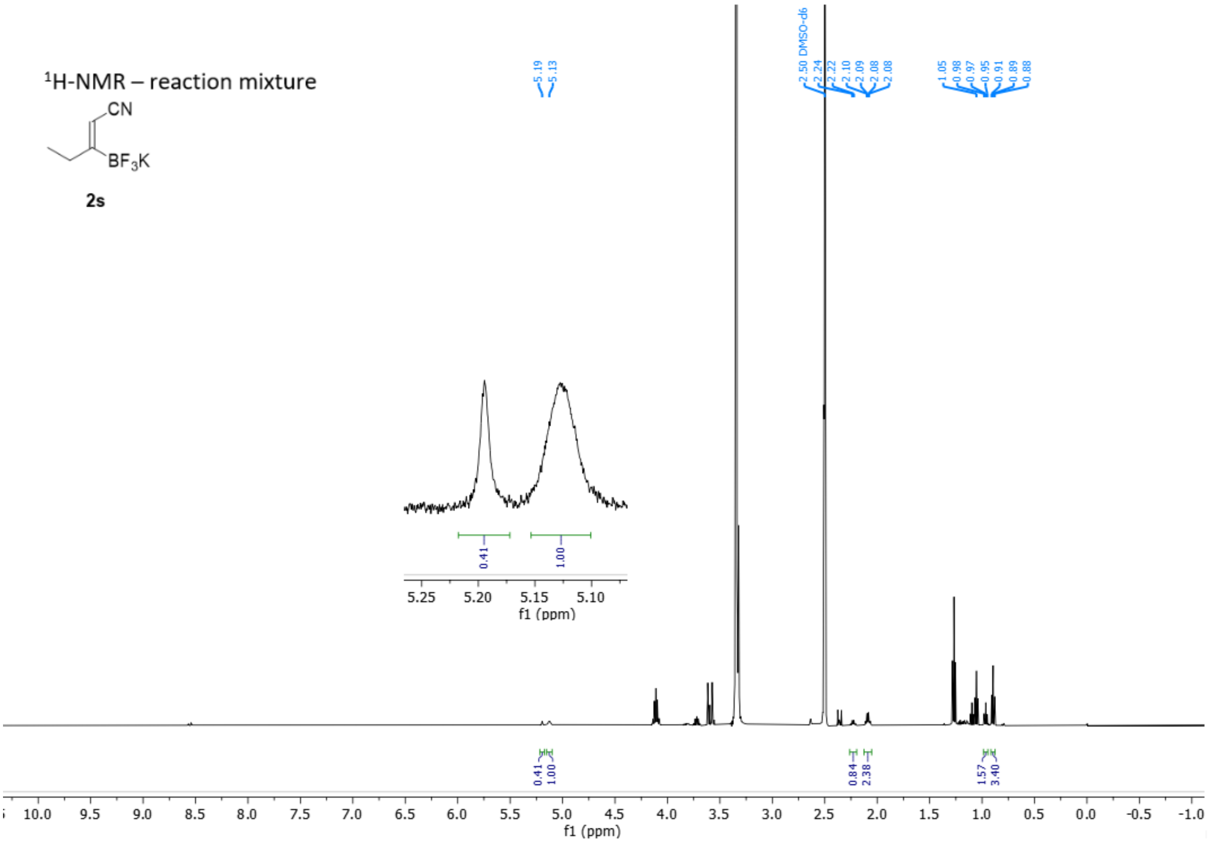

^{1}H-NMR (500 MHz, DMSO-d_6) of **2s**:

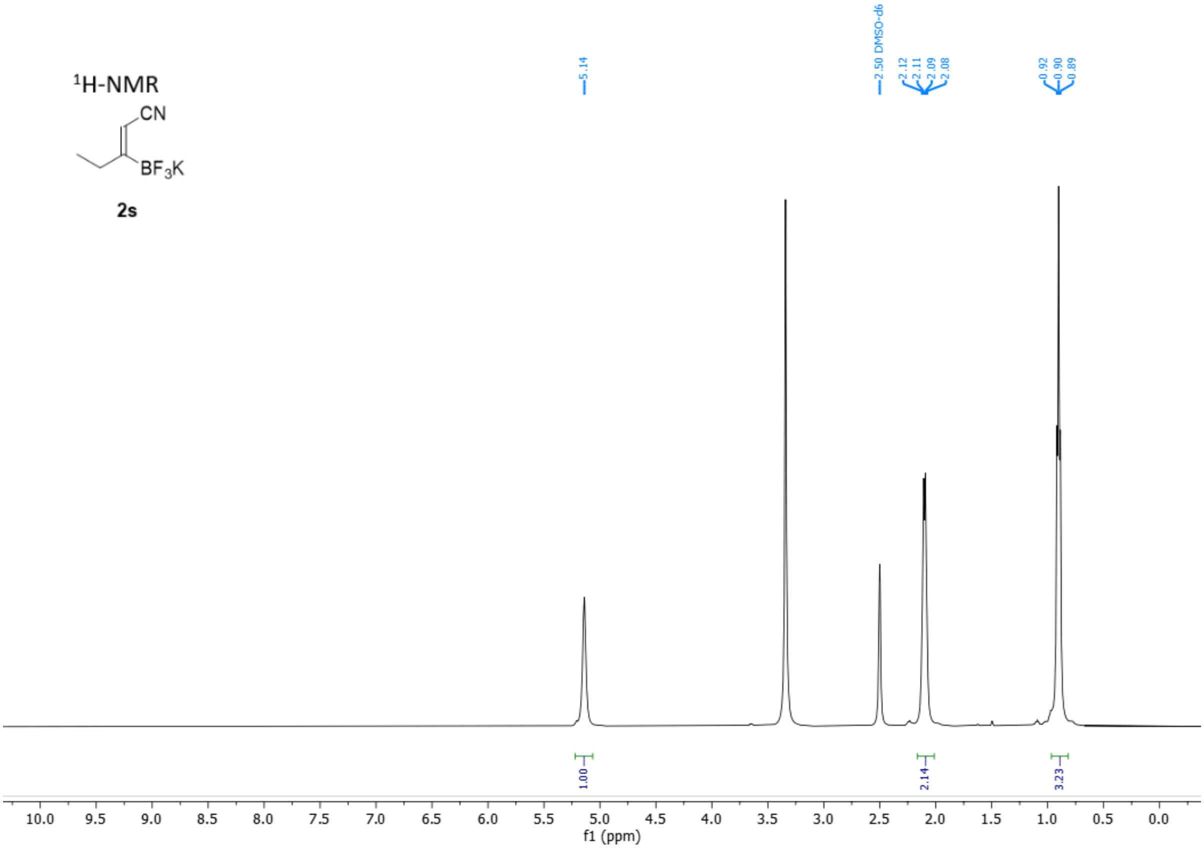

$^{13}C\{^1H\}$-NMR (126 MHz, DMSO-d_6) of **2s**:

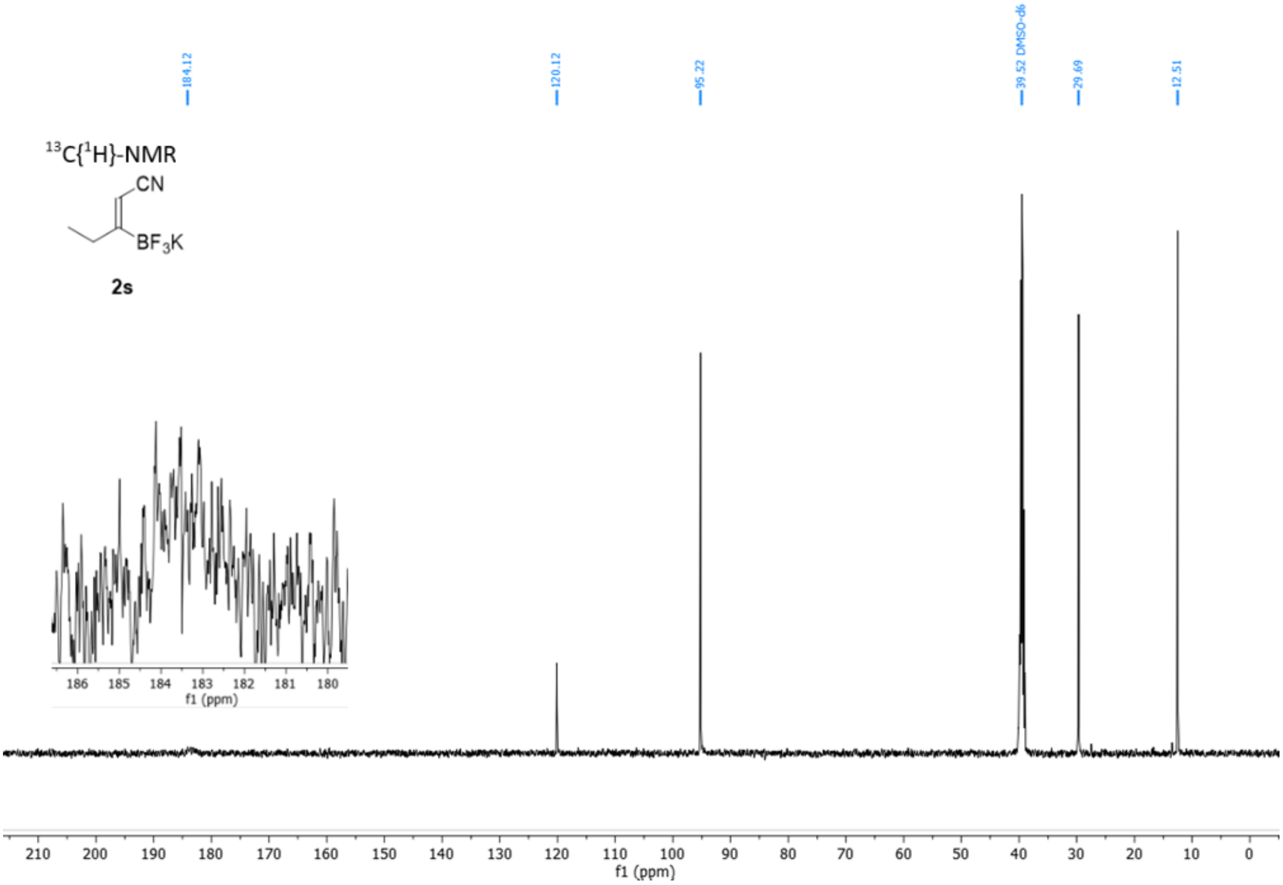

^{11}B-NMR (160 MHz, DMSO-d_6) of **2s**:

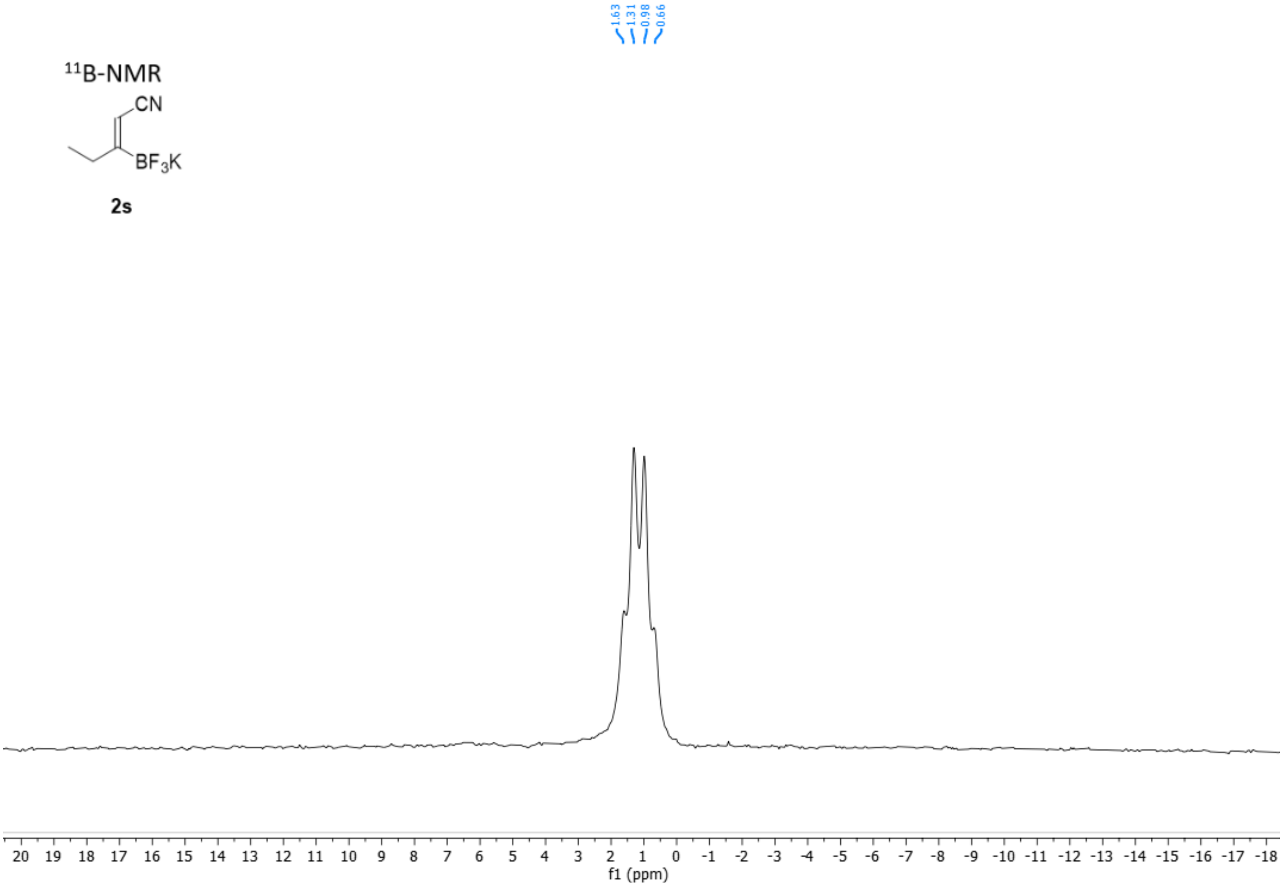

^{19}F-NMR (470 MHz, DMSO-d_6) of **2s**:

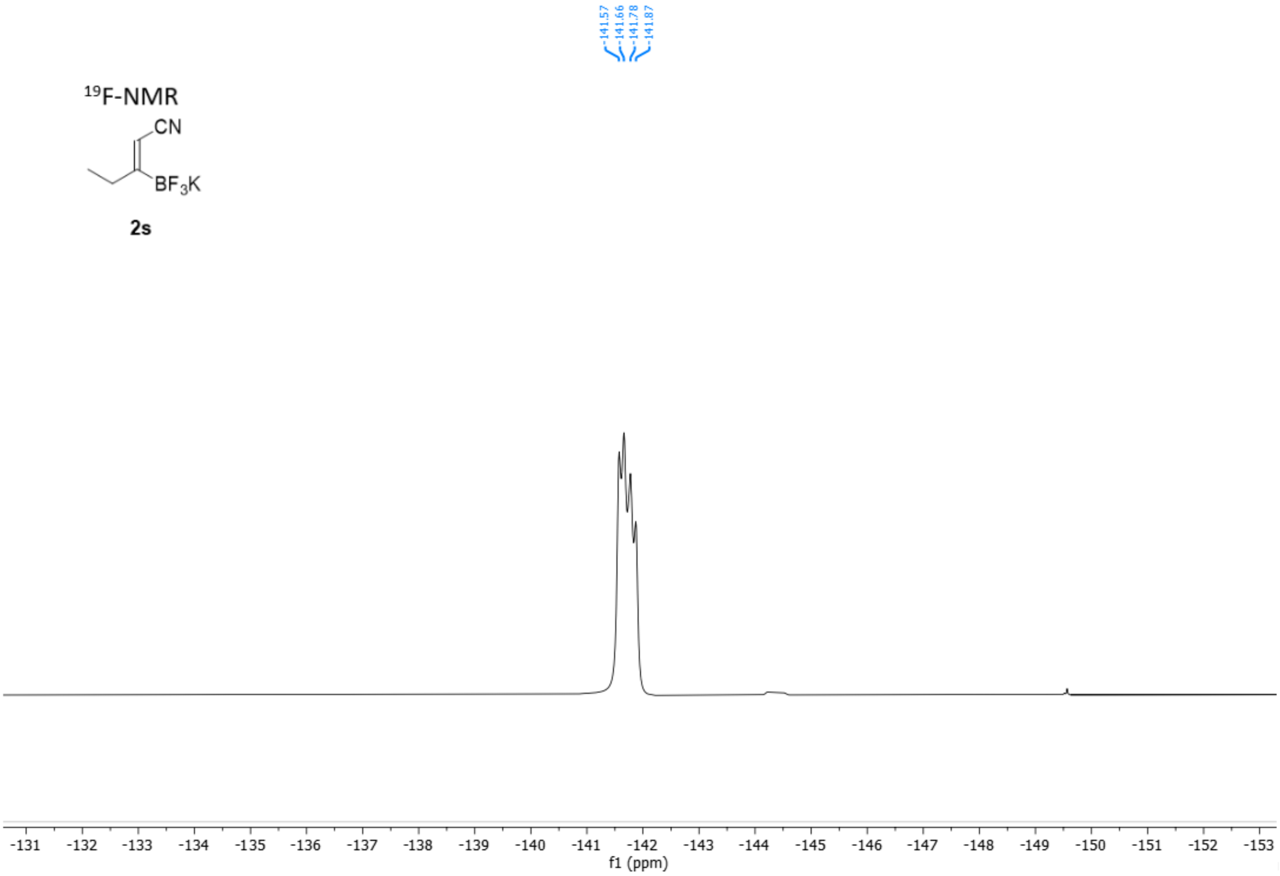

19F-NMR
CN
BF3K
-141.57
-141.66
-141.78
-141.87
-131 -132 -133 -134 -135 -136 -137 -138 -139 -140 -141 -142 -143 -144 -145 -146 -147 -148 -149 -150 -151 -152 -153
f1 (ppm)

2s

(E)-5-methyl-3-(trifluoro-λ⁴-boraneyl)hex-2-enenitrile, potassium salt (2t)

^{1}H-NMR (500 MHz, DMSO-d_6) of the reaction mixture of **2t**:

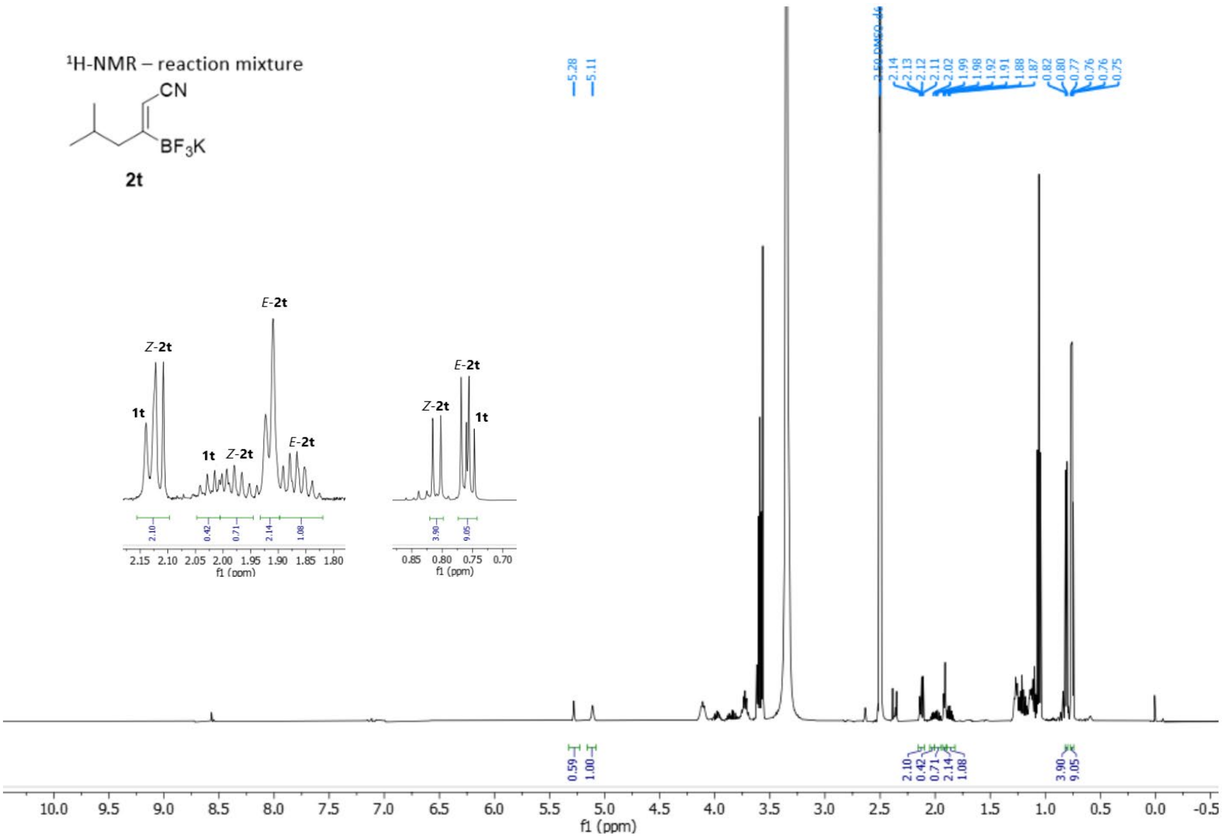

^{1}H-NMR (500 MHz, DMSO-d_6) of **2t**:

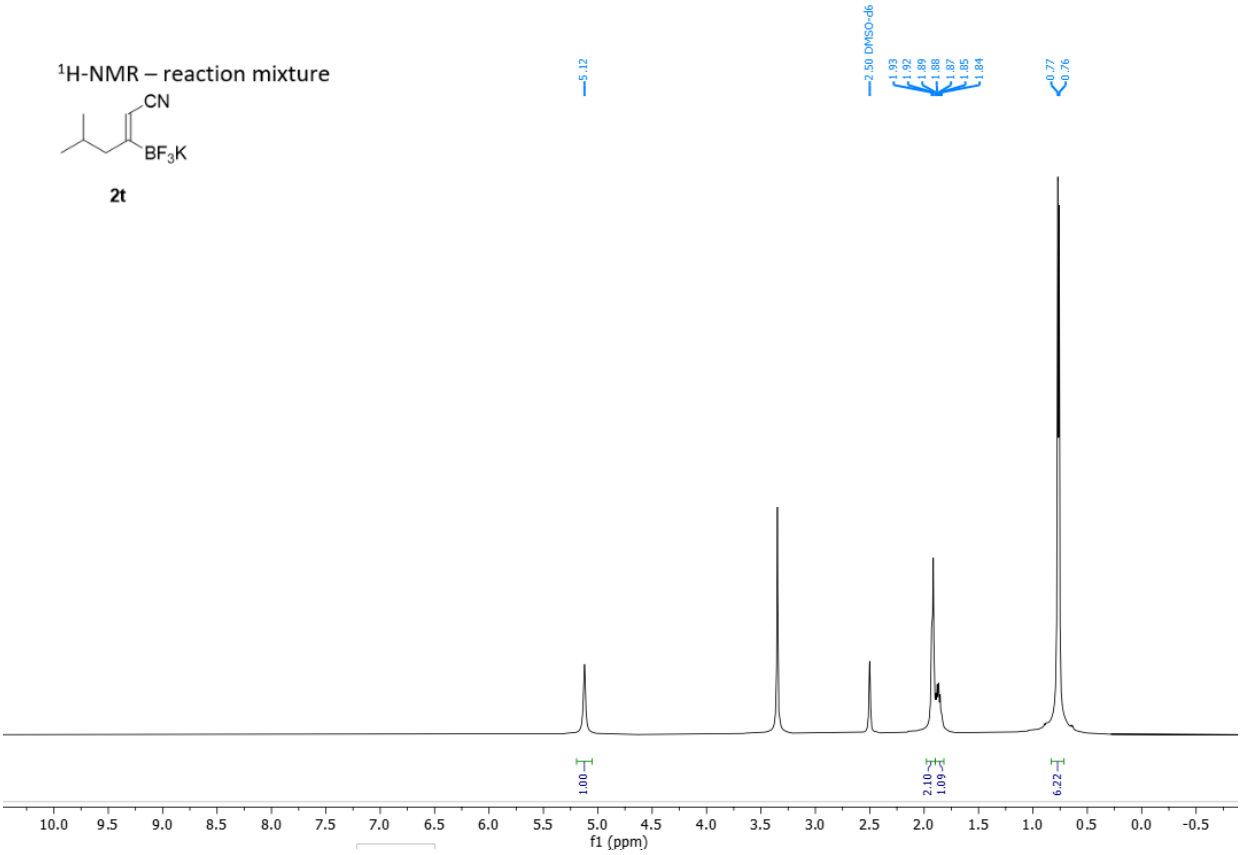

$^{13}C\{^{1}H\}$-NMR (126 MHz, DMSO-d_6) of **2t**:

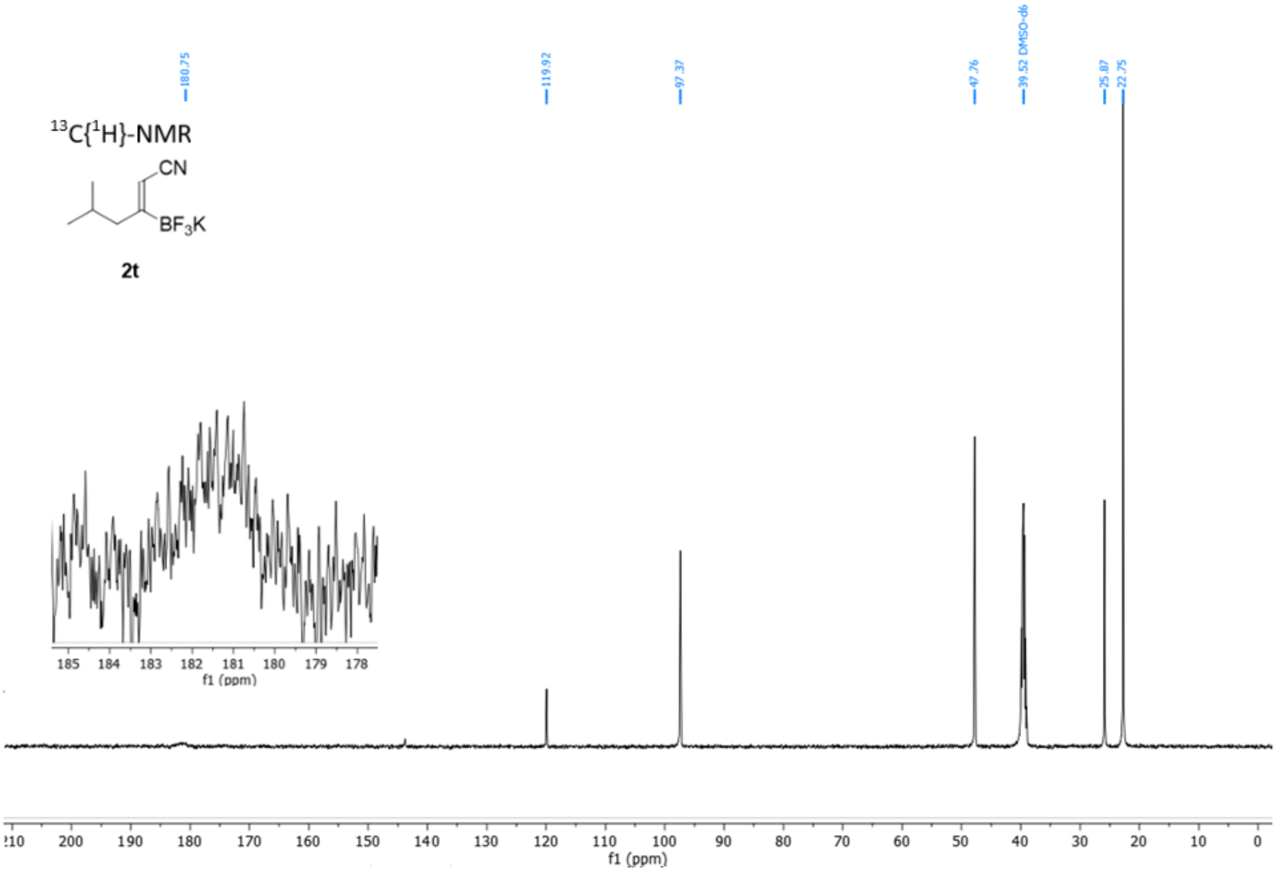

^{11}B-NMR (160 MHz, DMSO-d_6) of **2t**:

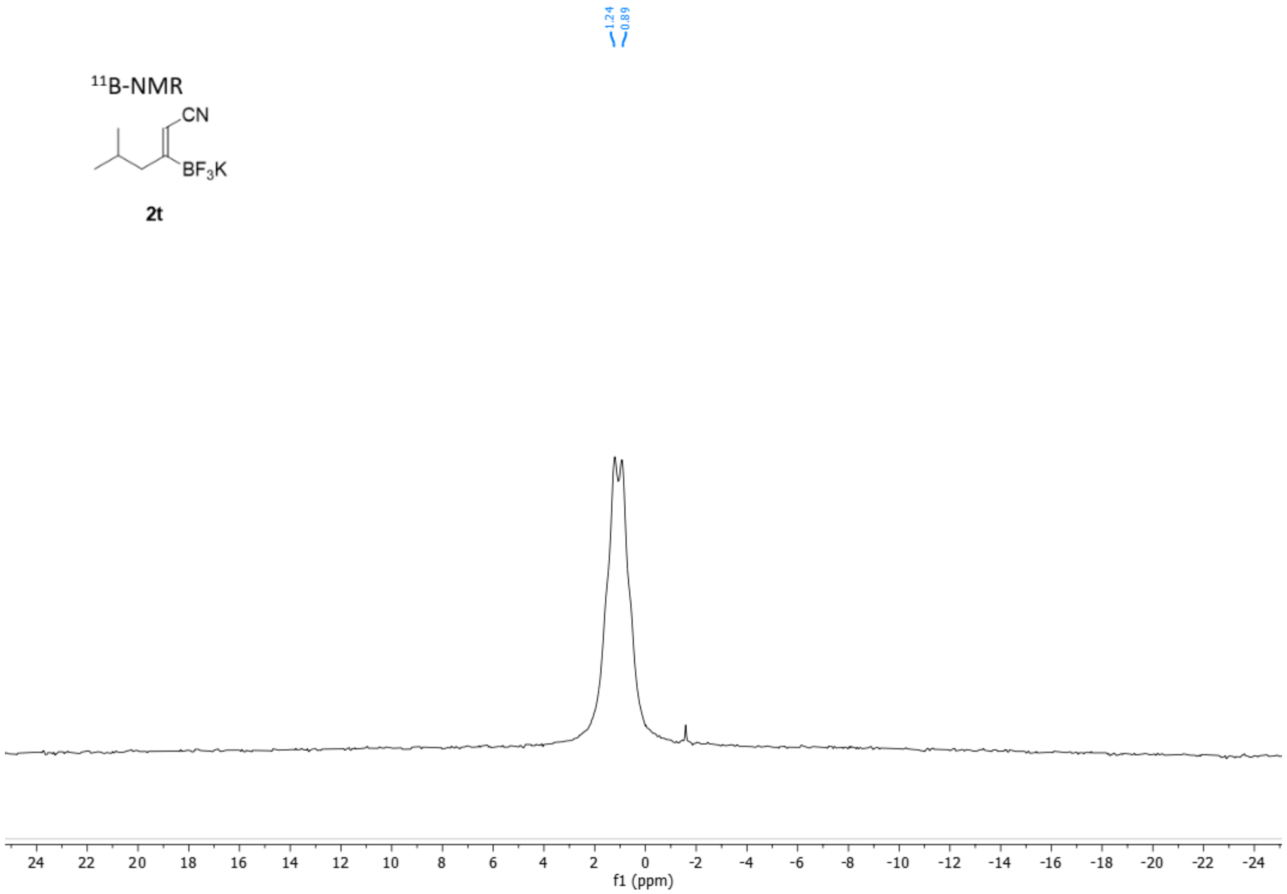

^{19}F-NMR (470 MHz, DMSO-d_6) of **2t**:

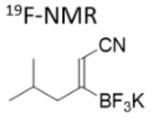

2t

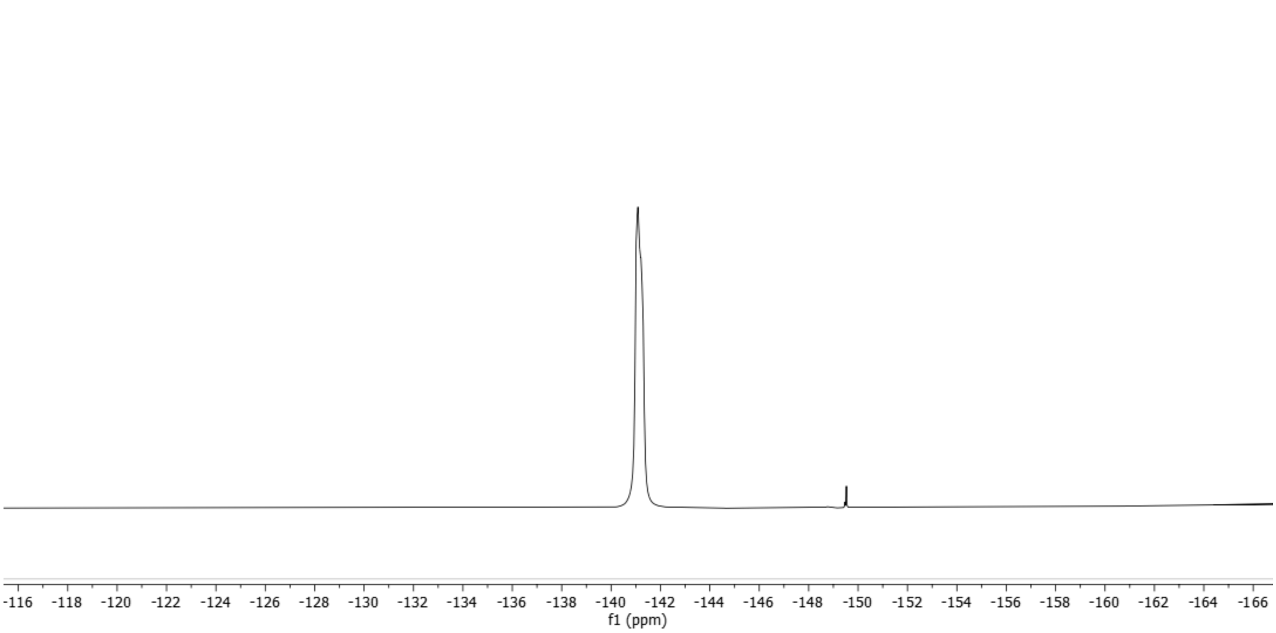

(*E*)-3-cyclohexyl-3-(trifluoro-λ^4-boraneyl)acrylonitrile, potassium salt (2v)

^{1}H-NMR (500 MHz, DMSO-d_6) of the reaction mixture of **2v**:

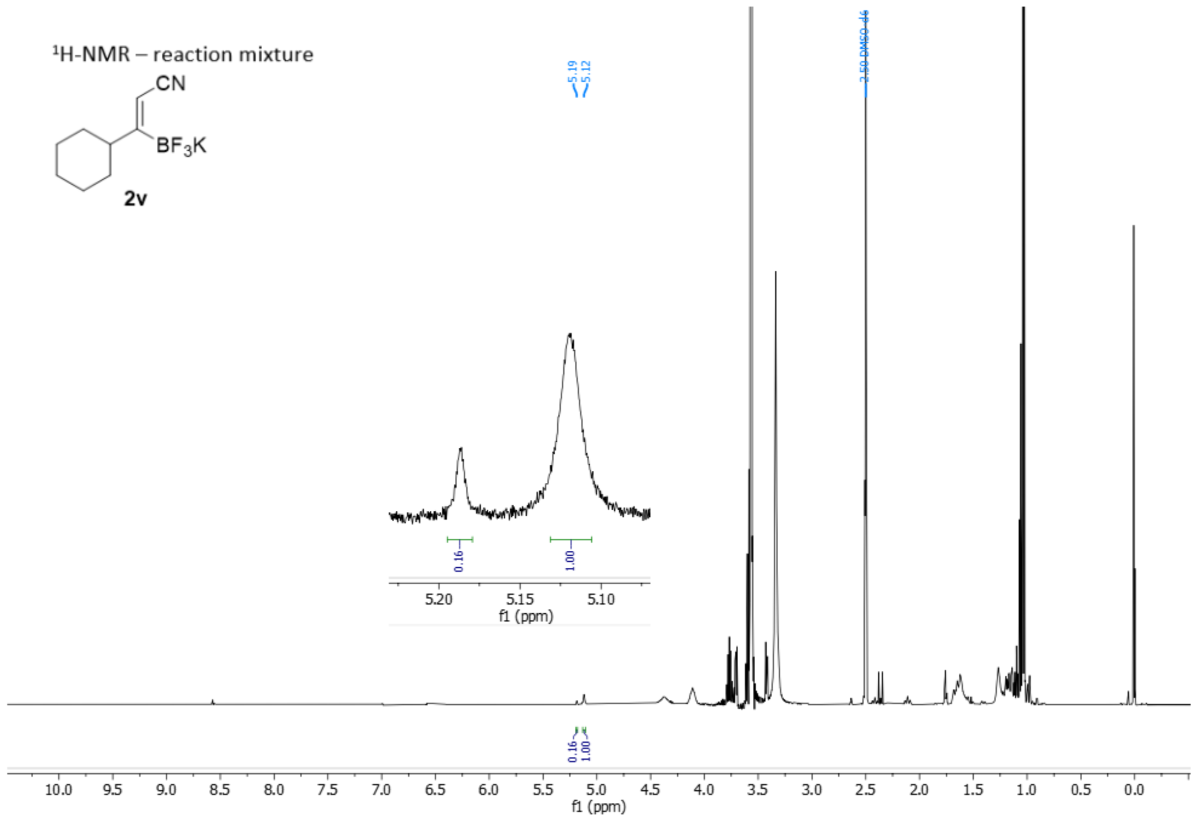

^{1}H-NMR (500 MHz, DMSO-d_6) of **2v**:

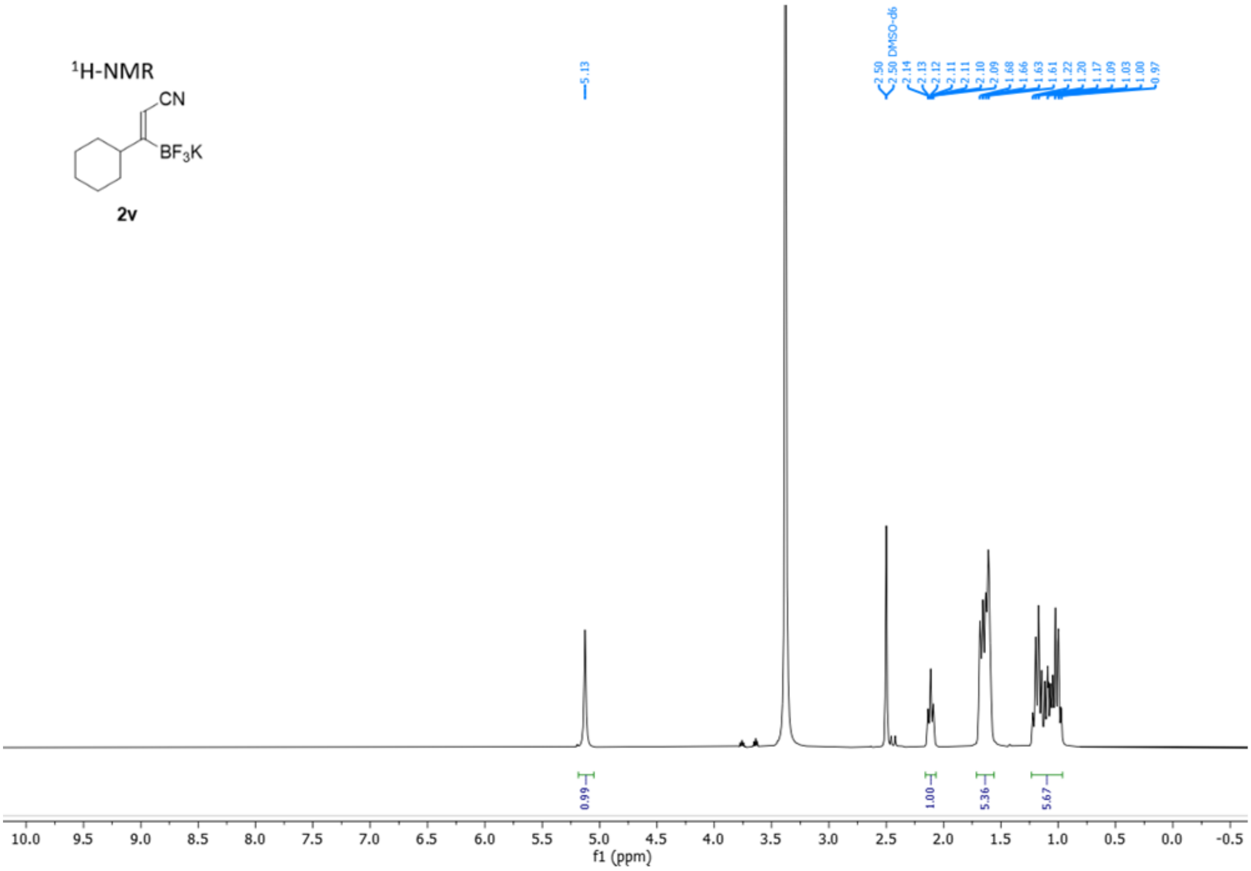

$^{13}C\{^1H\}$-NMR (126 MHz, DMSO-d_6) of **2v**:

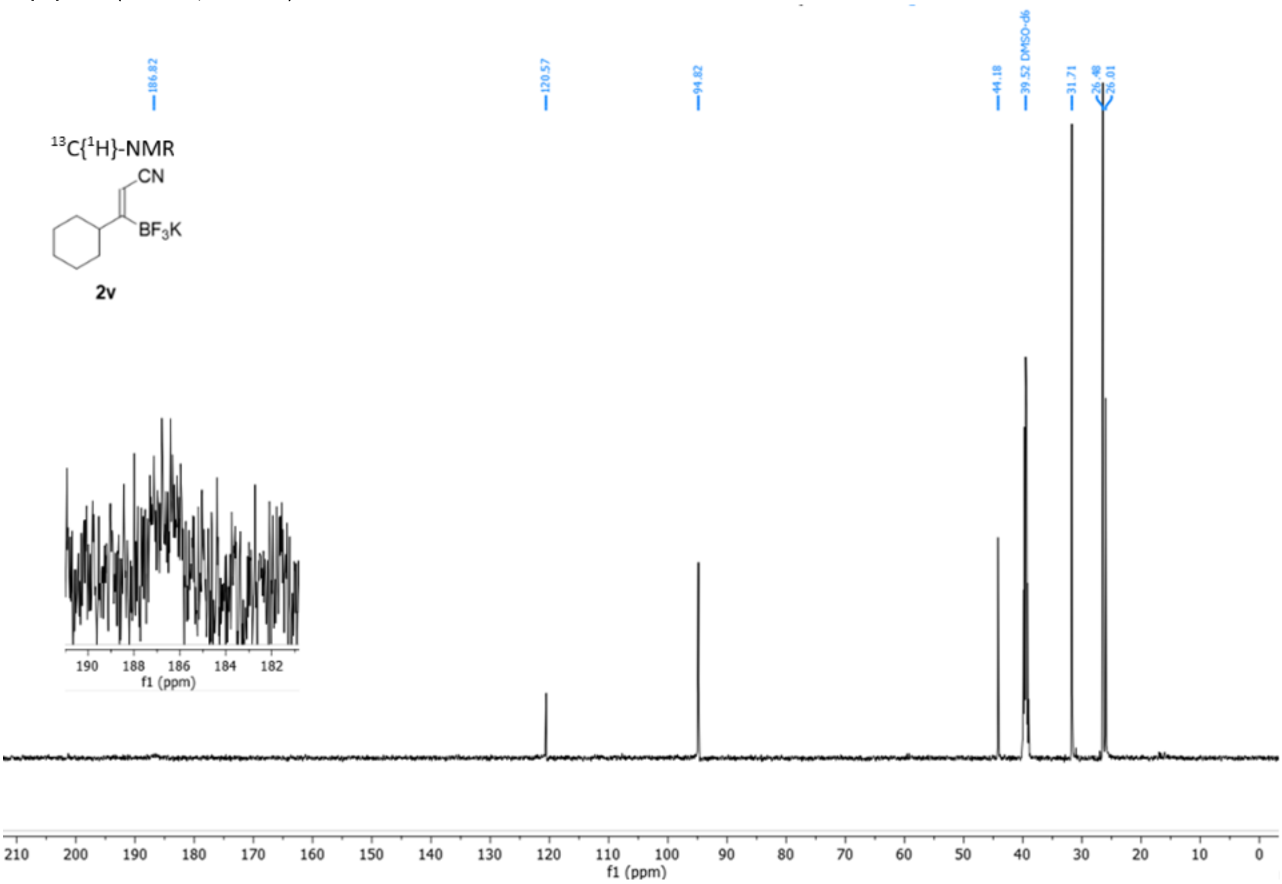

^{11}B-NMR (160 MHz, DMSO-d_6) of **2v**:

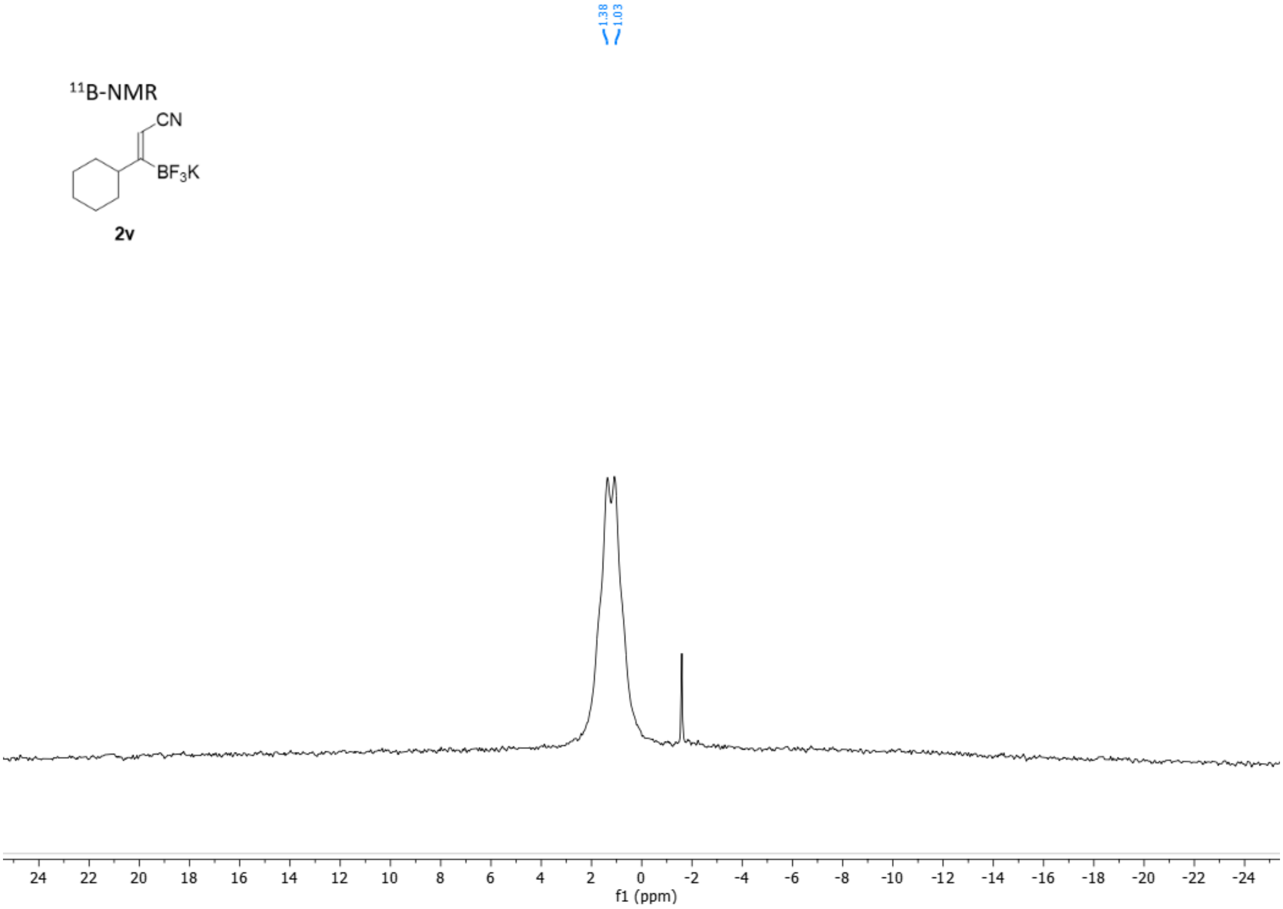

^{19}F-NMR (470 MHz, DMSO-d_6) of **2v**:

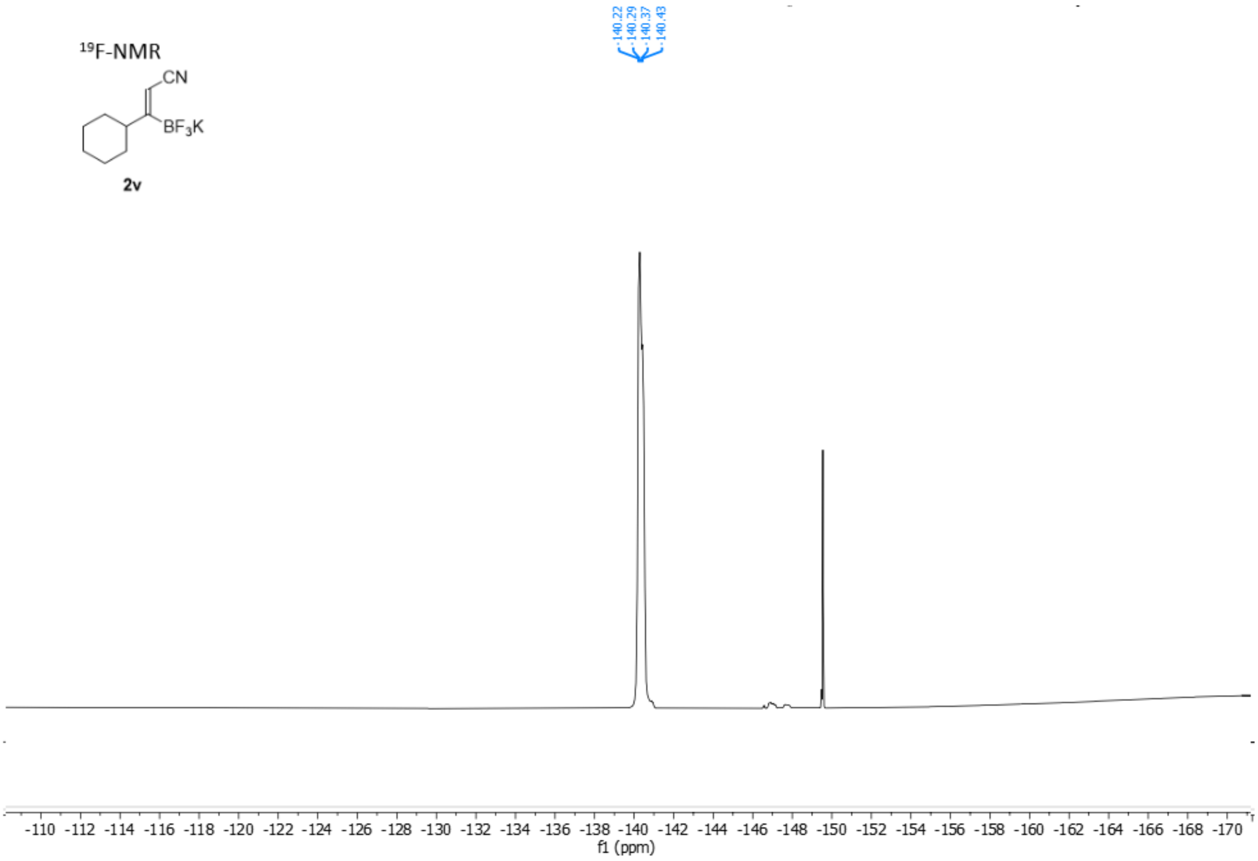

(*E*)-3-cyclopentyl-3-(trifluoro-λ4-boraneyl)acrylonitrile, potassium salt (2w)

^{1}H-NMR (500 MHz, DMSO-d_6) of the reaction mixture of **2w**:

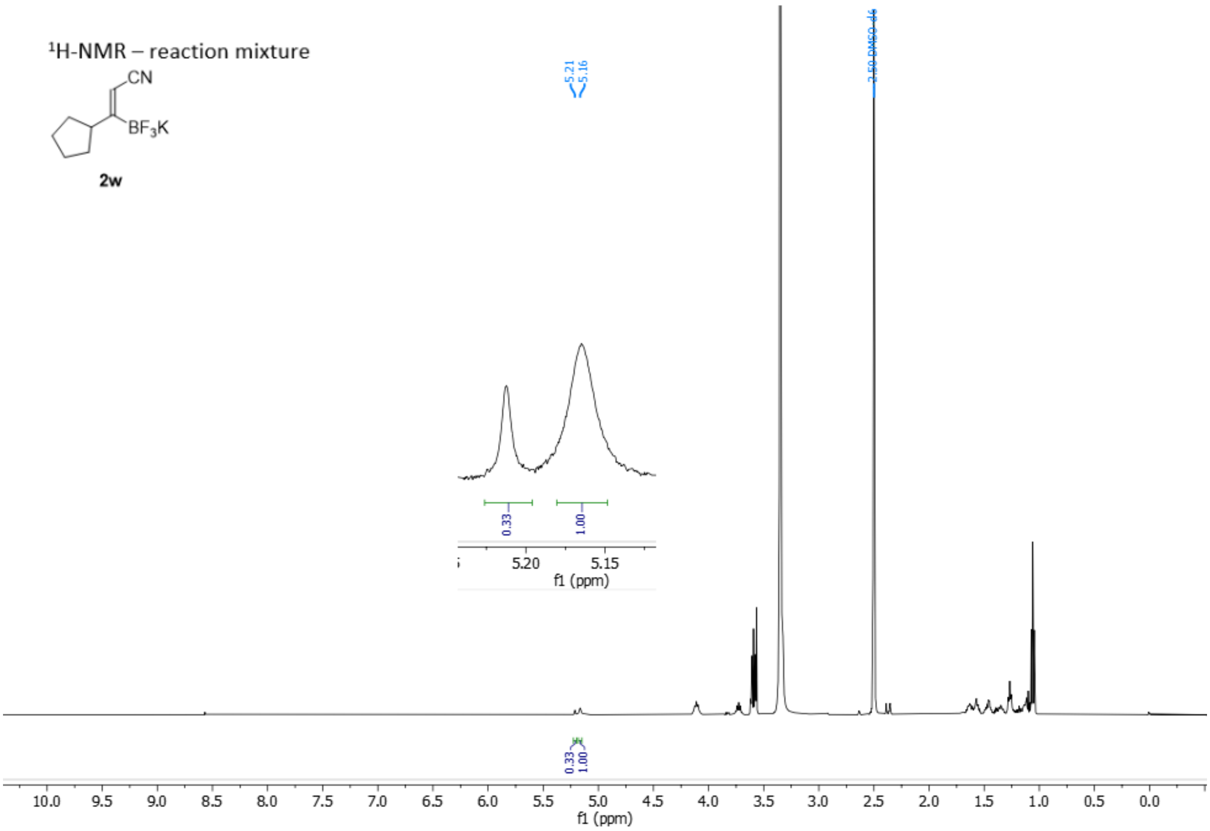

^{1}H-NMR (500 MHz, DMSO-d_6) of **2w**:

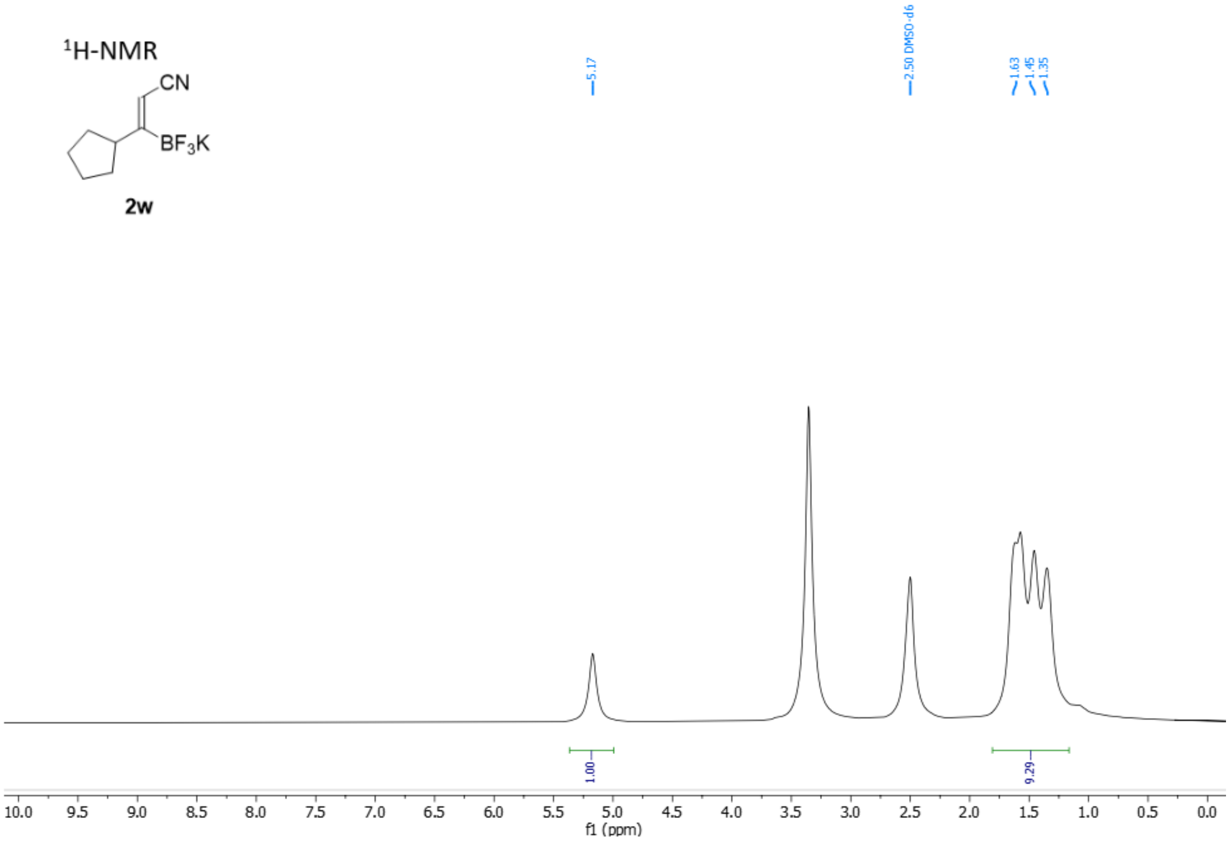

$^{13}C\{^1H\}$-NMR (126 MHz, DMSO-d_6) of **2w**:

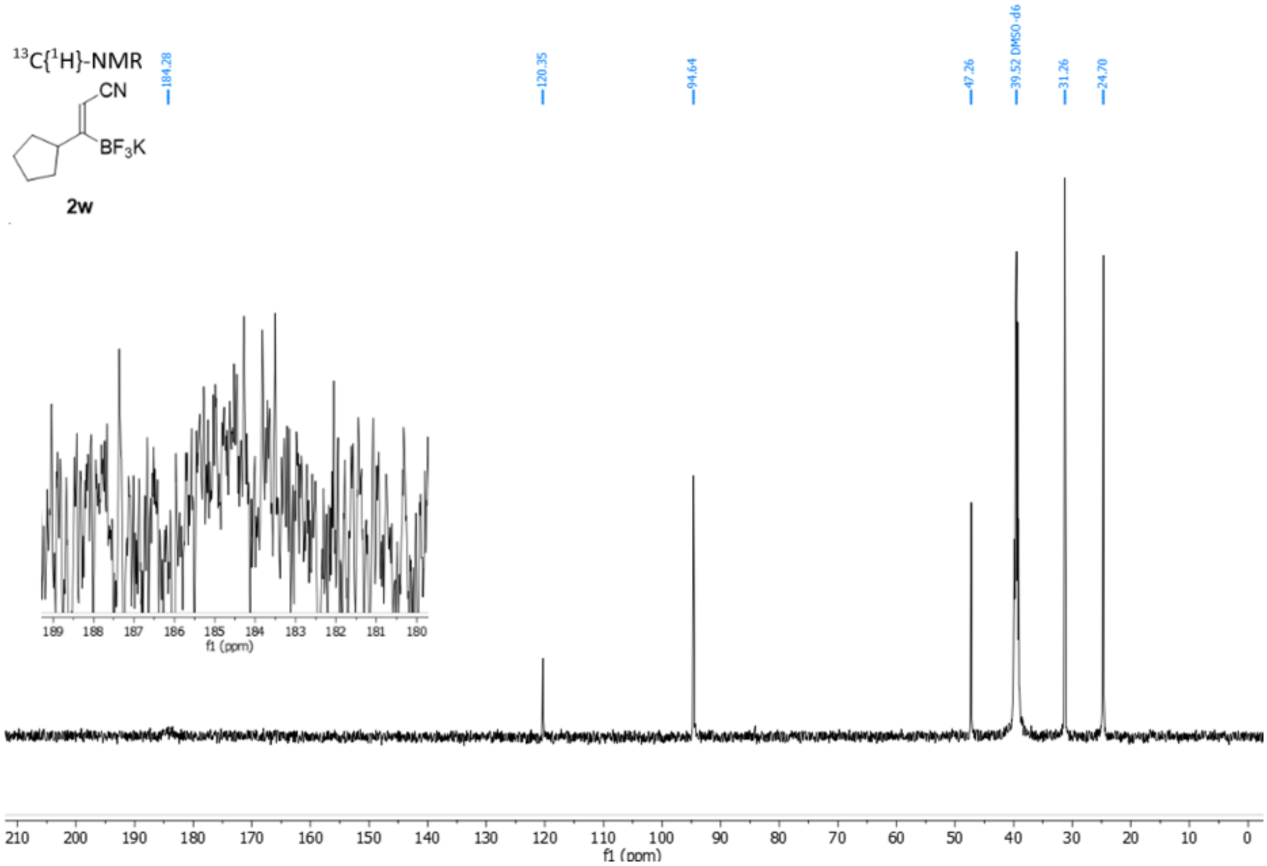

^{11}B-NMR (160 MHz, DMSO-d_6) of **2w**:

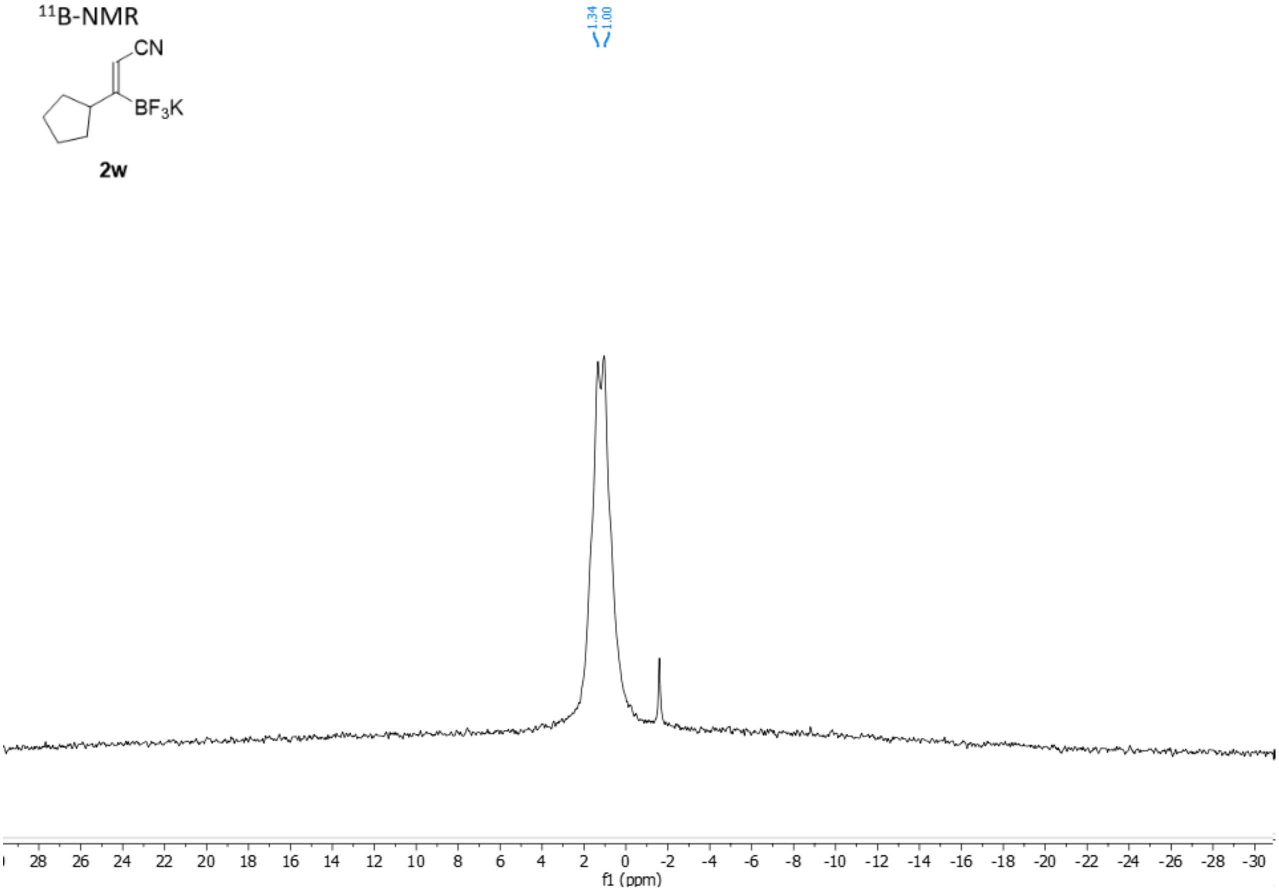

^{19}F-NMR (470 MHz, DMSO-d_6) of **2w**:

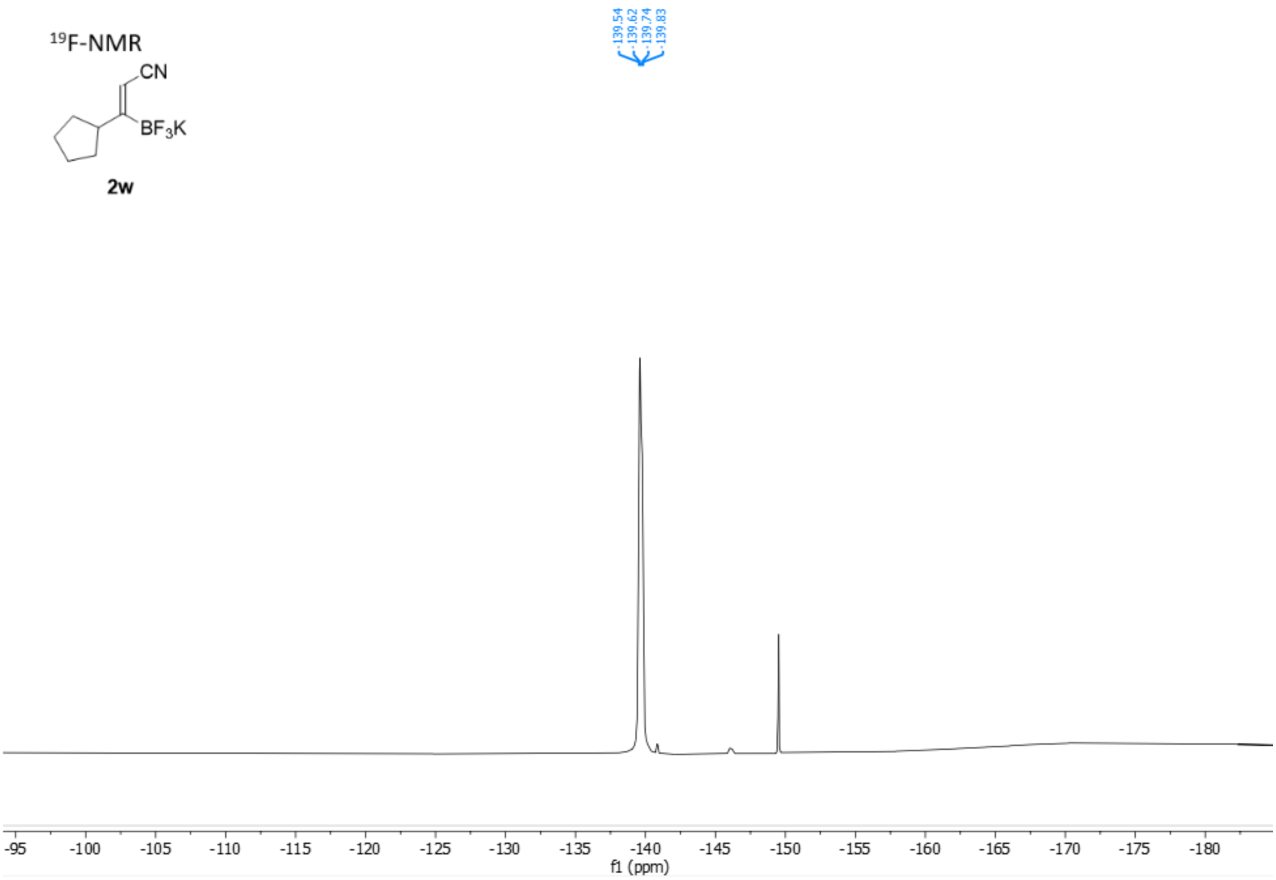

(*E*)-3-cyclopropyl-3-(trifluoro-λ[4]-boraneyl)acrylonitrile, potassium salt (2x)

^{1}H-NMR (500 MHz, DMSO-d_6) of the reaction mixture of **2x**:

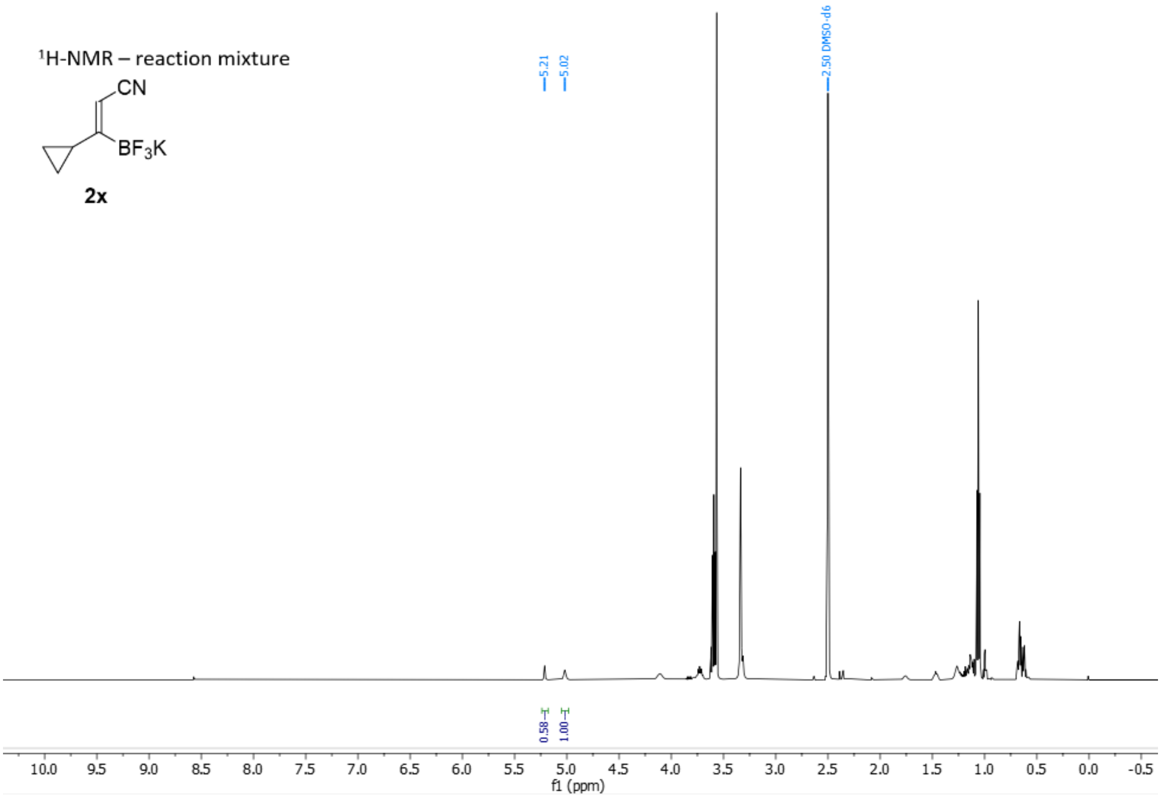

^{1}H-NMR (500 MHz, DMSO-d_6) of **2x**:

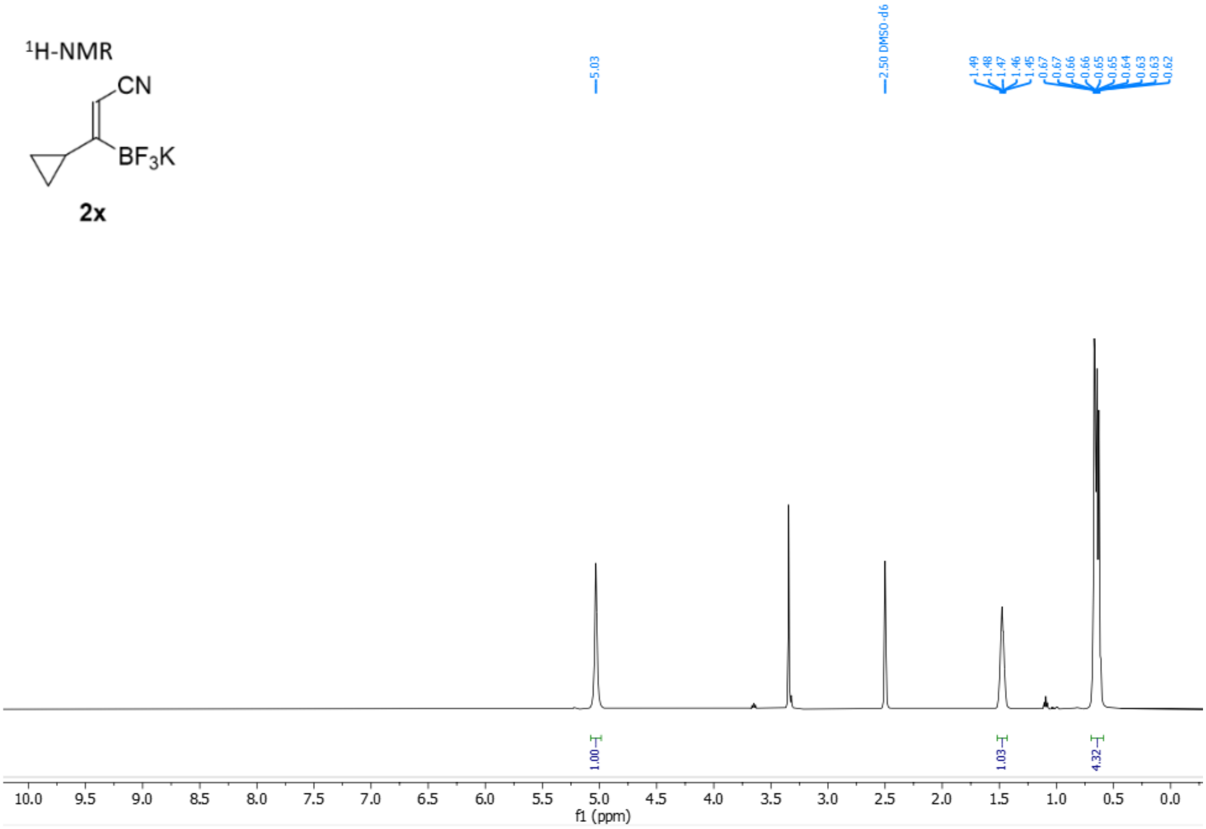

$^{13}C\{^{1}H\}$-NMR (126 MHz, DMSO-d_6) of **2x**:

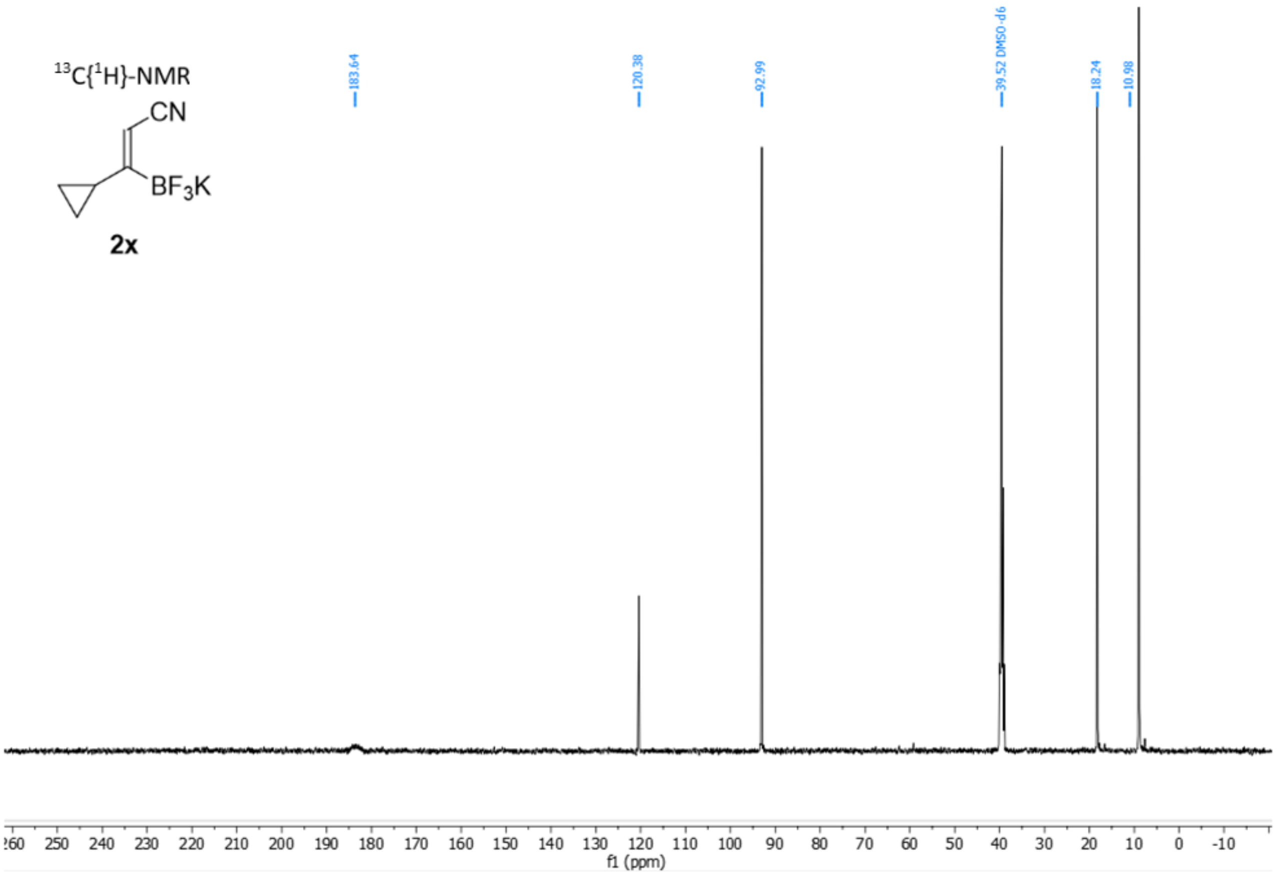

^{11}B-NMR (160 MHz, DMSO-d_6) of **2x**:

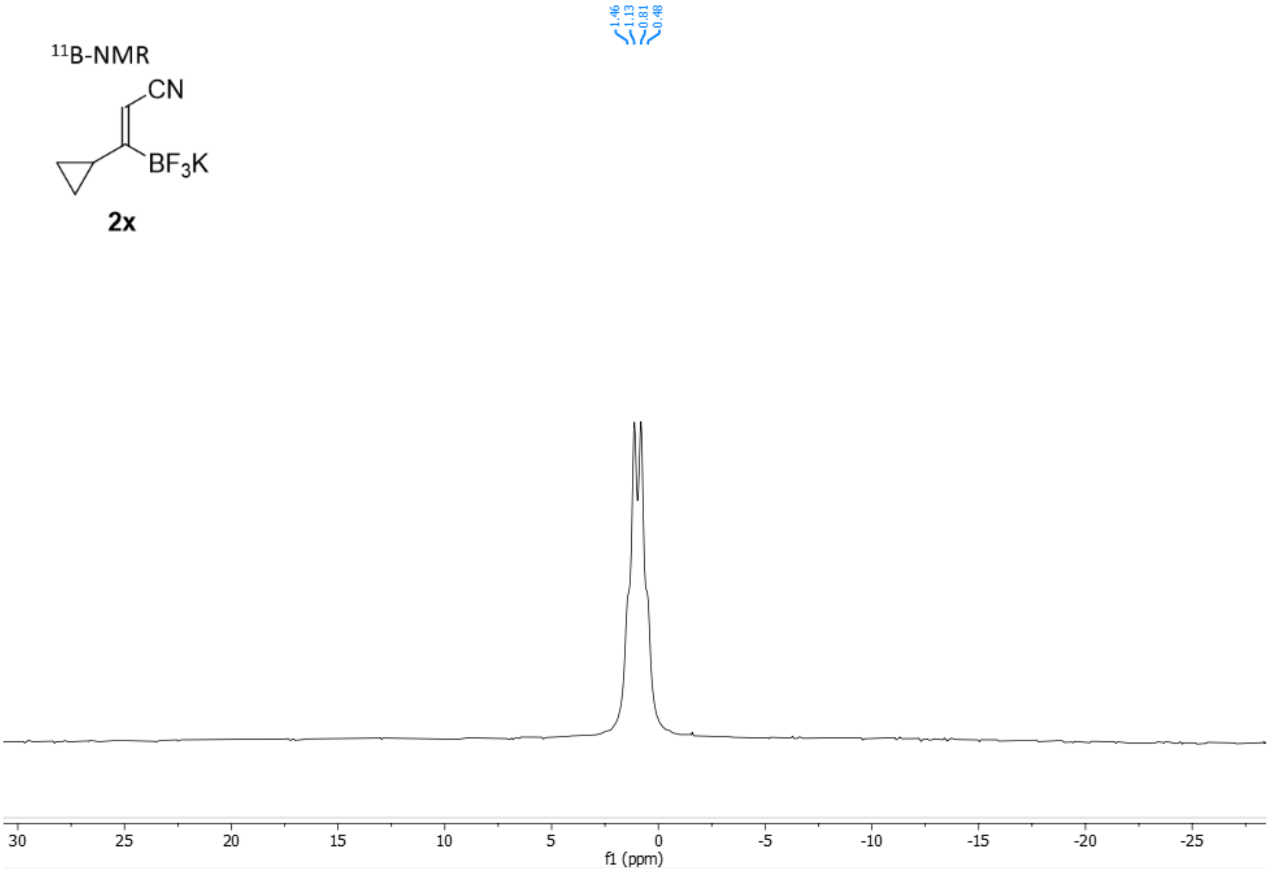

^{19}F-NMR (470 MHz, DMSO-d_6) of **2x**:

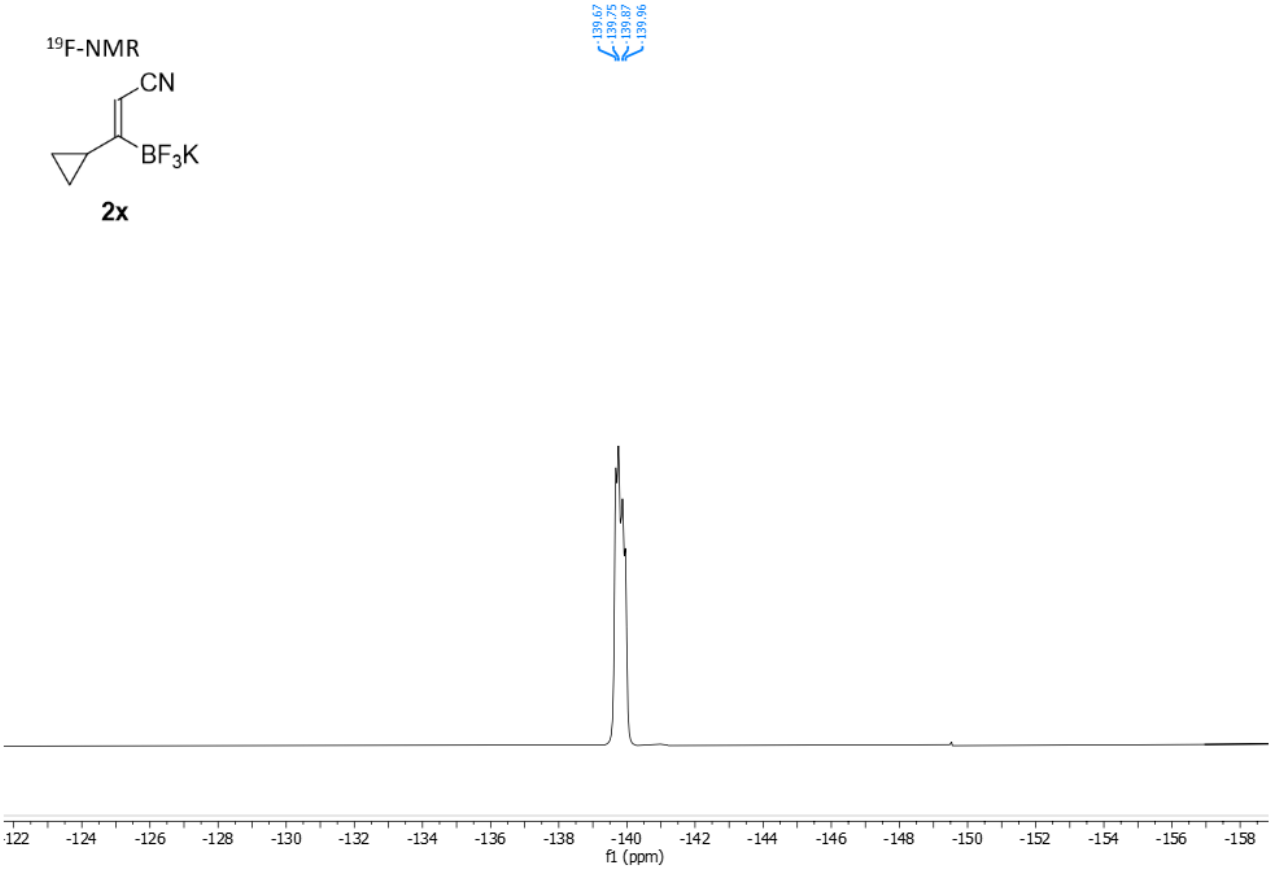

3-(4-bromophenyl)-3-oxopropanenitrile (3a)

^{1}H-NMR (500 MHz, $CDCl_3$) of **3a**:

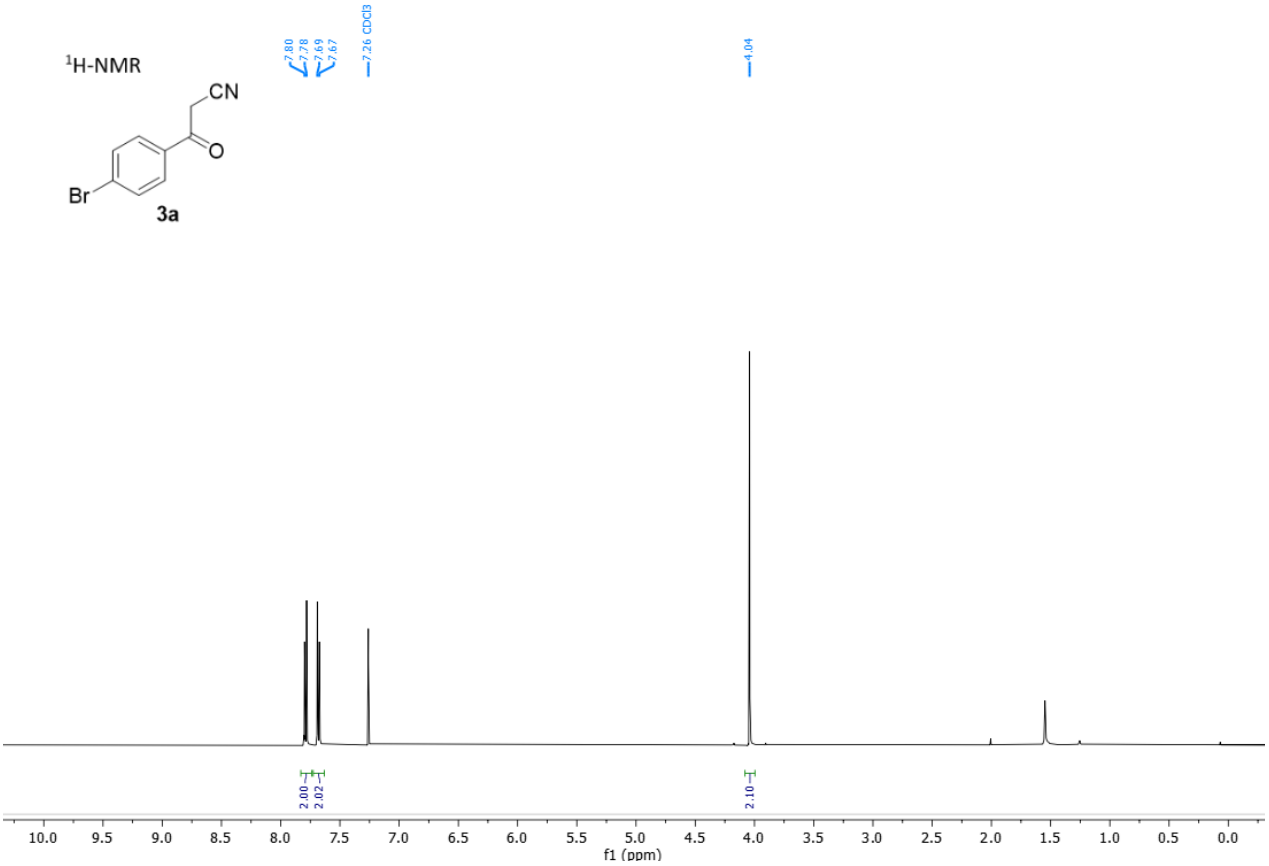

$^{13}C\{^1H\}$-NMR (126 MHz, $CDCl_3$) of **3a**:

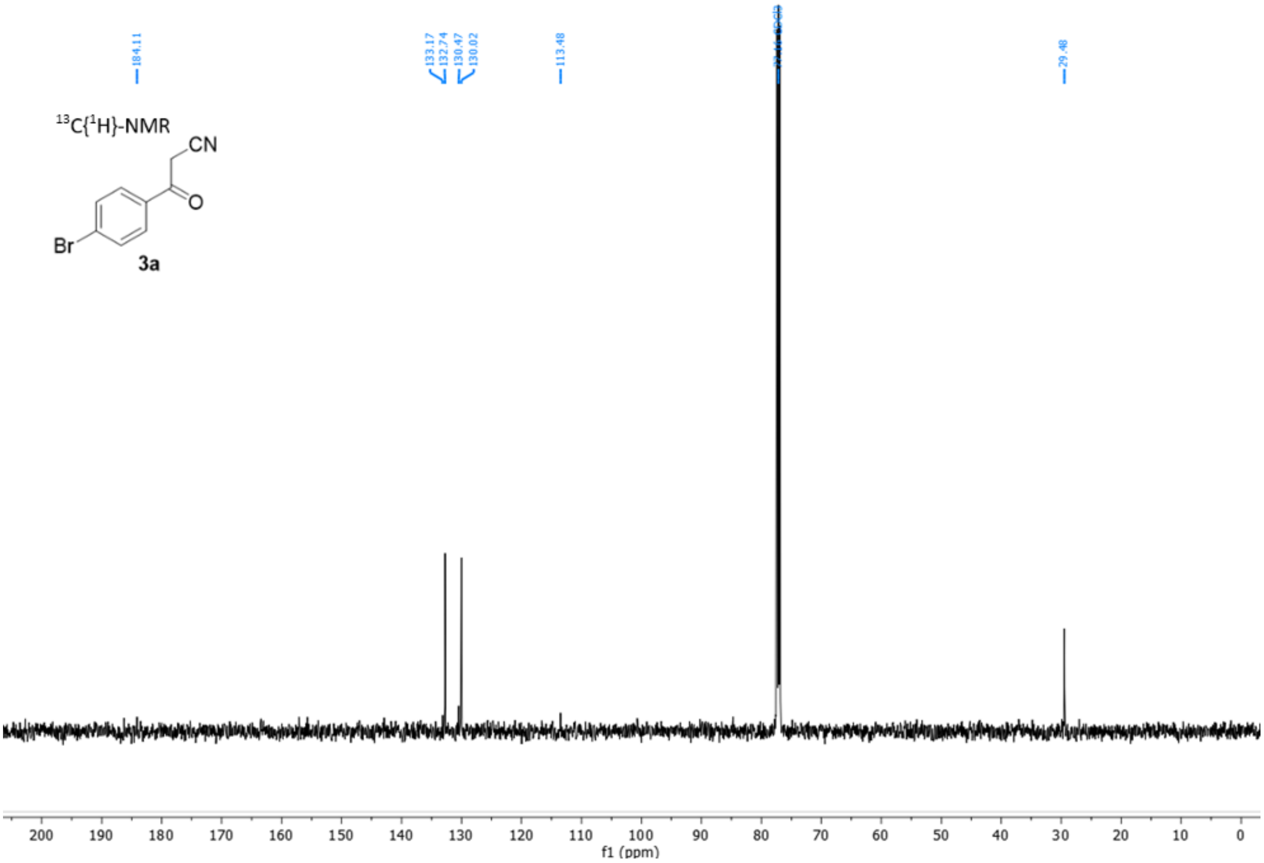

(*E*)-3-phenyl-3-(4,4,5,5-tetramethyl-1,3,2-dioxaborolan-2-yl)acrylonitrile (4b)

^{1}H-NMR (500 MHz, $CDCl_3$) of **4b**:

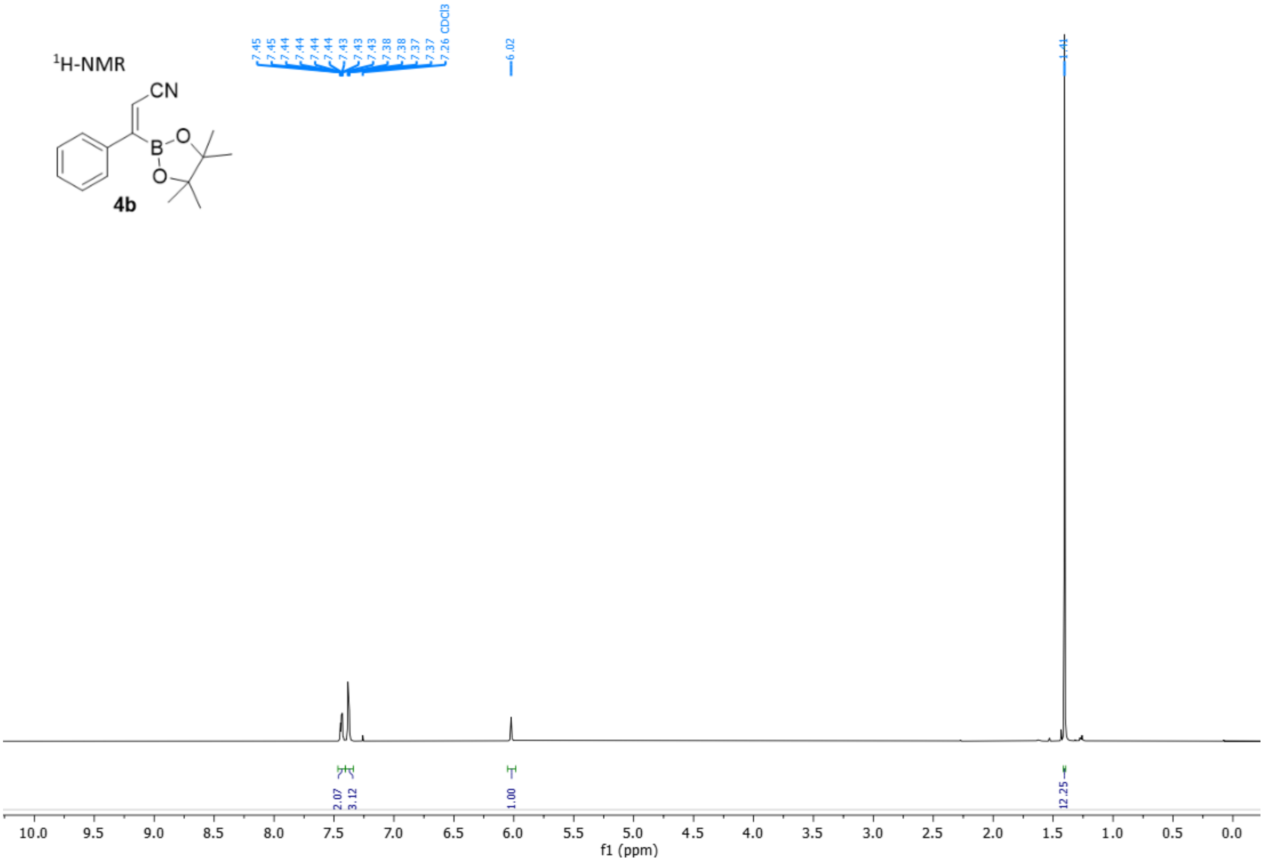

$^{13}C\{^{1}H\}$-NMR (126 MHz, $CDCl_3$) of **4b**:

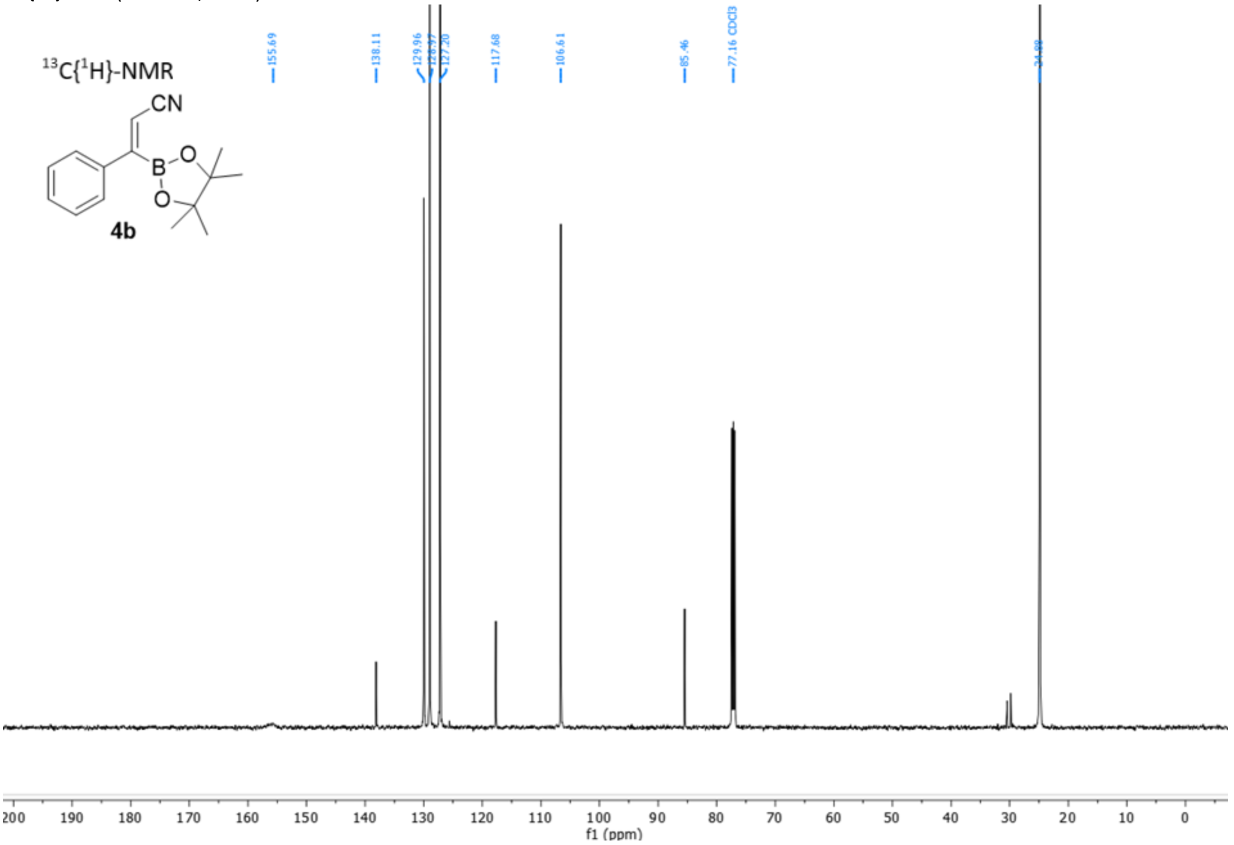

^{11}B-NMR (160 MHz, DMSO-d_6) of **4b**:

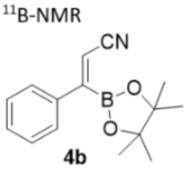

4b

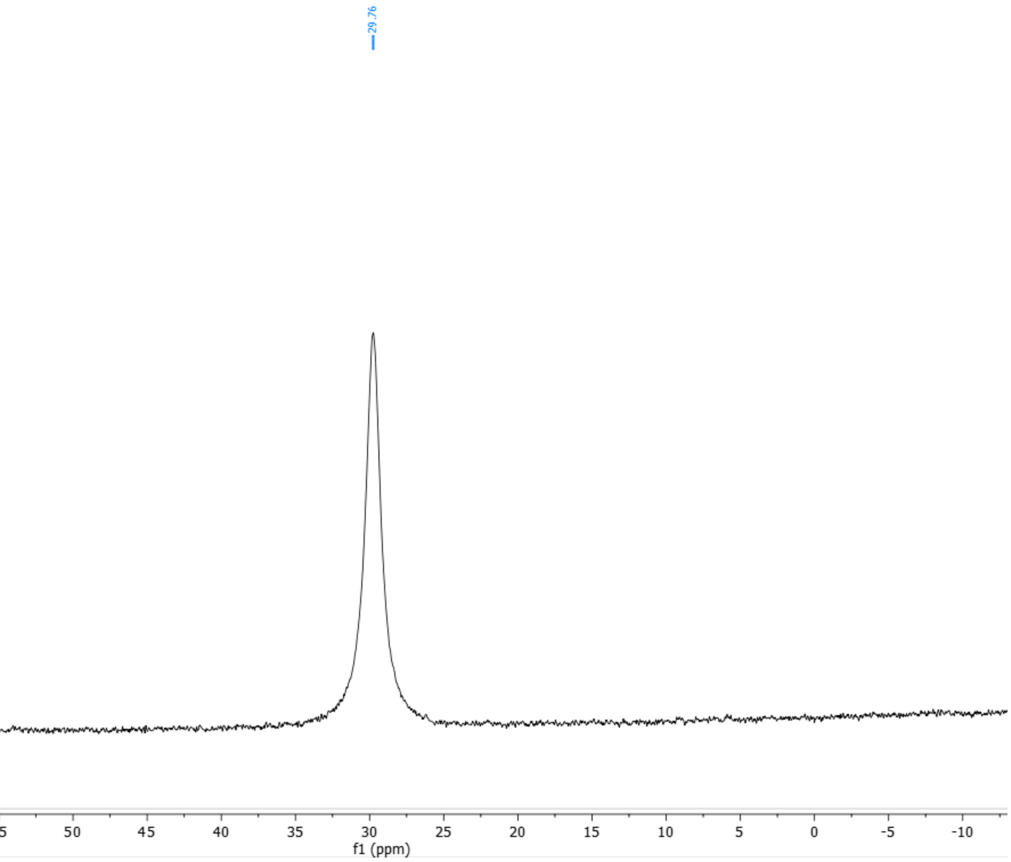

3,3-diphenylacrylonitrile (5b)

^{1}H-NMR (500 MHz, $CDCl_3$) of **5b**:

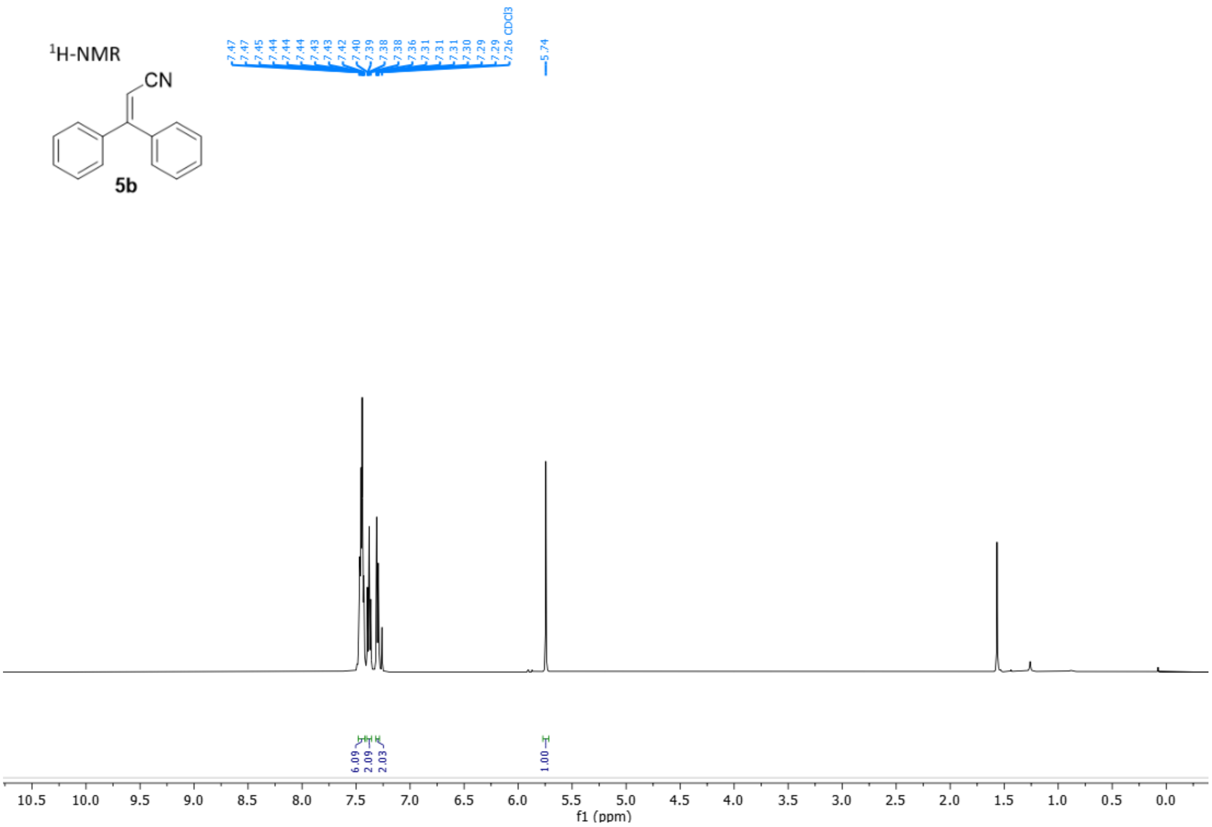

$^{13}C\{^{1}H\}$-NMR (126 MHz, $CDCl_3$) of **5b**:

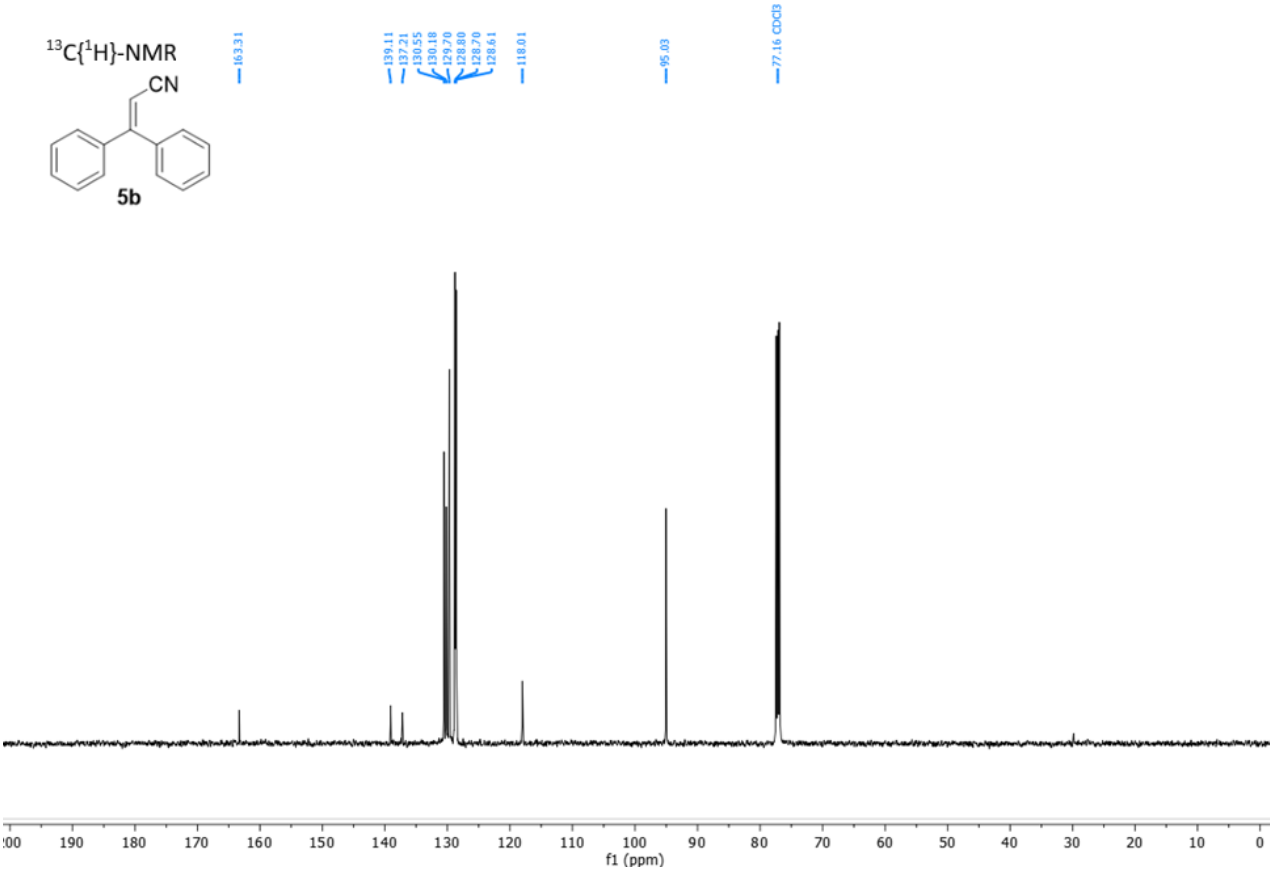

Ethyl (E)-3-(4-bromophenyl)-3-(trifluoro-l4-boraneyl)acrylate, potassium salt (6a)

^{1}H-NMR (500 MHz, DMSO-d_6) of the reaction mixture of **6a**:

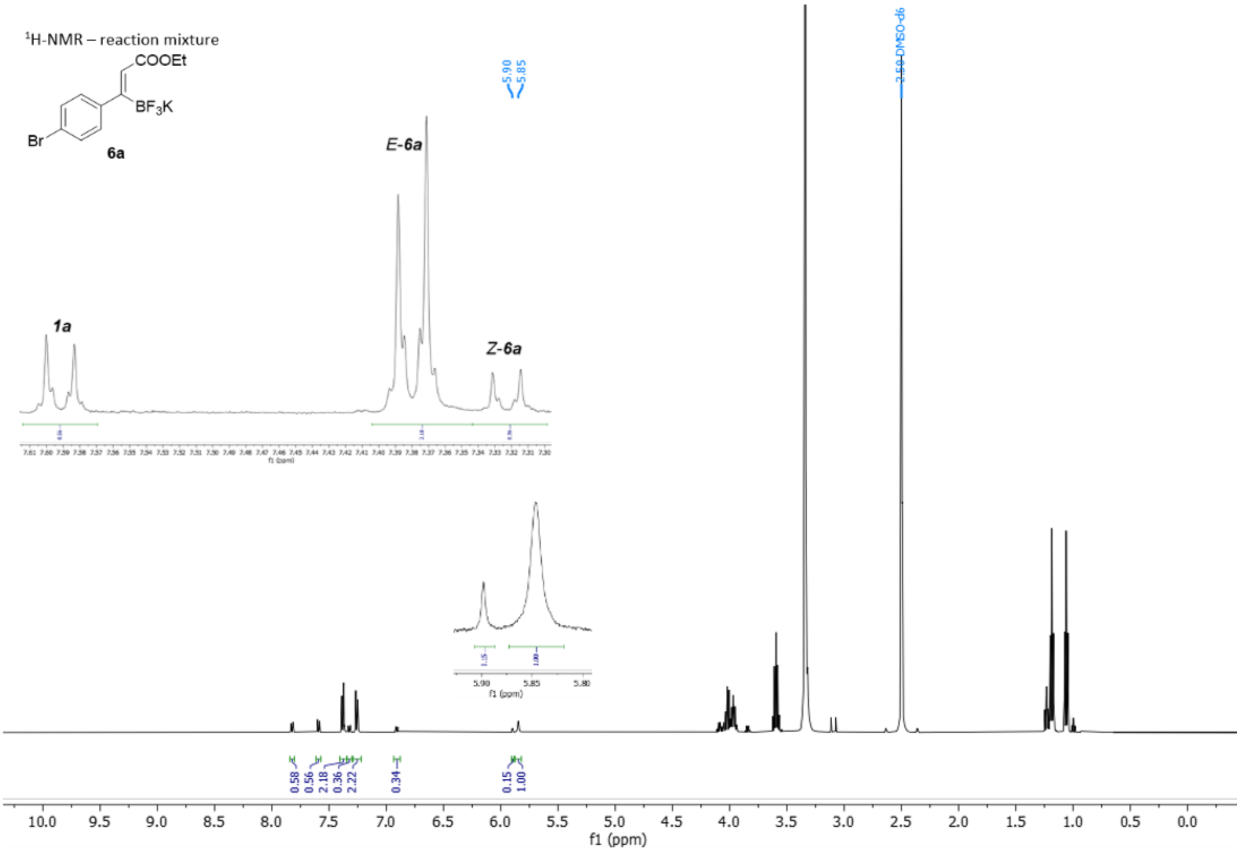

^{1}H-NMR (500 MHz, DMSO-d_6) of **6a**:

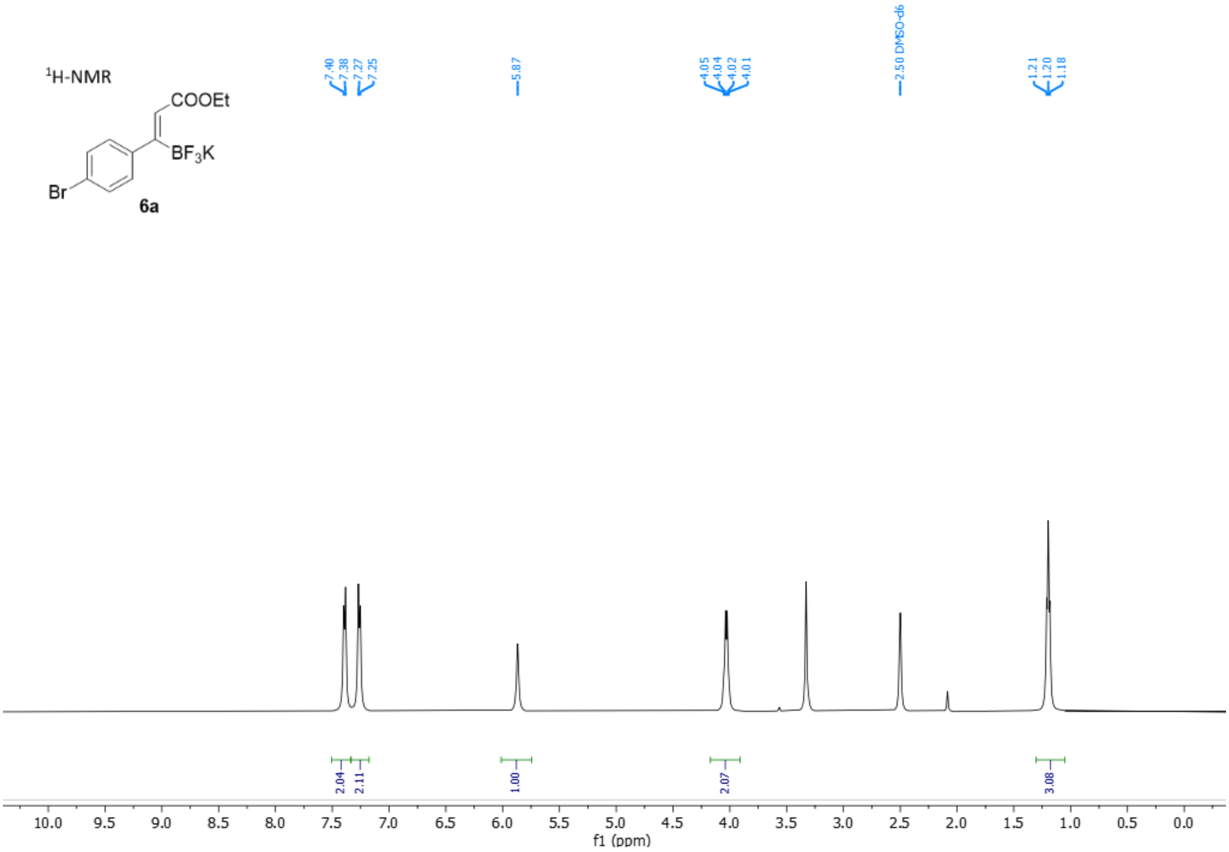

$^{13}C\{^{1}H\}$-NMR (126 MHz, DMSO-d_6) of **6a**:

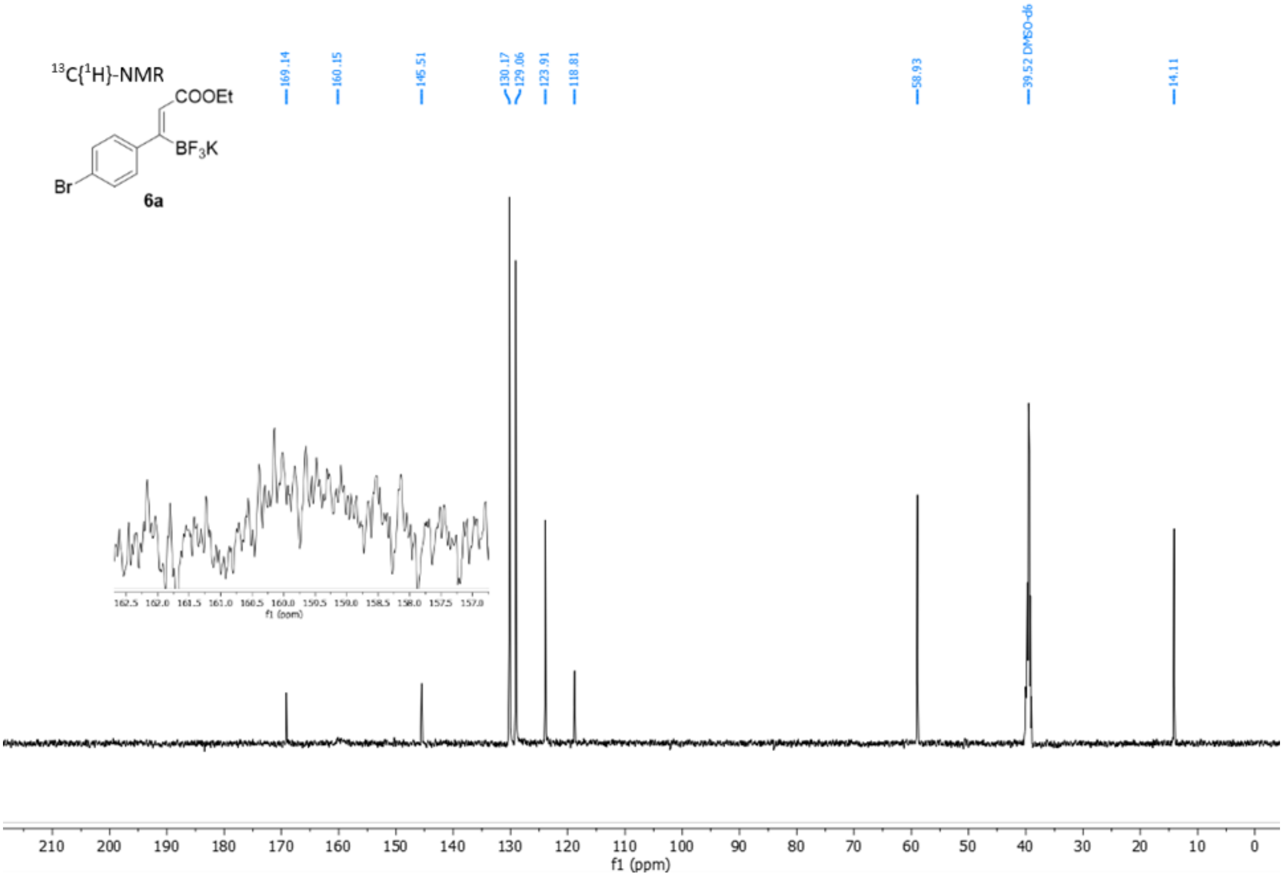

^{11}B-NMR (160 MHz, DMSO-d_6) of **6a**:

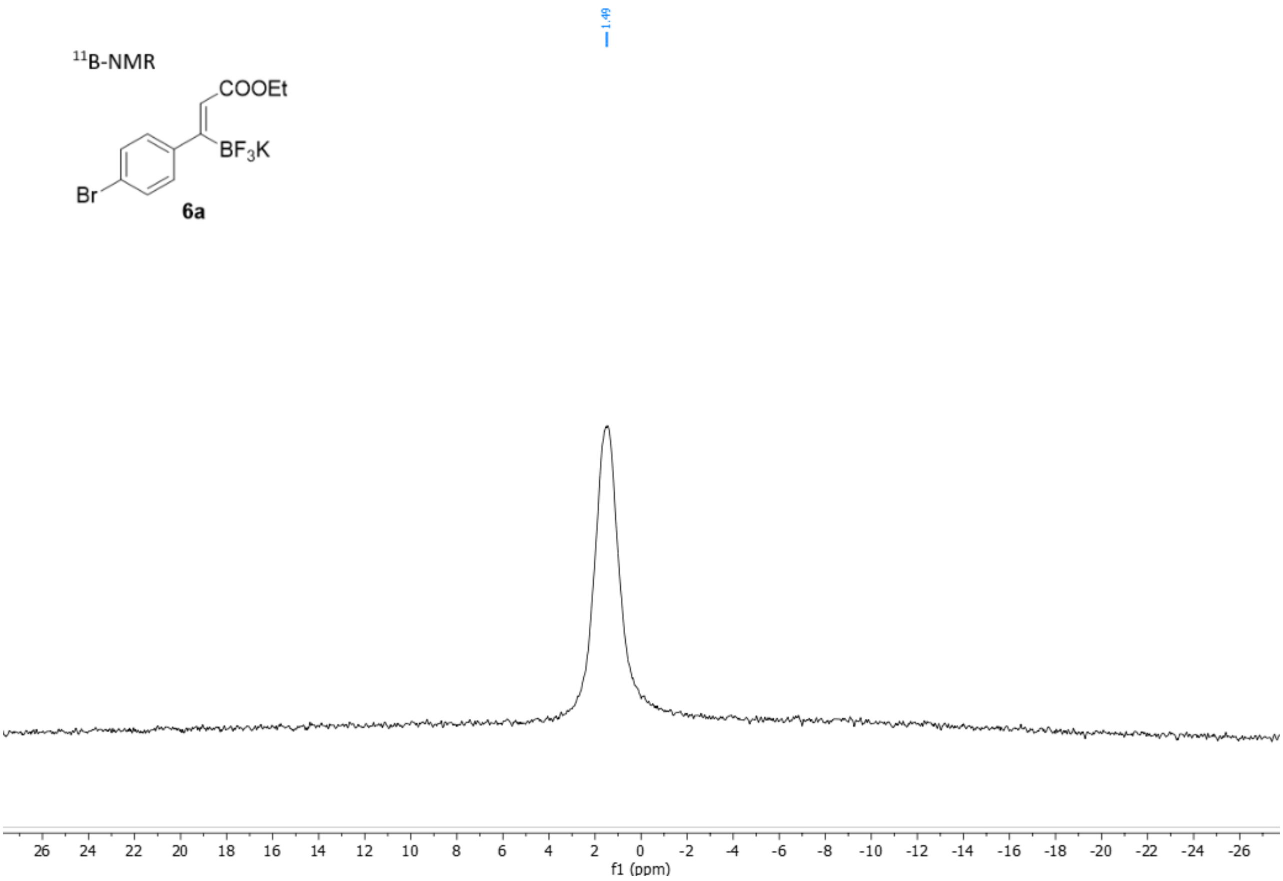

^{19}F-NMR (470 MHz, DMSO-d_6) of **6a**:

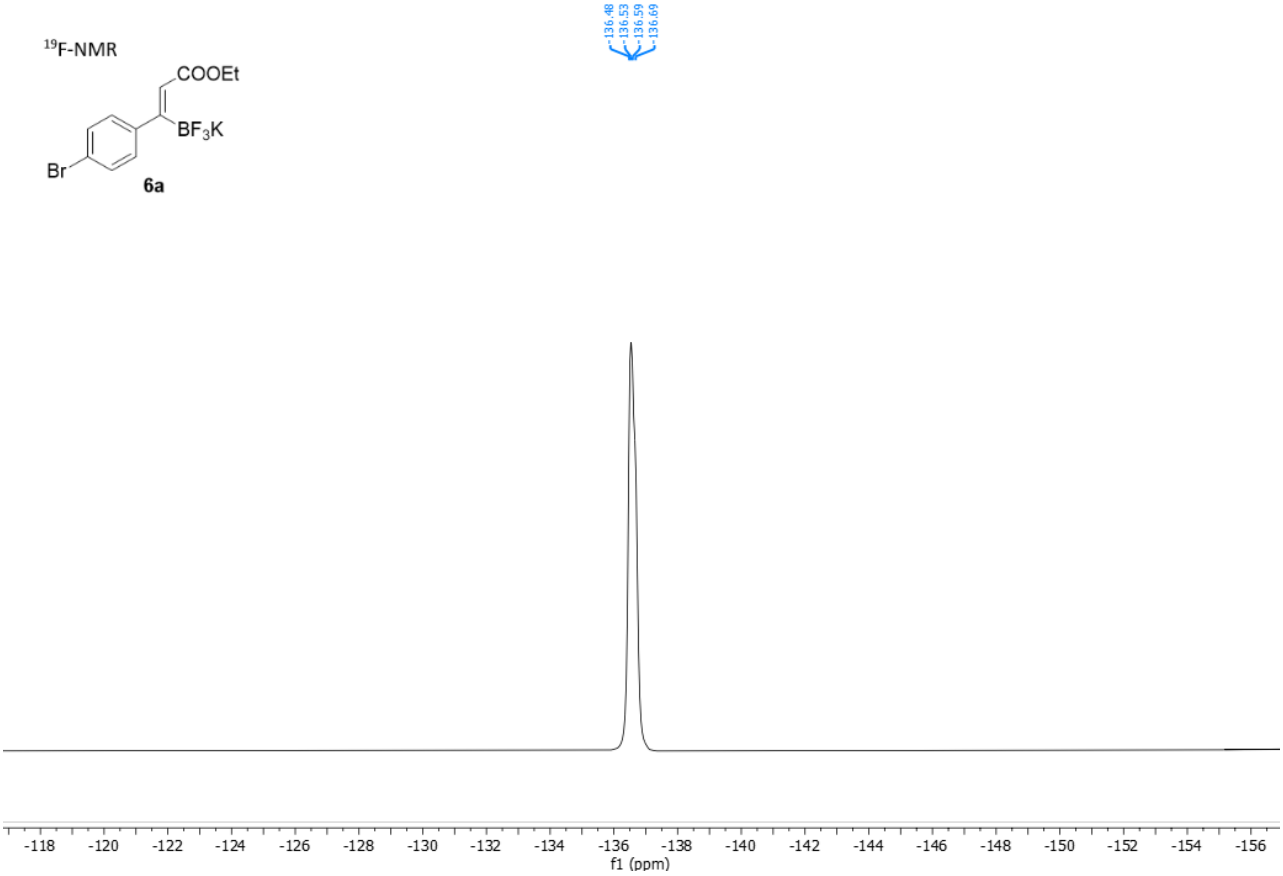

Supplement: Supplementary file 1 [file jo4c02915_si_001.pdf]
